# MicroRNA-200b/c-3p regulate epithelial plasticity and inhibit cutaneous wound healing by modulating TGF-β-mediated RAC1 signaling

**DOI:** 10.1038/s41419-020-03132-2

**Published:** 2020-10-29

**Authors:** Huiyi Tang, Xueer Wang, Min Zhang, Yuan Yan, Simin Huang, Jiahao Ji, Jinfu Xu, Yijia Zhang, Yongjie Cai, Bobo Yang, Wenqi Lan, Mianbo Huang, Lin Zhang

**Affiliations:** Department of Histology and Embryology, Guangdong Provincial Key Laboratory of Construction and Detection in Tissue Engineering, School of Basic Medical Sciences, Southern Medical University, Guangzhou, Guangdong People’s Republic of China

**Keywords:** RNA, RHO signalling

## Abstract

Cutaneous wound healing is pivotal for human skin to regain barrier function against pathogens. MicroRNAs (miRNAs) have been found to play regulatory roles in wound healing. However, the mechanism of miRNA regulation remains largely unknown. In this study, we focused on microRNA-200b/c-3p (miR-200b/c-3p) whose expression was abundant in intact epidermis, but dramatically decreased in skin wounds. In silico prediction identified RAC1 as a potential miR-200b/c-3p target. Luciferase reporter assay confirmed that miR-200b/c-p repressed RAC1 by direct targeting to its mRNA 3′UTR. Consistently, miR-200b/c-3p expression was discordantly related to RAC1 protein level during wound healing. Forced miR-200b/c-3p expression repressed RAC1 and inhibited keratinocyte migration as well as re-epithelialization in a mouse back skin full-thickness wound healing model. Mechanistically, miR-200b/c-3p modulated RAC1 to inhibit cell migration by repressing lamellipodia formation and intercellular adhesion dissolution in keratinocytes. Furthermore, we found that TGF-β1, which was highly expressed in skin wounds, contributed to the downregulation of miR-200b/c-3p in wound edge keratinocytes. Taken together, miR-200b/c-3p-mediated RAC1 repression inhibited keratinocyte migration to delay re-epithelialization. TGF-β1 induction attenuated miR-200b/c-3p regulation of RAC1 signaling in cutaneous wounds and the repression of miR-200b/c-3p accelerated keratinocyte migration to promote wound healing. Our data provide new insight into how miR-200b/c-3p affects keratinocyte migration and highlight the potential of miR-200b/c-3p targeting for accelerating wound healing.

## Introduction

Skin barrier integrity is central to protect the body from outside pathogens and keeping body fluid inside. Cutaneous wound healing is of significance to restore barrier function and integrity of injured skin. Impaired wound healing is harmful to human health or even life-threatening^1^. The healing process can be divided into three distinct but overlapping phases: the inflammatory, proliferation, and remodeling phase^2^. Wound re-epithelialization is the critical step of proliferation phase involving rapid regeneration of epidermis, and requires collective migration of keratinocytes in the wound edge^3^. Wound-edge keratinocytes reorganize their actin cytoskeletons to commence migration as an epithelial tongue between the wound scab and underlying granulation tissue^4^. Efficient cell migration in the wound margin is coordinated by lamellipodia formation in the forward direction, disassembly of actin stress fiber and lateral intercellular junction downregulation^5^.

The defining members of Rho guanosine triphosphatase (GTPase) family that include ras homolog family member A (RHOA), Rac family small GTPase 1 (RAC1), and cell division cycle 42 (CDC42), work in a coordinated fashion to regulate the actin cytoskeletal dynamics that control cell motility^5^. The homeostasis of normal tissues requires the regulation of actin cytoskeleton and adhesion properties, both of which are deeply influenced by Rho family GTPases and their effectors^6^. RHOA regulates the formation of stress fibers^7^, whereas CDC42 controls filopodia formation and epithelial intercellular junction^8^. RAC1 is responsible for driving actin polymerization to form lamellipodia at the leading edge of a migrating cell^9^, but appears to inhibit epidermal adherens junction through endocytosis of E-cadherin^10^. Impaired epidermal wound healing has been reported in mice when *Rac1* is knocked out or functionally inhibited by a dominant-negative mutant in the epidermis^11,12^. The activity of Rho-family members is typically regulated post-translationally by guanine nucleotide exchange factors (GEFs), GTPase-activating proteins (GAPs), and GDP dissociation inhibitors (GDIs), through modulating the GDP- or GTP-bound state switch^6^. Wound healing is impaired in mutant mice that lack the RAC1 associated GEF Tiam1, supporting the importance of RAC1 in promoting wound healing^13^. However, the mechanism of RAC1 regulation in epidermal wound repair is not fully elucidated.

MicroRNAs (miRNAs) are a family of ~22-nucleotides small RNAs that typically repress gene expression at the post-transcriptional level^14^. A group of miRNAs preferentially expressed in the epidermis have been found to play essential regulatory roles in skin development. miR-203 promotes epidermal differentiation by restricting proliferative potential and inducing cell-cycle exit^15,16^. miR-205 controls epidermal and hair follicle growth by governing the expansion of skin stem cells^17^. miR-24 regulates epithelial differentiation by controlling the actin cytoskeleton^18^. miR-200 regulates cell adhesion and proliferation in hair morphogenesis^19^. In cutaneous wound healing, the regulatory role of miRNAs and the underlying mechanisms are emerging^20,21^. The importance of miRNA regulation of wound healing has been supported by the observation that loss of the enzyme Dicer, which is a critical regulator of miRNA maturation, leads to delayed wound healing^22^. Individual function of miRNAs has been found in different phases of wound healing. miR-132 promotes skin wound healing by enhancing inflammatory to proliferation stage transition^23,24^. miR-31 promotes skin wound healing by enhancing keratinocyte proliferation and migration^25^. miR-200c which is highly expressed in intact epidermis, has been found to reduce its expression in normal skin wound healing process but remained highly expressed in aged skin wounds, suggesting an inhibitory role of miR-200c in wound healing^26^, in contrast to miR-132 and miR-31.

Keratinocytes in the vicinity of wound migrate in response to extracellular signals upon wounding to close wound bed through the process of re-epithelialization. The elucidation of the signaling pathways that control keratinocyte function is crucial to our understanding of the molecular mechanisms that govern the process of re-epithelialization. Transforming grow factor-β (TGF-β) superfamily cytokines have been found to be potent regulators for wound healing and their regulatory effects have been found to be mediated by Rho GTPases^27,28^. TGF-β induces keratinocyte migration by activating RAC1-MAPK signaling^29^. However, the regulatory mechanism of TGF-β signal on cutaneous wound healing remains elusive.

This study aims to investigate the regulatory function and the mechanism of action of miR-200b/c-3p in wound healing by utilizing a mouse back skin excisional wound model. We show that miR-200b/c-3p dampened keratinocyte migration and re-epithelialization of skin wounds by suppressing RAC1 signaling pathway. Extracellular TGF-β signal downregulated miR-200b/c-3p in keratinocytes and thus abolished the inhibitory effect of miR-200b/c-3p on wound healing. Thus, we propose that targeting TGF-β-miR-200b/c-3p-RAC1-mediated signaling may be beneficial for wound healing.

## Materials and methods

### Bioinformatics analysis

Gene ontology analysis was performed according to the Database for Annotation, Visualization and Integrated Discovery (DAVID) v6.8^30,31^. miR-200b/c-3p target sites were predicted by algorithms TargetScan v7.2^32^ and DIANA microT v5.0^33^. RNA hybridization was simulated with RNAhybrid^34^.

### Mice cutaneous wound healing model

Animal experiments were carried out in accordance with the ARRIVE (animal research: reporting in vivo experiments) guidelines. The protocol was approved by the Southern Medical University Animal Care and Use Committee (Protocol No. L2019133). Six to eight-week-old female C57BL/6 mice (purchased from Laboratory Animal Centre of Southern Medical University) were randomized into different groups, caged individually for one week, handled daily and then wounded. At least 6 mice as biological replicates were used for each experimental group. The mice were anesthetized by intraperitoneal injection of 1% sodium pentobarbital. The dorsal hairs were shaved and depilated with a depilatory cream. A full-thickness excisional wound was created by an 8-mm punch on depilated dorsal skin beside the midline. 20 μM miR-200b/c-3p mimics, small interfering RNA (siRNA) targeting *Rac1* (siRAC1) or negative control (NC) mimics (Ribobio, Guangzhou, China) packed in transfection reagent Lipofectamine 2000 (Invitrogen, Carlsbad, CA, USA), or 10 μg TGF-β receptor inhibitor SB431542 (Selleck Chemicals, Houston, TX, USA), or PBS was injected intradermally into the wound edges immediately after wounding and repeated once at 2 days post-wounding (dpw). Skin tissues at the wound site and wound edge were collected at 4 dpw. TGF-β receptor inhibitor SB431542 (Selleck Chemicals, Houston, TX, USA) was dissolved in 200 μl PBS containing 2% DMSO and 30% PEG 300, and intradermally injected into the wound edges at 10, 20, 50, and 100 μg per mouse daily after wounding. The mice were photographed at 0, 2, 4, 6, 8, and 10 dpw. Skin wound tissues were collected at 4 and 8 dpw for histological analysis. Phenotyping in wounded mice was performed blinded.

### Immunohistochemistry and histology

Tissues from C57BL/6 mouse wound healing model were fixed in 4% paraformaldehyde (PFA) at 4 °C, embedded in paraffin and sectioned (5 μm). Sections were deparaffinated, rehydrated, and performed to antigen retrieval in 10 mM sodium citrate buffer. After washing by PBS three times, sections were blocked with 5% bovine serum albumin (BSA), and incubated with primary antibody against Keratin-14 (K14) (1:200; Cat. No. ab7800, Abcam, Cambridge, MA, USA) at 4 °C overnight. Removing primary antibody next day, sections were washed by PBS three times and performed by a SABC-Cy3 immunohistochemistry staining kit (Cat. No. SA1072, Boster, Wuhan, China). Then sections were stained by 4′,6-diamidino-2-Phenylindole (DAPI) (1:1000, Cat. No. D9542, Sigma-Aldrich, St. Louis, MO, USA), mounted and photographed under a microscope (DM4000B/DFC500, Leica, Wetzlar, Germany). To evaluate the qualities of wound healing comparing different groups, re-epithelialization was observed using hematoxylin and eosin (H&E) staining according to standard procedures. The length of epithelial tongues of wound skin sections was measured between the wound edge and the leading edge of migrating epithelial tongue with ImageJ 1.52a software (National Institutes of Health, Bethesda, MD, USA).

### Cell culture

Human immortalized keratinocyte cell line HaCaT was purchased from OBiO Technology (Shanghai, China) and authenticated by immunostaining for epidermal marker Keratin-14. Cells were cultured in Dulbecco modified essential medium (DMEM) containing 10% fetal bovine serum (FBS) and 1% penicillin/streptomycin. Human embryonic kidney cell line 293T (HEK293T) were purchased from the Cell Bank of Chinese Academy of Sciences (Shanghai, China) and cultured in DMEM supplemented with 10% FBS. All cells were incubated at 37 °C in a 5% CO_2_ atmosphere. All materials used for cell culture were purchased from Gibco (Carlsbad, CA, USA). The cell lines were tested for mycoplasma contamination routinely after thawing and recovering from liquid nitrogen storage.

### miRNA mimics and siRNA transfection

HaCaT cells of passage 3–6 grown to 30–50% confluence were transfected with 100 nM miR-200b/c-3p mimics, NC or siRAC1 (Ribobio) using Lipofectamine 2000 (Invitrogen) according to the manufacturer’s description for 48 h. RNA sequences are listed in Supplementary Table 1.

### TGF-β1 stimulation

2–4 × 10^4^ HaCaT cells per well were seeded in a 24-well plate or on glass coverslips, serum-starved overnight and stimulated by TGF-β1 (20 ng/ml working concentration; Cat. No. 100-21, Peprotech, Rocky Hill, CT, USA) for 24–48 h. To observe the effect of TGF-β1 in HaCaT cells during migration, transfected cells wounds were made by a 200 μl pipette tip and stimulated by TGF-β1 during healing. Protein markers were observed by immunofluorescence staining under a microscope (Dmi8, Leica).

### RNA extraction and reverse transcription-quantitative polymerase chain reaction (RT-qPCR)

Total RNA was extracted from cells or tissues using RNAiso Plus Reagent (Takara, Dalian, China). Skin tissues from C57BL/6 Mice was homogenized in liquid nitrogen before extracting RNA preparation. 1 μg total RNA was reverse transcribed using the PrimeScript RT reagent kit with gDNA Eraser (Takara). miRNA RT-qPCR was performed as described previously^35^. Quantitative PCR was performed using the TB Green Premix Ex Taq (Takara) and quantified using the 2^−ΔΔC(t)^ method. The expression of miR-200b/c-3p (fold change) between samples was normalized on the basis of U6 snRNA, and mRNAs expression (fold change) were normalized to *Gapdh* mRNA. Primers used are listed in Supplementary Table 1.

### Rhodamine-phalloidin staining

HaCaT cells were seeded in 24-well plates on one glass coverslip in each well at a density of 4 × 10^4^ cells per well. The cells were transfected with 100 nM miR-200b/c-3p mimics or negative control mimic or siRAC1. The cells were then serum-starved overnight followed by treatment with or without TGF-β1 (20 ng/ml, Peprotech). 48 h after treatments, the coverslips were removed and the cells on the coverslips were fixed in 4% PFA, permeabilized with 0.1% Triton X-100 for 5 min, blocked with 1% BSA for 30 min at room temperature, and stained with phalloidin (Alexa Fluor 546-phalloidin, 1:40, Cat. No. A22283, Invitrogen). After washing with PBS, the cells were counterstained with DAPI (1:500, Sigma-Aldrich) for 1 min, washed again with PBS, mounted, and examined by fluorescence microscopy (DMi8, Leica).

### Immunofluorescence staining

HaCaT cells cultured on glass coverslips were fixed in 4% PFA for 10 min, permeabilized with 0.1% Triton X-100 for 5 min, blocked with 5% bovine serum albumin for 30 min at room temperature. The cells were incubated with specific primary antibodies against RAC1 (1:1000, Cat. No. 66122-1-Ig, Proteintech, Chicago, IL, USA), E-cadherin (1:100, Cat. No. 20874-1-AP, Proteintech) overnight at 4 °C. The cells were washed by PBS and incubated with Dylight 594 (1:100, Cat. No. 35560, Thermo Scientific, Waltham, MA, USA) or CoraLite 488-conjugated (1:100, Cat. No. CL488-66122, Proteintech) secondary antibodies for 1 h at room temperature after removing primary antibodies. Cells were stained in DAPI (1:1000, Sigma-Aldrich), mounted and observed using a Leica fluorescence microscope. Immunofluorescence artificial intensity was determined as average IOD (Integrated Optical density) by ImageJ software.

### Scratch wound healing assay

HaCaT cells were seeded in a 24-well plate or on 24-well size glass coverslips and transfected 100 nM miR-200b/c-3p mimics or siRAC1 or NC; 200 nM miR-200b/c-3p inhibitors or NC inhibitor at a 30–50% confluence. 48 h after transfection, a scratch wound was made by a 200 μl pipette tip on confluent cells. The wounds were photographed at 12 h, 24 h after scratching and stained for other assays.

### Transwell migration assay

HaCaT cells at passage 3 were transfected with miR-200b/c-3p mimics or siRAC1 or NC, resuspended in serum-free medium, and adjusted to a density of 1 × 10^6^ cells per milliliter. One hundred microliter of infected HaCaT cells were placed in the upper chamber of transwell plates (Corning, Corning, NY, USA). DMEM with 10% FBS was added to the lower chamber. After incubation for 24 h at 37 °C, migrated cells were collected from the lower chambers, stained with crystal violet (Beyotime, Shanghai, China), photographed (×20), and counted. Three independent experiments were performed.

### Cell scattering assay

2 × 10^4^ HaCaT cells were seeded on 24-well glass coverslips, allowed to grow for 24 h and transfected with 100 nM RNA duplexes at a 30% confluence. 24 h after transfection, cells were serum-starved overnight and stimulated by TGF-β1 (20 ng/ml, Peprotech) for 24 h. Cells were fixed in 4% PFA, stained with Alexa Fluor 546-phalloidin (1:40, Invitrogen) and examined by fluorescence microscopy (DMi8, Leica). Lamellipodia surface area of individual cells was measured by ImageJ software (NIH) and shown as percentage of total cell surface.

### Western blot analysis

HaCaT cells and tissues were lysed by RIPA (Beyotime) to extract proteins after treatments. Centrifuging cells lysis at 12,000 rpm for 10 min, supernatant was removed to clean Eppendorf tubes, added by 5 × SDS Loading Buffer at 1:4 ratio and boiled at 95 °C. The protein samples were separated on 10% sodium dodecyl sulfate polyacrylamide gels (SDS-PAGE) alongside with prestained standard molecular weight markers (Invitrogen) and transferred to a PVDF membrane (Millipore, Billerica, MA, USA). Blots were blocked in QuickBlock blocking buffer for western blot (Beyotime) for 1 h and incubated with primary antibody against RAC1 (1:1000, Cat. No. 610651, BD Biosciences, Franklin lakes, NJ, USA) at 4 °C overnight. The PVDF membranes were shook in goat-anti-mouse or goat-anti-rabbit secondary antibodies incubation for 1 h after washing by 1 × TBST at room temperature. Antibody binding was detected using an enhanced chemiluminescence kit (Cat. No. WBKLS0500, Millipore). The bands were quantified using Gel-Pro Analyzer Application (Media Cybernetics, Rockville, MD, USA) by measuring the band intensity.

### Dual luciferase reporter assay

Full length mRNA 3′UTR fragments were amplified from cDNA by PCR. Primers used for cloning can be found in Supplementary Table 1. PCR products were then inserted into psiCHECK2 vector (Promega, Madison, WI, USA) between *Xho* I and *Not* I restriction enzyme sites downstream of the *Renilla* luciferase reporter gene. *RAC1* 3′UTR mutant construct was generated using MutanBEST kit (Cat. No. R401, Takara) and the mutation was confirmed by sequencing. miR-200b/c-3p expression vectors were cloned by amplifying the miR-200b/c-3p hairpin from the genomic DNA and inserted into pcDNA6.2/EmGFP at the *Eco*R I/*Xho* I cloning sites. The pcDNA6.2/EmGFP-miR-neg plasmid (Invitrogen) was used as negative control. For the luciferase reporter assay, 50 ng of psiCHECK2-Target plasmid DNA, and 150 ng of pcDNA6.2-miRNA DNA were co-transfected into HEK293T cells in a 48-well plate using Lipofectamine 2000 (Invitrogen). Cell lysates were collected 48 h after transfection and *Renilla* and firefly luciferase activities were measured in relative light units (RLU) using the Dual-Glo Luciferase Assay System (Cat. No. E2920, Promega) and Cytation 5 multi-mode plate reader (Biotek, Winooski, VT). *Renilla* luciferase activity was normalized to the internal control firefly luciferase activity. Firefly luciferase is used as internal control luciferase for normalization because psiCHECK2 provides a moderate-level and constitutive expression of firefly luciferase among experimental groups. Relative luciferase activities are ratios of *Renilla*/Firefly RLU normalized to negative control for each reporter construct.

### Statistical analysis

Data were presented as mean ± standard deviation (SD) from at least three independent experiments. Individual data points displayed in the figures are mean values of all technical replicates for each independent experiment or biological replicates for animal experiments. Statistical analyses were performed by SPSS 22.0 (IBM Corp, Armonk, NY) or GraphPad Prism 8 (GraphPad Software, Inc, La Jolla, CA). Statistical differences among groups were analyzed using one-way analysis of variance (ANOVA) with Bonferroni *post hoc* multiple comparison analysis or two-tailed unpaired Student’s *t*-test. *P* value < 0.05 was considered as statistically significant.

## Results

### RAC1 is a direct target of miR-200b/c-3p

**Fig. 1:**
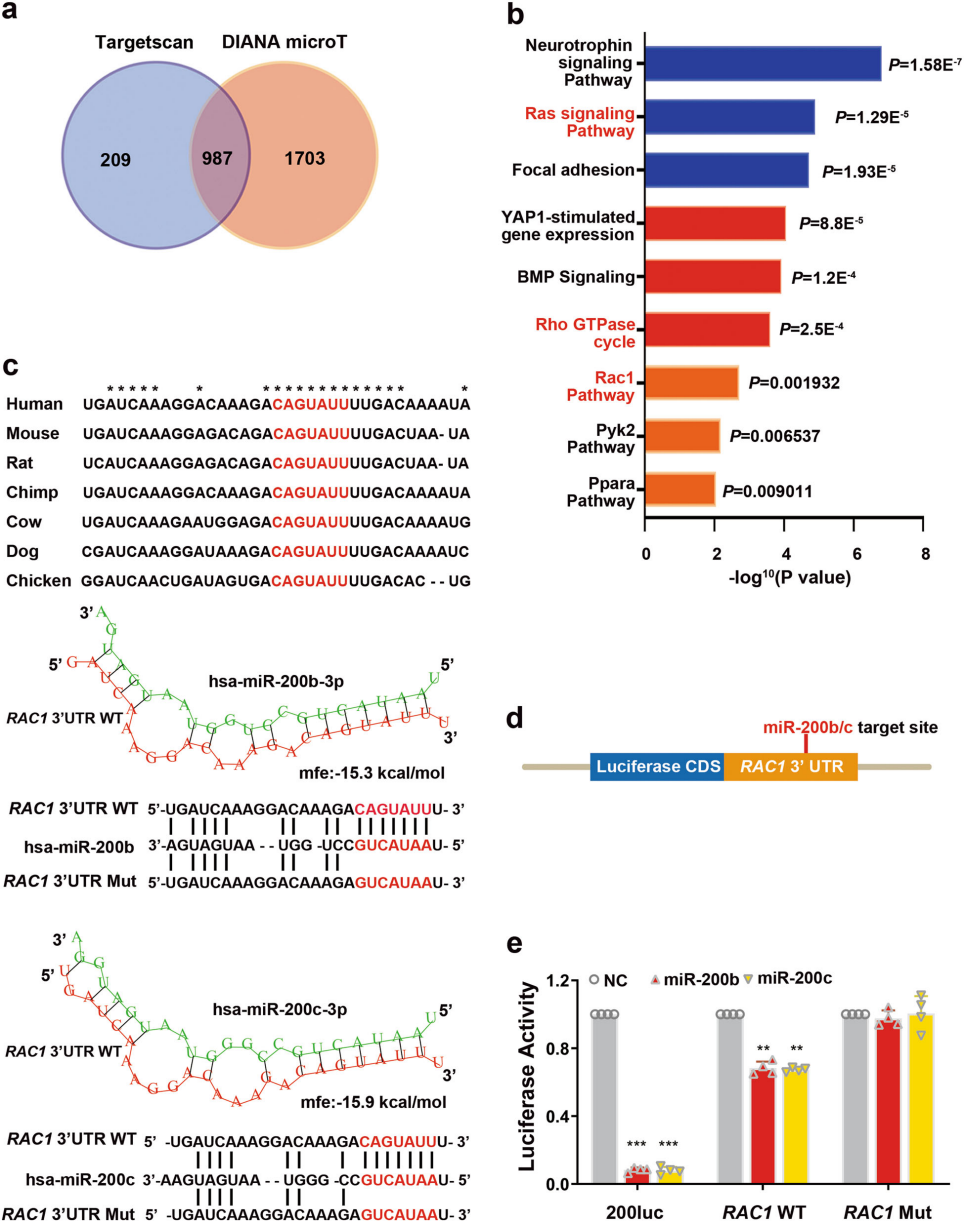
miR-200b/c-3p directly target to *RAC1* 3′UTR. **a** Venn diagram depicting predicted miR-200b/c-3p target gene numbers from two algorithms. **b** Top pathways enriched for putative miR-200b/c-3p target genes with potential binding sites in 3′UTRs. DAVID was used for gene ontology enrichment analysis. **c** Putative miR-200b/c-3p binding site in *RAC1* 3′UTR is conserved among vertebrates. miRNA-mRNA hybridization structures and folding energy was predicted by RNAhybrid. **d** Schematic representation of luciferase reporter construct with full length *RAC1* 3′UTR. **e** Luciferase reporter assay confirmed miR-200b/c-3p repressed RAC1 by direct binding to a conserved binding site in 3′UTR. *Renilla* luciferase activity was normalized to firefly luciferase activity. Relative luciferase activities were ratios of *Renilla*/Firefly luciferase normalized to negative control for each reporter construct. A reporter with reverse complement sequence of miR-200b/c-3p (200luc) was served as positive control (*n* = 4). Data presented as mean ± SD. **P < 0.01, ***P < 0.001 versus negative control, one-way ANOVA.

miRNAs exert biological functions by repressing target genes post-transcriptionally. They bind to 3′ untranslated region (3′UTR) of messenger RNA (mRNA) by base-pairing with the “seed sequence” which mostly situated at nucleotide 2–8 from the 5′ end of miRNAs^14^. To identify miR-200b/c-3p target genes, we first performed in silico prediction. Bioinformatics analysis using the publicly available algorithms TargetScan^32^ and DIANA microT^33^ predicted 1196 and 2690 genes containing potential miR-200b/c-3p target sites, respectively (Supplementary Table 2). 987 candidate targets of miR-200b/c-3p were shared by these two algorithms (Fig. 1a). Gene ontology analysis by the Database for Annotation, Visualization and Integrated Discovery (DAVID)^30,31^ found that RAC1 signaling pathway was among the top enriched terms (Fig. 1b). RNA hybridization prediction by RNAhybrid^34^ identified a conserved binding site of miR-200b/c-3p in the *RAC1* mRNA 3′UTR of the human and mouse transcripts (Fig. 1c).

To confirm a direct interaction between miR-200b/c-3p and *RAC1* mRNA 3′UTR, we constructed luciferase reporter vectors. Full length *RAC1* 3′UTR was inserted downstream of the *Renilla* luciferase coding sequence (Fig. 1d). This reporter was co-transfected with miR-200b/c-3p expressing vectors into HEK293T cells and luciferase activity was measured. As a positive control, the activity of a reporter construct containing the reverse complement sequence of miR-200b/c-3p was nearly abolished by miR-200b/c-3p. miR-200b/c-3p significantly repressed luciferase activity of the *RAC1* 3′UTR reporter, confirming that miR-200b/c-3p directly inhibited RAC1 (Fig. 1e). In contrast, such inhibitory effect was reversed when a mutation was introduced to the miR-200b/c-3p binding site in the 3′UTR of *RAC1* (Fig. 1c, e).

### miR-200b/c-3p repress RAC1 in keratinocytes

To study the potential regulatory effect of miR-200b/c-3p on RAC1 in cutaneous wound healing, we employed a mouse model with full-thickness back skin excision. In this model, skin wounds were healed through a reproducible time course^2^. Inflammatory phase started upon wounding as evidenced by the filtration of inflammatory cells in the wound edge at 1 dpw. Subsequent cellular phenotype represented the proliferation phase that keratinocytes-formed epithelial migration tongue was obvious at 2–7 dpw and the wounds were fully re-epithelialized by 8 dpw. Remodeling of the granulation tissues lasted until 14 dpw.

**Fig. 2:**
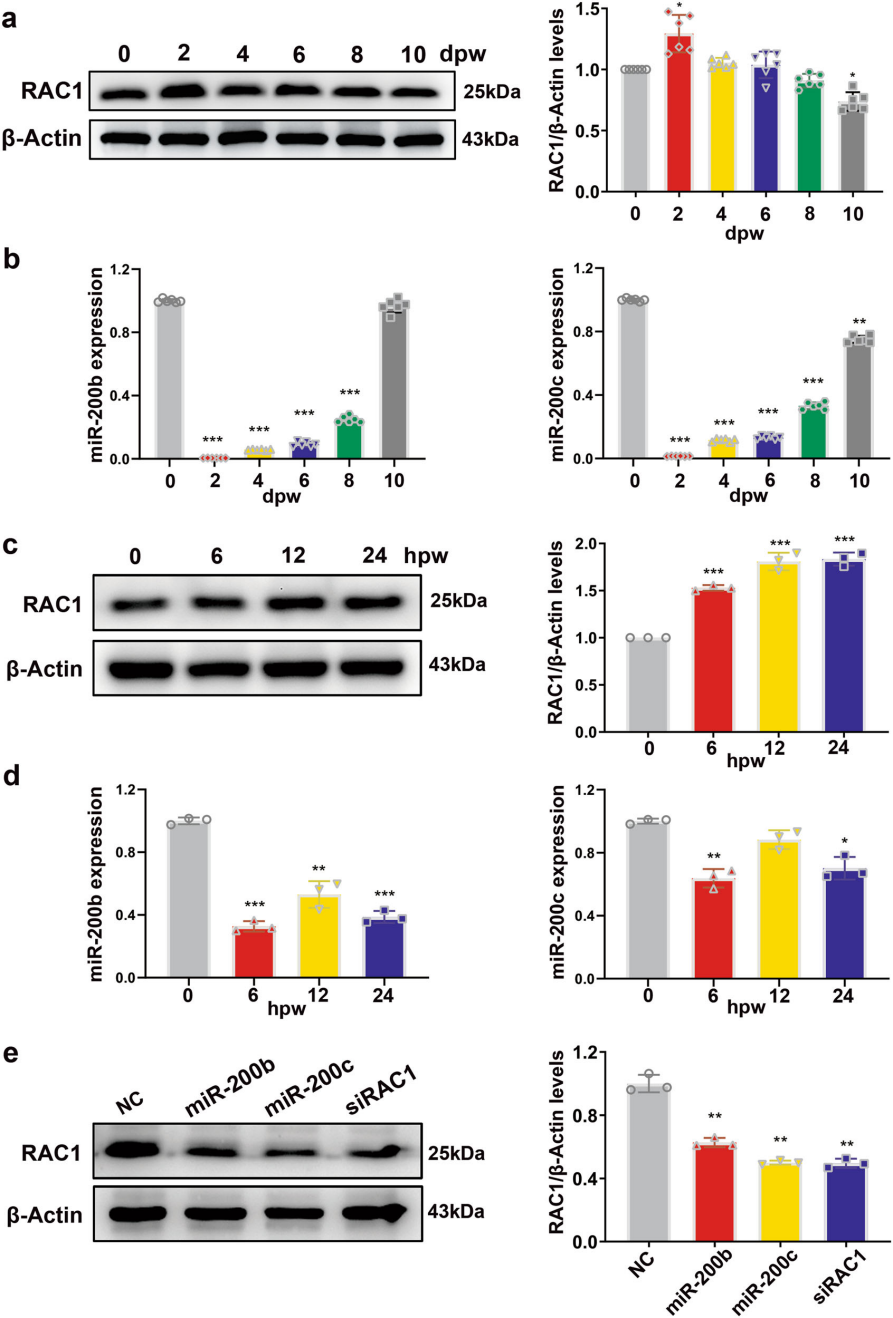
miR-200b/c-3p repress RAC1 signaling in keratinocytes. **a** Mice dorsal skin was wounded and wound edge samples were collected at the indicated time points post wounding for western blotting. Representative western blot and quantitative data showed RAC1 protein level was elevated in mice skin wounds (*n* = 6). **b** RT-qPCR analysis of wound edge samples collected as in (**a**) showed miR-200b/c-3p expression was decreased in mice skin wounds (*n* = 6). **c** Confluent HaCaT cells were scratch wounded and samples were collected at the indicated time points post wounding for western blotting. Representative western blot and quantitative data showed RAC1 protein level was elevated in HaCaT cell scratch wounds (*n* = 3). **d** RT-qPCR analysis of samples collected as in (**c**) showed miR-200b/c-3p expression was decreased in HaCaT cell scratch wounds (*n* = 3). **e** Western blot analysis of RAC1 in HaCaT cells transfected with RNA duplexes as indicated. Representative western blot and quantitative data showed RAC1 protein levels were significantly reduced by miR-200b/c-3p duplexes or siRNA (*n* = 3). dpw, days post-wounding; hpw, hours post-wounding. Data presented as mean ± SD. *P < 0.05, **P < 0.01, ***P < 0.001 versus control, one-way ANOVA.

Western blotting of RAC1 protein from the wound edge tissues showed a transient elevation of RAC1 protein level during the proliferation phase (Fig. 2a). In contrast, RT-qPCR revealed that miR-200b/c-3p transcript levels were significantly decreased upon wounding (Fig. 2b). Since miR-200b/c-3p were found abundantly expressed in epidermis^36^, we dissected the expression pattern of miR-200b/c-3p and RAC1 protein in epidermal keratinocytes sheet after scratch wounding. Confluent human immortalized keratinocyte HaCaT cells were scratch wounded and RAC1 protein level was determined by western blot at continuous time points after scratching. Consistent with the observation in the murine wound healing model, RAC1 protein level increased when miR-200b/c-3p levels were decreased in keratinocyte upon scratch wounding (Fig. 2c, d). The reciprocal expression pattern between miR-200b/c-3p and RAC1 protein suggests that RAC1 is targeted by miR-200b/c-3p in keratinocytes. To verify the negative regulation of RAC1 by miR-200b/c-3p in keratinocytes, we transfected HaCaT cells with miR-200b/c-3p mimics. Forced expression of miR-200b/c-3p dramatically downregulated RAC1 protein. As a positive control, an siRNA targeting *RAC1* mRNA (siRAC1) significantly inhibited RAC1 protein level (Fig. 2e). Collectively, these data confirm that RAC1 is an actual target of miR-200b/c-3p in keratinocytes.

### RAC1 repression by miR-200b/c-3p dampens keratinocyte migration and re-epithelialization of cutaneous wound

**Fig. 3:**
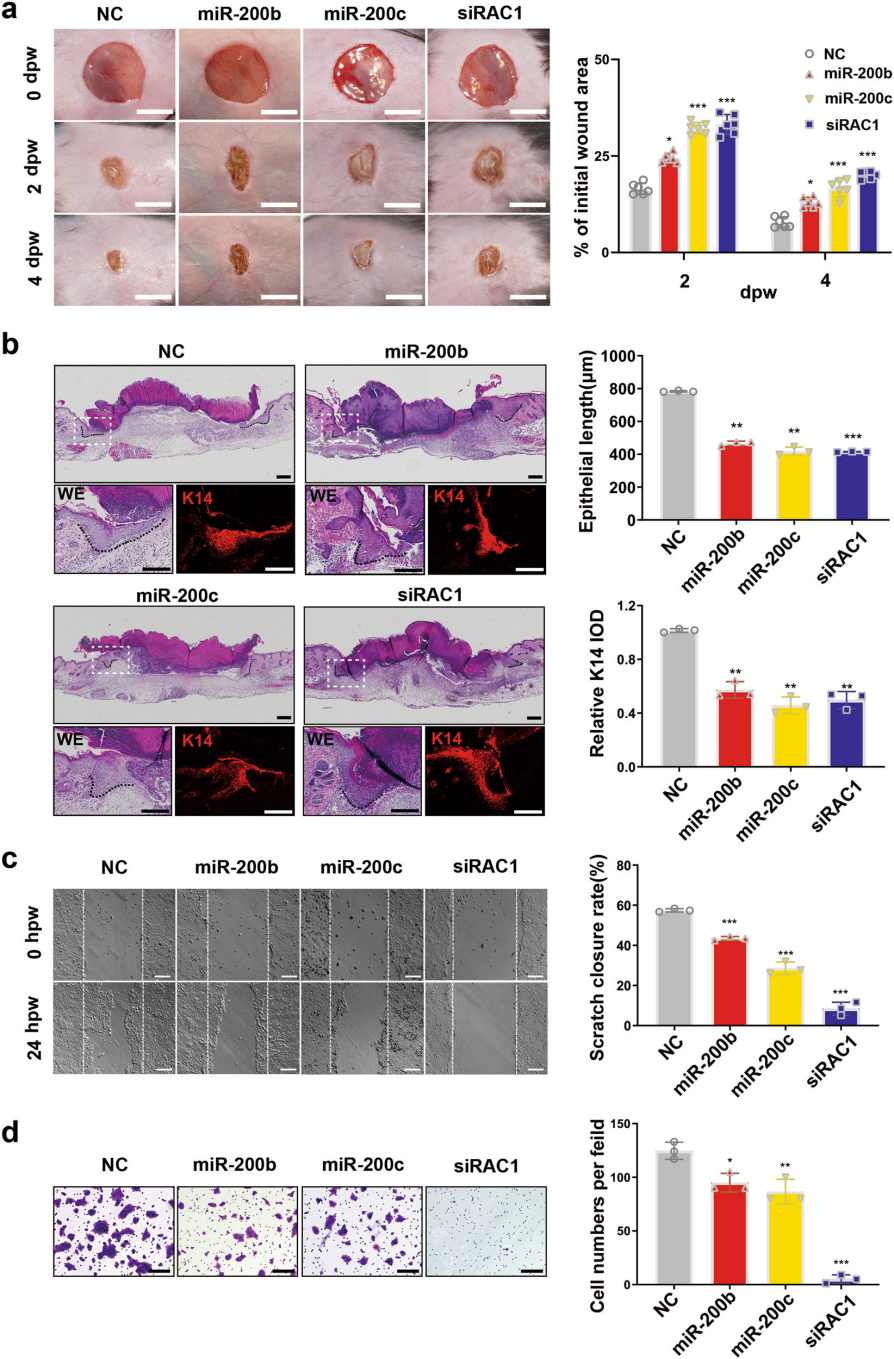
miR-200b/c-3p inhibit cutaneous wound re-epithelialization and keratinocyte migration through RAC1. **a** Mice dorsal skin was wounded and injected intradermally with RNA duplexes as indicated in the wound edge. Live images were captured at the indicated time points post wounding. Representative live images and quantitative data of wound healing rate show delayed wound healing in miR-200b/c-3p mimics or siRAC1-injected wounds (*n* = 6). **b** Wound edge samples as in (**a**) were collected at 4 dpw for H&E staining (WE) or immunofluorescence staining of keratin 14 (K14). Dashed lines indicate the epithelial tongues. Representative images and quantitative data of epithelial tongues showed repression of re-epithelialization in miR-200b/c-3p or siRAC1-injected wounds (*n* = 3). **c** HaCaT cells transfected with RNA duplexes were used for measuring their migration capacity with scratch wound assay. Representative images and quantitative data showed repressed cell migration in miR-200b/c-transfected cells when siRAC1 almost abolished cell migration (*n* = 3). **d** Transwell assay showed repressed HaCaT cell migration as in (**c**) (*n* = 3). dpw, days post-wounding; hpw, hours post-wounding; IOD, integrated optical intensity. Data presented as mean ± SD. *P < 0.05, **P < 0.01, ***P < 0.001 versus negative control, one-way ANOVA. Scale bars: 5 mm in (**a**), 200 μm in (**b**–**d**).

To investigate the regulatory function of miR-200b/c-3p in cutaneous wound healing, miR-200b/c-3p mimics or scrambled negative control were injected intradermally into the wound edge at 0 and 2 dpw. Live photography capturing the wound healing process showed that miR-200b/c-3p-injected wounds healed more slowly than negative control. Similarly, siRAC1 injection attenuated wound healing as seen with miR-200b/c-3p overexpression (Fig. 3a). Hematoxylin and eosin (H&E) staining of skin wounds at 4 dpw demonstrated shorter epithelial migration tongue in miR-200b/c-3p-injected wound edge (Fig. 3b). Furthermore, immunohistochemistry analysis of keratin-14 (K14), which is specifically expressed in the basal layer keratinocytes in epidermis, revealed that the migration of basal keratinocytes and the re-epithelialization process was inhibited in miR-200b/c-3p-injected wound edge (Fig. 3b). Silencing of RAC1 expression with siRNA injection also ameliorated basal keratinocyte migration (Fig. 3b). This delay in wound re-epithelialization is consistent with the finding in transgenic mice harboring a dominant-negative mutant of RAC1^11^.

To explore how miR-200b/c-3p exert an inhibitory effect on re-epithelialization, we studied the cellular function of miR-200b/c-3p in keratinocytes migration. Overexpression of miR-200b/c-3p reduced HaCaT cell migration in scratch wound assay, which was recapitulated by RAC1 deficient keratinocytes (Fig. 3c). Moreover, in transwell migration assay, miR-200b/c-3p overexpression dampened the migration of keratinocytes and the motility of RAC1 deficient cells was almost abolished (Fig. 3d). Together, our results reveal that miR-200b/c-3p inhibition of RAC1 dampened keratinocyte migration and contributed to the delay of re-epithelialization.

### TGF-β1 inhibits miR-200b/c-3p expression in keratinocytes

**Fig. 4:**
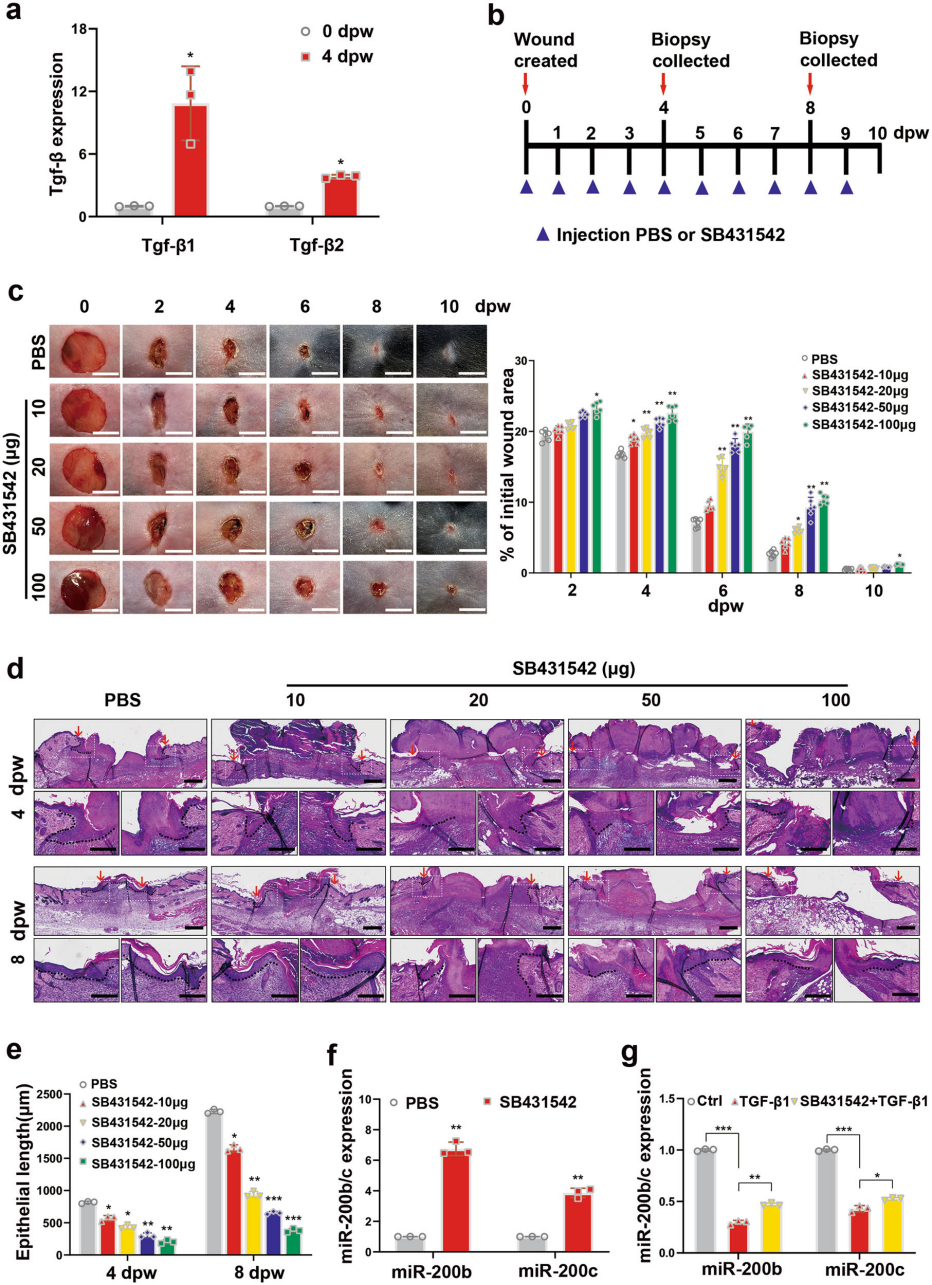
TGF-β1 represses miR-200b/c-3p expression in keratinocytes. **a** RT-qPCR analysis of mice dorsal skin wounds at the indicated time points showed elevated TGF-β1 expression (*n* = 3). **b** Mice dorsal skin was intradermally injected at the wound edge with TGF-β type I receptor inhibitor SB431542 (10, 20, 50, and 100 μg per mouse) or PBS as negative control. Wound edge samples were collected at 4 and 8 dpw. **c** Representative live images of wounds and quantitative data of wound healing rate at 0, 2, 4, 6, 8, and 10 dpw showed delayed wound healing in SB431542-injected wounds (*n* = 6). **d** Wound edge samples of 4 and 8 dpw treated as in (**c**) were used for H&E staining. Representative images of epithelial tongues (indicated by dashed lines) showed repression of re-epithelialization in SB431542-injected wounds. Arrows indicate wound edges. **e** Quantitative data of the length of epithelial tongues from samples as in (**d**) (*n* = 3). **f** RT-qPCR analysis of samples of 4 dpw treated with PBS or SB431542 showed elevated miR-200b/c-3p expression when TGF-β1 signaling was inhibited (*n* = 3). **g** RT-qPCR analysis of HaCaT cells treated as indicated with TGF-β1 (10 ng/ml) and SB431542 (10 nM) for 24 h (*n* = 3). dpw, days post-wounding. Data presented as mean ± SD. *P < 0.05, **P < 0.01, ***P < 0.001 versus control, Student’s *t*-test in (**a**, **f**, **g**), one-way ANOVA in (**c**, **e**). Scale bars**:** 5 mm in (**c**), 200 μm in (**d**).

Our data have indicated that reduced miR-200b/c-3p expression in skin wound would improve the healing process. To gain insight into the mechanism that mediates the downregulation of miR-200b/c-3p in skin wound, we focused on TGF-βs that are upregulated in skin wounds and may promote wound healing^37^. RT-qPCR showed that the abundance of TGF-β1 transcript elevated more significantly than TGF-β2 upon skin wounding in mice (Fig. 4a). When the pharmacological inhibitor of TGF-β receptor SB431542 was injected intradermally around the wound edge, the healing process was delayed when compared to control (Fig. 4b, c). Furthermore, histology analysis showed that inhibition of TGF-β signaling reduced wound closure rate and epithelial tongue length (Fig. 4d, e). These observations confirm a pro-healing effect of TGF-β signaling in this model. We then evaluated the effect of TGF-β1 on miR-200b/c-3p expression. In SB431542-injected mice skin wounds, miR-200b/c-3p expression was higher than control (Fig. 4f). In keratinocytes, while TGF-β1 reduced miR-200b/c-3p expression in HaCaT cells, HaCaT cells treated with SB431542 abolished the repressive effect of TGF-β1 on miR-200b/c-3p (Fig. 4g). Collectively, our results suggest that the increased level of TGF-β1 in the wound epidermis contribute to trigger the downregulation of miR-200b/c-3p in the keratinocytes during skin wound healing.

### miR-200b/c-3p repress TGF-β1-induced lamellipodia and stress fiber formation in keratinocytes

**Fig. 5:**
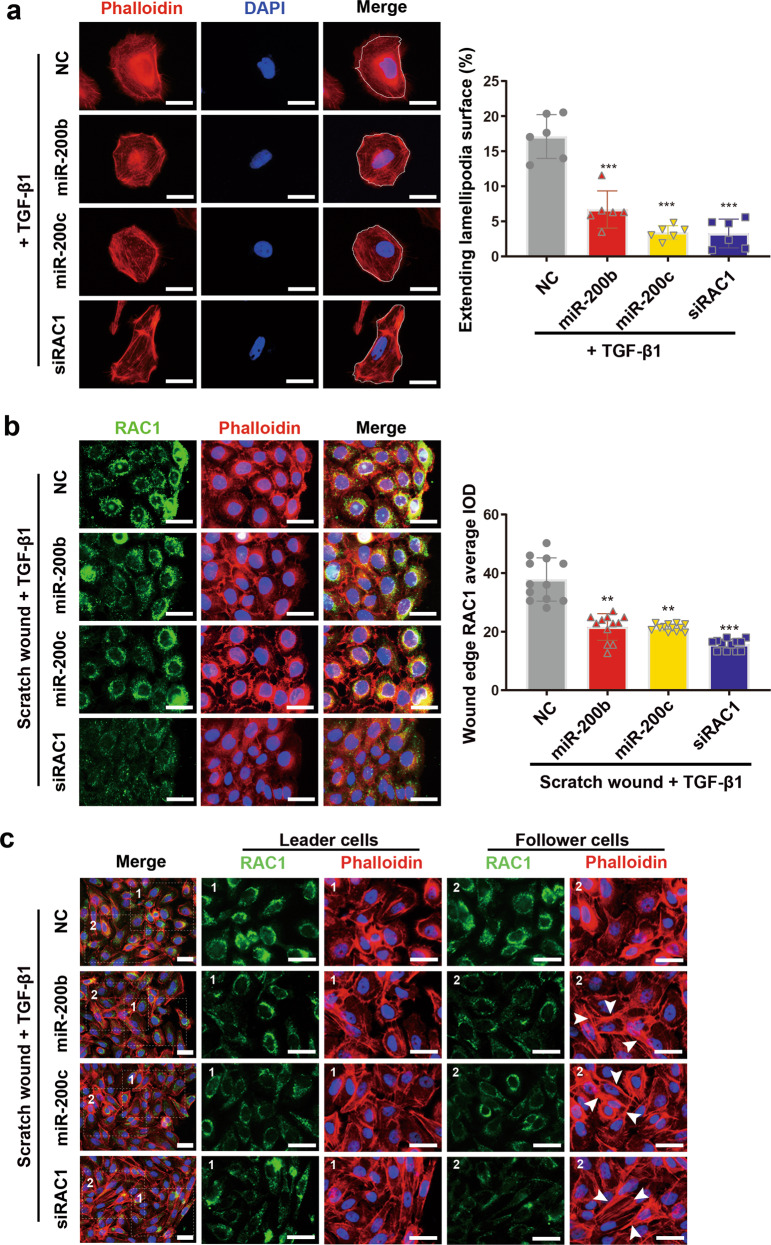
Effects of miR-200b/c-3p on keratinocyte lamellipodia and stress fiber formation. **a** Phalloidin staining of actin cytoskeleton in HaCaT cells transfected with RNA duplexes as indicated at 24 h post TGF-β1 treatment. Representative images and quantitative data of lamellipodia versus cell surface showed attenuated lamellipodia formation in miR-200b/c-3p or siRAC1-transfected cells (lamellipodia versus cell surface, *n* = 6). **b** HaCaT cells were transfected with RNA duplexes as indicated and confluent cells were scratch wounded and treated with TGF-β1 for 6 h. RAC1 protein was shown by immunofluorescence staining (green) and actin cytoskeleton was shown by phalloidin staining (red). Control cells in the leading edge form lamellipodia and RAC1 protein level increased and accumulated in the membrane ruffles facing the migrating direction. RAC1 in miR-200b/c-3p-transfected cells remained in perinuclear area with slight elevation in the leading edge. These cells seldom form lamellipodia. siRAC1 significantly reduced RAC1 indicated by immunofluorescence average integrated optical intensity (IOD) and prevented cells from lamellipodia formation (*n* = 12). **c** HaCaT cells treated as in (**b**) were fixed at 24 hpw. Leader cells transfected with miR-200b/c-3p or siRAC1 became elongated. Stress fibers (indicated by arrowheads) were more obvious in miR-200b/c-3p or siRAC1-transfected cells than control in follower cells. Data presented as mean ± SD. **P < 0.01, ***P < 0.001 versus negative control, one-way ANOVA. Scale bars: 25 μm.

Upon wounding, basal keratinocytes at the wound edge undergo epithelial-mesenchymal-transition (EMT)-like switch to commence migration. TGF-β1 is a potent activator of EMT and mediates cell migration, partially through RAC1 signaling pathway^29,38^. To determine whether and how miR-200b/c-3p regulate TGF-β1-stimulated keratinocyte movements, we used TGF-β1 to treat HaCaT cells with or without forced expression of miR-200b/c-3p and studied the motility properties. First we studied undirected single-cell migration by using cell scattering assay to define cellular morphological changes. HaCaT cells were spread sparsely and the morphology of individual cells was observed with phalloidin staining against actin cytoskeleton. TGF-β1-induced lamellipodia extension in the cell periphery in HaCaT cells. Noting that TGF-β1 inhibits miR-200b/c-3p expression, forced expression of miR-200b/c-3p in TGF-β1-treated cells led to less extending lamellipodia surface with the cells becoming rounded. Furthermore, these cells showed a number of stress fibers in the periphery of cytoplasm. In line with the finding that miR-200b/c-3p target RAC1, cells transfected with siRAC1 mimicked the cells with forced expression of miR-200b/c-3p, as manifested by less lamellipodia formation and more stress fiber formation with elongated morphology (Fig. 5a).

We then studied the directed collective cell migration of keratinocytes, using a tissue culture scratch wound assay in which cells at the wound edge undergo collective migration. In scratched HaCaT cells exposed to TGF-β1 for 6 h, RAC1 protein level was upregulated in the front row of cells at the migration front, as demonstrated by immunofluorescence. Furthermore, RAC1 was not only localized to the perinuclear area, but in the ruffles at the cell front towards migrating direction (Fig. 5b). This is consistent with the fact that RAC1 is required for TGF-β1-induced cell migration^29^. These cells forming lamellipodia at the migration front represent the leader cells of collectively migrating wound epidermis. TGF-β1-induced up-regulation of RAC1 in leader cells was reversed by forced expression of miR-200b/c-3p and RAC1 was retained to the perinuclear area. In addition, the leader cells bearing miR-200b/c-3p overexpression or RAC1 silencing failed to form lamellipodia (Fig. 5b). These leader cells became elongated upon extended treatment with TGF-β1 for 24 h. Interestingly, within the follower cells that harbored miR-200b/c-3p overexpression or RAC1 silencing and located several rows back from the migration front, stress fibers became obvious, whereas the followers in control group exhibited very few stress fibers (Fig. 5c). This may be a consequence of unreleased tension within cells. These data suggest that a failure of lamellipodia formation in leader cells and exhibition of stress fibers in follower cells contribute to the stalling of epithelial migration. Taken together, these observations indicate that downregulation of miR-200b/c-3p contributes to TGF-β1-induced keratinocyte migration through RAC1-mediated lamellipodia formation.

### miR-200b/c-3p repress TGF-β1-induced intercellular junctional dissolution in keratinocytes

**Fig. 6:**
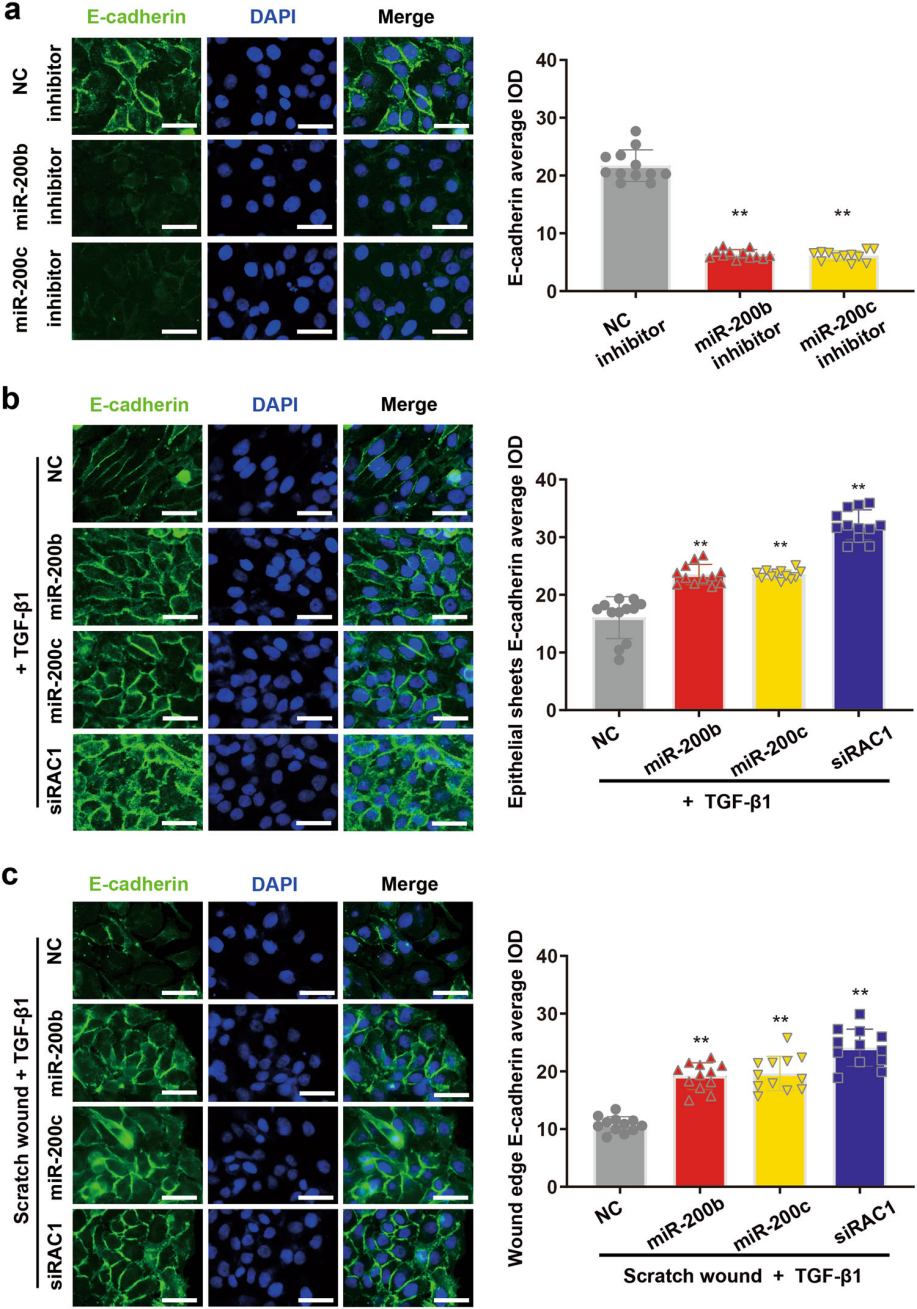
TGF-β1/miR-200b/c-3p/RAC1 axis regulates intercellular adhesion. **a** HaCaT cells were transfected with RNA inhibitors as indicated and allowed to grow to confluent. E-cadherin protein was shown by immunofluorescence staining (green). Inhibition of miR-200b/c-3p led to dissociation of adherens junction as shown by loss of E-cadherin indicated by immunofluorescence average integrated optical intensity (IOD) (*n* = 12). **b** HaCaT cells transfected with RNA duplexes as indicated were fixed at 24 h post TGF-β1 treatment. Overexpression of miR-200b/c-3p or repression of RAC1 maintained the expression of E-cadherin (*n* = 12). **c** HaCaT cells were transfected with RNA duplexes as indicated and confluent cells were scratch wounded and treated with TGF-β1 for 24 h. Wound edge cells loss E-cadherin. Overexpression of miR-200b/c-3p or repression of RAC1 maintained the expression of E-cadherin (*n* = 12). Data presented as mean ± SD. **P < 0.01 versus negative control, one-way ANOVA. Scale bars: 25 μm.

To explore whether stress fiber formation is a consequence of unreleased tension between neighboring cells, we investigated the intercellular junctions, whose loosening is needed for epithelial migration^5^. In intact epithelial sheet formed by keratinocytes, intercellular adhesion was tightly formed as shown by immunofluorescence against adherens junction marker E-cadherin (Fig. 6a). Due to the high expression of miR-200b/c-3p in epidermis, we transfected HaCaT cells with miR-200b/c-3p inhibitors. Conversely, adherens junctions in these cells were dissolved as shown by compromised E-cadherin expression, emphasizing a critical role of miR-200b/c-3p in maintaining epithelial characteristics (Fig. 6a). TGF-β1 is a potent inducer of EMT that stimulates intercellular junctional dissolution between keratinocytes via RAC1 signaling^29^. 24-h exposure of TGF-β1 reduced E-cadherin expression in confluent HaCaT keratinocytes. Depletion of RAC1 with siRNA abolished TGF-β1-induced downregulation of E-cadherin. We further tested the regulation of intercellular adhesion by miR-200b/c-3p in TGF-β1-treated cells. In line with miR-200b/c-3p targeting to RAC1, miR-200b/c-3p-transfected cells retained the expression of E-cadherin in the presence of TGF-β1 (Fig. 6b), indicating that TGF-β1-induced intercellular adhesions loosening is mediated by miR-200b/c-3p regulation of RAC1. Moreover, when the epithelial cell sheet was wounded, significant downregulation of E-cadherin was observed in control cells at the migration front. This loosening of epidermal junctions enables collective forward migration of leader cells and follower cells to repair the wounds. In contrast, HaCaT cells with miR-200b/c-3p or siRAC1 transfection maintained adherens junctions (Fig. 6c). These results support the notion that RAC1 repression by miR-200b/c-3p inhibits TGF-β1-induced intercellular adhesion dissolution.

## Discussion

**Fig. 7:**
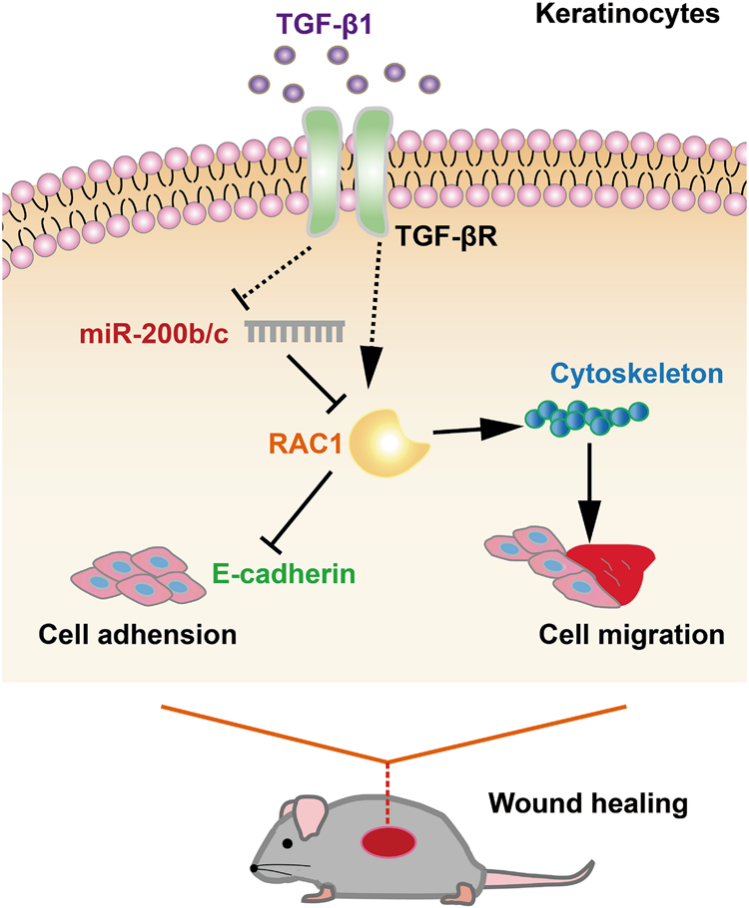
Schematic model for TGF-β1/miR-200b/c-3p/RAC1 regulation of cutaneous wound healing. Upon cutaneous wounding, TGF-β1 activates RAC1 by repressing miR-200b/c-3p expression, thus stimulating keratinocyte intercellular contact dissolution and migration to promote wound healing.

In this study, we characterize the biological function of miR-200b/c-3p and the underlying regulatory mechanism during skin wound healing. The expression of miR-200b/c-3p is abundant in the intact skin but significantly downregulates in the proliferative phase upon wounding. Mechanistically, we propose a pathway in which miR-200b/c-3p suppress RAC1 to inhibit the formation of lamellipodia, intercellular adherens junction dissolution, stress fiber disassembly and hence the migration of epidermal keratinocytes. TGF-β1 promotes the re-epithelialization process by downregulating miR-200b/c-3p expression and antagonizes their anti-migratory effect to heal cutaneous wound (Fig. 7).

In the epidermis, distinct cell lineages, including keratinocytes, epidermal stem cells, and hair follicle stem cells coordinate the healing process of epidermal wounds^39,40^. After injury, the keratinocytes that re-epithelialize the skin wound exhibit considerable plasticity and undergo a partial epithelial-to-mesenchymal transition (EMT) to heal the wound^41^. These epithelial cells migrate towards the site of damage in a coordinated manner to contribute to repair^41–43^. The basal cells at the immediate wound margin take the leadership role, guiding collective migration of the epidermal layer. These leader cells extend lamella protrusion to the migratory direction and form the driving force for the epithelial sheet. The follower cells back from the leading edge are activated, and in turn contribute to forward migration of the keratinocytes sheet. In these leader cells and followers, coordinated interplay between protrusion extension, adhesion dissolution, and stress fibers disassembly releases epithelial tension and provides space for cells to migrate effectively^5,44^. We observed an EMT-like switch in healing epidermal sheet with downregulation of epithelial marker E-cadherin in keratinocytes. Accordingly, when the leader cells at the wound edge migrated, cell–cell junctions dissolved within the leader and follower cell groups (Fig. 6).

The regulatory roles of miRNAs in skin disorders, such as psoriasis, scleroderma, and dermatomyositis have been widely reported^45^. The importance of miRNAs in regulating cutaneous wound healing has also been emphasized by recent studies^21^. With respective to miR-200s, they have been shown to regulate epithelial intercellular junction and hair follicle morphogenesis^19^. Our present study emphasizes the regulatory role of miR-200b/c-3p for keratinocyte cell migration involving lamellipodia formation (Fig. 5). Furthermore, we report that the modulation of cell–cell cohesion by miR-200b/c-3p is important for collective epidermal keratinocyte migration (Fig. 6). These findings reveal the cellular function of miR-200b/c-3p in the regulation of epidermal repair by modulating keratinocyte plasticity.

Cell motility is tightly regulated by the Rho family GTPases, which are pivotal regulators of cytoskeleton, cell junction, and cell migration^46,47^. RAC1 promotes lamella protrusion extension but inhibits adherens junction by endocytosis of E-cadherin, when RHOA is required for stress fiber formation^7,10^. Here we find RAC1 a direct target of miR-200b/c-3p in keratinocytes. Overexpression of miR-200b/c-3p inhibits the formation of lamellipodia in keratinocytes, phenocopying loss of function of RAC1 in keratinocytes^11^. In the wound margin, defects of lamellipodia formation in leader cells failed to generate a forward force (Fig. 5). In addition, to fulfil their role in force transmission between cells, lateral cell–cell junctions are connected to the actin cytoskeleton and wound re-epithelialization requires dissolve of intercellular junctions which influences epidermal sheet migration. In contrast to the finding that activation of RAC1 in the leader and follower cells compromised adherens junctions, RAC1 deficiency lead to sustained cell adherens junction^29^. In line with the finding that miR-200b/c-3p represses RAC1, overexpression of miR-200b/c-3p inhibits the intercellular junction dissolution in keratinocytes and antagonize the collective cell migration in the wound edge (Fig. 6). Moreover, In RAC1 deficient cells or miR-200b/c-3p-overexpressing cells several rows behind the leading edge, stress fiber formation is more obvious than control (Fig. 5). Since RHOA and RAC1 antagonism was observed in migrating cells^48^, RAC1 inhibition may result in high RHOA activity that mediates stress fiber formation leading to drawing tension between cells. Therefore, the migration phenotype of these follower cells might be due to RHOA-mediated failure of relaxation of epithelial tension in migrating keratinocytes^5^. In this context, the repression of RAC1 signaling might dampen collective cell migration by inhibition of both adhesion dissolution and stress fiber disassembly. Taken together, we demonstrated that miR-200b/c-3p regulated cell migration by modulating actin cytoskeleton and cell junctions through targeting the RAC1 signaling pathway. Interestingly, CDC42 is also a predicted target for miR-200a-3p (data not shown). Whether miR-200a-3p regulation of CDC42-mediated filopodia formation plays a role for keratinocyte migration in skin wounds awaits further study.

Our data and others showed that the epidermis-enriched miR-200b/c-3p were downregulated upon cutaneous wounding^22^. However, in aged skin wound, miR-200c-3p remained highly expressed during the healing process^26^. Considering our observed anti-migratory effect of miR-200c-3p on keratinocytes, it is conceivable that high miR-200c-3p hinders efficient healing of aged skin wounds. Thus, miR-200c-3p may serve as a potential therapeutic target by using a knock down strategy in aged skin wound^21,49^. However, the mechanism that maintains high miR-200c-3p expression in aged skin wound remains unexplored. TGF-β is potent regulator of keratinocyte plasticity through both autocrine and paracrine mechanisms during cutaneous wound healing^50^. Interestingly, keratinocytes tend to migrate in sheets to close wound and thus undergo an incomplete EMT. This induction of keratinocytes partial EMT is also attributable to TGF-β stimulation. As a potent stimulator of cell migration, TGF-β1 activates RAC1 signaling pathways^29^. We identified TGF-β1 acting as an upstream signal to suppress miR-200b/c-3p expression in keratinocytes, leading to a de-repression of keratinocyte migration (Fig. 4). It will be desirable to test whether dysregulation of TGF-β1 contributes to the sustained expression of miR-200c-3p in chronic wound. In line with this notion, TGF-β signaling is impaired in aged skin or chronic wound^38,51,52^. In addition, altered expression of a number of miRNAs has been found in chronic wounds such as diabetic or aged skin wounds^21,26,53^. It is worthy to define how the overall miRNA profile changes in the context of aged skin or chronic wounds with altered TGF-β signaling. TGF-β has been reported to repress miR-200s by ZEB in epithelial tumor cells^54^. Moreover, the expression of Slug, which belongs to the Snail superfamily of transcriptional repressors downstream of TGF-β signaling, is elevated in keratinocytes at the wound margins^55^. It will be interesting to clarify whether TGF-β1 represses miR-200b/c-3p by ZEB or Snail family transcription factors in the healing epidermis. Furthermore, the canonical TGF-β-induced signals such as SMAD may play a role in TGF-β-regulated miR-200b/c-3p expression and wound healing^56,57^. Lastly, recent studies report that TGF-β has opposing regulatory effects on a broad spectrum of miRNAs in skin wound. When TGF-β induces the expression of miR-21, miR-31, and miR-132, it represses miR-198 to promote wound healing^23,25,58,59^. How TGF-β exerts opposing effect on the expression of miRNAs and coordinates to regulate the healing process is an interesting question awaits answer.

In conclusion, our study reveals a pathway that miR-200b/c-3p inhibit RAC1 by direct targeting to its 3′UTR in keratinocytes. TGF-β1 stimulates RAC1 signaling by repressing miR-200b/c-3p expression and promotes keratinocyte migration, thus enhancing the re-epithelialization process. Therefore, we suggest that inhibition of miR-200b/c-3p function represents a novel therapeutic approach for improving wound healing.

## Supplementary information

Supplementary Table Legends

Supplementary Table 1

Supplementary Table 2

Supplementary Table 3
