# Supplementary material for: MicroRNA-200b/c-3p regulate epithelial plasticity and inhibit cutaneous wound healing by modulating TGF-β-mediated RAC1 signaling
Source: Cell Death Dis. 2020 Oct 29;11(10):931. doi: 10.1038/s41419-020-03132-2 (PMC7596237; doi:10.1038/s41419-020-03132-2)
Supplement: Supplementary file 1 — Supplementary Table Legends [file 41419_2020_3132_MOESM1_ESM.docx]

**Supplementary Table Legends**

**Supplementary Table 1.** **Nucleic acids sequences.** Sequences of miRNA mimics, miRNA inhibitors, primers for RT-qPCR, vector construction and mutagenesis are listed.

**Supplementary Table 2.** **Predicted miR-200b/c-3p target genes.** Listed 1196 and 2690 miR-200b/c-3p target genes were predicted by TargetScan and DIANA microT, respectively. Gene symbols of 987 shared putative target genes by the two algorithms are listed.

**Supplementary Table 3.** **Enriched pathways for predicted miR-200b/c-3p target genes.** Gene ontology analysis of 987 putative miR-200b/c-3p target genes by DAVID showed pathway enrichment in the databases of BIOCARTA, KEGG and REACTOME.
