# Supplementary material for: MicroRNA-200b/c-3p regulate epithelial plasticity and inhibit cutaneous wound healing by modulating TGF-β-mediated RAC1 signaling
Source: Cell Death Dis. 2020 Oct 29;11(10):931. doi: 10.1038/s41419-020-03132-2 (PMC7596237; doi:10.1038/s41419-020-03132-2)
Supplement: Supplementary file 2 — Supplementary Table 1 [file 41419_2020_3132_MOESM2_ESM.docx]

**Supplementary Table 1. Nucleic acids sequences.**

| **miRNA mimics** | **Accession** | **Sequences** (start from 5’ end) |
| --- | --- | --- |
| Negative control | Ribobio Catalog No. miR1N0000002 | N/A (Not available) |
|  |  |  |
| miR-200b-3p | MIMAT0000318 | Sense: UAAUACUGCCUGGUAAUGAUGA |
|  |  | Antisense: UCAUCAUUACCAGGCAGUAUUA |
|  |  |  |
| miR-200c-3p | MIMAT0000617 | Sense: UAAUACUGCCGGGUAAUGAUGGA |
|  |  | Antisense: UCCAUCAUUACCCGGCAGUAUUA |
|  |  |  |
| **miRNA inhibitors** |  |  |
| Negative control | Ribobio Catalog No. miR2N0000002 | N/A |
|  |  |  |
| miR-200b-3p | MIMAT0000318 | UCAUCAUUACCAGGCAGUAUUA |
|  |  |  |
| miR-200c-3p | MIMAT0000617 | UCCAUCAUUACCCGGCAGUAUUA |
|  |  |  |
| **siRNA** |  |  |
| siRAC1 | NM_006908.5 | Sense: UUUACCUACAGCUCCGUCUdTdT |
|  |  | Antisense: AGACGGAGCUGUAGGUAAAdTdT |
|  |  |  |
| **Primers for RT-qPCR** |  |  |
| miR-200b-3p | MIMAT0000318 | Forward: TAATACTGCCTGGTAAT |
|  |  | Reverse: GTGCAGGGTCCGAGGT |
|  |  |  |
|  |  | RT: GTCGTATCCAGTGCAGGGTCCGAGGTATTC GCACTGGATACGACTCATCA |
|  |  |  |
| miR-200c-3p | MIMAT0000617 | Forward: TAATACTGCCGGGTAATG |
|  |  | Reverse: GTGCAGGGTCCGAGGT |
|  |  |  |
|  |  | RT: GTCGTATCCAGTGCAGGGTCCGAGGTATTC  GCACTGGATACGACTCCATC |
|  |  |  |
| U6 | NR_004394.1 | Forward: ATACAGAGAAGATTAGCATGGCC |
|  |  | Reverse: CGAATTTGCGTGTCATCCTTG |
|  |  |  |
| *Tgfb1* | NM_011577.2 | Forward: TCGCTTTGTACAACAGCACC |
|  |  | Reverse: ACTGCTTCCCGAATGTCTGA |
|  |  |  |
| *Tgfb2* | NM_009367.4 | Forward: AGGCAGAGTTCAGGGTCTTC |
|  |  | Reverse: GTCTGTCACGTCGAAGGAGA |
|  |  |  |
| *Gapdh* | NM_001289726.1 | Forward: ATCACTGCCACCCAGAAGACT |
|  |  | Reverse: AGGTGGAAGAGTGGGAGTTGC |
|  |  |  |
| **Primers for vector construction** | |  |
| psiCHECK2-200luc | N/A | Forward: TCGAGTCATCATTACCCGGCAGTATTAGTT |
|  |  | TAAACTCATCATTACCCGGCAGTATTAGC |
|  |  | Reverse: GGCCGCTAATACTGCCGGGTAATGATGAG |
|  |  | TTTAAACTAATACTGCCGGGTAATGATGAC |
|  |  |  |
| psiCHECK2-RAC1-WT | NM_006908.5 | Forward: CATACTCGAGCCCTTGGAACCTTTGTACGC |
|  |  | Reverse: ATAAGCGGCCGCTGTTGTAGTGGCTGA AGGGT |
|  |  |  |
| psiCHECK2-RAC1-Mut | NM_006908.5 | Forward: GAGTCATAATTGACAAAATACGAAGTGGAG |
|  |  | Reverse: TTTGTCCTTTGATCACACAC |
|  |  |  |
| pcDNA6.2-miR-200b-3p | MIMAT0000318 | Forward: CGTGAATTCTTGAGAAGAGAAGGGGCTGG |
|  |  | Reverse: TCTGCTCGAGGCGTCCTAATCCCCAGATCA |
|  |  |  |
| pcDNA6.2-miR-200c-3p | MIMAT0000617 | Forward: CGTGAATTCGGGTAAATCGGTGTGTGTCG |
|  |  | Reverse: TCTGCTCGAGCCTGAAGGTTACTGCCGAGA |
|  |  |  |
