# Supplementary material for: MicroRNA-200b/c-3p regulate epithelial plasticity and inhibit cutaneous wound healing by modulating TGF-β-mediated RAC1 signaling
Source: Cell Death Dis. 2020 Oct 29;11(10):931. doi: 10.1038/s41419-020-03132-2 (PMC7596237; doi:10.1038/s41419-020-03132-2)
Supplement: Supplementary file 3 — Supplementary Table 2 [file 41419_2020_3132_MOESM3_ESM.docx]

| **Supplementary Table 2. Predicted miR-200b/c-3p target genes.** | | |
| --- | --- | --- |
| TargetScan |  |  |
| <http://www.targetscan.org/vert_72/> | | |
| DIANA microT |  |  |
| <http://diana.imis.athena-innovation.gr/DianaTools/index.php?r=microT_CDS/index> | | |
| **TargetScan prediction** |  |  |
| **Gene symbol** | **Gene name** | **Representative transcript** |
| ZEB1 | zinc finger E-box binding homeobox 1 | ENST00000361642.5 |
| FAM8A1 | family with sequence similarity 8, member A1 | ENST00000259963.3 |
| GMFB | glia maturation factor, beta | ENST00000554908.1 |
| TCEB1 | transcription elongation factor B (SIII), polypeptide 1 (15kDa, elongin C) | ENST00000518127.1 |
| FEZ2 | fasciculation and elongation protein zeta 2 (zygin II) | ENST00000379245.4 |
| CFL2 | cofilin 2 (muscle) | ENST00000341223.3 |
| LHFP | lipoma HMGIC fusion partner | ENST00000379589.3 |
| PMAIP1 | phorbol-12-myristate-13-acetate-induced protein 1 | ENST00000316660.6 |
| RAP1B | RAP1B, member of RAS oncogene family | ENST00000250559.9 |
| INTS8 | integrator complex subunit 8 | ENST00000523731.1 |
| DZIP1 | DAZ interacting zinc finger protein 1 | ENST00000347108.3 |
| GPM6A | glycoprotein M6A | ENST00000280187.7 |
| AP1S2 | adaptor-related protein complex 1, sigma 2 subunit | ENST00000329235.2 |
| KIAA0101 | KIAA0101 | ENST00000558008.1 |
| HIPK3 | homeodomain interacting protein kinase 3 | ENST00000303296.4 |
| ZFPM2 | zinc finger protein, FOG family member 2 | ENST00000407775.2 |
| CNEP1R1 | CTD nuclear envelope phosphatase 1 regulatory subunit 1 | ENST00000562576.1 |
| SEC23A | Sec23 homolog A (S. cerevisiae) | ENST00000537403.1 |
| PPP4R2 | protein phosphatase 4, regulatory subunit 2 | ENST00000356692.5 |
| HMGB3 | high mobility group box 3 | ENST00000325307.7 |
| CCNJ | cyclin J | ENST00000265992.5 |
| NR5A2 | nuclear receptor subfamily 5, group A, member 2 | ENST00000367362.3 |
| ERRFI1 | ERBB receptor feedback inhibitor 1 | ENST00000377482.5 |
| APOO | apolipoprotein O | ENST00000379226.4 |
| VASH2 | vasohibin 2 | ENST00000366968.4 |
| SERPINI1 | serpin peptidase inhibitor, clade I (neuroserpin), member 1 | ENST00000446050.2 |
| PAPD5 | PAP associated domain containing 5 | ENST00000357464.3 |
| PTHLH | parathyroid hormone-like hormone | ENST00000395872.1 |
| RECK | reversion-inducing-cysteine-rich protein with kazal motifs | ENST00000377966.3 |
| GTF2E1 | general transcription factor IIE, polypeptide 1, alpha 56kDa | ENST00000283875.5 |
| TMEFF2 | transmembrane protein with EGF-like and two follistatin-like domains 2 | ENST00000392314.1 |
| YWHAG | tyrosine 3-monooxygenase/tryptophan 5-monooxygenase activation protein, gamma polypeptide | ENST00000307630.3 |
| RASSF8 | Ras association (RalGDS/AF-6) domain family (N-terminal) member 8 | ENST00000541490.1 |
| RAP2C | RAP2C, member of RAS oncogene family | ENST00000342983.2 |
| ZFAND6 | zinc finger, AN1-type domain 6 | ENST00000261749.6 |
| PRKACB | protein kinase, cAMP-dependent, catalytic, beta | ENST00000370689.2 |
| PTPN21 | protein tyrosine phosphatase, non-receptor type 21 | ENST00000328736.3 |
| C16orf52 | chromosome 16 open reading frame 52 | ENST00000542527.2 |
| NOG | noggin | ENST00000332822.4 |
| MMD | monocyte to macrophage differentiation-associated | ENST00000262065.3 |
| CNN3 | calponin 3, acidic | ENST00000370206.4 |
| IMMP2L | IMP2 inner mitochondrial membrane peptidase-like (S. cerevisiae) | ENST00000405709.2 |
| FLI1 | Fli-1 proto-oncogene, ETS transcription factor | ENST00000527786.2 |
| MCFD2 | multiple coagulation factor deficiency 2 | ENST00000444761.2 |
| CRKL | v-crk avian sarcoma virus CT10 oncogene homolog-like | ENST00000354336.3 |
| LBR | lamin B receptor | ENST00000272163.4 |
| ELAVL2 | ELAV like neuron-specific RNA binding protein 2 | ENST00000380110.4 |
| SESN1 | sestrin 1 | ENST00000436639.2 |
| ZEB2 | zinc finger E-box binding homeobox 2 | ENST00000558170.2 |
| YPEL2 | yippee-like 2 (Drosophila) | ENST00000312655.4 |
| SMIM5 | small integral membrane protein 5 | ENST00000375215.3 |
| MSN | moesin | ENST00000360270.5 |
| KDELC1 | KDEL (Lys-Asp-Glu-Leu) containing 1 | ENST00000376004.4 |
| RP6-24A23.6 | Uncharacterized protein | ENST00000563887.1 |
| SLC14A1 | solute carrier family 14 (urea transporter), member 1 | ENST00000321925.4 |
| PSAT1 | phosphoserine aminotransferase 1 | ENST00000376588.3 |
| RBFOX3 | RNA binding protein, fox-1 homolog (C. elegans) 3 | ENST00000583458.1 |
| EPS8 | epidermal growth factor receptor pathway substrate 8 | ENST00000543523.1 |
| CEP41 | centrosomal protein 41kDa | ENST00000223208.5 |
| TMOD3 | tropomodulin 3 (ubiquitous) | ENST00000308580.7 |
| MARCKS | myristoylated alanine-rich protein kinase C substrate | ENST00000368635.4 |
| DNAJB5 | DnaJ (Hsp40) homolog, subfamily B, member 5 | ENST00000545841.1 |
| XKR8 | XK, Kell blood group complex subunit-related family, member 8 | ENST00000373884.5 |
| RANBP10 | RAN binding protein 10 | ENST00000317506.3 |
| PRDM16 | PR domain containing 16 | ENST00000511072.1 |
| ZNF532 | zinc finger protein 532 | ENST00000336078.4 |
| C6orf120 | chromosome 6 open reading frame 120 | ENST00000332290.2 |
| NOVA1 | neuro-oncological ventral antigen 1 | ENST00000465357.2 |
| LPAR1 | lysophosphatidic acid receptor 1 | ENST00000374431.3 |
| USP27X | ubiquitin specific peptidase 27, X-linked | ENST00000508866.2 |
| LIN7B | lin-7 homolog B (C. elegans) | ENST00000391864.3 |
| B3GNT1 | UDP-GlcNAc:betaGal beta-1,3-N-acetylglucosaminyltransferase 1 | ENST00000311181.4 |
| LCA5 | Leber congenital amaurosis 5 | ENST00000369846.4 |
| OSTM1 | osteopetrosis associated transmembrane protein 1 | ENST00000193322.3 |
| WAPAL | wings apart-like homolog (Drosophila) | ENST00000298767.5 |
| PAIP2 | poly(A) binding protein interacting protein 2 | ENST00000394795.2 |
| ZNF131 | zinc finger protein 131 | ENST00000505606.2 |
| NTF3 | neurotrophin 3 | ENST00000423158.3 |
| CRTAP | cartilage associated protein | ENST00000320954.6 |
| CDK17 | cyclin-dependent kinase 17 | ENST00000543119.2 |
| SNAP25 | synaptosomal-associated protein, 25kDa | ENST00000254976.2 |
| BNC2 | basonuclin 2 | ENST00000380672.4 |
| SLC35F4 | solute carrier family 35, member F4 | ENST00000556826.1 |
| ASF1A | anti-silencing function 1A histone chaperone | ENST00000229595.5 |
| GIT2 | G protein-coupled receptor kinase interacting ArfGAP 2 | ENST00000355312.3 |
| CCDC82 | coiled-coil domain containing 82 | ENST00000278520.5 |
| TCAIM | T cell activation inhibitor, mitochondrial | ENST00000342649.4 |
| NRG1 | neuregulin 1 | ENST00000341377.5 |
| ADIPOR2 | adiponectin receptor 2 | ENST00000357103.4 |
| PPAPDC2 | phosphatidic acid phosphatase type 2 domain containing 2 | ENST00000381883.2 |
| ADD3 | adducin 3 (gamma) | ENST00000277900.8 |
| ETS1 | v-ets avian erythroblastosis virus E26 oncogene homolog 1 | ENST00000531611.1 |
| JHDM1D | jumonji C domain containing histone demethylase 1 homolog D (S. cerevisiae) | ENST00000397560.2 |
| FLII | flightless I homolog (Drosophila) | ENST00000327031.4 |
| SLIT2 | slit homolog 2 (Drosophila) | ENST00000504154.1 |
| DUSP1 | dual specificity phosphatase 1 | ENST00000239223.3 |
| CLIC4 | chloride intracellular channel 4 | ENST00000374379.4 |
| TRAPPC8 | trafficking protein particle complex 8 | ENST00000283351.4 |
| FAM118B | family with sequence similarity 118, member B | ENST00000533050.1 |
| TMEM17 | transmembrane protein 17 | ENST00000335390.5 |
| SCOC | short coiled-coil protein | ENST00000608372.1 |
| CACUL1 | CDK2-associated, cullin domain 1 | ENST00000369151.3 |
| JUN | jun proto-oncogene | ENST00000371222.2 |
| BAG6 | BCL2-associated athanogene 6 | ENST00000375976.4 |
| C16orf72 | chromosome 16 open reading frame 72 | ENST00000327827.7 |
| RAB21 | RAB21, member RAS oncogene family | ENST00000261263.3 |
| KIAA0087 | KIAA0087 | ENST00000242109.3 |
| SGCE | sarcoglycan, epsilon | ENST00000265735.7 |
| HS3ST3A1 | heparan sulfate (glucosamine) 3-O-sulfotransferase 3A1 | ENST00000284110.1 |
| GJC1 | gap junction protein, gamma 1, 45kDa | ENST00000426548.1 |
| SLK | STE20-like kinase | ENST00000335753.4 |
| SULF1 | sulfatase 1 | ENST00000458141.2 |
| SNAPC1 | small nuclear RNA activating complex, polypeptide 1, 43kDa | ENST00000216294.4 |
| KIAA1432 | KIAA1432 | ENST00000414202.2 |
| TFAP2A | transcription factor AP-2 alpha (activating enhancer binding protein 2 alpha) | ENST00000379613.3 |
| RANBP9 | RAN binding protein 9 | ENST00000011619.3 |
| CDH20 | cadherin 20, type 2 | ENST00000262717.4 |
| CTD-2510F5.6 | Uncharacterized protein | ENST00000577660.1 |
| RIMKLB | ribosomal modification protein rimK-like family member B | ENST00000357529.3 |
| TBC1D12 | TBC1 domain family, member 12 | ENST00000225235.4 |
| GATSL2 | GATS protein-like 2 | ENST00000426327.3 |
| BDP1 | B double prime 1, subunit of RNA polymerase III transcription initiation factor IIIB | ENST00000380675.2 |
| GPR27 | G protein-coupled receptor 27 | ENST00000304411.2 |
| STRN | striatin, calmodulin binding protein | ENST00000263918.4 |
| WASF3 | WAS protein family, member 3 | ENST00000335327.5 |
| FUBP1 | far upstream element (FUSE) binding protein 1 | ENST00000370767.1 |
| IER5 | immediate early response 5 | ENST00000367577.4 |
| EFNA1 | ephrin-A1 | ENST00000368407.3 |
| SLC6A1 | solute carrier family 6 (neurotransmitter transporter), member 1 | ENST00000287766.4 |
| KLHL14 | kelch-like family member 14 | ENST00000359358.4 |
| LPPR1 | Lipid phosphate phosphatase-related protein type 1 | ENST00000374874.3 |
| DNAJB9 | DnaJ (Hsp40) homolog, subfamily B, member 9 | ENST00000249356.3 |
| ZBTB7C | zinc finger and BTB domain containing 7C | ENST00000535628.2 |
| PPM1F | protein phosphatase, Mg2+/Mn2+ dependent, 1F | ENST00000263212.5 |
| UBE2V1 | ubiquitin-conjugating enzyme E2 variant 1 | ENST00000371657.5 |
| FBXO30 | F-box protein 30 | ENST00000237281.4 |
| MMD2 | monocyte to macrophage differentiation-associated 2 | ENST00000406755.1 |
| SRSF10 | serine/arginine-rich splicing factor 10 | ENST00000343255.5 |
| AGFG1 | ArfGAP with FG repeats 1 | ENST00000310078.8 |
| PPP2CA | protein phosphatase 2, catalytic subunit, alpha isozyme | ENST00000481195.1 |
| JKAMP | JNK1/MAPK8-associated membrane protein | ENST00000261247.9 |
| IAH1 | isoamyl acetate-hydrolyzing esterase 1 homolog (S. cerevisiae) | ENST00000470914.1 |
| GAL3ST1 | galactose-3-O-sulfotransferase 1 | ENST00000406955.1 |
| FUBP3 | far upstream element (FUSE) binding protein 3 | ENST00000319725.9 |
| ARL5A | ADP-ribosylation factor-like 5A | ENST00000295087.8 |
| FOXF1 | forkhead box F1 | ENST00000262426.4 |
| ANP32B | acidic (leucine-rich) nuclear phosphoprotein 32 family, member B | ENST00000339399.4 |
| EGLN1 | egl-9 family hypoxia-inducible factor 1 | ENST00000366641.3 |
| TWISTNB | TWIST neighbor | ENST00000222567.5 |
| C2ORF15 | Uncharacterized protein C2orf15 | ENST00000302513.2 |
| CEBPD | CCAAT/enhancer binding protein (C/EBP), delta | ENST00000408965.3 |
| HSPA13 | heat shock protein 70kDa family, member 13 | ENST00000285667.3 |
| PCMTD1 | protein-L-isoaspartate (D-aspartate) O-methyltransferase domain containing 1 | ENST00000360540.5 |
| PPFIA1 | protein tyrosine phosphatase, receptor type, f polypeptide (PTPRF), interacting protein (liprin), alpha 1 | ENST00000253925.7 |
| GUCY1A3 | guanylate cyclase 1, soluble, alpha 3 | ENST00000296518.7 |
| DNMT3B | DNA (cytosine-5-)-methyltransferase 3 beta | ENST00000344505.4 |
| PIGM | phosphatidylinositol glycan anchor biosynthesis, class M | ENST00000368090.2 |
| GNAQ | guanine nucleotide binding protein (G protein), q polypeptide | ENST00000286548.4 |
| MYB | v-myb avian myeloblastosis viral oncogene homolog | ENST00000367814.4 |
| CKAP4 | cytoskeleton-associated protein 4 | ENST00000378026.4 |
| RNF2 | ring finger protein 2 | ENST00000367510.3 |
| UBE2I | ubiquitin-conjugating enzyme E2I | ENST00000355803.4 |
| ARHGAP6 | Rho GTPase activating protein 6 | ENST00000380736.1 |
| RHOA | ras homolog family member A | ENST00000454011.2 |
| NANOS1 | nanos homolog 1 (Drosophila) | ENST00000425699.1 |
| CTDSPL2 | CTD (carboxy-terminal domain, RNA polymerase II, polypeptide A) small phosphatase like 2 | ENST00000260327.4 |
| MFAP5 | microfibrillar associated protein 5 | ENST00000359478.2 |
| PPAP2B | phosphatidic acid phosphatase type 2B | ENST00000371250.3 |
| BAG5 | BCL2-associated athanogene 5 | ENST00000299204.4 |
| RAB37 | RAB37, member RAS oncogene family | ENST00000392610.1 |
| PIN1 | peptidylprolyl cis/trans isomerase, NIMA-interacting 1 | ENST00000247970.4 |
| CHST9 | carbohydrate (N-acetylgalactosamine 4-0) sulfotransferase 9 | ENST00000580774.1 |
| KDR | kinase insert domain receptor (a type III receptor tyrosine kinase) | ENST00000263923.4 |
| FXR2 | fragile X mental retardation, autosomal homolog 2 | ENST00000250113.7 |
| NEDD1 | neural precursor cell expressed, developmentally down-regulated 1 | ENST00000266742.4 |
| HS3ST1 | heparan sulfate (glucosamine) 3-O-sulfotransferase 1 | ENST00000002596.5 |
| SPTSSA | serine palmitoyltransferase, small subunit A | ENST00000298130.4 |
| KLF10 | Kruppel-like factor 10 | ENST00000285407.6 |
| MIEF1 | mitochondrial elongation factor 1 | ENST00000325301.2 |
| FXR1 | fragile X mental retardation, autosomal homolog 1 | ENST00000357559.4 |
| COPS8 | COP9 signalosome subunit 8 | ENST00000354371.2 |
| EIF2S1 | eukaryotic translation initiation factor 2, subunit 1 alpha, 35kDa | ENST00000256383.4 |
| RDH10 | retinol dehydrogenase 10 (all-trans) | ENST00000240285.5 |
| ITM2B | integral membrane protein 2B | ENST00000378565.5 |
| FBXW7 | F-box and WD repeat domain containing 7, E3 ubiquitin protein ligase | ENST00000281708.4 |
| PALM2 | paralemmin 2 | ENST00000448454.2 |
| RND3 | Rho family GTPase 3 | ENST00000375734.2 |
| REV1 | REV1, polymerase (DNA directed) | ENST00000393445.3 |
| TOB1 | transducer of ERBB2, 1 | ENST00000499247.2 |
| FBXO33 | F-box protein 33 | ENST00000298097.7 |
| HDHD2 | haloacid dehalogenase-like hydrolase domain containing 2 | ENST00000300605.6 |
| SMG8 | SMG8 nonsense mediated mRNA decay factor | ENST00000300917.5 |
| THAP1 | THAP domain containing, apoptosis associated protein 1 | ENST00000345117.2 |
| SLC30A5 | solute carrier family 30 (zinc transporter), member 5 | ENST00000396591.3 |
| HNRNPD | heterogeneous nuclear ribonucleoprotein D (AU-rich element RNA binding protein 1, 37kDa) | ENST00000313899.7 |
| MXD3 | MAX dimerization protein 3 | ENST00000439742.2 |
| ABI2 | abl-interactor 2 | ENST00000295851.5 |
| ZDHHC17 | zinc finger, DHHC-type containing 17 | ENST00000426126.2 |
| CXorf23 | chromosome X open reading frame 23 | ENST00000379687.3 |
| ELL2 | elongation factor, RNA polymerase II, 2 | ENST00000237853.4 |
| PUM2 | pumilio RNA-binding family member 2 | ENST00000338086.5 |
| UBE2W | ubiquitin-conjugating enzyme E2W (putative) | ENST00000517608.1 |
| CASC4 | cancer susceptibility candidate 4 | ENST00000360824.3 |
| FSCN1 | fascin homolog 1, actin-bundling protein (Strongylocentrotus purpuratus) | ENST00000382361.3 |
| CERS6 | ceramide synthase 6 | ENST00000305747.6 |
| NFIB | nuclear factor I/B | ENST00000397575.3 |
| NDN | necdin, melanoma antigen (MAGE) family member | ENST00000331837.4 |
| ARGLU1 | arginine and glutamate rich 1 | ENST00000400198.3 |
| SNX30 | sorting nexin family member 30 | ENST00000374232.3 |
| GNPDA1 | glucosamine-6-phosphate deaminase 1 | ENST00000311337.6 |
| PPP1R18 | protein phosphatase 1, regulatory subunit 18 | ENST00000399199.3 |
| MAPRE1 | microtubule-associated protein, RP/EB family, member 1 | ENST00000375571.5 |
| CNTFR | ciliary neurotrophic factor receptor | ENST00000351266.4 |
| BTF3L4 | basic transcription factor 3-like 4 | ENST00000489308.2 |
| FLJ20373 |  | ENST00000414004.2 |
| CDKN1B | cyclin-dependent kinase inhibitor 1B (p27, Kip1) | ENST00000228872.4 |
| PSPH | phosphoserine phosphatase | ENST00000275605.3 |
| WDR91 | WD repeat domain 91 | ENST00000344400.5 |
| HSPA9 | heat shock 70kDa protein 9 (mortalin) | ENST00000297185.3 |
| ZCCHC24 | zinc finger, CCHC domain containing 24 | ENST00000372336.3 |
| BAP1 | BRCA1 associated protein-1 (ubiquitin carboxy-terminal hydrolase) | ENST00000460680.1 |
| CLASP1 | cytoplasmic linker associated protein 1 | ENST00000409078.3 |
| DGKA | diacylglycerol kinase, alpha 80kDa | ENST00000551156.1 |
| NFIA | nuclear factor I/A | ENST00000403491.3 |
| SIX1 | SIX homeobox 1 | ENST00000247182.6 |
| SIX3 | SIX homeobox 3 | ENST00000260653.3 |
| NLGN4X | neuroligin 4, X-linked | ENST00000381095.3 |
| TSC22D1 | TSC22 domain family, member 1 | ENST00000458659.2 |
| DCBLD2 | discoidin, CUB and LCCL domain containing 2 | ENST00000326840.6 |
| ARL2BP | ADP-ribosylation factor-like 2 binding protein | ENST00000219204.3 |
| PFDN4 | prefoldin subunit 4 | ENST00000371419.2 |
| RPS6KB1 | ribosomal protein S6 kinase, 70kDa, polypeptide 1 | ENST00000225577.4 |
| CNOT7 | CCR4-NOT transcription complex, subunit 7 | ENST00000361272.4 |
| PSIP1 | PC4 and SFRS1 interacting protein 1 | ENST00000380738.4 |
| SEMA6D | sema domain, transmembrane domain (TM), and cytoplasmic domain, (semaphorin) 6D | ENST00000355997.3 |
| HMBOX1 | homeobox containing 1 | ENST00000397358.3 |
| FOXG1 | forkhead box G1 | ENST00000382535.3 |
| TMCC1 | transmembrane and coiled-coil domain family 1 | ENST00000432054.2 |
| TMEM189-UBE2V1 | TMEM189-UBE2V1 readthrough | ENST00000341698.2 |
| RSPRY1 | ring finger and SPRY domain containing 1 | ENST00000394420.4 |
| SPATS2L | spermatogenesis associated, serine-rich 2-like | ENST00000358677.5 |
| SCHIP1 | schwannomin interacting protein 1 | ENST00000445224.2 |
| PI4K2B | phosphatidylinositol 4-kinase type 2 beta | ENST00000264864.6 |
| TMEM189 | transmembrane protein 189 | ENST00000557021.1 |
| SOX2 | SRY (sex determining region Y)-box 2 | ENST00000325404.1 |
| CKLF | chemokine-like factor | ENST00000264001.4 |
| MIB1 | mindbomb E3 ubiquitin protein ligase 1 | ENST00000261537.6 |
| KIAA1430 | KIAA1430 | ENST00000458385.2 |
| SGIP1 | SH3-domain GRB2-like (endophilin) interacting protein 1 | ENST00000371036.3 |
| PCNP | PEST proteolytic signal containing nuclear protein | ENST00000296024.5 |
| NRBP1 | nuclear receptor binding protein 1 | ENST00000233557.3 |
| LOX | lysyl oxidase | ENST00000231004.4 |
| TMEM33 | transmembrane protein 33 | ENST00000504986.1 |
| TRIM44 | tripartite motif containing 44 | ENST00000299413.5 |
| DNAJC3 | DnaJ (Hsp40) homolog, subfamily C, member 3 | ENST00000602402.1 |
| TSC22D2 | TSC22 domain family, member 2 | ENST00000361875.3 |
| FAM49B | family with sequence similarity 49, member B | ENST00000519824.2 |
| ARID4B | AT rich interactive domain 4B (RBP1-like) | ENST00000349213.3 |
| AEBP2 | AE binding protein 2 | ENST00000266508.9 |
| PCDH8 | protocadherin 8 | ENST00000377942.3 |
| SMARCAD1 | SWI/SNF-related, matrix-associated actin-dependent regulator of chromatin, subfamily a, containing DEAD/H box 1 | ENST00000354268.4 |
| GATA4 | GATA binding protein 4 | ENST00000335135.4 |
| BASP1 | brain abundant, membrane attached signal protein 1 | ENST00000322611.3 |
| SURF4 | surfeit 4 | ENST00000545297.1 |
| NCS1 | neuronal calcium sensor 1 | ENST00000372398.3 |
| MAP3K5 | mitogen-activated protein kinase kinase kinase 5 | ENST00000359015.4 |
| NXPH1 | neurexophilin 1 | ENST00000405863.1 |
| SPG20 | spastic paraplegia 20 (Troyer syndrome) | ENST00000438666.2 |
| ERI1 | exoribonuclease 1 | ENST00000250263.7 |
| GOLGA7 | golgin A7 | ENST00000405786.2 |
| SYS1 | SYS1 Golgi-localized integral membrane protein homolog (S. cerevisiae) | ENST00000243918.5 |
| PKIA | protein kinase (cAMP-dependent, catalytic) inhibitor alpha | ENST00000396418.2 |
| SCD | stearoyl-CoA desaturase (delta-9-desaturase) | ENST00000370355.2 |
| SOX1 | SRY (sex determining region Y)-box 1 | ENST00000330949.1 |
| VTI1B | vesicle transport through interaction with t-SNAREs 1B | ENST00000554659.1 |
| IDS | Iduronate 2-sulfatase (Hunter syndrome), isoform CRA_e; Iduronate 2-sulfatase 14 kDa chain; cDNA FLJ42669 fis, clone BRAMY2022168, highly similar to IDURONATE 2-SULFATASE | ENST00000422081.2 |
| MYLK | myosin light chain kinase | ENST00000360772.3 |
| KLF6 | Kruppel-like factor 6 | ENST00000542957.1 |
| FSTL1 | follistatin-like 1 | ENST00000295633.3 |
| MBNL3 | muscleblind-like splicing regulator 3 | ENST00000370839.3 |
| SLC35E2B | solute carrier family 35, member E2B | ENST00000378662.1 |
| ZBTB8A | zinc finger and BTB domain containing 8A | ENST00000316459.4 |
| ENSA | endosulfine alpha | ENST00000369014.5 |
| MTFR1 | mitochondrial fission regulator 1 | ENST00000458689.2 |
| ARHGEF3 | Rho guanine nucleotide exchange factor (GEF) 3 | ENST00000296315.3 |
| MEX3B | mex-3 RNA binding family member B | ENST00000558133.1 |
| ZSWIM4 | zinc finger, SWIM-type containing 4 | ENST00000254323.2 |
| XKR6 | XK, Kell blood group complex subunit-related family, member 6 | ENST00000304437.2 |
| COL4A3BP | collagen, type IV, alpha 3 (Goodpasture antigen) binding protein | ENST00000380494.5 |
| NUDT4 | nudix (nucleoside diphosphate linked moiety X)-type motif 4 | ENST00000337179.5 |
| SERINC1 | serine incorporator 1 | ENST00000339697.4 |
| CCNYL1 | cyclin Y-like 1 | ENST00000295414.3 |
| SSR3 | signal sequence receptor, gamma (translocon-associated protein gamma) | ENST00000476217.1 |
| EVI5 | ecotropic viral integration site 5 | ENST00000370331.1 |
| IKBKB | inhibitor of kappa light polypeptide gene enhancer in B-cells, kinase beta | ENST00000379708.3 |
| E2F3 | E2F transcription factor 3 | ENST00000346618.3 |
| GRAP2 | GRB2-related adaptor protein 2 | ENST00000344138.4 |
| NEGR1 | neuronal growth regulator 1 | ENST00000357731.5 |
| ADAMTS3 | ADAM metallopeptidase with thrombospondin type 1 motif, 3 | ENST00000286657.4 |
| LMO7 | LIM domain 7 | ENST00000341547.4 |
| IQCJ-SCHIP1 | IQCJ-SCHIP1 readthrough | ENST00000337808.6 |
| KLF4 | Kruppel-like factor 4 (gut) | ENST00000374672.4 |
| MBLAC2 | metallo-beta-lactamase domain containing 2 | ENST00000316610.6 |
| ING2 | inhibitor of growth family, member 2 | ENST00000302327.3 |
| ORMDL3 | ORM1-like 3 (S. cerevisiae) | ENST00000304046.2 |
| TMEM170B | transmembrane protein 170B | ENST00000379426.1 |
| NBR1 | neighbor of BRCA1 gene 1 | ENST00000542611.1 |
| PIK3CA | phosphatidylinositol-4,5-bisphosphate 3-kinase, catalytic subunit alpha | ENST00000263967.3 |
| TMEM237 | transmembrane protein 237 | ENST00000409444.2 |
| KIAA0040 | KIAA0040 | ENST00000423313.1 |
| AMOTL2 | angiomotin like 2 | ENST00000249883.5 |
| ARIH1 | ariadne RBR E3 ubiquitin protein ligase 1 | ENST00000379887.4 |
| GXYLT1 | glucoside xylosyltransferase 1 | ENST00000398675.3 |
| USP25 | ubiquitin specific peptidase 25 | ENST00000285681.2 |
| G6PC | glucose-6-phosphatase, catalytic subunit | ENST00000253801.2 |
| CHRDL1 | chordin-like 1 | ENST00000372045.1 |
| LRP1B | low density lipoprotein receptor-related protein 1B | ENST00000389484.3 |
| CDK2 | cyclin-dependent kinase 2 | ENST00000266970.4 |
| PAM | peptidylglycine alpha-amidating monooxygenase | ENST00000438793.3 |
| TMEM170A | transmembrane protein 170A | ENST00000357613.4 |
| SLC6A11 | solute carrier family 6 (neurotransmitter transporter), member 11 | ENST00000254488.2 |
| PLS3 | plastin 3 | ENST00000420625.2 |
| RBM12B | RNA binding motif protein 12B | ENST00000399300.2 |
| LFNG | LFNG O-fucosylpeptide 3-beta-N-acetylglucosaminyltransferase | ENST00000402045.1 |
| CSRNP3 | cysteine-serine-rich nuclear protein 3 | ENST00000314499.7 |
| SNAI2 | snail family zinc finger 2 | ENST00000020945.1 |
| CITED2 | Cbp/p300-interacting transactivator, with Glu/Asp-rich carboxy-terminal domain, 2 | ENST00000367651.2 |
| SYDE1 | synapse defective 1, Rho GTPase, homolog 1 (C. elegans) | ENST00000342784.2 |
| PHACTR3 | phosphatase and actin regulator 3 | ENST00000359926.3 |
| FAM81A | family with sequence similarity 81, member A | ENST00000288228.5 |
| CCSER1 | coiled-coil serine-rich protein 1 | ENST00000509176.1 |
| DIRAS2 | DIRAS family, GTP-binding RAS-like 2 | ENST00000375765.3 |
| UBE2D1 | ubiquitin-conjugating enzyme E2D 1 | ENST00000373910.4 |
| TRIM33 | tripartite motif containing 33 | ENST00000358465.2 |
| CBL | Cbl proto-oncogene, E3 ubiquitin protein ligase | ENST00000264033.4 |
| CALU | calumenin | ENST00000535011.2 |
| MARCH6 | membrane-associated ring finger (C3HC4) 6, E3 ubiquitin protein ligase | ENST00000274140.5 |
| BHLHE41 | basic helix-loop-helix family, member e41 | ENST00000242728.4 |
| RAPGEF2 | Rap guanine nucleotide exchange factor (GEF) 2 | ENST00000264431.4 |
| ANLN | anillin, actin binding protein | ENST00000265748.2 |
| LARP1B | La ribonucleoprotein domain family, member 1B | ENST00000441387.1 |
| MTSS1L | metastasis suppressor 1-like | ENST00000338779.6 |
| ACVR1C | activin A receptor, type IC | ENST00000243349.8 |
| ELF2 | E74-like factor 2 (ets domain transcription factor) | ENST00000394235.2 |
| PLCG1 | phospholipase C, gamma 1 | ENST00000244007.3 |
| KLF12 | Kruppel-like factor 12 | ENST00000377669.2 |
| TAF12 | TAF12 RNA polymerase II, TATA box binding protein (TBP)-associated factor, 20kDa | ENST00000373824.4 |
| RHOT1 | ras homolog family member T1 | ENST00000333942.6 |
| SRSF1 | serine/arginine-rich splicing factor 1 | ENST00000258962.4 |
| CORO1C | coronin, actin binding protein, 1C | ENST00000261401.3 |
| TBK1 | TANK-binding kinase 1 | ENST00000331710.5 |
| ARHGAP19 | Rho GTPase activating protein 19 | ENST00000358531.4 |
| SLITRK1 | SLIT and NTRK-like family, member 1 | ENST00000377084.2 |
| PCSK2 | proprotein convertase subtilisin/kexin type 2 | ENST00000377899.1 |
| SLC39A14 | solute carrier family 39 (zinc transporter), member 14 | ENST00000381237.1 |
| MXD4 | MAX dimerization protein 4 | ENST00000337190.2 |
| TAOK3 | TAO kinase 3 | ENST00000419821.2 |
| GPR146 | G protein-coupled receptor 146 | ENST00000297468.3 |
| ACE2 | angiotensin I converting enzyme 2 | ENST00000252519.3 |
| SELK | Selenoprotein K | ENST00000541726.1 |
| PLCL1 | phospholipase C-like 1 | ENST00000428675.1 |
| RUSC2 | RUN and SH3 domain containing 2 | ENST00000455600.1 |
| RAB11FIP2 | RAB11 family interacting protein 2 (class I) | ENST00000355624.3 |
| PHF6 | PHD finger protein 6 | ENST00000332070.3 |
| ZNF423 | zinc finger protein 423 | ENST00000561648.1 |
| RABEP1 | rabaptin, RAB GTPase binding effector protein 1 | ENST00000262477.6 |
| VEGFA | vascular endothelial growth factor A | ENST00000417285.2 |
| NREP | neuronal regeneration related protein | ENST00000379671.3 |
| KIF13A | kinesin family member 13A | ENST00000378814.5 |
| HLF | hepatic leukemia factor | ENST00000226067.5 |
| TLL2 | tolloid-like 2 | ENST00000357947.3 |
| DNAJC5 | DnaJ (Hsp40) homolog, subfamily C, member 5 | ENST00000360864.4 |
| GLI3 | GLI family zinc finger 3 | ENST00000395925.3 |
| FN1 | fibronectin 1 | ENST00000357009.2 |
| SCRT2 | scratch homolog 2, zinc finger protein (Drosophila) | ENST00000246104.6 |
| KBTBD6 | kelch repeat and BTB (POZ) domain containing 6 | ENST00000379485.1 |
| TTC33 | tetratricopeptide repeat domain 33 | ENST00000337702.4 |
| CDH11 | cadherin 11, type 2, OB-cadherin (osteoblast) | ENST00000394156.3 |
| S100PBP | S100P binding protein | ENST00000373475.5 |
| CNKSR3 | CNKSR family member 3 | ENST00000607772.1 |
| BAZ2B | bromodomain adjacent to zinc finger domain, 2B | ENST00000392782.1 |
| NUP35 | nucleoporin 35kDa | ENST00000295119.4 |
| FHOD1 | formin homology 2 domain containing 1 | ENST00000258201.4 |
| MSL2 | male-specific lethal 2 homolog (Drosophila) | ENST00000309993.2 |
| HNRNPH1 | heterogeneous nuclear ribonucleoprotein H1 (H) | ENST00000393432.4 |
| HS2ST1 | heparan sulfate 2-O-sulfotransferase 1 | ENST00000370550.5 |
| MBOAT2 | membrane bound O-acyltransferase domain containing 2 | ENST00000305997.3 |
| SLC16A2 | solute carrier family 16, member 2 (thyroid hormone transporter) | ENST00000587091.1 |
| FBXL17 | F-box and leucine-rich repeat protein 17 | ENST00000359660.5 |
| FNBP4 | formin binding protein 4 | ENST00000263773.5 |
| KIAA0355 | KIAA0355 | ENST00000299505.6 |
| SCAMP1 | secretory carrier membrane protein 1 | ENST00000538629.1 |
| PRKAR2B | protein kinase, cAMP-dependent, regulatory, type II, beta | ENST00000265717.4 |
| MAP4K3 | mitogen-activated protein kinase kinase kinase kinase 3 | ENST00000263881.3 |
| HNRNPU | heterogeneous nuclear ribonucleoprotein U (scaffold attachment factor A) | ENST00000444376.2 |
| PRKAR1A | protein kinase, cAMP-dependent, regulatory, type I, alpha | ENST00000589228.1 |
| KCTD15 | potassium channel tetramerization domain containing 15 | ENST00000284006.6 |
| SEMA3F | sema domain, immunoglobulin domain (Ig), short basic domain, secreted, (semaphorin) 3F | ENST00000002829.3 |
| SECISBP2L | SECIS binding protein 2-like | ENST00000559471.1 |
| GLIS2 | GLIS family zinc finger 2 | ENST00000262366.3 |
| TMEM229B | transmembrane protein 229B | ENST00000557006.1 |
| FAM219A | family with sequence similarity 219, member A | ENST00000379089.1 |
| PTPN12 | protein tyrosine phosphatase, non-receptor type 12 | ENST00000248594.6 |
| MGAT2 | mannosyl (alpha-1,6-)-glycoprotein beta-1,2-N-acetylglucosaminyltransferase | ENST00000305386.2 |
| LRP4 | low density lipoprotein receptor-related protein 4 | ENST00000378623.1 |
| PDIK1L | PDLIM1 interacting kinase 1 like | ENST00000374271.4 |
| RELN | reelin | ENST00000428762.1 |
| KHDRBS1 | KH domain containing, RNA binding, signal transduction associated 1 | ENST00000327300.7 |
| LMAN1 | lectin, mannose-binding, 1 | ENST00000251047.5 |
| GPR158 | G protein-coupled receptor 158 | ENST00000376351.3 |
| B3GNT2 | UDP-GlcNAc:betaGal beta-1,3-N-acetylglucosaminyltransferase 2 | ENST00000301998.4 |
| PPP6R3 | protein phosphatase 6, regulatory subunit 3 | ENST00000393800.2 |
| NFYA | nuclear transcription factor Y, alpha | ENST00000341376.6 |
| OCLN | occludin | ENST00000355237.2 |
| PHF21B | PHD finger protein 21B | ENST00000403565.1 |
| KIAA0895 | KIAA0895 | ENST00000297063.6 |
| KIAA2018 | KIAA2018 | ENST00000316407.4 |
| MMP16 | matrix metallopeptidase 16 (membrane-inserted) | ENST00000286614.6 |
| OSBPL11 | oxysterol binding protein-like 11 | ENST00000296220.5 |
| FRMD4B | FERM domain containing 4B | ENST00000398540.3 |
| WIPF3 | WAS/WASL interacting protein family, member 3 | ENST00000409290.1 |
| NCOA2 | nuclear receptor coactivator 2 | ENST00000452400.2 |
| PAN3 | PAN3 poly(A) specific ribonuclease subunit homolog (S. cerevisiae) | ENST00000282391.5 |
| RAB18 | RAB18, member RAS oncogene family | ENST00000356940.6 |
| ARL6IP6 | ADP-ribosylation-like factor 6 interacting protein 6 | ENST00000326446.5 |
| NYAP1 | neuronal tyrosine-phosphorylated phosphoinositide-3-kinase adaptor 1 | ENST00000300179.2 |
| SUGT1 | SGT1, suppressor of G2 allele of SKP1 (S. cerevisiae) | ENST00000310528.8 |
| PPP2R5E | protein phosphatase 2, regulatory subunit B', epsilon isoform | ENST00000337537.3 |
| CYTH1 | cytohesin 1 | ENST00000585509.1 |
| C11orf87 | chromosome 11 open reading frame 87 | ENST00000327419.6 |
| CUL4A | cullin 4A | ENST00000326335.4 |
| VCPIP1 | valosin containing protein (p97)/p47 complex interacting protein 1 | ENST00000310421.4 |
| FLT1 | fms-related tyrosine kinase 1 | ENST00000282397.4 |
| ZBTB12 | zinc finger and BTB domain containing 12 | ENST00000375527.2 |
| FRS2 | fibroblast growth factor receptor substrate 2 | ENST00000550389.1 |
| ZC3H6 | zinc finger CCCH-type containing 6 | ENST00000343936.4 |
| ARIH2 | ariadne RBR E3 ubiquitin protein ligase 2 | ENST00000356401.4 |
| PTPN11 | protein tyrosine phosphatase, non-receptor type 11 | ENST00000351677.2 |
| SDC2 | syndecan 2 | ENST00000302190.4 |
| CDYL | chromodomain protein, Y-like | ENST00000343762.5 |
| KANK1 | KN motif and ankyrin repeat domains 1 | ENST00000382303.1 |
| USP6NL | USP6 N-terminal like | ENST00000609104.1 |
| PLCXD3 | phosphatidylinositol-specific phospholipase C, X domain containing 3 | ENST00000377801.3 |
| TIMP2 | TIMP metallopeptidase inhibitor 2 | ENST00000585421.1 |
| ELMOD2 | ELMO/CED-12 domain containing 2 | ENST00000323570.3 |
| DHX36 | DEAH (Asp-Glu-Ala-His) box polypeptide 36 | ENST00000496811.1 |
| UBA6 | ubiquitin-like modifier activating enzyme 6 | ENST00000322244.5 |
| ZNF771 | zinc finger protein 771 | ENST00000319296.5 |
| VTI1A | vesicle transport through interaction with t-SNAREs 1A | ENST00000393077.2 |
| DDX26B | DEAD/H (Asp-Glu-Ala-Asp/His) box polypeptide 26B | ENST00000370752.4 |
| AMFR | autocrine motility factor receptor, E3 ubiquitin protein ligase | ENST00000290649.5 |
| PTGDR | prostaglandin D2 receptor (DP) | ENST00000553372.1 |
| ETS2 | v-ets avian erythroblastosis virus E26 oncogene homolog 2 | ENST00000360214.3 |
| GNAI3 | guanine nucleotide binding protein (G protein), alpha inhibiting activity polypeptide 3 | ENST00000369851.4 |
| ZNF292 | zinc finger protein 292 | ENST00000369577.3 |
| EIF5B | eukaryotic translation initiation factor 5B | ENST00000289371.6 |
| PTPN13 | protein tyrosine phosphatase, non-receptor type 13 (APO-1/CD95 (Fas)-associated phosphatase) | ENST00000436978.1 |
| MTF2 | metal response element binding transcription factor 2 | ENST00000370298.4 |
| PBX3 | pre-B-cell leukemia homeobox 3 | ENST00000342287.5 |
| RIMS2 | regulating synaptic membrane exocytosis 2 | ENST00000507740.1 |
| TAF1D | TATA box binding protein (TBP)-associated factor, RNA polymerase I, D, 41kDa | ENST00000448108.2 |
| EFNB2 | ephrin-B2 | ENST00000245323.4 |
| ZBTB38 | zinc finger and BTB domain containing 38 | ENST00000514251.1 |
| SYT1 | synaptotagmin I | ENST00000457153.2 |
| DCAF17 | DDB1 and CUL4 associated factor 17 | ENST00000375255.3 |
| PTPN14 | protein tyrosine phosphatase, non-receptor type 14 | ENST00000366956.5 |
| R3HDM2 | R3H domain containing 2 | ENST00000347140.3 |
| CPED1 | cadherin-like and PC-esterase domain containing 1 | ENST00000310396.5 |
| KRAS | Kirsten rat sarcoma viral oncogene homolog | ENST00000256078.4 |
| DUSP7 | dual specificity phosphatase 7 | ENST00000495880.1 |
| BMI1 | BMI1 polycomb ring finger oncogene | ENST00000376663.3 |
| CCNT2 | cyclin T2 | ENST00000295238.6 |
| CNTN4 | contactin 4 | ENST00000427331.1 |
| SRF | serum response factor (c-fos serum response element-binding transcription factor) | ENST00000265354.4 |
| FIGN | fidgetin | ENST00000333129.3 |
| RTF1 | Rtf1, Paf1/RNA polymerase II complex component, homolog (S. cerevisiae) | ENST00000389629.4 |
| ZNF711 | zinc finger protein 711 | ENST00000360700.4 |
| ATL2 | atlastin GTPase 2 | ENST00000406122.1 |
| RSRC2 | arginine/serine-rich coiled-coil 2 | ENST00000331738.7 |
| DIXDC1 | DIX domain containing 1 | ENST00000440460.2 |
| AC068987.1 | HCG1997999; cDNA FLJ33996 fis, clone DFNES2008881 | ENST00000599343.1 |
| TARDBP | TAR DNA binding protein | ENST00000240185.3 |
| RAC1 | ras-related C3 botulinum toxin substrate 1 (rho family, small GTP binding protein Rac1) | ENST00000348035.4 |
| PPP1R10 | protein phosphatase 1, regulatory subunit 10 | ENST00000376511.2 |
| DACH1 | dachshund homolog 1 (Drosophila) | ENST00000305425.4 |
| BICC1 | bicaudal C homolog 1 (Drosophila) | ENST00000373886.3 |
| PTBP1 | polypyrimidine tract binding protein 1 | ENST00000350092.4 |
| ZMAT3 | zinc finger, matrin-type 3 | ENST00000311417.2 |
| VLDLR | very low density lipoprotein receptor | ENST00000382100.3 |
| SLC1A2 | solute carrier family 1 (glial high affinity glutamate transporter), member 2 | ENST00000278379.3 |
| CBX4 | chromobox homolog 4 | ENST00000269397.4 |
| SMAD2 | SMAD family member 2 | ENST00000262160.6 |
| KLF9 | Kruppel-like factor 9 | ENST00000377126.2 |
| HOXA5 | homeobox A5 | ENST00000222726.3 |
| KMT2E | lysine (K)-specific methyltransferase 2E | ENST00000334877.4 |
| CHD2 | chromodomain helicase DNA binding protein 2 | ENST00000394196.4 |
| CSNK1G3 | casein kinase 1, gamma 3 | ENST00000360683.2 |
| PDS5B | PDS5, regulator of cohesion maintenance, homolog B (S. cerevisiae) | ENST00000315596.10 |
| QKI | QKI, KH domain containing, RNA binding | ENST00000392127.2 |
| ADCY2 | adenylate cyclase 2 (brain) | ENST00000338316.4 |
| RASA2 | RAS p21 protein activator 2 | ENST00000286364.3 |
| BRMS1L | breast cancer metastasis-suppressor 1-like | ENST00000216807.7 |
| DR1 | down-regulator of transcription 1, TBP-binding (negative cofactor 2) | ENST00000370272.4 |
| CDR2 | cerebellar degeneration-related protein 2, 62kDa | ENST00000268383.2 |
| CDC73 | cell division cycle 73 | ENST00000367435.3 |
| PIKFYVE | phosphoinositide kinase, FYVE finger containing | ENST00000264380.4 |
| GPR107 | G protein-coupled receptor 107 | ENST00000372410.3 |
| NPTX1 | neuronal pentraxin I | ENST00000306773.4 |
| GATA2 | GATA binding protein 2 | ENST00000341105.2 |
| CNOT4 | CCR4-NOT transcription complex, subunit 4 | ENST00000541284.1 |
| FOXF2 | forkhead box F2 | ENST00000259806.1 |
| ETV5 | ets variant 5 | ENST00000306376.5 |
| GBX2 | gastrulation brain homeobox 2 | ENST00000551105.1 |
| SLC24A4 | solute carrier family 24 (sodium/potassium/calcium exchanger), member 4 | ENST00000393265.2 |
| DNMT3A | DNA (cytosine-5-)-methyltransferase 3 alpha | ENST00000380746.4 |
| DLGAP2 | discs, large (Drosophila) homolog-associated protein 2 | ENST00000421627.2 |
| ATXN1 | ataxin 1 | ENST00000244769.4 |
| MED13 | mediator complex subunit 13 | ENST00000397786.2 |
| PHTF2 | putative homeodomain transcription factor 2 | ENST00000416283.2 |
| ZNF217 | zinc finger protein 217 | ENST00000371471.2 |
| ZNF280C | zinc finger protein 280C | ENST00000370978.4 |
| RPE | ribulose-5-phosphate-3-epimerase | ENST00000429907.1 |
| CCNA2 | cyclin A2 | ENST00000274026.5 |
| NOVA2 | neuro-oncological ventral antigen 2 | ENST00000263257.5 |
| ANKH | ANKH inorganic pyrophosphate transport regulator | ENST00000284268.6 |
| PARD6B | par-6 family cell polarity regulator beta | ENST00000371610.2 |
| NPC1 | Niemann-Pick disease, type C1 | ENST00000269228.5 |
| FAM60A | family with sequence similarity 60, member A | ENST00000539409.1 |
| HCFC2 | host cell factor C2 | ENST00000229330.4 |
| THRA | thyroid hormone receptor, alpha | ENST00000450525.2 |
| POLK | polymerase (DNA directed) kappa | ENST00000241436.4 |
| LAMC1 | laminin, gamma 1 (formerly LAMB2) | ENST00000258341.4 |
| COMMD3-BMI1 | COMMD3-BMI1 readthrough | ENST00000602390.1 |
| KIAA1456 | KIAA1456 | ENST00000524591.2 |
| VAT1L | vesicle amine transport 1-like | ENST00000302536.2 |
| DOT1L | DOT1-like histone H3K79 methyltransferase | ENST00000398665.3 |
| ZBTB5 | zinc finger and BTB domain containing 5 | ENST00000307750.4 |
| FUT4 | fucosyltransferase 4 (alpha (1,3) fucosyltransferase, myeloid-specific) | ENST00000358752.2 |
| CCDC177 | Homo sapiens coiled-coil domain containing 177 (CCDC177), mRNA. | ENST00000599174.1 |
| NCOA7 | nuclear receptor coactivator 7 | ENST00000392477.2 |
| MATR3 | matrin 3 | ENST00000510056.1 |
| FOXN2 | forkhead box N2 | ENST00000340553.3 |
| PIP4K2B | phosphatidylinositol-5-phosphate 4-kinase, type II, beta | ENST00000269554.3 |
| LRRTM3 | leucine rich repeat transmembrane neuronal 3 | ENST00000361320.4 |
| SYVN1 | synovial apoptosis inhibitor 1, synoviolin | ENST00000294256.8 |
| GALNT2 | UDP-N-acetyl-alpha-D-galactosamine:polypeptide N-acetylgalactosaminyltransferase 2 (GalNAc-T2) | ENST00000366672.4 |
| PIK3CB | phosphatidylinositol-4,5-bisphosphate 3-kinase, catalytic subunit beta | ENST00000477593.1 |
| CYP1B1 | cytochrome P450, family 1, subfamily B, polypeptide 1 | ENST00000260630.3 |
| MYZAP | myocardial zonula adherens protein | ENST00000267853.5 |
| EIF2B5 | eukaryotic translation initiation factor 2B, subunit 5 epsilon, 82kDa | ENST00000273783.3 |
| WDR45B | WD repeat domain 45B | ENST00000392325.4 |
| DCUN1D1 | DCN1, defective in cullin neddylation 1, domain containing 1 | ENST00000292782.4 |
| ZNF697 | zinc finger protein 697 | ENST00000421812.2 |
| KCND2 | potassium voltage-gated channel, Shal-related subfamily, member 2 | ENST00000331113.4 |
| WWC3 | WWC family member 3 | ENST00000380861.4 |
| CDYL2 | chromodomain protein, Y-like 2 | ENST00000570137.2 |
| GLIPR2 | GLI pathogenesis-related 2 | ENST00000396613.3 |
| PHF21A | PHD finger protein 21A | ENST00000257821.4 |
| PAG1 | phosphoprotein associated with glycosphingolipid microdomains 1 | ENST00000220597.4 |
| TBP | TATA box binding protein | ENST00000230354.6 |
| PVRL4 | poliovirus receptor-related 4 | ENST00000368012.3 |
| ZC3H15 | zinc finger CCCH-type containing 15 | ENST00000337859.6 |
| UNC119B | unc-119 homolog B (C. elegans) | ENST00000344651.4 |
| CDR2L | cerebellar degeneration-related protein 2-like | ENST00000337231.5 |
| NEK9 | NIMA-related kinase 9 | ENST00000238616.5 |
| NRBF2 | nuclear receptor binding factor 2 | ENST00000277746.6 |
| CAMSAP2 | calmodulin regulated spectrin-associated protein family, member 2 | ENST00000358823.2 |
| TMEM178A | transmembrane protein 178A | ENST00000281961.2 |
| TRIM62 | tripartite motif containing 62 | ENST00000291416.5 |
| UGCG | UDP-glucose ceramide glucosyltransferase | ENST00000374279.3 |
| INHBA | inhibin, beta A | ENST00000242208.4 |
| SPAST | spastin | ENST00000315285.3 |
| ST6GALNAC5 | ST6 (alpha-N-acetyl-neuraminyl-2,3-beta-galactosyl-1,3)-N-acetylgalactosaminide alpha-2,6-sialyltransferase 5 | ENST00000477717.1 |
| PHLDB1 | pleckstrin homology-like domain, family B, member 1 | ENST00000361417.2 |
| TUBB3 | Tubulin beta-3 chain | ENST00000315491.7 |
| VASH1 | vasohibin 1 | ENST00000167106.4 |
| CECR2 | cat eye syndrome chromosome region, candidate 2 | ENST00000400573.5 |
| PPP2R2C | protein phosphatase 2, regulatory subunit B, gamma | ENST00000335585.5 |
| ARRDC3 | arrestin domain containing 3 | ENST00000265138.3 |
| PTP4A1 | protein tyrosine phosphatase type IVA, member 1 | ENST00000370651.3 |
| GABPA | GA binding protein transcription factor, alpha subunit 60kDa | ENST00000354828.3 |
| TBC1D22B | TBC1 domain family, member 22B | ENST00000373491.3 |
| CTNND2 | catenin (cadherin-associated protein), delta 2 | ENST00000304623.8 |
| YWHAQ | tyrosine 3-monooxygenase/tryptophan 5-monooxygenase activation protein, theta polypeptide | ENST00000381844.4 |
| ZYG11B | zyg-11 family member B, cell cycle regulator | ENST00000294353.6 |
| TLN2 | talin 2 | ENST00000561311.1 |
| WBP1L | WW domain binding protein 1-like | ENST00000369889.4 |
| TTC5 | tetratricopeptide repeat domain 5 | ENST00000258821.3 |
| PRDM1 | PR domain containing 1, with ZNF domain | ENST00000369089.3 |
| NCOA3 | nuclear receptor coactivator 3 | ENST00000341724.6 |
| THSD7A | thrombospondin, type I, domain containing 7A | ENST00000423059.4 |
| GABBR2 | gamma-aminobutyric acid (GABA) B receptor, 2 | ENST00000259455.2 |
| N4BP2 | NEDD4 binding protein 2 | ENST00000261435.6 |
| ELK3 | ELK3, ETS-domain protein (SRF accessory protein 2) | ENST00000228741.3 |
| PIP4K2A | phosphatidylinositol-5-phosphate 4-kinase, type II, alpha | ENST00000376573.4 |
| SPAG9 | sperm associated antigen 9 | ENST00000262013.7 |
| GPR173 | G protein-coupled receptor 173 | ENST00000332582.4 |
| ANKRD28 | ankyrin repeat domain 28 | ENST00000399451.2 |
| TRHDE | thyrotropin-releasing hormone degrading enzyme | ENST00000261180.4 |
| ARID4A | AT rich interactive domain 4A (RBP1-like) | ENST00000431317.2 |
| GOLGA1 | golgin A1 | ENST00000373555.4 |
| AFF3 | AF4/FMR2 family, member 3 | ENST00000409236.2 |
| RIPK2 | receptor-interacting serine-threonine kinase 2 | ENST00000220751.4 |
| PEAK1 | pseudopodium-enriched atypical kinase 1 | ENST00000312493.4 |
| GPATCH8 | G patch domain containing 8 | ENST00000591680.1 |
| HOOK1 | hook microtubule-tethering protein 1 | ENST00000371208.3 |
| REEP1 | receptor accessory protein 1 | ENST00000165698.5 |
| GLCCI1 | glucocorticoid induced transcript 1 | ENST00000223145.5 |
| WDR82 | WD repeat domain 82 | ENST00000296490.3 |
| NEK10 | NIMA-related kinase 10 | ENST00000295720.6 |
| ZFAND5 | zinc finger, AN1-type domain 5 | ENST00000237937.3 |
| CACNA1C | calcium channel, voltage-dependent, L type, alpha 1C subunit | ENST00000399655.1 |
| C10orf118 | chromosome 10 open reading frame 118 | ENST00000543782.1 |
| PDPK1 | 3-phosphoinositide dependent protein kinase-1 | ENST00000441549.3 |
| SBSPON | somatomedin B and thrombospondin, type 1 domain containing | ENST00000297354.6 |
| PPP1R9B | protein phosphatase 1, regulatory subunit 9B | ENST00000316878.6 |
| ATP11C | ATPase, class VI, type 11C | ENST00000327569.3 |
| CHST2 | carbohydrate (N-acetylglucosamine-6-O) sulfotransferase 2 | ENST00000309575.3 |
| CLIP1 | CAP-GLY domain containing linker protein 1 | ENST00000361654.4 |
| PPFIBP1 | PTPRF interacting protein, binding protein 1 (liprin beta 1) | ENST00000318304.8 |
| STARD13 | StAR-related lipid transfer (START) domain containing 13 | ENST00000336934.5 |
| ZMYM4 | zinc finger, MYM-type 4 | ENST00000314607.6 |
| XIAP | X-linked inhibitor of apoptosis | ENST00000371199.3 |
| MOB4 | MOB family member 4, phocein | ENST00000233892.4 |
| UBQLN1 | ubiquilin 1 | ENST00000376395.4 |
| FAM19A5 | family with sequence similarity 19 (chemokine (C-C motif)-like), member A5 | ENST00000358295.5 |
| ACTR3 | ARP3 actin-related protein 3 homolog (yeast) | ENST00000263238.2 |
| SDK2 | sidekick cell adhesion molecule 2 | ENST00000392650.3 |
| PCSK5 | proprotein convertase subtilisin/kexin type 5 | ENST00000376752.4 |
| CASD1 | CAS1 domain containing 1 | ENST00000297273.4 |
| FERMT2 | fermitin family member 2 | ENST00000395631.2 |
| C7orf43 | chromosome 7 open reading frame 43 | ENST00000394035.2 |
| APAF1 | apoptotic peptidase activating factor 1 | ENST00000333991.1 |
| SMAD9 | SMAD family member 9 | ENST00000399275.2 |
| HIPK1 | homeodomain interacting protein kinase 1 | ENST00000369558.1 |
| KCND3 | potassium voltage-gated channel, Shal-related subfamily, member 3 | ENST00000369697.1 |
| OXR1 | oxidation resistance 1 | ENST00000312046.6 |
| FBXO22 | F-box protein 22 | ENST00000308275.3 |
| EGR3 | early growth response 3 | ENST00000519492.1 |
| DOCK4 | dedicator of cytokinesis 4 | ENST00000428084.1 |
| MAP3K1 | mitogen-activated protein kinase kinase kinase 1, E3 ubiquitin protein ligase | ENST00000399503.3 |
| DLC1 | deleted in liver cancer 1 | ENST00000276297.4 |
| MARCH8 | membrane-associated ring finger (C3HC4) 8, E3 ubiquitin protein ligase | ENST00000453424.2 |
| CASR | calcium-sensing receptor | ENST00000498619.1 |
| PAK6 | p21 protein (Cdc42/Rac)-activated kinase 6 | ENST00000260404.4 |
| WASF1 | WAS protein family, member 1 | ENST00000392587.2 |
| KIAA1462 | KIAA1462 | ENST00000375377.1 |
| ZIC3 | Zic family member 3 | ENST00000287538.5 |
| KANK2 | KN motif and ankyrin repeat domains 2 | ENST00000586659.1 |
| LPIN1 | lipin 1 | ENST00000256720.2 |
| SOX5 | SRY (sex determining region Y)-box 5 | ENST00000546136.1 |
| SCN3B | sodium channel, voltage-gated, type III, beta subunit | ENST00000392770.2 |
| CHSY1 | chondroitin sulfate synthase 1 | ENST00000254190.3 |
| ARL14EPL | ADP-ribosylation factor-like 14 effector protein-like | ENST00000601302.2 |
| SHC1 | SHC (Src homology 2 domain containing) transforming protein 1 | ENST00000368445.5 |
| ARHGEF17 | Rho guanine nucleotide exchange factor (GEF) 17 | ENST00000263674.3 |
| CASZ1 | castor zinc finger 1 | ENST00000377022.3 |
| FGD1 | FYVE, RhoGEF and PH domain containing 1 | ENST00000375135.3 |
| TBX5 | T-box 5 | ENST00000349716.5 |
| C6orf62 | chromosome 6 open reading frame 62 | ENST00000378119.4 |
| SCN1A | sodium channel, voltage-gated, type I, alpha subunit | ENST00000423058.2 |
| DESI1 | desumoylating isopeptidase 1 | ENST00000263256.6 |
| SLC4A7 | solute carrier family 4, sodium bicarbonate cotransporter, member 7 | ENST00000295736.5 |
| STRN3 | striatin, calmodulin binding protein 3 | ENST00000355683.5 |
| HSPE1-MOB4 | HSPE1-MOB4 readthrough | ENST00000604458.1 |
| CNST | consortin, connexin sorting protein | ENST00000366513.4 |
| ZNF236 | zinc finger protein 236 | ENST00000253159.8 |
| RBM26 | RNA binding motif protein 26 | ENST00000267229.7 |
| CHN2 | chimerin 2 | ENST00000222792.6 |
| GFI1 | growth factor independent 1 transcription repressor | ENST00000370332.1 |
| FYN | FYN oncogene related to SRC, FGR, YES | ENST00000368682.3 |
| WDFY3 | WD repeat and FYVE domain containing 3 | ENST00000322366.6 |
| ERG | v-ets avian erythroblastosis virus E26 oncogene homolog | ENST00000398905.1 |
| SH3GL1 | SH3-domain GRB2-like 1 | ENST00000269886.3 |
| ARHGAP20 | Rho GTPase activating protein 20 | ENST00000260283.4 |
| CACNA2D1 | calcium channel, voltage-dependent, alpha 2/delta subunit 1 | ENST00000356860.3 |
| ULK2 | unc-51 like autophagy activating kinase 2 | ENST00000395544.4 |
| PTCH1 | patched 1 | ENST00000430669.2 |
| CHD1 | chromodomain helicase DNA binding protein 1 | ENST00000284049.3 |
| PAK7 | p21 protein (Cdc42/Rac)-activated kinase 7 | ENST00000378423.1 |
| SMURF2 | SMAD specific E3 ubiquitin protein ligase 2 | ENST00000262435.9 |
| CNOT6 | CCR4-NOT transcription complex, subunit 6 | ENST00000393356.1 |
| TP53INP1 | tumor protein p53 inducible nuclear protein 1 | ENST00000448464.2 |
| TXLNG | taxilin gamma | ENST00000380122.5 |
| ZNF662 | zinc finger protein 662 | ENST00000541208.1 |
| TMEM164 | transmembrane protein 164 | ENST00000372073.1 |
| PPP1R12B | protein phosphatase 1, regulatory subunit 12B | ENST00000608999.1 |
| ZDHHC18 | zinc finger, DHHC-type containing 18 | ENST00000374142.4 |
| CDX4 | caudal type homeobox 4 | ENST00000373514.2 |
| CPT1A | carnitine palmitoyltransferase 1A (liver) | ENST00000265641.5 |
| PPP1CB | protein phosphatase 1, catalytic subunit, beta isozyme | ENST00000395366.2 |
| ICK | intestinal cell (MAK-like) kinase | ENST00000350082.5 |
| TENM1 | teneurin transmembrane protein 1 | ENST00000371130.3 |
| PRKG1 | protein kinase, cGMP-dependent, type I | ENST00000373985.1 |
| SRP72 | signal recognition particle 72kDa | ENST00000342756.5 |
| JAZF1 | JAZF zinc finger 1 | ENST00000283928.5 |
| FMR1 | fragile X mental retardation 1 | ENST00000370471.3 |
| RBM20 | RNA binding motif protein 20 | ENST00000369519.3 |
| DACT1 | dishevelled-binding antagonist of beta-catenin 1 | ENST00000395153.3 |
| LEMD3 | LEM domain containing 3 | ENST00000308330.2 |
| MAP2 | microtubule-associated protein 2 | ENST00000360351.4 |
| KLF3 | Kruppel-like factor 3 (basic) | ENST00000261438.5 |
| NR3C1 | nuclear receptor subfamily 3, group C, member 1 (glucocorticoid receptor) | ENST00000394464.2 |
| FOXK1 | forkhead box K1 | ENST00000328914.4 |
| CALHM1 | calcium homeostasis modulator 1 | ENST00000329905.5 |
| PAPOLG | poly(A) polymerase gamma | ENST00000238714.3 |
| GOLIM4 | golgi integral membrane protein 4 | ENST00000470487.1 |
| ZBTB16 | zinc finger and BTB domain containing 16 | ENST00000335953.4 |
| ACTR1A | ARP1 actin-related protein 1 homolog A, centractin alpha (yeast) | ENST00000487599.1 |
| GAB1 | GRB2-associated binding protein 1 | ENST00000262995.4 |
| NKAP | NFKB activating protein | ENST00000371410.3 |
| FOSL2 | FOS-like antigen 2 | ENST00000379619.1 |
| SLC38A4 | solute carrier family 38, member 4 | ENST00000447411.1 |
| MYCN | v-myc avian myelocytomatosis viral oncogene neuroblastoma derived homolog | ENST00000281043.3 |
| PCNX | pecanex homolog (Drosophila) | ENST00000304743.2 |
| ABAT | 4-aminobutyrate aminotransferase | ENST00000569156.1 |
| BPTF | bromodomain PHD finger transcription factor | ENST00000321892.4 |
| CLASP2 | cytoplasmic linker associated protein 2 | ENST00000539981.1 |
| KIAA1468 | KIAA1468 | ENST00000398130.2 |
| SCN8A | sodium channel, voltage gated, type VIII, alpha subunit | ENST00000354534.6 |
| ITPR1 | inositol 1,4,5-trisphosphate receptor, type 1 | ENST00000302640.8 |
| CDH6 | cadherin 6, type 2, K-cadherin (fetal kidney) | ENST00000265071.2 |
| RNF38 | ring finger protein 38 | ENST00000259605.6 |
| HAS2 | hyaluronan synthase 2 | ENST00000303924.4 |
| CCDC117 | coiled-coil domain containing 117 | ENST00000249064.4 |
| NCMAP | noncompact myelin associated protein | ENST00000374392.2 |
| FGF18 | fibroblast growth factor 18 | ENST00000274625.5 |
| ATMIN | ATM interactor | ENST00000299575.4 |
| PI4KB | phosphatidylinositol 4-kinase, catalytic, beta | ENST00000368874.4 |
| SBF1 | SET binding factor 1 | ENST00000380817.3 |
| AKT2 | v-akt murine thymoma viral oncogene homolog 2 | ENST00000392038.2 |
| PFN2 | profilin 2 | ENST00000239940.7 |
| SMARCD1 | SWI/SNF related, matrix associated, actin dependent regulator of chromatin, subfamily d, member 1 | ENST00000394963.4 |
| KATNAL1 | katanin p60 subunit A-like 1 | ENST00000380615.3 |
| XKR4 | XK, Kell blood group complex subunit-related family, member 4 | ENST00000327381.6 |
| PKP1 | plakophilin 1 (ectodermal dysplasia/skin fragility syndrome) | ENST00000367324.3 |
| KCNJ15 | potassium inwardly-rectifying channel, subfamily J, member 15 | ENST00000328656.4 |
| RLF | rearranged L-myc fusion | ENST00000372771.4 |
| FRMD6 | FERM domain containing 6 | ENST00000395718.2 |
| DTNA | dystrobrevin, alpha | ENST00000283365.9 |
| DGKH | diacylglycerol kinase, eta | ENST00000261491.5 |
| BCL11B | B-cell CLL/lymphoma 11B (zinc finger protein) | ENST00000357195.3 |
| HNF1B | HNF1 homeobox B | ENST00000225893.4 |
| NKD1 | naked cuticle homolog 1 (Drosophila) | ENST00000268459.3 |
| ZNF362 | zinc finger protein 362 | ENST00000539719.1 |
| ITSN1 | intersectin 1 (SH3 domain protein) | ENST00000379960.5 |
| RLIM | ring finger protein, LIM domain interacting | ENST00000332687.6 |
| PKD1 | polycystic kidney disease 1 (autosomal dominant) | ENST00000262304.4 |
| INPP4A | inositol polyphosphate-4-phosphatase, type I, 107kDa | ENST00000409016.4 |
| ESRP1 | epithelial splicing regulatory protein 1 | ENST00000358397.5 |
| MFHAS1 | malignant fibrous histiocytoma amplified sequence 1 | ENST00000276282.6 |
| LRRC8A | leucine rich repeat containing 8 family, member A | ENST00000372600.4 |
| RPS6KA2 | ribosomal protein S6 kinase, 90kDa, polypeptide 2 | ENST00000265678.4 |
| LHX9 | LIM homeobox 9 | ENST00000367390.3 |
| DENND5B | DENN/MADD domain containing 5B | ENST00000389082.5 |
| OSR1 | odd-skipped related 1 (Drosophila) | ENST00000272223.2 |
| MIER3 | mesoderm induction early response 1, family member 3 | ENST00000381226.3 |
| NUDT3 | nudix (nucleoside diphosphate linked moiety X)-type motif 3 | ENST00000607016.1 |
| RAB23 | RAB23, member RAS oncogene family | ENST00000317483.3 |
| LEPROTL1 | leptin receptor overlapping transcript-like 1 | ENST00000321250.8 |
| TIAL1 | TIA1 cytotoxic granule-associated RNA binding protein-like 1 | ENST00000369093.2 |
| HCN1 | hyperpolarization activated cyclic nucleotide-gated potassium channel 1 | ENST00000303230.4 |
| SRSF2 | serine/arginine-rich splicing factor 2 | ENST00000392485.2 |
| CDC42EP3 | CDC42 effector protein (Rho GTPase binding) 3 | ENST00000295324.3 |
| HCCS | holocytochrome c synthase | ENST00000321143.4 |
| RBFOX1 | RNA binding protein, fox-1 homolog (C. elegans) 1 | ENST00000355637.4 |
| SFXN1 | sideroflexin 1 | ENST00000321442.5 |
| EXOC6B | exocyst complex component 6B | ENST00000272427.6 |
| GAN | gigaxonin | ENST00000568107.2 |
| RALGPS2 | Ral GEF with PH domain and SH3 binding motif 2 | ENST00000367635.3 |
| CSMD3 | CUB and Sushi multiple domains 3 | ENST00000343508.3 |
| YOD1 | YOD1 deubiquitinase | ENST00000315927.4 |
| NRIP1 | nuclear receptor interacting protein 1 | ENST00000400199.1 |
| ANK3 | ankyrin 3, node of Ranvier (ankyrin G) | ENST00000280772.2 |
| HNRNPK | heterogeneous nuclear ribonucleoprotein K | ENST00000376281.4 |
| OSMR | oncostatin M receptor | ENST00000274276.3 |
| SYNJ1 | synaptojanin 1 | ENST00000357345.3 |
| TMX4 | thioredoxin-related transmembrane protein 4 | ENST00000246024.2 |
| IGSF3 | immunoglobulin superfamily, member 3 | ENST00000369486.3 |
| COL4A3 | collagen, type IV, alpha 3 (Goodpasture antigen) | ENST00000396578.3 |
| EIF3J | eukaryotic translation initiation factor 3, subunit J | ENST00000261868.5 |
| BACH2 | BTB and CNC homology 1, basic leucine zipper transcription factor 2 | ENST00000257749.4 |
| CREB5 | cAMP responsive element binding protein 5 | ENST00000357727.2 |
| UBN2 | ubinuclein 2 | ENST00000473989.3 |
| CAB39 | calcium binding protein 39 | ENST00000258418.5 |
| SOAT1 | sterol O-acyltransferase 1 | ENST00000367619.3 |
| EFNA5 | ephrin-A5 | ENST00000333274.6 |
| IGF2R | insulin-like growth factor 2 receptor | ENST00000356956.1 |
| ESRRG | estrogen-related receptor gamma | ENST00000361525.3 |
| ATP2A2 | ATPase, Ca++ transporting, cardiac muscle, slow twitch 2 | ENST00000395494.2 |
| FAM168B | family with sequence similarity 168, member B | ENST00000409185.1 |
| DCAF5 | DDB1 and CUL4 associated factor 5 | ENST00000341516.5 |
| WDR12 | WD repeat domain 12 | ENST00000261015.4 |
| MYT1 | myelin transcription factor 1 | ENST00000328439.1 |
| SRRM4 | serine/arginine repetitive matrix 4 | ENST00000267260.4 |
| NPNT | nephronectin | ENST00000379987.2 |
| ASXL3 | additional sex combs like 3 (Drosophila) | ENST00000269197.5 |
| CLIP2 | CAP-GLY domain containing linker protein 2 | ENST00000223398.6 |
| DDX3Y | DEAD (Asp-Glu-Ala-Asp) box helicase 3, Y-linked | ENST00000336079.3 |
| TP73 | tumor protein p73 | ENST00000378280.1 |
| MOSPD2 | motile sperm domain containing 2 | ENST00000380492.3 |
| DNAJB14 | DnaJ (Hsp40) homolog, subfamily B, member 14 | ENST00000442697.2 |
| TRIO | trio Rho guanine nucleotide exchange factor | ENST00000344204.4 |
| SLC38A2 | solute carrier family 38, member 2 | ENST00000256689.5 |
| TRIM2 | tripartite motif containing 2 | ENST00000338700.5 |
| GDI2 | GDP dissociation inhibitor 2 | ENST00000380191.4 |
| DPY19L1 | dpy-19-like 1 (C. elegans) | ENST00000310974.4 |
| DCDC2 | doublecortin domain containing 2 | ENST00000378450.3 |
| PTEN | phosphatase and tensin homolog | ENST00000371953.3 |
| KCNB1 | potassium voltage-gated channel, Shab-related subfamily, member 1 | ENST00000371741.4 |
| IMPAD1 | inositol monophosphatase domain containing 1 | ENST00000262644.4 |
| RUNX1T1 | runt-related transcription factor 1; translocated to, 1 (cyclin D-related) | ENST00000523629.1 |
| GREM2 | gremlin 2, DAN family BMP antagonist | ENST00000318160.4 |
| SLC23A2 | solute carrier family 23 (ascorbic acid transporter), member 2 | ENST00000338244.1 |
| TMEM245 | transmembrane protein 245 | ENST00000374586.3 |
| TEC | tec protein tyrosine kinase | ENST00000381501.3 |
| NGEF | neuronal guanine nucleotide exchange factor | ENST00000264051.3 |
| EIF4E3 | eukaryotic translation initiation factor 4E family member 3 | ENST00000425534.3 |
| CYTH3 | cytohesin 3 | ENST00000350796.3 |
| LYSMD3 | LysM, putative peptidoglycan-binding, domain containing 3 | ENST00000509384.1 |
| FSTL4 | follistatin-like 4 | ENST00000265342.7 |
| CDK13 | cyclin-dependent kinase 13 | ENST00000181839.4 |
| SHROOM4 | shroom family member 4 | ENST00000376020.2 |
| MBD5 | methyl-CpG binding domain protein 5 | ENST00000407073.1 |
| NAPB | N-ethylmaleimide-sensitive factor attachment protein, beta | ENST00000377026.4 |
| NIP7 | NIP7, nucleolar pre-rRNA processing protein | ENST00000254940.5 |
| SEC61A2 | Sec61 alpha 2 subunit (S. cerevisiae) | ENST00000379020.4 |
| RNF169 | ring finger protein 169 | ENST00000299563.4 |
| SRGAP1 | SLIT-ROBO Rho GTPase activating protein 1 | ENST00000355086.3 |
| CASK | calcium/calmodulin-dependent serine protein kinase (MAGUK family) | ENST00000421587.2 |
| LCOR | ligand dependent nuclear receptor corepressor | ENST00000371103.3 |
| TAOK1 | TAO kinase 1 | ENST00000261716.3 |
| RAB4A | RAB4A, member RAS oncogene family | ENST00000366690.4 |
| STX1A | syntaxin 1A (brain) | ENST00000395156.3 |
| FAM107B | family with sequence similarity 107, member B | ENST00000378470.1 |
| DAG1 | dystroglycan 1 (dystrophin-associated glycoprotein 1) | ENST00000515359.2 |
| PRKAB1 | protein kinase, AMP-activated, beta 1 non-catalytic subunit | ENST00000229328.5 |
| OTUD4 | OTU domain containing 4 | ENST00000454497.2 |
| TOR1AIP1 | torsin A interacting protein 1 | ENST00000606911.2 |
| KLHL3 | kelch-like family member 3 | ENST00000541417.1 |
| CGGBP1 | CGG triplet repeat binding protein 1 | ENST00000309534.6 |
| YME1L1 | YME1-like 1 ATPase | ENST00000326799.3 |
| MAP1B | microtubule-associated protein 1B | ENST00000296755.7 |
| ATP11B | ATPase, class VI, type 11B | ENST00000323116.5 |
| SLC2A3 | solute carrier family 2 (facilitated glucose transporter), member 3 | ENST00000075120.7 |
| DDIT4 | DNA-damage-inducible transcript 4 | ENST00000307365.3 |
| IFIT5 | interferon-induced protein with tetratricopeptide repeats 5 | ENST00000371795.4 |
| TEAD1 | TEA domain family member 1 (SV40 transcriptional enhancer factor) | ENST00000361905.4 |
| PHF16 | PHD finger protein 16 | ENST00000397189.1 |
| FAT3 | FAT atypical cadherin 3 | ENST00000298047.6 |
| NUMB | numb homolog (Drosophila) | ENST00000554546.1 |
| NAB1 | NGFI-A binding protein 1 (EGR1 binding protein 1) | ENST00000337386.5 |
| SPATA2 | spermatogenesis associated 2 | ENST00000289431.5 |
| ENDOD1 | endonuclease domain containing 1 | ENST00000278505.4 |
| ELK4 | ELK4, ETS-domain protein (SRF accessory protein 1) | ENST00000357992.4 |
| SEC24A | SEC24 family, member A (S. cerevisiae) | ENST00000398844.2 |
| WNK3 | WNK lysine deficient protein kinase 3 | ENST00000375169.3 |
| CACHD1 | cache domain containing 1 | ENST00000371073.2 |
| DMRT2 | doublesex and mab-3 related transcription factor 2 | ENST00000259622.6 |
| GDF6 | growth differentiation factor 6 | ENST00000287020.5 |
| CEP85L | centrosomal protein 85kDa-like | ENST00000368491.3 |
| ASH1L | ash1 (absent, small, or homeotic)-like (Drosophila) | ENST00000368346.3 |
| LPPR4 | Lipid phosphate phosphatase-related protein type 4 | ENST00000370185.3 |
| WIPF1 | WAS/WASL interacting protein family, member 1 | ENST00000392547.2 |
| RPS6KA3 | ribosomal protein S6 kinase, 90kDa, polypeptide 3 | ENST00000379565.3 |
| AP5M1 | adaptor-related protein complex 5, mu 1 subunit | ENST00000261558.3 |
| RAPGEF1 | Rap guanine nucleotide exchange factor (GEF) 1 | ENST00000372189.3 |
| RAB8B | RAB8B, member RAS oncogene family | ENST00000321437.4 |
| NCAM1 | neural cell adhesion molecule 1 | ENST00000316851.7 |
| PLEKHM3 | pleckstrin homology domain containing, family M, member 3 | ENST00000427836.2 |
| MLEC | malectin | ENST00000228506.3 |
| ZNF652 | zinc finger protein 652 | ENST00000362063.2 |
| MAPK1IP1L | mitogen-activated protein kinase 1 interacting protein 1-like | ENST00000395468.4 |
| SNTB2 | syntrophin, beta 2 (dystrophin-associated protein A1, 59kDa, basic component 2) | ENST00000336278.4 |
| BRWD1 | bromodomain and WD repeat domain containing 1 | ENST00000342449.3 |
| MED1 | mediator complex subunit 1 | ENST00000300651.6 |
| KBTBD8 | kelch repeat and BTB (POZ) domain containing 8 | ENST00000295568.4 |
| EP300 | E1A binding protein p300 | ENST00000263253.7 |
| GRID2 | glutamate receptor, ionotropic, delta 2 | ENST00000282020.4 |
| PPP6C | protein phosphatase 6, catalytic subunit | ENST00000373547.4 |
| ERLIN1 | ER lipid raft associated 1 | ENST00000421367.2 |
| ASAP1 | ArfGAP with SH3 domain, ankyrin repeat and PH domain 1 | ENST00000357668.1 |
| KRT80 | keratin 80 | ENST00000313234.5 |
| ZNF365 | zinc finger protein 365 | ENST00000395254.3 |
| FARP1 | FERM, RhoGEF (ARHGEF) and pleckstrin domain protein 1 (chondrocyte-derived) | ENST00000595437.1 |
| ZNF281 | zinc finger protein 281 | ENST00000294740.3 |
| GPC6 | glypican 6 | ENST00000377047.4 |
| HEG1 | heart development protein with EGF-like domains 1 | ENST00000311127.4 |
| LIMK1 | LIM domain kinase 1 | ENST00000418310.1 |
| MGA | MGA, MAX dimerization protein | ENST00000219905.7 |
| GABRB3 | gamma-aminobutyric acid (GABA) A receptor, beta 3 | ENST00000311550.5 |
| ANGEL2 | angel homolog 2 (Drosophila) | ENST00000535388.1 |
| RNF24 | ring finger protein 24 | ENST00000336095.6 |
| SYNJ2BP | synaptojanin 2 binding protein | ENST00000256366.4 |
| UBE2B | ubiquitin-conjugating enzyme E2B | ENST00000265339.2 |
| RGL1 | ral guanine nucleotide dissociation stimulator-like 1 | ENST00000304685.4 |
| NFASC | neurofascin | ENST00000401399.1 |
| FBXW11 | F-box and WD repeat domain containing 11 | ENST00000296933.6 |
| DENND2C | DENN/MADD domain containing 2C | ENST00000393276.3 |
| AKAP2 | A kinase (PRKA) anchor protein 2 | ENST00000374525.1 |
| KSR1 | kinase suppressor of ras 1 | ENST00000398988.3 |
| KDM1B | lysine (K)-specific demethylase 1B | ENST00000388870.2 |
| ELAVL4 | ELAV like neuron-specific RNA binding protein 4 | ENST00000371824.1 |
| ZFX | zinc finger protein, X-linked | ENST00000539115.1 |
| NOTCH1 | notch 1 | ENST00000277541.6 |
| AAK1 | AP2 associated kinase 1 | ENST00000409085.4 |
| CCNJL | cyclin J-like | ENST00000393977.3 |
| PRRG4 | proline rich Gla (G-carboxyglutamic acid) 4 (transmembrane) | ENST00000257836.3 |
| NDNF | neuron-derived neurotrophic factor | ENST00000379692.4 |
| CEP97 | centrosomal protein 97kDa | ENST00000341893.3 |
| CHMP5 | charged multivesicular body protein 5 | ENST00000419016.2 |
| TCF4 | transcription factor 4 | ENST00000354452.3 |
| AFF4 | AF4/FMR2 family, member 4 | ENST00000265343.5 |
| GPRIN3 | GPRIN family member 3 | ENST00000609438.1 |
| BCL2 | B-cell CLL/lymphoma 2 | ENST00000398117.1 |
| FNDC3B | fibronectin type III domain containing 3B | ENST00000336824.4 |
| GRIP1 | glutamate receptor interacting protein 1 | ENST00000398016.3 |
| SLC5A3 | sodium/myo-inositol cotransporter | ENST00000608209.1 |
| PLK2 | polo-like kinase 2 | ENST00000274289.3 |
| CLOCK | clock circadian regulator | ENST00000309964.4 |
| THRB | thyroid hormone receptor, beta | ENST00000396671.2 |
| RFX7 | regulatory factor X, 7 | ENST00000423270.1 |
| AHNAK | AHNAK nucleoprotein | ENST00000378024.4 |
| FAM178A | family with sequence similarity 178, member A | ENST00000238961.4 |
| RAB3B | RAB3B, member RAS oncogene family | ENST00000371655.3 |
| ANGPTL3 | angiopoietin-like 3 | ENST00000371129.3 |
| PALM2-AKAP2 | PALM2-AKAP2 readthrough | ENST00000374530.3 |
| ST8SIA3 | ST8 alpha-N-acetyl-neuraminide alpha-2,8-sialyltransferase 3 | ENST00000324000.3 |
| NACC2 | NACC family member 2, BEN and BTB (POZ) domain containing | ENST00000371753.1 |
| ANO5 | anoctamin 5 | ENST00000324559.8 |
| STYX | serine/threonine/tyrosine interacting protein | ENST00000354586.4 |
| DHX9 | DEAH (Asp-Glu-Ala-His) box helicase 9 | ENST00000367549.3 |
| ROCK2 | Rho-associated, coiled-coil containing protein kinase 2 | ENST00000315872.6 |
| NR2C2 | nuclear receptor subfamily 2, group C, member 2 | ENST00000425241.1 |
| MPRIP | myosin phosphatase Rho interacting protein | ENST00000341712.4 |
| SPRED1 | sprouty-related, EVH1 domain containing 1 | ENST00000299084.4 |
| PLXNC1 | plexin C1 | ENST00000258526.4 |
| CDC27 | cell division cycle 27 | ENST00000066544.3 |
| AUTS2 | autism susceptibility candidate 2 | ENST00000342771.4 |
| NUP153 | nucleoporin 153kDa | ENST00000262077.2 |
| SLC2A14 | solute carrier family 2 (facilitated glucose transporter), member 14 | ENST00000340749.5 |
| FBXL16 | F-box and leucine-rich repeat protein 16 | ENST00000397621.1 |
| RIMS3 | regulating synaptic membrane exocytosis 3 | ENST00000372684.3 |
| ETF1 | eukaryotic translation termination factor 1 | ENST00000499810.2 |
| PRPF38B | pre-mRNA processing factor 38B | ENST00000370025.4 |
| ANO6 | anoctamin 6 | ENST00000320560.8 |
| PITPNM3 | PITPNM family member 3 | ENST00000421306.3 |
| CAND1 | cullin-associated and neddylation-dissociated 1 | ENST00000545606.1 |
| ARHGDIA | Rho GDP dissociation inhibitor (GDI) alpha | ENST00000269321.7 |
| FRMD4A | FERM domain containing 4A | ENST00000358621.4 |
| KRR1 | KRR1, small subunit (SSU) processome component, homolog (yeast) | ENST00000229214.4 |
| MAFG | v-maf avian musculoaponeurotic fibrosarcoma oncogene homolog G | ENST00000357736.4 |
| ERN1 | endoplasmic reticulum to nucleus signaling 1 | ENST00000433197.3 |
| CDH7 | cadherin 7, type 2 | ENST00000397968.2 |
| MYO9A | myosin IXA | ENST00000564571.1 |
| SLC30A10 | solute carrier family 30, member 10 | ENST00000366926.3 |
| KIF26B | kinesin family member 26B | ENST00000366518.4 |
| PMPCB | peptidase (mitochondrial processing) beta | ENST00000249269.4 |
| MKL2 | MKL/myocardin-like 2 | ENST00000318282.5 |
| ARID5B | AT rich interactive domain 5B (MRF1-like) | ENST00000279873.7 |
| SEPHS1 | selenophosphate synthetase 1 | ENST00000545675.1 |
| AGO2 | argonaute RISC catalytic component 2 | ENST00000220592.5 |
| ZBTB8B | zinc finger and BTB domain containing 8B | ENST00000609129.1 |
| CELF1 | CUGBP, Elav-like family member 1 | ENST00000395290.2 |
| C1orf131 | chromosome 1 open reading frame 131 | ENST00000318906.2 |
| ATXN1L | ataxin 1-like | ENST00000427980.2 |
| LATS2 | large tumor suppressor kinase 2 | ENST00000382592.4 |
| PARD3B | par-3 family cell polarity regulator beta | ENST00000406610.2 |
| FAM63B | family with sequence similarity 63, member B | ENST00000559228.1 |
| SORT1 | sortilin 1 | ENST00000256637.6 |
| PTAR1 | protein prenyltransferase alpha subunit repeat containing 1 | ENST00000377200.5 |
| ERGIC2 | ERGIC and golgi 2 | ENST00000360150.4 |
| CTBP2 | C-terminal binding protein 2 | ENST00000337195.5 |
| PLXNA2 | plexin A2 | ENST00000367033.3 |
| GDNF | glial cell derived neurotrophic factor | ENST00000326524.2 |
| PLXNA4 | plexin A4 | ENST00000321063.4 |
| GNA13 | guanine nucleotide binding protein (G protein), alpha 13 | ENST00000439174.2 |
| ATF7IP | activating transcription factor 7 interacting protein | ENST00000261168.4 |
| KIAA0430 | KIAA0430 | ENST00000396368.3 |
| A1CF | APOBEC1 complementation factor | ENST00000374001.2 |
| SLC4A4 | solute carrier family 4 (sodium bicarbonate cotransporter), member 4 | ENST00000340595.3 |
| MCC | mutated in colorectal cancers | ENST00000302475.4 |
| MAF | v-maf avian musculoaponeurotic fibrosarcoma oncogene homolog | ENST00000393350.1 |
| TAF4 | TAF4 RNA polymerase II, TATA box binding protein (TBP)-associated factor, 135kDa | ENST00000252996.4 |
| BRWD3 | bromodomain and WD repeat domain containing 3 | ENST00000373275.4 |
| WDFY2 | WD repeat and FYVE domain containing 2 | ENST00000298125.5 |
| CDK12 | cyclin-dependent kinase 12 | ENST00000447079.4 |
| ZCCHC14 | zinc finger, CCHC domain containing 14 | ENST00000268616.4 |
| CBX5 | chromobox homolog 5 | ENST00000209875.4 |
| UBE2R2 | ubiquitin-conjugating enzyme E2R 2 | ENST00000263228.3 |
| ZNF516 | zinc finger protein 516 | ENST00000443185.2 |
| ABCA1 | ATP-binding cassette, sub-family A (ABC1), member 1 | ENST00000374736.3 |
| ERBB4 | v-erb-b2 avian erythroblastic leukemia viral oncogene homolog 4 | ENST00000342788.4 |
| ARHGEF1 | Rho guanine nucleotide exchange factor (GEF) 1 | ENST00000347545.4 |
| CHSY3 | chondroitin sulfate synthase 3 | ENST00000305031.4 |
| EMB | embigin | ENST00000303221.5 |
| ZNF395 | zinc finger protein 395 | ENST00000344423.5 |
| LRIG1 | leucine-rich repeats and immunoglobulin-like domains 1 | ENST00000273261.3 |
| HIVEP3 | human immunodeficiency virus type I enhancer binding protein 3 | ENST00000372583.1 |
| SH3PXD2A | SH3 and PX domains 2A | ENST00000369774.4 |
| NCOR2 | nuclear receptor corepressor 2 | ENST00000405201.1 |
| ATAD2B | ATPase family, AAA domain containing 2B | ENST00000238789.5 |
| PAQR5 | progestin and adipoQ receptor family member V | ENST00000395407.2 |
| FAM179B | family with sequence similarity 179, member B | ENST00000382233.2 |
| BICD2 | bicaudal D homolog 2 (Drosophila) | ENST00000356884.6 |
| MEX3D | mex-3 RNA binding family member D | ENST00000402693.4 |
| PCDH19 | protocadherin 19 | ENST00000420881.2 |
| STXBP6 | syntaxin binding protein 6 (amisyn) | ENST00000396700.1 |
| TBL1XR1 | transducin (beta)-like 1 X-linked receptor 1 | ENST00000430069.1 |
| HIPK2 | homeodomain interacting protein kinase 2 | ENST00000406875.3 |
| GPR63 | G protein-coupled receptor 63 | ENST00000229955.3 |
| GIGYF1 | GRB10 interacting GYF protein 1 | ENST00000275732.5 |
| PVRL1 | poliovirus receptor-related 1 (herpesvirus entry mediator C) | ENST00000264025.3 |
| USH1G | Usher syndrome 1G (autosomal recessive) | ENST00000319642.1 |
| ABCC9 | ATP-binding cassette, sub-family C (CFTR/MRP), member 9 | ENST00000261200.4 |
| SLC6A17 | solute carrier family 6 (neutral amino acid transporter), member 17 | ENST00000331565.4 |
| AMBRA1 | autophagy/beclin-1 regulator 1 | ENST00000314845.3 |
| PPP2R5C | protein phosphatase 2, regulatory subunit B', gamma | ENST00000422945.2 |
| JAKMIP3 | Janus kinase and microtubule interacting protein 3 | ENST00000298622.4 |
| REL | v-rel avian reticuloendotheliosis viral oncogene homolog | ENST00000295025.8 |
| GREB1L | growth regulation by estrogen in breast cancer-like | ENST00000580732.2 |
| CUL5 | cullin 5 | ENST00000393094.2 |
| USH2A | Usher syndrome 2A (autosomal recessive, mild) | ENST00000307340.3 |
| DFFA | DNA fragmentation factor, 45kDa, alpha polypeptide | ENST00000377038.3 |
| GSE1 | Gse1 coiled-coil protein | ENST00000253458.7 |
| IPO8 | importin 8 | ENST00000256079.4 |
| GOSR2 | golgi SNAP receptor complex member 2 | ENST00000576910.2 |
| ZC3H4 | zinc finger CCCH-type containing 4 | ENST00000253048.5 |
| EED | embryonic ectoderm development | ENST00000327320.4 |
| FAM46C | family with sequence similarity 46, member C | ENST00000369448.3 |
| ARHGAP26 | Rho GTPase activating protein 26 | ENST00000378004.3 |
| SUZ12 | SUZ12 polycomb repressive complex 2 subunit | ENST00000322652.5 |
| DCUN1D3 | DCN1, defective in cullin neddylation 1, domain containing 3 | ENST00000324344.4 |
| SIAH1 | siah E3 ubiquitin protein ligase 1 | ENST00000380006.2 |
| ONECUT2 | one cut homeobox 2 | ENST00000491143.2 |
| NTPCR | nucleoside-triphosphatase, cancer-related | ENST00000366628.5 |
| PPP1R9A | protein phosphatase 1, regulatory subunit 9A | ENST00000289495.5 |
| FRMD5 | FERM domain containing 5 | ENST00000484674.1 |
| ACVR2B | activin A receptor, type IIB | ENST00000352511.4 |
| ELL | elongation factor RNA polymerase II | ENST00000262809.4 |
| TRIM71 | tripartite motif containing 71, E3 ubiquitin protein ligase | ENST00000383763.5 |
| ANKRD52 | ankyrin repeat domain 52 | ENST00000267116.7 |
| TPCN1 | two pore segment channel 1 | ENST00000335509.6 |
| CEP350 | centrosomal protein 350kDa | ENST00000367607.3 |
| PSME4 | proteasome (prosome, macropain) activator subunit 4 | ENST00000404125.1 |
| PAK3 | p21 protein (Cdc42/Rac)-activated kinase 3 | ENST00000372007.5 |
| UHRF1BP1 | UHRF1 binding protein 1 | ENST00000192788.5 |
| JMY | junction mediating and regulatory protein, p53 cofactor | ENST00000396137.4 |
| SYNCRIP | synaptotagmin binding, cytoplasmic RNA interacting protein | ENST00000355238.6 |
| SMURF1 | SMAD specific E3 ubiquitin protein ligase 1 | ENST00000361368.2 |
| UBE2J1 | ubiquitin-conjugating enzyme E2, J1 | ENST00000435041.2 |
| SENP5 | SUMO1/sentrin specific peptidase 5 | ENST00000323460.5 |
| USP31 | ubiquitin specific peptidase 31 | ENST00000219689.7 |
| RNF11 | ring finger protein 11 | ENST00000242719.3 |
| PDE7B | phosphodiesterase 7B | ENST00000308191.6 |
| NUFIP2 | nuclear fragile X mental retardation protein interacting protein 2 | ENST00000225388.4 |
| ADCY9 | adenylate cyclase 9 | ENST00000294016.3 |
| ZNF148 | zinc finger protein 148 | ENST00000360647.4 |
| KCTD16 | potassium channel tetramerization domain containing 16 | ENST00000507359.3 |
| MOCS1 | molybdenum cofactor synthesis 1 | ENST00000373186.4 |
| DYRK2 | dual-specificity tyrosine-(Y)-phosphorylation regulated kinase 2 | ENST00000344096.3 |
| RBBP8 | retinoblastoma binding protein 8 | ENST00000399722.2 |
| JAG2 | jagged 2 | ENST00000331782.3 |
| TRPM3 | transient receptor potential cation channel, subfamily M, member 3 | ENST00000377110.3 |
| AVL9 | AVL9 homolog (S. cerevisiase) | ENST00000318709.4 |
| CNNM2 | cyclin M2 | ENST00000369878.4 |
| TMEM136 | transmembrane protein 136 | ENST00000529187.1 |
| ST3GAL2 | ST3 beta-galactoside alpha-2,3-sialyltransferase 2 | ENST00000342907.2 |
| NDST1 | N-deacetylase/N-sulfotransferase (heparan glucosaminyl) 1 | ENST00000261797.6 |
| NRP2 | neuropilin 2 | ENST00000360409.3 |
| SCN2A | sodium channel, voltage-gated, type II, alpha subunit | ENST00000375437.2 |
| KDM3B | lysine (K)-specific demethylase 3B | ENST00000314358.5 |
| KCNQ4 | potassium voltage-gated channel, KQT-like subfamily, member 4 | ENST00000347132.5 |
| SMAD7 | SMAD family member 7 | ENST00000262158.2 |
| MPP5 | membrane protein, palmitoylated 5 (MAGUK p55 subfamily member 5) | ENST00000261681.4 |
| SCN5A | sodium channel, voltage-gated, type V, alpha subunit | ENST00000414099.2 |
| TLN1 | talin 1 | ENST00000314888.9 |
| IL6ST | interleukin 6 signal transducer (gp130, oncostatin M receptor) | ENST00000381287.4 |
| PHF12 | PHD finger protein 12 | ENST00000577226.1 |
| IQSEC2 | IQ motif and Sec7 domain 2 | ENST00000375365.2 |
| DCAF7 | DDB1 and CUL4 associated factor 7 | ENST00000310827.4 |
| SOCS6 | suppressor of cytokine signaling 6 | ENST00000397942.3 |
| CHD9 | chromodomain helicase DNA binding protein 9 | ENST00000566029.1 |
| ERC1 | ELKS/RAB6-interacting/CAST family member 1 | ENST00000355446.5 |
| SLC6A6 | solute carrier family 6 (neurotransmitter transporter), member 6 | ENST00000454876.2 |
| PROX1 | prospero homeobox 1 | ENST00000366958.4 |
| TUBB | tubulin, beta class I | ENST00000327892.8 |
| UBXN7 | UBX domain protein 7 | ENST00000296328.4 |
| USP47 | ubiquitin specific peptidase 47 | ENST00000339865.5 |
| SP1 | Sp1 transcription factor | ENST00000426431.2 |
| DDX3X | DEAD (Asp-Glu-Ala-Asp) box helicase 3, X-linked | ENST00000399959.2 |
| GAD2 | glutamate decarboxylase 2 (pancreatic islets and brain, 65kDa) | ENST00000376261.3 |
| RPRD1A | regulation of nuclear pre-mRNA domain containing 1A | ENST00000399022.4 |
| SWAP70 | SWAP switching B-cell complex 70kDa subunit | ENST00000318950.6 |
| IRS1 | insulin receptor substrate 1 | ENST00000305123.5 |
| SETD9 | SET domain containing 9 | ENST00000285947.2 |
| FBXW2 | F-box and WD repeat domain containing 2 | ENST00000608872.1 |
| MAP4K4 | mitogen-activated protein kinase kinase kinase kinase 4 | ENST00000413150.2 |
| USP46 | ubiquitin specific peptidase 46 | ENST00000441222.3 |
| C4orf32 | chromosome 4 open reading frame 32 | ENST00000309733.5 |
| FREM2 | FRAS1 related extracellular matrix protein 2 | ENST00000280481.7 |
| POU6F1 | POU class 6 homeobox 1 | ENST00000389243.4 |
| COL9A3 | collagen, type IX, alpha 3 | ENST00000343916.3 |
| LPIN2 | lipin 2 | ENST00000261596.4 |
| GATAD2B | GATA zinc finger domain containing 2B | ENST00000368655.4 |
| LYPLA2 | lysophospholipase II | ENST00000374505.2 |
| MYPN | myopalladin | ENST00000358913.5 |
| CDK19 | cyclin-dependent kinase 19 | ENST00000368911.3 |
| CELF2 | CUGBP, Elav-like family member 2 | ENST00000379261.4 |
| TJP1 | tight junction protein 1 | ENST00000346128.6 |
| AGAP1 | ArfGAP with GTPase domain, ankyrin repeat and PH domain 1 | ENST00000304032.8 |
| SUV420H1 | suppressor of variegation 4-20 homolog 1 (Drosophila) | ENST00000304363.4 |
| TMEM14A | transmembrane protein 14A | ENST00000211314.4 |
| HDAC4 | histone deacetylase 4 | ENST00000345617.3 |
| AFF1 | AF4/FMR2 family, member 1 | ENST00000395146.4 |
| KMT2C | lysine (K)-specific methyltransferase 2C | ENST00000262189.6 |
| SLC16A10 | solute carrier family 16 (aromatic amino acid transporter), member 10 | ENST00000368850.3 |
| MED6 | mediator complex subunit 6 | ENST00000440435.2 |
| NEDD4L | neural precursor cell expressed, developmentally down-regulated 4-like, E3 ubiquitin protein ligase | ENST00000256832.7 |
| RAB7A | RAB7A, member RAS oncogene family | ENST00000265062.3 |
| ACVR2A | activin A receptor, type IIA | ENST00000241416.7 |
| SETD7 | SET domain containing (lysine methyltransferase) 7 | ENST00000274031.3 |
| FOXP1 | forkhead box P1 | ENST00000318789.4 |
| RBFOX2 | RNA binding protein, fox-1 homolog (C. elegans) 2 | ENST00000449924.2 |
| NEO1 | neogenin 1 | ENST00000261908.6 |
| TIGD2 | tigger transposable element derived 2 | ENST00000317005.2 |
| SESN3 | sestrin 3 | ENST00000536441.1 |
| MBNL1 | muscleblind-like splicing regulator 1 | ENST00000357472.3 |
| CAMK2D | calcium/calmodulin-dependent protein kinase II delta | ENST00000296402.5 |
| PTDSS1 | phosphatidylserine synthase 1 | ENST00000517309.1 |
| KCNK12 | potassium channel, subfamily K, member 12 | ENST00000327876.4 |
| CASKIN1 | CASK interacting protein 1 | ENST00000343516.6 |
| ZFHX4 | zinc finger homeobox 4 | ENST00000521891.2 |
| KLF13 | Kruppel-like factor 13 | ENST00000307145.3 |
| PAPLN | papilin, proteoglycan-like sulfated glycoprotein | ENST00000381166.3 |
| FAM126A | family with sequence similarity 126, member A | ENST00000409923.1 |
| SAMD8 | sterile alpha motif domain containing 8 | ENST00000372687.4 |
| SIN3A | SIN3 transcription regulator family member A | ENST00000394947.3 |
| BBX | bobby sox homolog (Drosophila) | ENST00000415149.2 |
| MXI1 | MAX interactor 1, dimerization protein | ENST00000332674.5 |
| BCL9 | B-cell CLL/lymphoma 9 | ENST00000234739.3 |
| ZC3H12C | zinc finger CCCH-type containing 12C | ENST00000278590.3 |
| GM2A | GM2 ganglioside activator | ENST00000357164.3 |
| DENND5A | DENN/MADD domain containing 5A | ENST00000530044.1 |
| APLP2 | amyloid beta (A4) precursor-like protein 2 | ENST00000263574.5 |
| NFIC | nuclear factor I/C (CCAAT-binding transcription factor) | ENST00000346156.5 |
| TMPPE | transmembrane protein with metallophosphoesterase domain | ENST00000416695.2 |
| CBX8 | chromobox homolog 8 | ENST00000269385.4 |
| SOX6 | SRY (sex determining region Y)-box 6 | ENST00000316399.6 |
| SIK2 | salt-inducible kinase 2 | ENST00000304987.3 |
| HECW2 | HECT, C2 and WW domain containing E3 ubiquitin protein ligase 2 | ENST00000260983.3 |
| ROBO2 | roundabout, axon guidance receptor, homolog 2 (Drosophila) | ENST00000461745.1 |
| COPS2 | COP9 signalosome subunit 2 | ENST00000388901.5 |
| TRPV3 | transient receptor potential cation channel, subfamily V, member 3 | ENST00000301365.4 |
| PISD | phosphatidylserine decarboxylase | ENST00000397500.1 |
| TBCEL | tubulin folding cofactor E-like | ENST00000422003.2 |
| PAQR3 | progestin and adipoQ receptor family member III | ENST00000512733.1 |
| PRKCA | protein kinase C, alpha | ENST00000413366.3 |
| DNAJC18 | DnaJ (Hsp40) homolog, subfamily C, member 18 | ENST00000302060.5 |
| CDK16 | cyclin-dependent kinase 16 | ENST00000457458.2 |
| PHB2 | prohibitin 2 | ENST00000546111.1 |
| C2orf68 | chromosome 2 open reading frame 68 | ENST00000306336.5 |
| KCMF1 | potassium channel modulatory factor 1 | ENST00000409785.4 |
| FGF12 | fibroblast growth factor 12 | ENST00000445105.2 |
| SHROOM2 | shroom family member 2 | ENST00000380913.3 |
| CREB1 | cAMP responsive element binding protein 1 | ENST00000432329.2 |
| HIC2 | hypermethylated in cancer 2 | ENST00000407464.2 |
| TRMT10B | tRNA methyltransferase 10 homolog B (S. cerevisiae) | ENST00000297994.3 |
| YWHAB | tyrosine 3-monooxygenase/tryptophan 5-monooxygenase activation protein, beta polypeptide | ENST00000353703.4 |
| SAR1B | SAR1 homolog B (S. cerevisiae) | ENST00000402673.2 |
| ARL3 | ADP-ribosylation factor-like 3 | ENST00000260746.5 |
| MLLT3 | myeloid/lymphoid or mixed-lineage leukemia (trithorax homolog, Drosophila); translocated to, 3 | ENST00000380338.4 |
| SPRYD4 | SPRY domain containing 4 | ENST00000338146.5 |
| EIF4E | eukaryotic translation initiation factor 4E | ENST00000450253.2 |
| SLC31A1 | solute carrier family 31 (copper transporter), member 1 | ENST00000374212.4 |
| ATP6V0A2 | ATPase, H+ transporting, lysosomal V0 subunit a2 | ENST00000330342.3 |
| NIPBL | Nipped-B homolog (Drosophila) | ENST00000448238.2 |
| PLEKHA6 | pleckstrin homology domain containing, family A member 6 | ENST00000272203.3 |
| GRK5 | G protein-coupled receptor kinase 5 | ENST00000392870.2 |
| MRPS25 | mitochondrial ribosomal protein S25 | ENST00000253686.2 |
| DMD | dystrophin | ENST00000378677.2 |
| SEC31B | SEC31 homolog B (S. cerevisiae) | ENST00000370345.3 |
| WHAMM | WAS protein homolog associated with actin, golgi membranes and microtubules | ENST00000286760.4 |
| GLG1 | golgi glycoprotein 1 | ENST00000422840.2 |
| LMO4 | LIM domain only 4 | ENST00000370544.5 |
| LRP1 | low density lipoprotein receptor-related protein 1 | ENST00000243077.3 |
| SLC35E2 | solute carrier family 35, member E2 | ENST00000246421.4 |
| ZBTB20 | zinc finger and BTB domain containing 20 | ENST00000462705.1 |
| PTCHD3 | patched domain containing 3 | ENST00000438700.3 |
| FGF10 | fibroblast growth factor 10 | ENST00000264664.4 |
| PRMT7 | protein arginine methyltransferase 7 | ENST00000339507.5 |
| MEF2D | myocyte enhancer factor 2D | ENST00000464356.2 |
| GRIN2A | glutamate receptor, ionotropic, N-methyl D-aspartate 2A | ENST00000562109.1 |
| CADM1 | cell adhesion molecule 1 | ENST00000452722.3 |
| JAKMIP2 | janus kinase and microtubule interacting protein 2 | ENST00000507386.1 |
| SERPINC1 | serpin peptidase inhibitor, clade C (antithrombin), member 1 | ENST00000367698.3 |
| **DIANA microT prediction** |  |  |
| Gene symbol | Gene ID | Transcript ID |
| ZEB2 | ENSG00000169554 | ENST00000558170 |
| ZEB1 | ENSG00000148516 | ENST00000437844 |
| SOX5 | ENSG00000134532 | ENST00000546136 |
| FOXG1 | ENSG00000176165 | ENST00000313071 |
| GPM6A | ENSG00000150625 | ENST00000280187 |
| KIAA1432 | ENSG00000107036 | ENST00000414202 |
| PSIP1 | ENSG00000164985 | ENST00000380733 |
| HIPK3 | ENSG00000110422 | ENST00000303296 |
| SYNJ1 | ENSG00000159082 | ENST00000357345 |
| SESN1 | ENSG00000080546 | ENST00000436639 |
| RND3 | ENSG00000115963 | ENST00000263895 |
| MARCKS | ENSG00000155130 | ENST00000368635 |
| RPS6KB1 | ENSG00000108443 | ENST00000225577 |
| PTPN21 | ENSG00000070778 | ENST00000536337 |
| RUSC2 | ENSG00000198853 | ENST00000455600 |
| DTNA | ENSG00000134769 | ENST00000283365 |
| MAP2 | ENSG00000078018 | ENST00000392194 |
| MMD | ENSG00000108960 | ENST00000262065 |
| OXR1 | ENSG00000164830 | ENST00000312046 |
| ZFPM2 | ENSG00000169946 | ENST00000407775 |
| SORT1 | ENSG00000134243 | ENST00000256637 |
| ZNF217 | ENSG00000171940 | ENST00000371471 |
| RECK | ENSG00000122707 | ENST00000377966 |
| KDR | ENSG00000128052 | ENST00000263923 |
| KIAA0087 | ENSG00000122548 | ENST00000242109 |
| USP6NL | ENSG00000148429 | ENST00000609104 |
| WASF3 | ENSG00000132970 | ENST00000335327 |
| LPPR4 | ENSG00000117600 | ENST00000370185 |
| TSC22D2 | ENSG00000196428 | ENST00000361875 |
| WIPF1 | ENSG00000115935 | ENST00000392547 |
| DUSP1 | ENSG00000120129 | ENST00000239223 |
| ELL2 | ENSG00000118985 | ENST00000237853 |
| LRP1B | ENSG00000168702 | ENST00000389484 |
| FIGN | ENSG00000182263 | ENST00000333129 |
| HMBOX1 | ENSG00000147421 | ENST00000397358 |
| CFL2 | ENSG00000165410 | ENST00000341223 |
| VASH2 | ENSG00000143494 | ENST00000366968 |
| SEC23A | ENSG00000100934 | ENST00000537403 |
| KDM7A | ENSG00000006459 | ENST00000472616 |
| WAPAL | ENSG00000062650 | ENST00000298767 |
| EFNB2 | ENSG00000125266 | ENST00000245323 |
| CCNJ | ENSG00000107443 | ENST00000265992 |
| CRKL | ENSG00000099942 | ENST00000354336 |
| FBXW7 | ENSG00000109670 | ENST00000281708 |
| AMFR | ENSG00000159461 | ENST00000290649 |
| UBE2W | ENSG00000104343 | ENST00000517608 |
| TRIM33 | ENSG00000197323 | ENST00000358465 |
| CLIC4 | ENSG00000169504 | ENST00000374379 |
| MAPRE1 | ENSG00000101367 | ENST00000375571 |
| FAT3 | ENSG00000165323 | ENST00000298047 |
| TSC22D1 | ENSG00000102804 | ENST00000458659 |
| BPY2 | ENSG00000183753 | ENST00000331070 |
| RTF1 | ENSG00000137815 | ENST00000389629 |
| BPY2B | ENSG00000183795 | ENST00000382392 |
| BPY2C | ENSG00000185894 | ENST00000382287 |
| PPFIA1 | ENSG00000131626 | ENST00000526262 |
| RAP2C | ENSG00000123728 | ENST00000342983 |
| FBXO33 | ENSG00000165355 | ENST00000298097 |
| A1CF | ENSG00000148584 | ENST00000374001 |
| MTSS1L | ENSG00000132613 | ENST00000338779 |
| EVI5 | ENSG00000067208 | ENST00000370331 |
| QKI | ENSG00000112531 | ENST00000361752 |
| FOXF1 | ENSG00000103241 | ENST00000262426 |
| PCSK2 | ENSG00000125851 | ENST00000262545 |
| MARCH8 | ENSG00000165406 | ENST00000453424 |
| YPEL2 | ENSG00000175155 | ENST00000312655 |
| RAP1B | ENSG00000127314 | ENST00000250559 |
| GIT2 | ENSG00000139436 | ENST00000354574 |
| RBFOX3 | ENSG00000167281 | ENST00000583458 |
| EFNA1 | ENSG00000169242 | ENST00000368407 |
| KMT2C | ENSG00000055609 | ENST00000558084 |
| MATR3 | ENSG00000015479 | ENST00000394800 |
| CLIP1 | ENSG00000130779 | ENST00000361654 |
| PDIK1L | ENSG00000175087 | ENST00000374271 |
| TIMP2 | ENSG00000035862 | ENST00000262768 |
| DNAJB5 | ENSG00000137094 | ENST00000538278 |
| DIXDC1 | ENSG00000150764 | ENST00000315253 |
| SLC38A2 | ENSG00000134294 | ENST00000549258 |
| NOG | ENSG00000183691 | ENST00000332822 |
| STYX | ENSG00000198252 | ENST00000354586 |
| JAZF1 | ENSG00000153814 | ENST00000427814 |
| USP27X | ENSG00000242013 | ENST00000508866 |
| MPRIP | ENSG00000133030 | ENST00000395811 |
| MAP4K3 | ENSG00000011566 | ENST00000263881 |
| GNAQ | ENSG00000156052 | ENST00000286548 |
| CASR | ENSG00000036828 | ENST00000498619 |
| CDK17 | ENSG00000059758 | ENST00000261211 |
| PALM2 | ENSG00000243444 | ENST00000448454 |
| CCNYL1 | ENSG00000163249 | ENST00000295414 |
| CDH20 | ENSG00000101542 | ENST00000262717 |
| SLIT2 | ENSG00000145147 | ENST00000504154 |
| SFXN1 | ENSG00000164466 | ENST00000321442 |
| PVRL4 | ENSG00000143217 | ENST00000368012 |
| C16orf52 | ENSG00000185716 | ENST00000542527 |
| GJC1 | ENSG00000182963 | ENST00000426548 |
| AC068987.1 | ENSG00000260415 | ENST00000599343 |
| COL4A3BP | ENSG00000113163 | ENST00000380494 |
| CDC73 | ENSG00000134371 | ENST00000367435 |
| CEP350 | ENSG00000135837 | ENST00000367607 |
| GTF2E1 | ENSG00000153767 | ENST00000283875 |
| SESN3 | ENSG00000149212 | ENST00000536441 |
| TLN2 | ENSG00000171914 | ENST00000306829 |
| PRTG | ENSG00000166450 | ENST00000389286 |
| LOX | ENSG00000113083 | ENST00000231004 |
| DACH1 | ENSG00000165659 | ENST00000305425 |
| YWHAG | ENSG00000170027 | ENST00000307630 |
| HS3ST1 | ENSG00000002587 | ENST00000002596 |
| RAPGEF2 | ENSG00000109756 | ENST00000264431 |
| FAM63B | ENSG00000128923 | ENST00000559228 |
| ADAMTS3 | ENSG00000156140 | ENST00000286657 |
| SLC6A1 | ENSG00000157103 | ENST00000287766 |
| PHTF2 | ENSG00000006576 | ENST00000416283 |
| TMX4 | ENSG00000125827 | ENST00000246024 |
| PMAIP1 | ENSG00000141682 | ENST00000316660 |
| CUX1 | ENSG00000257923 | ENST00000360264 |
| KLHL14 | ENSG00000197705 | ENST00000359358 |
| TBX18 | ENSG00000112837 | ENST00000369663 |
| CNEP1R1 | ENSG00000205423 | ENST00000568890 |
| RASA2 | ENSG00000155903 | ENST00000286364 |
| PLEKHA3 | ENSG00000116095 | ENST00000234453 |
| JUN | ENSG00000177606 | ENST00000371222 |
| GOLIM4 | ENSG00000173905 | ENST00000470487 |
| WIF1 | ENSG00000156076 | ENST00000286574 |
| DENND5B | ENSG00000170456 | ENST00000306833 |
| ARL5A | ENSG00000162980 | ENST00000295087 |
| RELN | ENSG00000189056 | ENST00000343529 |
| XKR8 | ENSG00000158156 | ENST00000373884 |
| SYDE1 | ENSG00000105137 | ENST00000342784 |
| PTPN12 | ENSG00000127947 | ENST00000248594 |
| NAB1 | ENSG00000138386 | ENST00000337386 |
| PPP4R2 | ENSG00000163605 | ENST00000356692 |
| MTF2 | ENSG00000143033 | ENST00000370298 |
| OSBPL11 | ENSG00000144909 | ENST00000296220 |
| NR5A2 | ENSG00000116833 | ENST00000367362 |
| ZFAND6 | ENSG00000086666 | ENST00000261749 |
| GAS2L3 | ENSG00000139354 | ENST00000552854 |
| PPP1R18 | ENSG00000146112 | ENST00000274853 |
| ESRRG | ENSG00000196482 | ENST00000361525 |
| SLC14A1 | ENSG00000141469 | ENST00000321925 |
| ZBTB7C | ENSG00000184828 | ENST00000586438 |
| MED13 | ENSG00000108510 | ENST00000397786 |
| SUPT20H | ENSG00000102710 | ENST00000490716 |
| SLC6A11 | ENSG00000132164 | ENST00000254488 |
| NRBP1 | ENSG00000115216 | ENST00000233557 |
| CDYL | ENSG00000153046 | ENST00000343762 |
| GABPA | ENSG00000154727 | ENST00000400075 |
| PAIP2 | ENSG00000120727 | ENST00000394795 |
| SLC35B4 | ENSG00000205060 | ENST00000378509 |
| ZFX | ENSG00000005889 | ENST00000379188 |
| DPY19L1 | ENSG00000173852 | ENST00000310974 |
| PCDH19 | ENSG00000165194 | ENST00000373034 |
| ZNF536 | ENSG00000198597 | ENST00000592773 |
| NFYA | ENSG00000001167 | ENST00000341376 |
| CCDC82 | ENSG00000149231 | ENST00000278520 |
| PHACTR3 | ENSG00000087495 | ENST00000361300 |
| FLT1 | ENSG00000102755 | ENST00000282397 |
| RANBP9 | ENSG00000010017 | ENST00000011619 |
| NUP153 | ENSG00000124789 | ENST00000262077 |
| EGLN1 | ENSG00000135766 | ENST00000366641 |
| RASSF8 | ENSG00000123094 | ENST00000541490 |
| LHFP | ENSG00000183722 | ENST00000379589 |
| ZNF532 | ENSG00000074657 | ENST00000336078 |
| MSN | ENSG00000147065 | ENST00000360270 |
| LPPR1 | ENSG00000148123 | ENST00000374874 |
| CEP41 | ENSG00000106477 | ENST00000223208 |
| PHF21B | ENSG00000056487 | ENST00000313237 |
| SLC15A5 | ENSG00000188991 | ENST00000344941 |
| TMEM136 | ENSG00000181264 | ENST00000529187 |
| KIAA1462 | ENSG00000165757 | ENST00000375377 |
| SUSD5 | ENSG00000173705 | ENST00000309558 |
| ADIPOR2 | ENSG00000006831 | ENST00000357103 |
| BAP1 | ENSG00000163930 | ENST00000460680 |
| MAP3K1 | ENSG00000095015 | ENST00000399503 |
| RBFOX2 | ENSG00000100320 | ENST00000405409 |
| C21orf91 | ENSG00000154642 | ENST00000284881 |
| CNKSR3 | ENSG00000153721 | ENST00000607772 |
| CADM2 | ENSG00000175161 | ENST00000383699 |
| IPO7 | ENSG00000205339 | ENST00000379719 |
| INTS8 | ENSG00000164941 | ENST00000523206 |
| KANK2 | ENSG00000197256 | ENST00000586659 |
| DNAJC3 | ENSG00000102580 | ENST00000602402 |
| ASH1L | ENSG00000116539 | ENST00000368346 |
| GTPBP10 | ENSG00000105793 | ENST00000380058 |
| AFF3 | ENSG00000144218 | ENST00000409236 |
| PKP4 | ENSG00000144283 | ENST00000426248 |
| THAP1 | ENSG00000131931 | ENST00000345117 |
| CHN2 | ENSG00000106069 | ENST00000222792 |
| POLK | ENSG00000122008 | ENST00000241436 |
| CDYL2 | ENSG00000166446 | ENST00000570137 |
| DGKH | ENSG00000102780 | ENST00000261491 |
| MSL2 | ENSG00000174579 | ENST00000309993 |
| CASK | ENSG00000147044 | ENST00000318588 |
| ZNF292 | ENSG00000188994 | ENST00000369577 |
| TBC1D12 | ENSG00000108239 | ENST00000225235 |
| TNRC6A | ENSG00000090905 | ENST00000491718 |
| PPP2R5E | ENSG00000154001 | ENST00000337537 |
| FAM8A1 | ENSG00000137414 | ENST00000259963 |
| HNRNPD | ENSG00000138668 | ENST00000313899 |
| FLI1 | ENSG00000151702 | ENST00000429175 |
| PI4KB | ENSG00000143393 | ENST00000368874 |
| SECISBP2L | ENSG00000138593 | ENST00000559471 |
| NFIA | ENSG00000162599 | ENST00000403491 |
| ELAVL2 | ENSG00000107105 | ENST00000380110 |
| MCFD2 | ENSG00000180398 | ENST00000444761 |
| RRP15 | ENSG00000067533 | ENST00000366932 |
| ARID4A | ENSG00000032219 | ENST00000431317 |
| GNAI3 | ENSG00000065135 | ENST00000369851 |
| CECR2 | ENSG00000099954 | ENST00000400573 |
| C16orf72 | ENSG00000182831 | ENST00000327827 |
| ARIH1 | ENSG00000166233 | ENST00000379887 |
| N4BP2 | ENSG00000078177 | ENST00000511480 |
| SUZ12 | ENSG00000178691 | ENST00000322652 |
| YOD1 | ENSG00000180667 | ENST00000367084 |
| CACNB2 | ENSG00000165995 | ENST00000396576 |
| NANOS1 | ENSG00000188613 | ENST00000425699 |
| SCRT2 | ENSG00000215397 | ENST00000246104 |
| ACOT11 | ENSG00000162390 | ENST00000371316 |
| KIAA0101 | ENSG00000166803 | ENST00000300035 |
| ACTR6 | ENSG00000075089 | ENST00000548180 |
| PTPN14 | ENSG00000152104 | ENST00000366956 |
| WHSC1 | ENSG00000109685 | ENST00000398261 |
| CBL | ENSG00000110395 | ENST00000264033 |
| PLXNC1 | ENSG00000136040 | ENST00000258526 |
| DNA2 | ENSG00000138346 | ENST00000551118 |
| EIF4E2 | ENSG00000135930 | ENST00000409514 |
| NLGN4X | ENSG00000146938 | ENST00000381095 |
| OSTM1 | ENSG00000081087 | ENST00000193322 |
| XKR4 | ENSG00000206579 | ENST00000327381 |
| TENM1 | ENSG00000009694 | ENST00000371130 |
| FAM9C | ENSG00000187268 | ENST00000542843 |
| ADD3 | ENSG00000148700 | ENST00000277900 |
| WDR70 | ENSG00000082068 | ENST00000265107 |
| IPO8 | ENSG00000133704 | ENST00000256079 |
| BDNF | ENSG00000176697 | ENST00000530786 |
| MAP4K4 | ENSG00000071054 | ENST00000413150 |
| ZBED3 | ENSG00000132846 | ENST00000255198 |
| PPP2R2C | ENSG00000074211 | ENST00000335585 |
| RGL1 | ENSG00000143344 | ENST00000360851 |
| GPATCH2L | ENSG00000089916 | ENST00000261530 |
| GXYLT1 | ENSG00000151233 | ENST00000398675 |
| KIAA2018 | ENSG00000176542 | ENST00000316407 |
| ROBO2 | ENSG00000185008 | ENST00000461745 |
| PPP1R9A | ENSG00000158528 | ENST00000456331 |
| MEX3B | ENSG00000183496 | ENST00000558133 |
| NOVA1 | ENSG00000139910 | ENST00000483536 |
| SOS1 | ENSG00000115904 | ENST00000426016 |
| STX16 | ENSG00000124222 | ENST00000371132 |
| COPS8 | ENSG00000198612 | ENST00000354371 |
| SLC16A2 | ENSG00000147100 | ENST00000587091 |
| TMEM220 | ENSG00000187824 | ENST00000341871 |
| FRMD6 | ENSG00000139926 | ENST00000395718 |
| LIN7A | ENSG00000111052 | ENST00000552864 |
| LRRTM3 | ENSG00000198739 | ENST00000361320 |
| SYNCRIP | ENSG00000135316 | ENST00000355238 |
| SRP72 | ENSG00000174780 | ENST00000342756 |
| MBNL2 | ENSG00000139793 | ENST00000345429 |
| TOB1 | ENSG00000141232 | ENST00000499247 |
| ZFYVE20 | ENSG00000131381 | ENST00000253699 |
| KLF9 | ENSG00000119138 | ENST00000377126 |
| B3GNT1 | ENSG00000174684 | ENST00000311181 |
| MAMDC2 | ENSG00000165072 | ENST00000377182 |
| FHL1 | ENSG00000022267 | ENST00000394155 |
| ABI2 | ENSG00000138443 | ENST00000295851 |
| KLHL3 | ENSG00000146021 | ENST00000508657 |
| PCDH10 | ENSG00000138650 | ENST00000264360 |
| HOOK1 | ENSG00000134709 | ENST00000371208 |
| RBM46 | ENSG00000151962 | ENST00000510397 |
| GLI3 | ENSG00000106571 | ENST00000395925 |
| IAH1 | ENSG00000134330 | ENST00000470914 |
| DAZ3 | ENSG00000187191 | ENST00000382365 |
| DAZ1 | ENSG00000188120 | ENST00000405239 |
| ANK3 | ENSG00000151150 | ENST00000280772 |
| HSPA9 | ENSG00000113013 | ENST00000297185 |
| CEP85L | ENSG00000111860 | ENST00000368491 |
| GMFB | ENSG00000197045 | ENST00000554908 |
| IGSF10 | ENSG00000152580 | ENST00000282466 |
| UBN2 | ENSG00000157741 | ENST00000473989 |
| SNAPC1 | ENSG00000023608 | ENST00000216294 |
| CBWD3 | ENSG00000196873 | ENST00000478048 |
| LCOR | ENSG00000196233 | ENST00000540664 |
| MAP4K5 | ENSG00000012983 | ENST00000013125 |
| SNX16 | ENSG00000104497 | ENST00000353788 |
| CRTAP | ENSG00000170275 | ENST00000320954 |
| DAZ2 | ENSG00000205944 | ENST00000382449 |
| GOLGA8F | ENSG00000153684 | ENST00000337838 |
| DLC1 | ENSG00000164741 | ENST00000276297 |
| VAT1L | ENSG00000171724 | ENST00000302536 |
| AFF4 | ENSG00000072364 | ENST00000265343 |
| PGM2L1 | ENSG00000165434 | ENST00000298198 |
| SLITRK1 | ENSG00000178235 | ENST00000377084 |
| WNT16 | ENSG00000002745 | ENST00000222462 |
| INO80D | ENSG00000114933 | ENST00000403263 |
| ANLN | ENSG00000011426 | ENST00000396068 |
| KIAA0430 | ENSG00000166783 | ENST00000396368 |
| MYO9A | ENSG00000066933 | ENST00000356056 |
| SLK | ENSG00000065613 | ENST00000369755 |
| GPR27 | ENSG00000170837 | ENST00000304411 |
| COPS2 | ENSG00000166200 | ENST00000388901 |
| TBX5 | ENSG00000089225 | ENST00000349716 |
| CTNNA3 | ENSG00000183230 | ENST00000433211 |
| DYX1C1 | ENSG00000256061 | ENST00000524160 |
| ULK2 | ENSG00000083290 | ENST00000395544 |
| CNOT6L | ENSG00000138767 | ENST00000264903 |
| TLN1 | ENSG00000137076 | ENST00000314888 |
| ZMYM4 | ENSG00000146463 | ENST00000457946 |
| TBL1XR1 | ENSG00000177565 | ENST00000430069 |
| SNTB2 | ENSG00000168807 | ENST00000336278 |
| WDPCP | ENSG00000143951 | ENST00000417238 |
| PPM1F | ENSG00000100034 | ENST00000263212 |
| SNX30 | ENSG00000148158 | ENST00000374232 |
| CGGBP1 | ENSG00000163320 | ENST00000309534 |
| ZYG11B | ENSG00000162378 | ENST00000294353 |
| BRWD3 | ENSG00000165288 | ENST00000373275 |
| KIAA1456 | ENSG00000250305 | ENST00000524591 |
| CCDC144NL | ENSG00000205212 | ENST00000327925 |
| TIFA | ENSG00000145365 | ENST00000361717 |
| SLC25A27 | ENSG00000153291 | ENST00000371347 |
| SCAI | ENSG00000173611 | ENST00000336505 |
| ZC3H6 | ENSG00000188177 | ENST00000343936 |
| AGFG1 | ENSG00000173744 | ENST00000310078 |
| PDS5B | ENSG00000083642 | ENST00000315596 |
| WDR91 | ENSG00000105875 | ENST00000344400 |
| CCSER2 | ENSG00000107771 | ENST00000224756 |
| CERS6 | ENSG00000172292 | ENST00000392687 |
| ZDHHC17 | ENSG00000186908 | ENST00000426126 |
| SNAP91 | ENSG00000065609 | ENST00000518312 |
| SPAG9 | ENSG00000008294 | ENST00000262013 |
| CLASP2 | ENSG00000163539 | ENST00000539981 |
| G6PC | ENSG00000131482 | ENST00000253801 |
| BICC1 | ENSG00000122870 | ENST00000373886 |
| HBS1L | ENSG00000112339 | ENST00000367837 |
| ZNF711 | ENSG00000147180 | ENST00000360700 |
| FARP1 | ENSG00000152767 | ENST00000595437 |
| SLC4A7 | ENSG00000033867 | ENST00000428386 |
| NPY1R | ENSG00000164128 | ENST00000296533 |
| SYVN1 | ENSG00000162298 | ENST00000294256 |
| MMD2 | ENSG00000136297 | ENST00000406755 |
| ELAVL4 | ENSG00000162374 | ENST00000371824 |
| RAB11FIP2 | ENSG00000107560 | ENST00000355624 |
| TCAIM | ENSG00000179152 | ENST00000431657 |
| FAM179B | ENSG00000198718 | ENST00000557423 |
| FBXO10 | ENSG00000147912 | ENST00000276960 |
| SNAP25 | ENSG00000132639 | ENST00000254976 |
| POLB | ENSG00000070501 | ENST00000522610 |
| DNAJB9 | ENSG00000128590 | ENST00000249356 |
| FBXO30 | ENSG00000118496 | ENST00000237281 |
| AC023632.1 | ENSG00000212997 | ENST00000391679 |
| SUV420H1 | ENSG00000110066 | ENST00000304363 |
| WDR11 | ENSG00000120008 | ENST00000497136 |
| RTKN2 | ENSG00000182010 | ENST00000373789 |
| ZBTB38 | ENSG00000177311 | ENST00000514251 |
| EPS8 | ENSG00000151491 | ENST00000543468 |
| PAX6 | ENSG00000007372 | ENST00000419022 |
| FRMD4A | ENSG00000151474 | ENST00000358621 |
| HECTD2 | ENSG00000165338 | ENST00000371667 |
| ZCCHC24 | ENSG00000165424 | ENST00000372336 |
| SLC1A2 | ENSG00000110436 | ENST00000278379 |
| ATMIN | ENSG00000166454 | ENST00000299575 |
| PIKFYVE | ENSG00000115020 | ENST00000264380 |
| NOVA2 | ENSG00000104967 | ENST00000263257 |
| SULF1 | ENSG00000137573 | ENST00000458141 |
| FEZ2 | ENSG00000171055 | ENST00000413938 |
| PLXNA4 | ENSG00000221866 | ENST00000321063 |
| GPRASP2 | ENSG00000158301 | ENST00000535209 |
| ZNF281 | ENSG00000162702 | ENST00000294740 |
| AEBP2 | ENSG00000139154 | ENST00000266508 |
| SLC25A36 | ENSG00000114120 | ENST00000446041 |
| ZMYND8 | ENSG00000101040 | ENST00000311275 |
| ITSN2 | ENSG00000198399 | ENST00000416160 |
| PLK2 | ENSG00000145632 | ENST00000274289 |
| AC107021.1 | ENSG00000232013 | ENST00000422482 |
| RPS6KA3 | ENSG00000177189 | ENST00000379565 |
| UBA6 | ENSG00000033178 | ENST00000322244 |
| RNF19A | ENSG00000034677 | ENST00000519449 |
| REV1 | ENSG00000135945 | ENST00000413697 |
| NPTX1 | ENSG00000171246 | ENST00000306773 |
| ELK4 | ENSG00000158711 | ENST00000357992 |
| VLDLR | ENSG00000147852 | ENST00000382100 |
| PDE5A | ENSG00000138735 | ENST00000354960 |
| ZBTB25 | ENSG00000089775 | ENST00000608382 |
| BICD1 | ENSG00000151746 | ENST00000548411 |
| SERF1A | ENSG00000172058 | ENST00000354833 |
| PATE4 | ENSG00000237353 | ENST00000457514 |
| NFIB | ENSG00000147862 | ENST00000380934 |
| ZSWIM4 | ENSG00000132003 | ENST00000254323 |
| LRIG1 | ENSG00000144749 | ENST00000383703 |
| KDELC1 | ENSG00000134901 | ENST00000376004 |
| SETD3 | ENSG00000183576 | ENST00000357563 |
| INHBA | ENSG00000122641 | ENST00000242208 |
| CAPRIN2 | ENSG00000110888 | ENST00000537553 |
| PAN3 | ENSG00000152520 | ENST00000282391 |
| PIK3CA | ENSG00000121879 | ENST00000263967 |
| STK38L | ENSG00000211455 | ENST00000389032 |
| PRMT3 | ENSG00000185238 | ENST00000330796 |
| NRBF2 | ENSG00000148572 | ENST00000277746 |
| MIB1 | ENSG00000101752 | ENST00000261537 |
| DNAJB14 | ENSG00000164031 | ENST00000442697 |
| NAP1L5 | ENSG00000177432 | ENST00000323061 |
| RET | ENSG00000165731 | ENST00000355710 |
| ROCK2 | ENSG00000134318 | ENST00000315872 |
| TMEM26 | ENSG00000196932 | ENST00000399298 |
| HOXA5 | ENSG00000106004 | ENST00000222726 |
| STXBP6 | ENSG00000168952 | ENST00000396700 |
| MFAP5 | ENSG00000197614 | ENST00000359478 |
| ARHGAP20 | ENSG00000137727 | ENST00000260283 |
| HNF1B | ENSG00000108753 | ENST00000225893 |
| BASP1 | ENSG00000176788 | ENST00000322611 |
| TMOD3 | ENSG00000138594 | ENST00000308580 |
| KIAA0355 | ENSG00000166398 | ENST00000299505 |
| CDH6 | ENSG00000113361 | ENST00000265071 |
| AC006455.1 | ENSG00000230000 | ENST00000456890 |
| TOPORS | ENSG00000197579 | ENST00000360538 |
| RQCD1 | ENSG00000144580 | ENST00000273064 |
| DOCK4 | ENSG00000128512 | ENST00000423057 |
| GRIP1 | ENSG00000155974 | ENST00000359742 |
| LETM2 | ENSG00000165046 | ENST00000523268 |
| MYCN | ENSG00000134323 | ENST00000281043 |
| DZIP1 | ENSG00000134874 | ENST00000361156 |
| MIEF1 | ENSG00000100335 | ENST00000325301 |
| PLCG1 | ENSG00000124181 | ENST00000373271 |
| PIGM | ENSG00000143315 | ENST00000368090 |
| SERPINI1 | ENSG00000163536 | ENST00000446050 |
| YIPF4 | ENSG00000119820 | ENST00000238831 |
| MKLN1 | ENSG00000128585 | ENST00000352689 |
| C2orf73 | ENSG00000177994 | ENST00000317627 |
| JMY | ENSG00000152409 | ENST00000396137 |
| CBX5 | ENSG00000094916 | ENST00000209875 |
| SETD7 | ENSG00000145391 | ENST00000274031 |
| PCNX | ENSG00000100731 | ENST00000304743 |
| ELMOD1 | ENSG00000110675 | ENST00000265840 |
| CHMP5 | ENSG00000086065 | ENST00000419016 |
| NOTCH1 | ENSG00000148400 | ENST00000277541 |
| RANBP10 | ENSG00000141084 | ENST00000317506 |
| PAK7 | ENSG00000101349 | ENST00000378429 |
| MMP16 | ENSG00000156103 | ENST00000286614 |
| GOLGA7 | ENSG00000147533 | ENST00000405786 |
| NCOA2 | ENSG00000140396 | ENST00000452400 |
| STK4 | ENSG00000101109 | ENST00000372806 |
| TLL2 | ENSG00000095587 | ENST00000357947 |
| E2F3 | ENSG00000112242 | ENST00000346618 |
| LRRC58 | ENSG00000163428 | ENST00000295628 |
| NBR1 | ENSG00000188554 | ENST00000341165 |
| FUBP3 | ENSG00000107164 | ENST00000319725 |
| ZBTB10 | ENSG00000205189 | ENST00000430430 |
| TFAP2A | ENSG00000137203 | ENST00000489805 |
| LHX9 | ENSG00000143355 | ENST00000367390 |
| CDH8 | ENSG00000150394 | ENST00000585315 |
| HEG1 | ENSG00000173706 | ENST00000311127 |
| C6orf120 | ENSG00000185127 | ENST00000332290 |
| ZNF773 | ENSG00000152439 | ENST00000599847 |
| EXOSC3 | ENSG00000107371 | ENST00000465229 |
| YWHAQ | ENSG00000134308 | ENST00000238081 |
| MBD5 | ENSG00000204406 | ENST00000416015 |
| FOXN2 | ENSG00000170802 | ENST00000340553 |
| ATAD2B | ENSG00000119778 | ENST00000238789 |
| CLVS2 | ENSG00000146352 | ENST00000275162 |
| ATXN1 | ENSG00000124788 | ENST00000244769 |
| THAP2 | ENSG00000173451 | ENST00000308086 |
| TXLNG | ENSG00000086712 | ENST00000380122 |
| CHM | ENSG00000188419 | ENST00000357749 |
| ANGEL2 | ENSG00000174606 | ENST00000360506 |
| RNGTT | ENSG00000111880 | ENST00000369485 |
| EGFR | ENSG00000146648 | ENST00000275493 |
| ZBTB8A | ENSG00000160062 | ENST00000373510 |
| AMOTL2 | ENSG00000114019 | ENST00000249883 |
| REEP1 | ENSG00000068615 | ENST00000165698 |
| GRID2 | ENSG00000152208 | ENST00000282020 |
| EIF2S3L | ENSG00000180574 | ENST00000322446 |
| GLRA3 | ENSG00000145451 | ENST00000274093 |
| NIPBL | ENSG00000164190 | ENST00000282516 |
| DNAJC10 | ENSG00000077232 | ENST00000264065 |
| C11orf87 | ENSG00000185742 | ENST00000327419 |
| DAZ4 | ENSG00000205916 | ENST00000382290 |
| UGCG | ENSG00000148154 | ENST00000374279 |
| COX11 | ENSG00000166260 | ENST00000576370 |
| SRF | ENSG00000112658 | ENST00000265354 |
| DDX1 | ENSG00000079785 | ENST00000381341 |
| XPO1 | ENSG00000082898 | ENST00000428210 |
| RNF2 | ENSG00000121481 | ENST00000367510 |
| ATP11C | ENSG00000101974 | ENST00000450801 |
| GATA2 | ENSG00000179348 | ENST00000341105 |
| PLEKHA8 | ENSG00000106086 | ENST00000440706 |
| MORC3 | ENSG00000159256 | ENST00000400485 |
| CSNK1G3 | ENSG00000151292 | ENST00000360683 |
| NDN | ENSG00000182636 | ENST00000331837 |
| RIPK2 | ENSG00000104312 | ENST00000522965 |
| IKBKB | ENSG00000104365 | ENST00000342222 |
| PTPRZ1 | ENSG00000106278 | ENST00000393386 |
| NRG1 | ENSG00000157168 | ENST00000341377 |
| KRR1 | ENSG00000111615 | ENST00000229214 |
| CYTH1 | ENSG00000108669 | ENST00000591455 |
| SLFN12 | ENSG00000172123 | ENST00000394562 |
| ZC3H4 | ENSG00000130749 | ENST00000253048 |
| C17orf85 | ENSG00000074356 | ENST00000574911 |
| GATSL2 | ENSG00000198750 | ENST00000426327 |
| C10orf91 | ENSG00000180066 | ENST00000392630 |
| PPP1R10 | ENSG00000204569 | ENST00000376511 |
| NRIP1 | ENSG00000180530 | ENST00000400202 |
| CHRDL1 | ENSG00000101938 | ENST00000372045 |
| ZNF423 | ENSG00000102935 | ENST00000561648 |
| PHACTR2 | ENSG00000112419 | ENST00000427704 |
| DR1 | ENSG00000117505 | ENST00000370272 |
| PKIA | ENSG00000171033 | ENST00000396418 |
| CAMSAP2 | ENSG00000118200 | ENST00000358823 |
| HMGB3 | ENSG00000029993 | ENST00000325307 |
| SLC15A2 | ENSG00000163406 | ENST00000489711 |
| SNRPB2 | ENSG00000125870 | ENST00000377943 |
| SIX3 | ENSG00000138083 | ENST00000260653 |
| EPS15 | ENSG00000085832 | ENST00000371730 |
| EXOG | ENSG00000157036 | ENST00000457367 |
| KIF14 | ENSG00000118193 | ENST00000367350 |
| AMER2 | ENSG00000165566 | ENST00000357816 |
| AMBRA1 | ENSG00000110497 | ENST00000534300 |
| DESI1 | ENSG00000100418 | ENST00000263256 |
| ARL2BP | ENSG00000102931 | ENST00000219204 |
| RWDD2A | ENSG00000013392 | ENST00000369724 |
| LMAN1 | ENSG00000074695 | ENST00000251047 |
| CPEB3 | ENSG00000107864 | ENST00000412050 |
| TAOK1 | ENSG00000160551 | ENST00000261716 |
| ASAP1 | ENSG00000153317 | ENST00000521075 |
| TMEM170B | ENSG00000205269 | ENST00000379426 |
| RANBP17 | ENSG00000204764 | ENST00000522066 |
| PAPD5 | ENSG00000121274 | ENST00000357464 |
| VWC2 | ENSG00000188730 | ENST00000340652 |
| MOSPD2 | ENSG00000130150 | ENST00000380492 |
| DMD | ENSG00000198947 | ENST00000541735 |
| PTK2 | ENSG00000169398 | ENST00000519993 |
| BNC2 | ENSG00000173068 | ENST00000380672 |
| GDI2 | ENSG00000057608 | ENST00000380191 |
| CACUL1 | ENSG00000151893 | ENST00000369151 |
| RIMKLB | ENSG00000166532 | ENST00000357529 |
| CRYBG3 | ENSG00000080200 | ENST00000389622 |
| OCLN | ENSG00000197822 | ENST00000355237 |
| TAF9B | ENSG00000187325 | ENST00000341864 |
| LNPEP | ENSG00000113441 | ENST00000231368 |
| MTCP1 | ENSG00000214827 | ENST00000369476 |
| MPP6 | ENSG00000105926 | ENST00000222644 |
| GOLGA8B | ENSG00000215252 | ENST00000342314 |
| TP53TG3C | ENSG00000205457 | ENST00000569719 |
| PRKG1 | ENSG00000185532 | ENST00000373985 |
| LIN28B | ENSG00000187772 | ENST00000345080 |
| OTUD6B | ENSG00000155100 | ENST00000285420 |
| TP53TG3D | ENSG00000205456 | ENST00000380148 |
| TP53TG3 | ENSG00000183632 | ENST00000565622 |
| ABRA | ENSG00000174429 | ENST00000311955 |
| NTF3 | ENSG00000185652 | ENST00000423158 |
| CYP11B1 | ENSG00000160882 | ENST00000519285 |
| FRS2 | ENSG00000166225 | ENST00000550389 |
| KDSR | ENSG00000119537 | ENST00000406396 |
| CALU | ENSG00000128595 | ENST00000535623 |
| STRN | ENSG00000115808 | ENST00000263918 |
| NEDD1 | ENSG00000139350 | ENST00000266742 |
| FRMD4B | ENSG00000114541 | ENST00000398540 |
| MED6 | ENSG00000133997 | ENST00000256379 |
| ZBTB37 | ENSG00000185278 | ENST00000367701 |
| SLC5A3 | ENSG00000198743 | ENST00000381151 |
| KIF13A | ENSG00000137177 | ENST00000378814 |
| DDX26B | ENSG00000165359 | ENST00000370752 |
| TYW5 | ENSG00000162971 | ENST00000483328 |
| ODF2L | ENSG00000122417 | ENST00000359242 |
| DIRAS2 | ENSG00000165023 | ENST00000375765 |
| LATS1 | ENSG00000131023 | ENST00000543571 |
| TROVE2 | ENSG00000116747 | ENST00000432079 |
| FAM60A | ENSG00000139146 | ENST00000337682 |
| TARDBP | ENSG00000120948 | ENST00000240185 |
| CTDSPL2 | ENSG00000137770 | ENST00000260327 |
| EPM2AIP1 | ENSG00000178567 | ENST00000322716 |
| MBLAC2 | ENSG00000176055 | ENST00000316610 |
| TMA16 | ENSG00000198498 | ENST00000358572 |
| PROK2 | ENSG00000163421 | ENST00000353065 |
| ANKRD40 | ENSG00000154945 | ENST00000285243 |
| NLRP11 | ENSG00000179873 | ENST00000590409 |
| ELMOD2 | ENSG00000179387 | ENST00000323570 |
| CAMK4 | ENSG00000152495 | ENST00000282356 |
| DENND4A | ENSG00000174485 | ENST00000443035 |
| C18orf63 | ENSG00000206043 | ENST00000579455 |
| AP1S2 | ENSG00000182287 | ENST00000380291 |
| TRIM71 | ENSG00000206557 | ENST00000383763 |
| PPM1A | ENSG00000100614 | ENST00000395076 |
| TP73 | ENSG00000078900 | ENST00000604074 |
| FAR2 | ENSG00000064763 | ENST00000536681 |
| RNF180 | ENSG00000164197 | ENST00000389100 |
| PARD3B | ENSG00000116117 | ENST00000406610 |
| PRRC2C | ENSG00000117523 | ENST00000338920 |
| SPG20 | ENSG00000133104 | ENST00000438666 |
| EDEM3 | ENSG00000116406 | ENST00000318130 |
| HS2ST1 | ENSG00000153936 | ENST00000370550 |
| CSRNP3 | ENSG00000178662 | ENST00000314499 |
| MRPL32 | ENSG00000106591 | ENST00000432845 |
| ICK | ENSG00000112144 | ENST00000350082 |
| ACVR1C | ENSG00000123612 | ENST00000243349 |
| KLRF1 | ENSG00000150045 | ENST00000279545 |
| MON1B | ENSG00000103111 | ENST00000248248 |
| FAM19A5 | ENSG00000219438 | ENST00000358295 |
| HNRNPK | ENSG00000165119 | ENST00000376281 |
| PDCD10 | ENSG00000114209 | ENST00000392750 |
| RALGAPA1 | ENSG00000174373 | ENST00000389698 |
| XRN2 | ENSG00000088930 | ENST00000377191 |
| TEC | ENSG00000135605 | ENST00000505452 |
| NCOA3 | ENSG00000124151 | ENST00000372004 |
| FAM76B | ENSG00000077458 | ENST00000398187 |
| RBM12B | ENSG00000183808 | ENST00000399300 |
| THUMPD3 | ENSG00000134077 | ENST00000345094 |
| PSPH | ENSG00000146733 | ENST00000275605 |
| MTMR10 | ENSG00000166912 | ENST00000568604 |
| TP53INP1 | ENSG00000164938 | ENST00000448464 |
| PCMTD1 | ENSG00000168300 | ENST00000544451 |
| KCNJ6 | ENSG00000157542 | ENST00000609713 |
| PRKAB2 | ENSG00000131791 | ENST00000254101 |
| LCA5 | ENSG00000135338 | ENST00000369846 |
| SCAMP1 | ENSG00000085365 | ENST00000538629 |
| TAP2 | ENSG00000204267 | ENST00000374897 |
| CHAF1B | ENSG00000159259 | ENST00000314103 |
| RAB33B | ENSG00000172007 | ENST00000305626 |
| KCNQ3 | ENSG00000184156 | ENST00000388996 |
| TMEM178A | ENSG00000152154 | ENST00000281961 |
| SDK2 | ENSG00000069188 | ENST00000392650 |
| WSB1 | ENSG00000109046 | ENST00000262394 |
| COL4A3 | ENSG00000169031 | ENST00000396578 |
| PHF6 | ENSG00000156531 | ENST00000332070 |
| IPMK | ENSG00000151151 | ENST00000373935 |
| LMBRD2 | ENSG00000164187 | ENST00000296603 |
| TBCA | ENSG00000171530 | ENST00000306388 |
| ZNF697 | ENSG00000143067 | ENST00000421812 |
| B4GALT6 | ENSG00000118276 | ENST00000306851 |
| SCN5A | ENSG00000183873 | ENST00000414099 |
| DCAF17 | ENSG00000115827 | ENST00000375255 |
| ATP11B | ENSG00000058063 | ENST00000323116 |
| BRCA1 | ENSG00000012048 | ENST00000461221 |
| CBX4 | ENSG00000141582 | ENST00000269397 |
| MGAT2 | ENSG00000168282 | ENST00000305386 |
| SOX6 | ENSG00000110693 | ENST00000352083 |
| CHSY1 | ENSG00000131873 | ENST00000254190 |
| TRIM62 | ENSG00000116525 | ENST00000291416 |
| VGLL3 | ENSG00000206538 | ENST00000398399 |
| ETF1 | ENSG00000120705 | ENST00000499810 |
| AL353791.1 | ENSG00000227921 | ENST00000426179 |
| KLF12 | ENSG00000118922 | ENST00000377669 |
| HIPK1 | ENSG00000163349 | ENST00000369558 |
| FAM3B | ENSG00000183844 | ENST00000398647 |
| ETV5 | ENSG00000244405 | ENST00000306376 |
| CHST2 | ENSG00000175040 | ENST00000309575 |
| DGKE | ENSG00000153933 | ENST00000284061 |
| ZNF207 | ENSG00000010244 | ENST00000394670 |
| PIM2 | ENSG00000102096 | ENST00000376509 |
| ZNF302 | ENSG00000089335 | ENST00000505365 |
| CDC27 | ENSG00000004897 | ENST00000066544 |
| SLC4A4 | ENSG00000080493 | ENST00000340595 |
| DCBLD2 | ENSG00000057019 | ENST00000326840 |
| EIF4E3 | ENSG00000163412 | ENST00000425534 |
| OTUD4 | ENSG00000164164 | ENST00000454497 |
| LRCH2 | ENSG00000130224 | ENST00000317135 |
| SUGT1 | ENSG00000165416 | ENST00000310528 |
| MSH2 | ENSG00000095002 | ENST00000406134 |
| PPIL4 | ENSG00000131013 | ENST00000253329 |
| TSTD2 | ENSG00000136925 | ENST00000341170 |
| KALRN | ENSG00000160145 | ENST00000291478 |
| SAMD8 | ENSG00000156671 | ENST00000372687 |
| EIF2S1 | ENSG00000134001 | ENST00000256383 |
| FN1 | ENSG00000115414 | ENST00000357009 |
| SMARCAD1 | ENSG00000163104 | ENST00000394961 |
| SLC30A10 | ENSG00000196660 | ENST00000356609 |
| PIH1D3 | ENSG00000080572 | ENST00000372453 |
| APAF1 | ENSG00000120868 | ENST00000333991 |
| RALGPS2 | ENSG00000116191 | ENST00000367635 |
| RSL24D1 | ENSG00000137876 | ENST00000260443 |
| CCNE2 | ENSG00000175305 | ENST00000520509 |
| MED1 | ENSG00000125686 | ENST00000577831 |
| BPTF | ENSG00000171634 | ENST00000342579 |
| ASF1A | ENSG00000111875 | ENST00000229595 |
| GDAP1 | ENSG00000104381 | ENST00000220822 |
| KIAA1244 | ENSG00000112379 | ENST00000251691 |
| PUM2 | ENSG00000055917 | ENST00000338086 |
| FBXL16 | ENSG00000127585 | ENST00000397621 |
| PARD6B | ENSG00000124171 | ENST00000371610 |
| HECW2 | ENSG00000138411 | ENST00000260983 |
| PCDH8 | ENSG00000136099 | ENST00000377942 |
| DENND6A | ENSG00000174839 | ENST00000311128 |
| TLK1 | ENSG00000198586 | ENST00000359766 |
| SLC8A1 | ENSG00000183023 | ENST00000406785 |
| PDK1 | ENSG00000152256 | ENST00000282077 |
| SPATS2L | ENSG00000196141 | ENST00000358677 |
| S100PBP | ENSG00000116497 | ENST00000373475 |
| GPR107 | ENSG00000148358 | ENST00000372410 |
| ING2 | ENSG00000168556 | ENST00000302327 |
| MORF4L1 | ENSG00000185787 | ENST00000331268 |
| ARHGAP6 | ENSG00000047648 | ENST00000495242 |
| FSD1L | ENSG00000106701 | ENST00000374710 |
| AKT3 | ENSG00000117020 | ENST00000366539 |
| RIMS2 | ENSG00000176406 | ENST00000507740 |
| ZBTB5 | ENSG00000168795 | ENST00000307750 |
| GUCY1A3 | ENSG00000164116 | ENST00000443668 |
| NCOA4 | ENSG00000138293 | ENST00000452682 |
| CASC4 | ENSG00000166734 | ENST00000360824 |
| CYYR1 | ENSG00000166265 | ENST00000299340 |
| PPP6R3 | ENSG00000110075 | ENST00000265637 |
| ZDHHC21 | ENSG00000175893 | ENST00000380916 |
| HSPA13 | ENSG00000155304 | ENST00000285667 |
| DCAF10 | ENSG00000122741 | ENST00000242323 |
| GPC6 | ENSG00000183098 | ENST00000377047 |
| AL953854.2 | ENSG00000236029 | ENST00000420228 |
| SCN8A | ENSG00000196876 | ENST00000354534 |
| SMIM5 | ENSG00000204323 | ENST00000375215 |
| ANKS3 | ENSG00000168096 | ENST00000592077 |
| BAG6 | ENSG00000204463 | ENST00000211379 |
| SLC7A11 | ENSG00000151012 | ENST00000280612 |
| ACTC1 | ENSG00000159251 | ENST00000290378 |
| HPS5 | ENSG00000110756 | ENST00000396253 |
| RIF1 | ENSG00000080345 | ENST00000243326 |
| YTHDF3 | ENSG00000185728 | ENST00000524135 |
| MOB4 | ENSG00000115540 | ENST00000233892 |
| PLAA | ENSG00000137055 | ENST00000397292 |
| PPAP2B | ENSG00000162407 | ENST00000371250 |
| MBOAT2 | ENSG00000143797 | ENST00000305997 |
| GUCY1A2 | ENSG00000152402 | ENST00000526355 |
| SPTSSA | ENSG00000165389 | ENST00000298130 |
| KCTD8 | ENSG00000183783 | ENST00000360029 |
| TREM1 | ENSG00000124731 | ENST00000591620 |
| CLCF1 | ENSG00000175505 | ENST00000312438 |
| LIFR | ENSG00000113594 | ENST00000263409 |
| PPP2R1B | ENSG00000137713 | ENST00000527614 |
| FUBP1 | ENSG00000162613 | ENST00000370767 |
| RBFOX1 | ENSG00000078328 | ENST00000340209 |
| PBX3 | ENSG00000167081 | ENST00000342287 |
| CDK2 | ENSG00000123374 | ENST00000555408 |
| UBE2R2 | ENSG00000107341 | ENST00000263228 |
| CHRM2 | ENSG00000181072 | ENST00000445907 |
| ZNF708 | ENSG00000182141 | ENST00000598046 |
| MAP2K5 | ENSG00000137764 | ENST00000178640 |
| PTAR1 | ENSG00000188647 | ENST00000377200 |
| ZFHX4 | ENSG00000091656 | ENST00000521891 |
| RRAS2 | ENSG00000133818 | ENST00000537760 |
| LMO7 | ENSG00000136153 | ENST00000341547 |
| CALCR | ENSG00000004948 | ENST00000359558 |
| HLCS | ENSG00000159267 | ENST00000336648 |
| EID1 | ENSG00000255302 | ENST00000530028 |
| GULP1 | ENSG00000144366 | ENST00000451191 |
| TTC5 | ENSG00000136319 | ENST00000258821 |
| EXT1 | ENSG00000182197 | ENST00000378204 |
| LYRM2 | ENSG00000083099 | ENST00000523377 |
| EXOSC9 | ENSG00000123737 | ENST00000513654 |
| PHC3 | ENSG00000173889 | ENST00000495893 |
| ZMAT3 | ENSG00000172667 | ENST00000311417 |
| DDX53 | ENSG00000184735 | ENST00000327968 |
| TLR4 | ENSG00000136869 | ENST00000355622 |
| SMAD2 | ENSG00000175387 | ENST00000402690 |
| MAP3K2 | ENSG00000169967 | ENST00000409947 |
| RDX | ENSG00000137710 | ENST00000343115 |
| SCML1 | ENSG00000047634 | ENST00000380043 |
| NUDT4 | ENSG00000173598 | ENST00000415493 |
| ICA1L | ENSG00000163596 | ENST00000392237 |
| C5orf56 | ENSG00000197536 | ENST00000337752 |
| KLF4 | ENSG00000136826 | ENST00000374672 |
| MMAA | ENSG00000151611 | ENST00000281317 |
| FNBP4 | ENSG00000109920 | ENST00000263773 |
| PCDH11Y | ENSG00000099715 | ENST00000400457 |
| TERF1 | ENSG00000147601 | ENST00000276602 |
| ARL3 | ENSG00000138175 | ENST00000260746 |
| INPP4A | ENSG00000040933 | ENST00000409016 |
| SSFA2 | ENSG00000138434 | ENST00000440623 |
| KHDRBS2 | ENSG00000112232 | ENST00000281156 |
| FXR2 | ENSG00000129245 | ENST00000250113 |
| DDX3Y | ENSG00000067048 | ENST00000336079 |
| MMS22L | ENSG00000146263 | ENST00000275053 |
| SCOC | ENSG00000153130 | ENST00000608372 |
| GAB1 | ENSG00000109458 | ENST00000262995 |
| CNTNAP3B | ENSG00000154529 | ENST00000377564 |
| PAG1 | ENSG00000076641 | ENST00000220597 |
| CARF | ENSG00000138380 | ENST00000402905 |
| CFHR5 | ENSG00000134389 | ENST00000367414 |
| SHROOM4 | ENSG00000158352 | ENST00000376020 |
| PLXNA2 | ENSG00000076356 | ENST00000367033 |
| RAB21 | ENSG00000080371 | ENST00000261263 |
| SH3GL1 | ENSG00000141985 | ENST00000269886 |
| CPED1 | ENSG00000106034 | ENST00000310396 |
| PTCHD1 | ENSG00000165186 | ENST00000379361 |
| DRP2 | ENSG00000102385 | ENST00000402866 |
| CNST | ENSG00000162852 | ENST00000366513 |
| MAL2 | ENSG00000147676 | ENST00000276681 |
| SLC35F5 | ENSG00000115084 | ENST00000245680 |
| TBRG1 | ENSG00000154144 | ENST00000284290 |
| CAST | ENSG00000153113 | ENST00000309190 |
| PHF21A | ENSG00000135365 | ENST00000257821 |
| NCOR2 | ENSG00000196498 | ENST00000405201 |
| NRXN1 | ENSG00000179915 | ENST00000342183 |
| HMGA2 | ENSG00000149948 | ENST00000541363 |
| SLC17A4 | ENSG00000146039 | ENST00000377905 |
| FAM219A | ENSG00000164970 | ENST00000379087 |
| NCKAP1 | ENSG00000061676 | ENST00000361354 |
| DPP10 | ENSG00000175497 | ENST00000410059 |
| XIAP | ENSG00000101966 | ENST00000371199 |
| BAZ2B | ENSG00000123636 | ENST00000392782 |
| CACHD1 | ENSG00000158966 | ENST00000290039 |
| VAPA | ENSG00000101558 | ENST00000400000 |
| TRIM59 | ENSG00000213186 | ENST00000543469 |
| TMEM167A | ENSG00000174695 | ENST00000502346 |
| PYGO1 | ENSG00000171016 | ENST00000302000 |
| FAM168B | ENSG00000152102 | ENST00000409185 |
| PRKRA | ENSG00000180228 | ENST00000424699 |
| BRIP1 | ENSG00000136492 | ENST00000259008 |
| MAP1B | ENSG00000131711 | ENST00000296755 |
| PPIP5K2 | ENSG00000145725 | ENST00000321521 |
| SH3PXD2A | ENSG00000107957 | ENST00000369774 |
| CCSAP | ENSG00000154429 | ENST00000366687 |
| KL | ENSG00000133116 | ENST00000380099 |
| KCNMB1 | ENSG00000145936 | ENST00000274629 |
| FUNDC1 | ENSG00000069509 | ENST00000378045 |
| MLIP | ENSG00000146147 | ENST00000514921 |
| ZFC3H1 | ENSG00000133858 | ENST00000552994 |
| SYT10 | ENSG00000110975 | ENST00000228567 |
| FUT4 | ENSG00000196371 | ENST00000358752 |
| SMAD5 | ENSG00000113658 | ENST00000545279 |
| SGPP1 | ENSG00000126821 | ENST00000247225 |
| GOLGA8G | ENSG00000183629 | ENST00000329523 |
| DLEU1 | ENSG00000176124 | ENST00000378180 |
| TMCC1 | ENSG00000172765 | ENST00000432054 |
| OTOGL | ENSG00000165899 | ENST00000551340 |
| CCDC109B | ENSG00000005059 | ENST00000394650 |
| CYP1B1 | ENSG00000138061 | ENST00000260630 |
| ACYP2 | ENSG00000170634 | ENST00000406041 |
| SKA3 | ENSG00000165480 | ENST00000298260 |
| LONRF2 | ENSG00000170500 | ENST00000393437 |
| BLM | ENSG00000197299 | ENST00000559724 |
| JADE3 | ENSG00000102221 | ENST00000397189 |
| SOX1 | ENSG00000182968 | ENST00000330949 |
| ZNF662 | ENSG00000182983 | ENST00000541208 |
| STRN3 | ENSG00000196792 | ENST00000355683 |
| PCDH11X | ENSG00000102290 | ENST00000373097 |
| ABCA1 | ENSG00000165029 | ENST00000374736 |
| TAF5L | ENSG00000135801 | ENST00000366675 |
| SLC35D1 | ENSG00000116704 | ENST00000235345 |
| ZNF395 | ENSG00000186918 | ENST00000344423 |
| RSBN1 | ENSG00000081019 | ENST00000261441 |
| TRIM2 | ENSG00000109654 | ENST00000338700 |
| KSR1 | ENSG00000141068 | ENST00000398988 |
| PTPN11 | ENSG00000179295 | ENST00000351677 |
| FAM210A | ENSG00000177150 | ENST00000322247 |
| H3F3A | ENSG00000163041 | ENST00000366814 |
| RBM20 | ENSG00000203867 | ENST00000369519 |
| RP1-27O5.3 | ENSG00000254553 | ENST00000480336 |
| RPRD1A | ENSG00000141425 | ENST00000399022 |
| MYNN | ENSG00000085274 | ENST00000544106 |
| FOXP2 | ENSG00000128573 | ENST00000441290 |
| FAM169A | ENSG00000198780 | ENST00000514215 |
| IRS1 | ENSG00000169047 | ENST00000305123 |
| CSMD3 | ENSG00000164796 | ENST00000297405 |
| HOOK3 | ENSG00000168172 | ENST00000307602 |
| DAZL | ENSG00000092345 | ENST00000250863 |
| DIEXF | ENSG00000117597 | ENST00000491415 |
| ALG6 | ENSG00000088035 | ENST00000371108 |
| FAM135B | ENSG00000147724 | ENST00000482951 |
| ZNF124 | ENSG00000196418 | ENST00000472531 |
| FOXR2 | ENSG00000189299 | ENST00000339140 |
| MYT1 | ENSG00000196132 | ENST00000328439 |
| SYNE1 | ENSG00000131018 | ENST00000367248 |
| SLC25A21 | ENSG00000183032 | ENST00000331299 |
| CREBL2 | ENSG00000111269 | ENST00000228865 |
| COG6 | ENSG00000133103 | ENST00000416691 |
| INSM2 | ENSG00000168348 | ENST00000307169 |
| SBSPON | ENSG00000164764 | ENST00000297354 |
| ESRP1 | ENSG00000104413 | ENST00000358397 |
| USP38 | ENSG00000170185 | ENST00000511739 |
| ARIH2 | ENSG00000177479 | ENST00000356401 |
| TAF12 | ENSG00000120656 | ENST00000373824 |
| DLGAP2 | ENSG00000198010 | ENST00000421627 |
| CORO1C | ENSG00000110880 | ENST00000261401 |
| DSC3 | ENSG00000134762 | ENST00000434452 |
| FGD1 | ENSG00000102302 | ENST00000375135 |
| MGA | ENSG00000174197 | ENST00000219905 |
| ZDHHC2 | ENSG00000104219 | ENST00000262096 |
| HMGCLL1 | ENSG00000146151 | ENST00000370852 |
| PIGX | ENSG00000163964 | ENST00000392391 |
| MUT | ENSG00000146085 | ENST00000274813 |
| DHX36 | ENSG00000174953 | ENST00000496811 |
| NPNT | ENSG00000168743 | ENST00000379987 |
| SIKE1 | ENSG00000052723 | ENST00000369528 |
| EDNRA | ENSG00000151617 | ENST00000339690 |
| C11orf58 | ENSG00000110696 | ENST00000228136 |
| TSPAN13 | ENSG00000106537 | ENST00000262067 |
| KLF10 | ENSG00000155090 | ENST00000285407 |
| GJA3 | ENSG00000121743 | ENST00000241125 |
| EP300 | ENSG00000100393 | ENST00000263253 |
| HIF1AN | ENSG00000166135 | ENST00000299163 |
| SURF4 | ENSG00000148248 | ENST00000371989 |
| PSAT1 | ENSG00000135069 | ENST00000376588 |
| RP11-219A15.1 | ENSG00000266302 | ENST00000448331 |
| FOSL2 | ENSG00000075426 | ENST00000379619 |
| BDP1 | ENSG00000145734 | ENST00000514903 |
| NR2F2 | ENSG00000185551 | ENST00000394171 |
| FUT9 | ENSG00000172461 | ENST00000302103 |
| TBC1D31 | ENSG00000156787 | ENST00000524307 |
| TET2 | ENSG00000168769 | ENST00000545826 |
| GATA4 | ENSG00000136574 | ENST00000335135 |
| PAK2 | ENSG00000180370 | ENST00000327134 |
| TRAPPC8 | ENSG00000153339 | ENST00000580104 |
| THSD7A | ENSG00000005108 | ENST00000423059 |
| NIN | ENSG00000100503 | ENST00000389868 |
| DNAJC5 | ENSG00000101152 | ENST00000470551 |
| FERMT2 | ENSG00000073712 | ENST00000341590 |
| CKAP4 | ENSG00000136026 | ENST00000378026 |
| BTF3L4 | ENSG00000134717 | ENST00000313334 |
| NAA16 | ENSG00000172766 | ENST00000379406 |
| FAM118B | ENSG00000197798 | ENST00000533050 |
| CLOCK | ENSG00000134852 | ENST00000309964 |
| PAM | ENSG00000145730 | ENST00000345721 |
| DGKA | ENSG00000065357 | ENST00000402956 |
| MKL2 | ENSG00000186260 | ENST00000318282 |
| FAM107B | ENSG00000065809 | ENST00000378470 |
| PELI2 | ENSG00000139946 | ENST00000267460 |
| RC3H1 | ENSG00000135870 | ENST00000258349 |
| AGAP1 | ENSG00000157985 | ENST00000304032 |
| SHC4 | ENSG00000185634 | ENST00000332408 |
| PARP8 | ENSG00000151883 | ENST00000503750 |
| ABAT | ENSG00000183044 | ENST00000396600 |
| CXorf24 | ENSG00000196741 | ENST00000357412 |
| PREPL | ENSG00000138078 | ENST00000541738 |
| ANO5 | ENSG00000171714 | ENST00000324559 |
| CNTFR | ENSG00000122756 | ENST00000378980 |
| RBM48 | ENSG00000127993 | ENST00000481551 |
| VEZF1 | ENSG00000136451 | ENST00000258963 |
| RFTN1 | ENSG00000131378 | ENST00000432519 |
| TMEM87B | ENSG00000153214 | ENST00000283206 |
| ARID5B | ENSG00000150347 | ENST00000279873 |
| KDM4C | ENSG00000107077 | ENST00000438023 |
| ZNF566 | ENSG00000186017 | ENST00000434377 |
| POM121L2 | ENSG00000158553 | ENST00000429945 |
| SLC30A7 | ENSG00000162695 | ENST00000370112 |
| MAFG | ENSG00000197063 | ENST00000357736 |
| MARCH7 | ENSG00000136536 | ENST00000259050 |
| UBE2I | ENSG00000103275 | ENST00000325437 |
| FNDC3B | ENSG00000075420 | ENST00000336824 |
| MBNL3 | ENSG00000076770 | ENST00000538204 |
| CHSY3 | ENSG00000198108 | ENST00000305031 |
| PEAK1 | ENSG00000173517 | ENST00000312493 |
| KLF13 | ENSG00000169926 | ENST00000307145 |
| PAPOLG | ENSG00000115421 | ENST00000412217 |
| TMEM33 | ENSG00000109133 | ENST00000504986 |
| LTN1 | ENSG00000198862 | ENST00000389194 |
| SPRED1 | ENSG00000166068 | ENST00000299084 |
| TAF3 | ENSG00000165632 | ENST00000344293 |
| LMO4 | ENSG00000143013 | ENST00000370544 |
| PPARA | ENSG00000186951 | ENST00000262735 |
| SEMA5A | ENSG00000112902 | ENST00000382496 |
| LDHC | ENSG00000166796 | ENST00000541669 |
| RFX7 | ENSG00000181827 | ENST00000423270 |
| XRN1 | ENSG00000114127 | ENST00000264951 |
| ADAM10 | ENSG00000137845 | ENST00000260408 |
| FBXO11 | ENSG00000138081 | ENST00000402508 |
| PANK3 | ENSG00000120137 | ENST00000239231 |
| ADIPOQ | ENSG00000181092 | ENST00000412955 |
| FBXO22 | ENSG00000167196 | ENST00000308275 |
| RABIF | ENSG00000183155 | ENST00000367262 |
| TAOK3 | ENSG00000135090 | ENST00000392533 |
| FBXW11 | ENSG00000072803 | ENST00000296933 |
| PARK2 | ENSG00000185345 | ENST00000366898 |
| GABARAPL2 | ENSG00000034713 | ENST00000563744 |
| DMRTA1 | ENSG00000176399 | ENST00000325870 |
| INS-IGF2 | ENSG00000129965 | ENST00000356578 |
| MSR1 | ENSG00000038945 | ENST00000350896 |
| SIX1 | ENSG00000126778 | ENST00000247182 |
| CLIP2 | ENSG00000106665 | ENST00000223398 |
| YWHAB | ENSG00000166913 | ENST00000372839 |
| CHMP2B | ENSG00000083937 | ENST00000263780 |
| OSGIN2 | ENSG00000164823 | ENST00000451899 |
| SERINC1 | ENSG00000111897 | ENST00000339697 |
| CCSER1 | ENSG00000184305 | ENST00000509176 |
| ARL15 | ENSG00000185305 | ENST00000502271 |
| SYT1 | ENSG00000067715 | ENST00000457153 |
| METTL8 | ENSG00000123600 | ENST00000375258 |
| ESM1 | ENSG00000164283 | ENST00000381405 |
| NAMPT | ENSG00000105835 | ENST00000222553 |
| ACE2 | ENSG00000130234 | ENST00000252519 |
| CDC14B | ENSG00000081377 | ENST00000412285 |
| COL4A1 | ENSG00000187498 | ENST00000375820 |
| ZFR | ENSG00000056097 | ENST00000265069 |
| FNTB | ENSG00000257365 | ENST00000246166 |
| FAM218A | ENSG00000250486 | ENST00000513876 |
| ZNF674 | ENSG00000251192 | ENST00000523374 |
| KAT2B | ENSG00000114166 | ENST00000263754 |
| FMN1 | ENSG00000248905 | ENST00000334528 |
| KCNA1 | ENSG00000111262 | ENST00000382545 |
| PLCL1 | ENSG00000115896 | ENST00000435320 |
| HTR5A-AS1 | ENSG00000220575 | ENST00000395731 |
| HS3ST3A1 | ENSG00000153976 | ENST00000284110 |
| RPL23 | ENSG00000125691 | ENST00000479035 |
| ARID4B | ENSG00000054267 | ENST00000349213 |
| PIK3C3 | ENSG00000078142 | ENST00000262039 |
| SCD | ENSG00000099194 | ENST00000370355 |
| SIRT1 | ENSG00000096717 | ENST00000406900 |
| KCNU1 | ENSG00000215262 | ENST00000522372 |
| SLC2A10 | ENSG00000197496 | ENST00000359271 |
| PITPNM3 | ENSG00000091622 | ENST00000421306 |
| ERBB4 | ENSG00000178568 | ENST00000342788 |
| ARID2 | ENSG00000189079 | ENST00000457135 |
| SCN3B | ENSG00000166257 | ENST00000299333 |
| PLGLB1 | ENSG00000183281 | ENST00000355705 |
| FMR1 | ENSG00000102081 | ENST00000370471 |
| ERI1 | ENSG00000104626 | ENST00000250263 |
| DPY19L3 | ENSG00000178904 | ENST00000586987 |
| HDAC9 | ENSG00000048052 | ENST00000406451 |
| COMMD3 | ENSG00000148444 | ENST00000448361 |
| ATP13A3 | ENSG00000133657 | ENST00000439040 |
| CUL2 | ENSG00000108094 | ENST00000374754 |
| CCDC50 | ENSG00000152492 | ENST00000392455 |
| BAG5 | ENSG00000166170 | ENST00000299204 |
| PLGLB2 | ENSG00000125551 | ENST00000359481 |
| GFPT1 | ENSG00000198380 | ENST00000357308 |
| SENP6 | ENSG00000112701 | ENST00000370014 |
| AGO3 | ENSG00000126070 | ENST00000373191 |
| B3GALTL | ENSG00000187676 | ENST00000343307 |
| KIAA1430 | ENSG00000164323 | ENST00000458385 |
| AL353698.1 | ENSG00000233889 | ENST00000433279 |
| ANKRD6 | ENSG00000135299 | ENST00000369408 |
| POU6F1 | ENSG00000184271 | ENST00000389243 |
| PJA2 | ENSG00000198961 | ENST00000361189 |
| AC011755.1 | ENSG00000269686 | ENST00000596384 |
| AGL | ENSG00000162688 | ENST00000370161 |
| MCC | ENSG00000171444 | ENST00000302475 |
| HFE | ENSG00000010704 | ENST00000357618 |
| SIAH1 | ENSG00000196470 | ENST00000356721 |
| CNTN1 | ENSG00000018236 | ENST00000551295 |
| NSUN2 | ENSG00000037474 | ENST00000504374 |
| IRX5 | ENSG00000176842 | ENST00000320990 |
| BDKRB2 | ENSG00000168398 | ENST00000306005 |
| LRRC40 | ENSG00000066557 | ENST00000370952 |
| GTDC1 | ENSG00000121964 | ENST00000392869 |
| MAP3K4 | ENSG00000085511 | ENST00000490904 |
| WDR82 | ENSG00000164091 | ENST00000296490 |
| ARHGEF10 | ENSG00000104728 | ENST00000520359 |
| CXorf23 | ENSG00000173681 | ENST00000379687 |
| THRA | ENSG00000126351 | ENST00000450525 |
| GSKIP | ENSG00000100744 | ENST00000555181 |
| PPP6C | ENSG00000119414 | ENST00000373547 |
| TRAPPC6B | ENSG00000182400 | ENST00000347691 |
| KBTBD6 | ENSG00000165572 | ENST00000379485 |
| CLEC7A | ENSG00000172243 | ENST00000353231 |
| FGF18 | ENSG00000156427 | ENST00000274625 |
| TMTC3 | ENSG00000139324 | ENST00000266712 |
| ANKRD13C | ENSG00000118454 | ENST00000370944 |
| UCHL5 | ENSG00000116750 | ENST00000367455 |
| MTFR1 | ENSG00000066855 | ENST00000518800 |
| MPV17L | ENSG00000156968 | ENST00000287594 |
| ENO4 | ENSG00000188316 | ENST00000409522 |
| HCCS | ENSG00000004961 | ENST00000321143 |
| SERF1B | ENSG00000205572 | ENST00000380750 |
| NFASC | ENSG00000163531 | ENST00000401399 |
| TMBIM4 | ENSG00000155957 | ENST00000358230 |
| PI15 | ENSG00000137558 | ENST00000260113 |
| GPRIN3 | ENSG00000185477 | ENST00000609438 |
| MTM1 | ENSG00000171100 | ENST00000370396 |
| ITGAV | ENSG00000138448 | ENST00000261023 |
| GAD2 | ENSG00000136750 | ENST00000376261 |
| ATXN1L | ENSG00000224470 | ENST00000427980 |
| GOLGA8A | ENSG00000175265 | ENST00000432566 |
| FOPNL | ENSG00000133393 | ENST00000255759 |
| HCFC2 | ENSG00000111727 | ENST00000229330 |
| NSMAF | ENSG00000035681 | ENST00000038176 |
| ZNF678 | ENSG00000181450 | ENST00000343776 |
| GPR137C | ENSG00000180998 | ENST00000321662 |
| CDC14A | ENSG00000079335 | ENST00000370125 |
| LARP1B | ENSG00000138709 | ENST00000326639 |
| PROX1 | ENSG00000117707 | ENST00000366958 |
| USP47 | ENSG00000170242 | ENST00000399455 |
| SWAP70 | ENSG00000133789 | ENST00000318950 |
| VTA1 | ENSG00000009844 | ENST00000367630 |
| SAMD12 | ENSG00000177570 | ENST00000409003 |
| RP11-383H13.1 | ENSG00000235531 | ENST00000522519 |
| FOXK1 | ENSG00000164916 | ENST00000328914 |
| CASD1 | ENSG00000127995 | ENST00000297273 |
| GPR180 | ENSG00000152749 | ENST00000376958 |
| KDM5A | ENSG00000073614 | ENST00000399788 |
| INTS7 | ENSG00000143493 | ENST00000366994 |
| TMTC1 | ENSG00000133687 | ENST00000256062 |
| ADCYAP1 | ENSG00000141433 | ENST00000579794 |
| MYOF | ENSG00000138119 | ENST00000463743 |
| ADCY9 | ENSG00000162104 | ENST00000294016 |
| BOLL | ENSG00000152430 | ENST00000321801 |
| CNTD1 | ENSG00000176563 | ENST00000588408 |
| SRSF1 | ENSG00000136450 | ENST00000582730 |
| APC | ENSG00000134982 | ENST00000508624 |
| CELF2 | ENSG00000048740 | ENST00000379261 |
| GRIN2A | ENSG00000183454 | ENST00000396573 |
| CCNA2 | ENSG00000145386 | ENST00000274026 |
| TTC9C | ENSG00000162222 | ENST00000294161 |
| FYN | ENSG00000010810 | ENST00000354650 |
| BRWD1 | ENSG00000185658 | ENST00000342449 |
| LRP4 | ENSG00000134569 | ENST00000378623 |
| MZT1 | ENSG00000204899 | ENST00000377818 |
| EIF5A2 | ENSG00000163577 | ENST00000295822 |
| BET1 | ENSG00000105829 | ENST00000357520 |
| G6PC2 | ENSG00000152254 | ENST00000282075 |
| NEO1 | ENSG00000067141 | ENST00000261908 |
| PMM1 | ENSG00000100417 | ENST00000216259 |
| SHC1 | ENSG00000160691 | ENST00000368449 |
| DDIT4 | ENSG00000168209 | ENST00000307365 |
| ZNF652 | ENSG00000198740 | ENST00000362063 |
| PPP1CB | ENSG00000213639 | ENST00000395366 |
| TMEM200C | ENSG00000206432 | ENST00000581347 |
| UBQLN1 | ENSG00000135018 | ENST00000376395 |
| DST | ENSG00000151914 | ENST00000312431 |
| MYZAP | ENSG00000263155 | ENST00000267853 |
| C16orf87 | ENSG00000155330 | ENST00000394806 |
| KBTBD8 | ENSG00000163376 | ENST00000417314 |
| ZNF148 | ENSG00000163848 | ENST00000360647 |
| PTP4A1 | ENSG00000112245 | ENST00000370651 |
| KIAA0895 | ENSG00000164542 | ENST00000297063 |
| TSPAN19 | ENSG00000231738 | ENST00000433494 |
| LGSN | ENSG00000146166 | ENST00000370658 |
| TRIML1 | ENSG00000184108 | ENST00000332517 |
| OGFRL1 | ENSG00000119900 | ENST00000370435 |
| TCF24 | ENSG00000261787 | ENST00000563496 |
| WDR72 | ENSG00000166415 | ENST00000396328 |
| LPAR5 | ENSG00000184574 | ENST00000431922 |
| ZBTB21 | ENSG00000173276 | ENST00000310826 |
| ZNF738 | ENSG00000172687 | ENST00000311015 |
| TOR1AIP2 | ENSG00000169905 | ENST00000367612 |
| SKP1 | ENSG00000113558 | ENST00000353411 |
| SLC35B3 | ENSG00000124786 | ENST00000426876 |
| GPALPP1 | ENSG00000133114 | ENST00000497558 |
| ABHD10 | ENSG00000144827 | ENST00000273359 |
| FAT4 | ENSG00000196159 | ENST00000394329 |
| PCYOX1 | ENSG00000116005 | ENST00000433351 |
| TENM3 | ENSG00000218336 | ENST00000511685 |
| VPS13A | ENSG00000197969 | ENST00000360280 |
| NT5DC1 | ENSG00000178425 | ENST00000319550 |
| SLC35E2B | ENSG00000189339 | ENST00000378662 |
| RAB37 | ENSG00000172794 | ENST00000392610 |
| KLHL42 | ENSG00000087448 | ENST00000381271 |
| TBC1D22B | ENSG00000065491 | ENST00000373491 |
| VEGFA | ENSG00000112715 | ENST00000372064 |
| ADAMTS4 | ENSG00000158859 | ENST00000367996 |
| TC2N | ENSG00000165929 | ENST00000435962 |
| GDA | ENSG00000119125 | ENST00000358399 |
| USP31 | ENSG00000103404 | ENST00000219689 |
| SHOC2 | ENSG00000108061 | ENST00000369452 |
| C1orf173 | ENSG00000178965 | ENST00000326665 |
| SERTAD4 | ENSG00000082497 | ENST00000367012 |
| VTI1A | ENSG00000151532 | ENST00000393077 |
| GOLGA8I | ENSG00000153666 | ENST00000450802 |
| PLCXD3 | ENSG00000182836 | ENST00000377801 |
| CENPQ | ENSG00000031691 | ENST00000335783 |
| DHX9 | ENSG00000135829 | ENST00000367549 |
| MAPK9 | ENSG00000050748 | ENST00000452135 |
| BMPER | ENSG00000164619 | ENST00000297161 |
| NCK1 | ENSG00000158092 | ENST00000469404 |
| PCDH7 | ENSG00000169851 | ENST00000511884 |
| CUL5 | ENSG00000166266 | ENST00000393094 |
| ARGLU1 | ENSG00000134884 | ENST00000400198 |
| TEAD1 | ENSG00000187079 | ENST00000361905 |
| GSTA4 | ENSG00000170899 | ENST00000370963 |
| ARPP21 | ENSG00000172995 | ENST00000441454 |
| ZNF516 | ENSG00000101493 | ENST00000443185 |
| FAM150B | ENSG00000189292 | ENST00000403610 |
| KATNAL1 | ENSG00000102781 | ENST00000380615 |
| KPNA4 | ENSG00000186432 | ENST00000334256 |
| ONECUT2 | ENSG00000119547 | ENST00000491143 |
| DCUN1D5 | ENSG00000137692 | ENST00000531571 |
| MCRS1 | ENSG00000187778 | ENST00000548602 |
| SOCS5 | ENSG00000171150 | ENST00000306503 |
| FGF9 | ENSG00000102678 | ENST00000382353 |
| LTBP1 | ENSG00000049323 | ENST00000404525 |
| SPTLC3 | ENSG00000172296 | ENST00000399002 |
| CCDC144A | ENSG00000170160 | ENST00000443444 |
| AMMECR1L | ENSG00000144233 | ENST00000272647 |
| ACPL2 | ENSG00000155893 | ENST00000286353 |
| CHURC1-FNTB | ENSG00000125954 | ENST00000552941 |
| GTF2H1 | ENSG00000110768 | ENST00000265963 |
| RBM24 | ENSG00000112183 | ENST00000379052 |
| ACACA | ENSG00000132142 | ENST00000353139 |
| NDUFA9 | ENSG00000139180 | ENST00000266544 |
| DDX3X | ENSG00000215301 | ENST00000399959 |
| JADE1 | ENSG00000077684 | ENST00000452328 |
| TNFRSF11B | ENSG00000164761 | ENST00000297350 |
| SLC23A2 | ENSG00000089057 | ENST00000338244 |
| KIAA1429 | ENSG00000164944 | ENST00000437199 |
| ADAMTS6 | ENSG00000049192 | ENST00000381052 |
| TCF4 | ENSG00000196628 | ENST00000354452 |
| ARL6IP6 | ENSG00000177917 | ENST00000326446 |
| ENOX2 | ENSG00000165675 | ENST00000370935 |
| EYA4 | ENSG00000112319 | ENST00000367895 |
| NCR3LG1 | ENSG00000188211 | ENST00000338965 |
| CLASP1 | ENSG00000074054 | ENST00000409078 |
| PKD2 | ENSG00000118762 | ENST00000237596 |
| RPL22L1 | ENSG00000163584 | ENST00000295830 |
| LUC7L3 | ENSG00000108848 | ENST00000505658 |
| CTAGE5 | ENSG00000150527 | ENST00000280083 |
| GAN | ENSG00000261609 | ENST00000568107 |
| PALB2 | ENSG00000083093 | ENST00000568219 |
| CCDC152 | ENSG00000198865 | ENST00000361970 |
| IGDCC4 | ENSG00000103742 | ENST00000352385 |
| PLCB4 | ENSG00000101333 | ENST00000378493 |
| NEGR1 | ENSG00000172260 | ENST00000357731 |
| PAQR3 | ENSG00000163291 | ENST00000512733 |
| METTL20 | ENSG00000139160 | ENST00000357721 |
| ITGA9 | ENSG00000144668 | ENST00000264741 |
| FER | ENSG00000151422 | ENST00000281092 |
| PPTC7 | ENSG00000196850 | ENST00000354300 |
| AHR | ENSG00000106546 | ENST00000242057 |
| CHML | ENSG00000203668 | ENST00000366553 |
| MAP3K5 | ENSG00000197442 | ENST00000359015 |
| AHRR | ENSG00000063438 | ENST00000316418 |
| LATS2 | ENSG00000150457 | ENST00000382592 |
| VKORC1L1 | ENSG00000196715 | ENST00000360768 |
| SATB2 | ENSG00000119042 | ENST00000417098 |
| ITGA1 | ENSG00000213949 | ENST00000282588 |
| GNPDA2 | ENSG00000163281 | ENST00000509756 |
| TMEM123 | ENSG00000152558 | ENST00000361236 |
| TMEM154 | ENSG00000170006 | ENST00000304385 |
| LYSMD3 | ENSG00000176018 | ENST00000500869 |
| ABCD3 | ENSG00000117528 | ENST00000370214 |
| NCKAP5 | ENSG00000176771 | ENST00000427594 |
| WDR45B | ENSG00000141580 | ENST00000572583 |
| SKOR1 | ENSG00000188779 | ENST00000341418 |
| LAMC1 | ENSG00000135862 | ENST00000258341 |
| ETS2 | ENSG00000157557 | ENST00000360214 |
| RBM8A | ENSG00000131795 | ENST00000330165 |
| SLC25A30 | ENSG00000174032 | ENST00000519676 |
| CYTH3 | ENSG00000008256 | ENST00000350796 |
| COL5A2 | ENSG00000204262 | ENST00000374866 |
| OR51E1 | ENSG00000180785 | ENST00000396952 |
| RPS23 | ENSG00000186468 | ENST00000296674 |
| SLAIN1 | ENSG00000139737 | ENST00000351546 |
| MAK | ENSG00000111837 | ENST00000354489 |
| HCN1 | ENSG00000164588 | ENST00000303230 |
| R3HDM2 | ENSG00000179912 | ENST00000347140 |
| CITED2 | ENSG00000164442 | ENST00000367651 |
| PRKG2 | ENSG00000138669 | ENST00000395578 |
| PPAPDC2 | ENSG00000205808 | ENST00000381883 |
| BCL2L11 | ENSG00000153094 | ENST00000393256 |
| LIMK1 | ENSG00000106683 | ENST00000336180 |
| SOX2 | ENSG00000181449 | ENST00000325404 |
| PKN2 | ENSG00000065243 | ENST00000370521 |
| ATP6V0A2 | ENSG00000185344 | ENST00000330342 |
| FSTL1 | ENSG00000163430 | ENST00000295633 |
| BMS1 | ENSG00000165733 | ENST00000374518 |
| DCAF5 | ENSG00000139990 | ENST00000341516 |
| MTX3 | ENSG00000177034 | ENST00000512560 |
| KCND2 | ENSG00000184408 | ENST00000331113 |
| MARCH6 | ENSG00000145495 | ENST00000274140 |
| ABCC9 | ENSG00000069431 | ENST00000261200 |
| TMEM185B | ENSG00000226479 | ENST00000426077 |
| ZRANB3 | ENSG00000121988 | ENST00000401392 |
| DCUN1D4 | ENSG00000109184 | ENST00000477560 |
| SLIT1 | ENSG00000187122 | ENST00000266058 |
| WASF1 | ENSG00000112290 | ENST00000392586 |
| UBE2D1 | ENSG00000072401 | ENST00000373910 |
| CHD2 | ENSG00000173575 | ENST00000394196 |
| ST6GALNAC5 | ENSG00000117069 | ENST00000477717 |
| STAM2 | ENSG00000115145 | ENST00000263904 |
| FXR1 | ENSG00000114416 | ENST00000357559 |
| CBWD1 | ENSG00000172785 | ENST00000382393 |
| NPIPA7 | ENSG00000183889 | ENST00000524823 |
| NPIPA5 | ENSG00000183793 | ENST00000432570 |
| FREM1 | ENSG00000164946 | ENST00000380881 |
| IMPAD1 | ENSG00000104331 | ENST00000262644 |
| FAM214A | ENSG00000047346 | ENST00000534964 |
| MGAT4A | ENSG00000071073 | ENST00000393487 |
| DDI1 | ENSG00000170967 | ENST00000302259 |
| UTY | ENSG00000183878 | ENST00000331397 |
| IFIT5 | ENSG00000152778 | ENST00000371795 |
| ALCAM | ENSG00000170017 | ENST00000306107 |
| WNK3 | ENSG00000196632 | ENST00000375169 |
| PRDX3 | ENSG00000165672 | ENST00000356951 |
| COL4A6 | ENSG00000197565 | ENST00000372216 |
| STAG1 | ENSG00000118007 | ENST00000483235 |
| FAM105B | ENSG00000154124 | ENST00000284274 |
| GAL3ST1 | ENSG00000128242 | ENST00000406955 |
| C10orf32 | ENSG00000166275 | ENST00000339834 |
| STS | ENSG00000101846 | ENST00000217961 |
| MTMR9 | ENSG00000104643 | ENST00000221086 |
| AMOTL1 | ENSG00000166025 | ENST00000433060 |
| CFHR1 | ENSG00000244414 | ENST00000320493 |
| OXGR1 | ENSG00000165621 | ENST00000298440 |
| ARPC2 | ENSG00000163466 | ENST00000414983 |
| YAP1 | ENSG00000137693 | ENST00000282441 |
| SGMS1 | ENSG00000198964 | ENST00000361781 |
| NUFIP2 | ENSG00000108256 | ENST00000225388 |
| ZBTB8B | ENSG00000273274 | ENST00000609129 |
| UGT2A3 | ENSG00000135220 | ENST00000251566 |
| SMURF2 | ENSG00000108854 | ENST00000582081 |
| SNAI2 | ENSG00000019549 | ENST00000020945 |
| CA6 | ENSG00000131686 | ENST00000480186 |
| KANK1 | ENSG00000107104 | ENST00000382286 |
| GIGYF1 | ENSG00000146830 | ENST00000275732 |
| JKAMP | ENSG00000050130 | ENST00000261247 |
| MKRN3 | ENSG00000179455 | ENST00000568252 |
| PATZ1 | ENSG00000100105 | ENST00000405309 |
| LPAR1 | ENSG00000198121 | ENST00000374431 |
| WHSC1L1 | ENSG00000147548 | ENST00000317025 |
| TGFBR3 | ENSG00000069702 | ENST00000533089 |
| SLC41A2 | ENSG00000136052 | ENST00000258538 |
| USP9X | ENSG00000124486 | ENST00000324545 |
| ZKSCAN8 | ENSG00000198315 | ENST00000330236 |
| MTR | ENSG00000116984 | ENST00000366577 |
| ZCCHC14 | ENSG00000140948 | ENST00000268616 |
| SAMD9 | ENSG00000205413 | ENST00000379958 |
| C18orf25 | ENSG00000152242 | ENST00000282059 |
| UHRF1BP1 | ENSG00000065060 | ENST00000192788 |
| DSG3 | ENSG00000134757 | ENST00000257189 |
| ZNF19 | ENSG00000157429 | ENST00000567225 |
| GIN1 | ENSG00000145723 | ENST00000512248 |
| TTC21B | ENSG00000123607 | ENST00000243344 |
| ORMDL3 | ENSG00000172057 | ENST00000579695 |
| KCTD16 | ENSG00000183775 | ENST00000507359 |
| NDUFS1 | ENSG00000023228 | ENST00000233190 |
| KCND3 | ENSG00000171385 | ENST00000369697 |
| USP15 | ENSG00000135655 | ENST00000353364 |
| KIAA1033 | ENSG00000136051 | ENST00000550053 |
| FAM227B | ENSG00000166262 | ENST00000559573 |
| MYB | ENSG00000118513 | ENST00000525514 |
| CELF1 | ENSG00000149187 | ENST00000395290 |
| ZNF521 | ENSG00000198795 | ENST00000361524 |
| ANKRD66 | ENSG00000230062 | ENST00000445060 |
| C2orf88 | ENSG00000187699 | ENST00000340623 |
| PRDM16 | ENSG00000142611 | ENST00000270722 |
| MTDH | ENSG00000147649 | ENST00000336273 |
| NUMB | ENSG00000133961 | ENST00000554546 |
| KLHDC1 | ENSG00000197776 | ENST00000359332 |
| RPRD2 | ENSG00000163125 | ENST00000401000 |
| FPGT | ENSG00000254685 | ENST00000609362 |
| FAM227A | ENSG00000184949 | ENST00000535113 |
| UFSP2 | ENSG00000109775 | ENST00000514247 |
| FBXL3 | ENSG00000005812 | ENST00000355619 |
| MEMO1 | ENSG00000162959 | ENST00000295065 |
| UGT8 | ENSG00000174607 | ENST00000310836 |
| TPCN1 | ENSG00000186815 | ENST00000335509 |
| TMEM17 | ENSG00000186889 | ENST00000335390 |
| SLCO5A1 | ENSG00000137571 | ENST00000260126 |
| CREG1 | ENSG00000143162 | ENST00000370509 |
| ACVR2A | ENSG00000121989 | ENST00000241416 |
| GOPC | ENSG00000047932 | ENST00000535237 |
| ENPEP | ENSG00000138792 | ENST00000265162 |
| PRDM1 | ENSG00000057657 | ENST00000369089 |
| PPP4R1 | ENSG00000154845 | ENST00000285124 |
| NAP1L2 | ENSG00000186462 | ENST00000373517 |
| CHRNA6 | ENSG00000147434 | ENST00000276410 |
| PTBP2 | ENSG00000117569 | ENST00000609116 |
| MED28 | ENSG00000118579 | ENST00000237380 |
| PRKAR2B | ENSG00000005249 | ENST00000265717 |
| CNOT7 | ENSG00000198791 | ENST00000361272 |
| VTI1B | ENSG00000100568 | ENST00000554659 |
| WDR7 | ENSG00000091157 | ENST00000254442 |
| SRSF10 | ENSG00000188529 | ENST00000344989 |
| FBLN5 | ENSG00000140092 | ENST00000267620 |
| MFHAS1 | ENSG00000147324 | ENST00000276282 |
| NR3C1 | ENSG00000113580 | ENST00000343796 |
| ITPR1 | ENSG00000150995 | ENST00000423119 |
| ERGIC2 | ENSG00000087502 | ENST00000360150 |
| PSMD12 | ENSG00000197170 | ENST00000356126 |
| SH3RF2 | ENSG00000156463 | ENST00000511217 |
| SYCP2L | ENSG00000153157 | ENST00000341041 |
| SASH1 | ENSG00000111961 | ENST00000367467 |
| CASKIN1 | ENSG00000167971 | ENST00000343516 |
| NEUROD1 | ENSG00000162992 | ENST00000295108 |
| KHDRBS1 | ENSG00000121774 | ENST00000327300 |
| UNC119B | ENSG00000175970 | ENST00000344651 |
| CDH19 | ENSG00000071991 | ENST00000262150 |
| FAM135A | ENSG00000082269 | ENST00000194672 |
| STOX2 | ENSG00000173320 | ENST00000308497 |
| ATP2B1 | ENSG00000070961 | ENST00000359142 |
| TTC14 | ENSG00000163728 | ENST00000465065 |
| PDE4B | ENSG00000184588 | ENST00000371045 |
| SLC2A13 | ENSG00000151229 | ENST00000280871 |
| ARHGAP5 | ENSG00000100852 | ENST00000432921 |
| RORA | ENSG00000069667 | ENST00000335670 |
| GPR173 | ENSG00000184194 | ENST00000332582 |
| CA2 | ENSG00000104267 | ENST00000285379 |
| SENP5 | ENSG00000119231 | ENST00000323460 |
| TMEM57 | ENSG00000204178 | ENST00000374343 |
| EIF5B | ENSG00000158417 | ENST00000289371 |
| C4orf27 | ENSG00000056050 | ENST00000393381 |
| NHLRC2 | ENSG00000196865 | ENST00000369301 |
| RHOT1 | ENSG00000126858 | ENST00000578205 |
| TMEM161B | ENSG00000164180 | ENST00000510089 |
| BRCA2 | ENSG00000139618 | ENST00000544455 |
| SBNO1 | ENSG00000139697 | ENST00000267176 |
| FRAS1 | ENSG00000138759 | ENST00000512123 |
| ZNF280B | ENSG00000198477 | ENST00000360412 |
| MCMDC2 | ENSG00000178460 | ENST00000422365 |
| LANCL1 | ENSG00000115365 | ENST00000450366 |
| ERBB2IP | ENSG00000112851 | ENST00000284037 |
| SEPT7 | ENSG00000122545 | ENST00000494488 |
| BCL9 | ENSG00000116128 | ENST00000234739 |
| ATP2A2 | ENSG00000174437 | ENST00000377685 |
| SEMA6D | ENSG00000137872 | ENST00000355997 |
| GNPDA1 | ENSG00000113552 | ENST00000311337 |
| TRIO | ENSG00000038382 | ENST00000512070 |
| PTGER3 | ENSG00000050628 | ENST00000370924 |
| EMCN | ENSG00000164035 | ENST00000296420 |
| TTC39B | ENSG00000155158 | ENST00000512701 |
| RP2 | ENSG00000102218 | ENST00000218340 |
| TMEM196 | ENSG00000173452 | ENST00000405844 |
| XKR6 | ENSG00000171044 | ENST00000382461 |
| RFPL3 | ENSG00000128276 | ENST00000249007 |
| SCN9A | ENSG00000169432 | ENST00000409672 |
| SLC24A4 | ENSG00000140090 | ENST00000525557 |
| SORCS3 | ENSG00000156395 | ENST00000369701 |
| TAF4 | ENSG00000130699 | ENST00000252996 |
| TNPO1 | ENSG00000083312 | ENST00000337273 |
| PI4K2B | ENSG00000038210 | ENST00000264864 |
| SOCS4 | ENSG00000180008 | ENST00000395472 |
| DUSP11 | ENSG00000144048 | ENST00000443070 |
| FBXL17 | ENSG00000145743 | ENST00000359660 |
| CDC42 | ENSG00000070831 | ENST00000344548 |
| FZD3 | ENSG00000104290 | ENST00000240093 |
| MTMR12 | ENSG00000150712 | ENST00000280285 |
| TJP1 | ENSG00000104067 | ENST00000346128 |
| NAA30 | ENSG00000139977 | ENST00000556492 |
| RAB4A | ENSG00000168118 | ENST00000366690 |
| RPF2 | ENSG00000197498 | ENST00000607388 |
| SOX15 | ENSG00000129194 | ENST00000250055 |
| TEX9 | ENSG00000151575 | ENST00000558083 |
| ANO6 | ENSG00000177119 | ENST00000441606 |
| PRKACB | ENSG00000142875 | ENST00000394839 |
| DDI2 | ENSG00000197312 | ENST00000480945 |
| HIVEP3 | ENSG00000127124 | ENST00000372584 |
| NCAM1 | ENSG00000149294 | ENST00000316851 |
| ZNF326 | ENSG00000162664 | ENST00000370447 |
| MTAP | ENSG00000099810 | ENST00000380172 |
| PAQR5 | ENSG00000137819 | ENST00000395407 |
| PPP1R9B | ENSG00000108819 | ENST00000316878 |
| IKZF2 | ENSG00000030419 | ENST00000457361 |
| SNX29 | ENSG00000048471 | ENST00000566228 |
| LCORL | ENSG00000178177 | ENST00000326877 |
| ASXL3 | ENSG00000141431 | ENST00000269197 |
| C14orf37 | ENSG00000139971 | ENST00000267485 |
| ARHGAP19-SLIT1 | ENSG00000269891 | ENST00000453547 |
| MAMLD1 | ENSG00000013619 | ENST00000426613 |
| ERG | ENSG00000157554 | ENST00000417133 |
| C1orf50 | ENSG00000164008 | ENST00000464081 |
| SMURF1 | ENSG00000198742 | ENST00000361368 |
| NUDT3 | ENSG00000272325 | ENST00000607016 |
| FAM161A | ENSG00000170264 | ENST00000456262 |
| C2orf69 | ENSG00000178074 | ENST00000319974 |
| ZNF850 | ENSG00000267041 | ENST00000591344 |
| NUDT21 | ENSG00000167005 | ENST00000300291 |
| TMEM170A | ENSG00000166822 | ENST00000357613 |
| PPM1B | ENSG00000138032 | ENST00000378551 |
| DDX55 | ENSG00000111364 | ENST00000238146 |
| PCNP | ENSG00000081154 | ENST00000460231 |
| RFPL2 | ENSG00000128253 | ENST00000248980 |
| PLCB1 | ENSG00000182621 | ENST00000378641 |
| USP18 | ENSG00000184979 | ENST00000215794 |
| DEFB132 | ENSG00000186458 | ENST00000382376 |
| CNOT8 | ENSG00000155508 | ENST00000285896 |
| TTF1 | ENSG00000125482 | ENST00000334270 |
| ZXDA | ENSG00000198205 | ENST00000358697 |
| DENND4C | ENSG00000137145 | ENST00000494124 |
| C5orf24 | ENSG00000181904 | ENST00000394976 |
| SP3 | ENSG00000172845 | ENST00000455789 |
| SULF2 | ENSG00000196562 | ENST00000359930 |
| LY75 | ENSG00000054219 | ENST00000263636 |
| WBP4 | ENSG00000120688 | ENST00000379487 |
| APOOL | ENSG00000155008 | ENST00000373173 |
| SEC63 | ENSG00000025796 | ENST00000369002 |
| PAWR | ENSG00000177425 | ENST00000328827 |
| NEK10 | ENSG00000163491 | ENST00000295720 |
| ZNF550 | ENSG00000251369 | ENST00000447310 |
| NKX3-2 | ENSG00000109705 | ENST00000382438 |
| TSSK1B | ENSG00000212122 | ENST00000390666 |
| HNRNPU | ENSG00000153187 | ENST00000444376 |
| SFTPA1 | ENSG00000122852 | ENST00000398636 |
| SPRTN | ENSG00000010072 | ENST00000295050 |
| TPBG | ENSG00000146242 | ENST00000369750 |
| KIDINS220 | ENSG00000134313 | ENST00000488729 |
| TPR | ENSG00000047410 | ENST00000367478 |
| MPZL3 | ENSG00000160588 | ENST00000278949 |
| KCNJ15 | ENSG00000157551 | ENST00000328656 |
| HGF | ENSG00000019991 | ENST00000222390 |
| TBX22 | ENSG00000122145 | ENST00000373291 |
| REEP3 | ENSG00000165476 | ENST00000373758 |
| ZNF692 | ENSG00000171163 | ENST00000463519 |
| TOMM70A | ENSG00000154174 | ENST00000284320 |
| B3GNT2 | ENSG00000170340 | ENST00000301998 |
| WWC3 | ENSG00000047644 | ENST00000380861 |
| EXOC6 | ENSG00000138190 | ENST00000371543 |
| GLIS2 | ENSG00000126603 | ENST00000262366 |
| SH3KBP1 | ENSG00000147010 | ENST00000397821 |
| ZC3H13 | ENSG00000123200 | ENST00000242848 |
| SORL1 | ENSG00000137642 | ENST00000260197 |
| SPAST | ENSG00000021574 | ENST00000315285 |
| RFWD2 | ENSG00000143207 | ENST00000367667 |
| SLC39A14 | ENSG00000104635 | ENST00000381237 |
| ESCO2 | ENSG00000171320 | ENST00000305188 |
| COPS3 | ENSG00000141030 | ENST00000578317 |
| SLC1A1 | ENSG00000106688 | ENST00000262352 |
| HMOX1 | ENSG00000100292 | ENST00000216117 |
| IER5 | ENSG00000162783 | ENST00000367577 |
| TUBA1C | ENSG00000167553 | ENST00000552448 |
| GLCCI1 | ENSG00000106415 | ENST00000223145 |
| FAM26E | ENSG00000178033 | ENST00000368599 |
| RANBP6 | ENSG00000137040 | ENST00000259569 |
| BBX | ENSG00000114439 | ENST00000415149 |
| STK17B | ENSG00000081320 | ENST00000263955 |
| TMEM14A | ENSG00000096092 | ENST00000211314 |
| SEC31A | ENSG00000138674 | ENST00000508479 |
| POU2F1 | ENSG00000143190 | ENST00000271411 |
| ETS1 | ENSG00000134954 | ENST00000535549 |
| DERL1 | ENSG00000136986 | ENST00000259512 |
| ZNF780A | ENSG00000197782 | ENST00000450241 |
| TWISTNB | ENSG00000105849 | ENST00000222567 |
| TMED2 | ENSG00000086598 | ENST00000262225 |
| ING5 | ENSG00000168395 | ENST00000313552 |
| CALN1 | ENSG00000183166 | ENST00000329008 |
| PAXIP1 | ENSG00000157212 | ENST00000457196 |
| CCDC85A | ENSG00000055813 | ENST00000407595 |
| RFPL1 | ENSG00000128250 | ENST00000354373 |
| EIF3J | ENSG00000104131 | ENST00000261868 |
| HSF5 | ENSG00000176160 | ENST00000323777 |
| ZNF770 | ENSG00000198146 | ENST00000356321 |
| TMEM189-UBE2V1 | ENSG00000124208 | ENST00000341698 |
| BACH2 | ENSG00000112182 | ENST00000537989 |
| BAG4 | ENSG00000156735 | ENST00000287322 |
| C2orf49 | ENSG00000135974 | ENST00000258457 |
| IFT81 | ENSG00000122970 | ENST00000361948 |
| USP25 | ENSG00000155313 | ENST00000400183 |
| SLC30A5 | ENSG00000145740 | ENST00000396591 |
| FGD4 | ENSG00000139132 | ENST00000427716 |
| LBR | ENSG00000143815 | ENST00000272163 |
| PIGN | ENSG00000197563 | ENST00000357637 |
| NPY2R | ENSG00000185149 | ENST00000506608 |
| GTF2H3 | ENSG00000111358 | ENST00000543341 |
| CAPZA2 | ENSG00000198898 | ENST00000361183 |
| TRIM23 | ENSG00000113595 | ENST00000231524 |
| PRPF3 | ENSG00000117360 | ENST00000543398 |
| TTC3 | ENSG00000182670 | ENST00000399017 |
| SYNRG | ENSG00000006114 | ENST00000339208 |
| CROT | ENSG00000005469 | ENST00000331536 |
| PAK3 | ENSG00000077264 | ENST00000372007 |
| BBS12 | ENSG00000181004 | ENST00000314218 |
| SAV1 | ENSG00000151748 | ENST00000555720 |
| TXNL1 | ENSG00000091164 | ENST00000217515 |
| CREBZF | ENSG00000137504 | ENST00000490820 |
| UBE2V1 | ENSG00000244687 | ENST00000371677 |
| TRHDE | ENSG00000072657 | ENST00000261180 |
| ACTR8 | ENSG00000113812 | ENST00000335754 |
| ZFAND5 | ENSG00000107372 | ENST00000237937 |
| DCDC1 | ENSG00000170959 | ENST00000303697 |
| CARM1 | ENSG00000142453 | ENST00000586221 |
| RPAP2 | ENSG00000122484 | ENST00000610020 |
| NAA15 | ENSG00000164134 | ENST00000296543 |
| CDC42EP3 | ENSG00000163171 | ENST00000295324 |
| ATF7IP2 | ENSG00000166669 | ENST00000568027 |
| RNF170 | ENSG00000120925 | ENST00000319073 |
| GPC5 | ENSG00000179399 | ENST00000377067 |
| NGEF | ENSG00000066248 | ENST00000264051 |
| SLC16A10 | ENSG00000112394 | ENST00000368850 |
| CEP120 | ENSG00000168944 | ENST00000508138 |
| RNF39 | ENSG00000204618 | ENST00000244360 |
| SLC35F1 | ENSG00000196376 | ENST00000360388 |
| FAM126B | ENSG00000155744 | ENST00000418596 |
| RP11-17M16.1 | ENSG00000220032 | ENST00000532511 |
| CCER1 | ENSG00000197651 | ENST00000358859 |
| GATA6 | ENSG00000141448 | ENST00000269216 |
| TBX4 | ENSG00000121075 | ENST00000393853 |
| CLCC1 | ENSG00000121940 | ENST00000369969 |
| WIPF3 | ENSG00000122574 | ENST00000409290 |
| KCNQ4 | ENSG00000117013 | ENST00000347132 |
| GFI1 | ENSG00000162676 | ENST00000427103 |
| ZC3H11A | ENSG00000058673 | ENST00000495527 |
| PGAP1 | ENSG00000197121 | ENST00000423035 |
| SSX2IP | ENSG00000117155 | ENST00000342203 |
| RABGAP1L | ENSG00000152061 | ENST00000489615 |
| ADAM12 | ENSG00000148848 | ENST00000368679 |
| KIAA1841 | ENSG00000162929 | ENST00000453186 |
| RNMT | ENSG00000101654 | ENST00000383314 |
| IREB2 | ENSG00000136381 | ENST00000258886 |
| GTF3C4 | ENSG00000125484 | ENST00000372146 |
| ZNF568 | ENSG00000198453 | ENST00000415168 |
| QSER1 | ENSG00000060749 | ENST00000399302 |
| CLN8 | ENSG00000182372 | ENST00000331222 |
| IL8 | ENSG00000169429 | ENST00000307407 |
| CALHM1 | ENSG00000185933 | ENST00000329905 |
| MAP9 | ENSG00000164114 | ENST00000311277 |
| ASPH | ENSG00000198363 | ENST00000541428 |
| SOWAHC | ENSG00000198142 | ENST00000356454 |
| TNFSF4 | ENSG00000117586 | ENST00000367718 |
| PISD | ENSG00000241878 | ENST00000437808 |
| TFPI | ENSG00000003436 | ENST00000392365 |
| INO80C | ENSG00000153391 | ENST00000592173 |
| SHISA2 | ENSG00000180730 | ENST00000319420 |
| CTSC | ENSG00000109861 | ENST00000524463 |
| CHD7 | ENSG00000171316 | ENST00000525508 |
| LPIN2 | ENSG00000101577 | ENST00000261596 |
| SEMA3F | ENSG00000001617 | ENST00000002829 |
| IRF4 | ENSG00000137265 | ENST00000380956 |
| NCK2 | ENSG00000071051 | ENST00000451463 |
| RALB | ENSG00000144118 | ENST00000272519 |
| ERN1 | ENSG00000178607 | ENST00000433197 |
| RBMXL2 | ENSG00000170748 | ENST00000306904 |
| ZNF200 | ENSG00000010539 | ENST00000414144 |
| CCNT1 | ENSG00000129315 | ENST00000261900 |
| LPIN1 | ENSG00000134324 | ENST00000256720 |
| HSDL1 | ENSG00000103160 | ENST00000219439 |
| RAB18 | ENSG00000099246 | ENST00000356940 |
| GPR158 | ENSG00000151025 | ENST00000376351 |
| BHLHE41 | ENSG00000123095 | ENST00000242728 |
| CLDN22 | ENSG00000177300 | ENST00000323319 |
| CNTNAP2 | ENSG00000174469 | ENST00000361727 |
| ZDHHC15 | ENSG00000102383 | ENST00000373367 |
| CNOT6 | ENSG00000113300 | ENST00000393356 |
| SEC24A | ENSG00000113615 | ENST00000398844 |
| SNX1 | ENSG00000028528 | ENST00000380285 |
| TTBK2 | ENSG00000128881 | ENST00000267890 |
| RBM47 | ENSG00000163694 | ENST00000510871 |
| PPFIBP1 | ENSG00000110841 | ENST00000228425 |
| MATN3 | ENSG00000132031 | ENST00000407540 |
| CDK19 | ENSG00000155111 | ENST00000368911 |
| ANGPTL1 | ENSG00000116194 | ENST00000234816 |
| NALCN | ENSG00000102452 | ENST00000251127 |
| WDR47 | ENSG00000085433 | ENST00000369962 |
| ALG8 | ENSG00000159063 | ENST00000526737 |
| MAPK1IP1L | ENSG00000168175 | ENST00000395468 |
| FAM160B1 | ENSG00000151553 | ENST00000369248 |
| MXD4 | ENSG00000123933 | ENST00000337190 |
| TCERG1 | ENSG00000113649 | ENST00000296702 |
| SOCS6 | ENSG00000170677 | ENST00000397942 |
| LRIG2 | ENSG00000198799 | ENST00000361127 |
| MTO1 | ENSG00000135297 | ENST00000498286 |
| MEX3C | ENSG00000176624 | ENST00000592416 |
| MAN1A2 | ENSG00000198162 | ENST00000356554 |
| TULP4 | ENSG00000130338 | ENST00000367097 |
| TMTC2 | ENSG00000179104 | ENST00000546590 |
| SOS2 | ENSG00000100485 | ENST00000216373 |
| KMT2E | ENSG00000005483 | ENST00000334884 |
| HPSE | ENSG00000173083 | ENST00000405413 |
| SAMD5 | ENSG00000203727 | ENST00000367474 |
| ZNF621 | ENSG00000172888 | ENST00000339296 |
| ELOVL5 | ENSG00000012660 | ENST00000370918 |
| RSF1 | ENSG00000048649 | ENST00000308488 |
| USH1G | ENSG00000182040 | ENST00000319642 |
| STRADB | ENSG00000082146 | ENST00000392249 |
| RLF | ENSG00000117000 | ENST00000372771 |
| RAB7A | ENSG00000075785 | ENST00000265062 |
| CLMN | ENSG00000165959 | ENST00000298912 |
| IGSF3 | ENSG00000143061 | ENST00000369483 |
| SLC19A3 | ENSG00000135917 | ENST00000425817 |
| C1orf52 | ENSG00000162642 | ENST00000471115 |
| EIF1AX | ENSG00000173674 | ENST00000379607 |
| SLC5A12 | ENSG00000148942 | ENST00000396005 |
| VPS8 | ENSG00000156931 | ENST00000424463 |
| ITM2B | ENSG00000136156 | ENST00000378565 |
| DYRK2 | ENSG00000127334 | ENST00000344096 |
| EEA1 | ENSG00000102189 | ENST00000322349 |
| TRMT11 | ENSG00000066651 | ENST00000479748 |
| ANKRD18B | ENSG00000230453 | ENST00000357927 |
| BBS2 | ENSG00000125124 | ENST00000562012 |
| MRS2 | ENSG00000124532 | ENST00000274747 |
| MARVELD3 | ENSG00000140832 | ENST00000268485 |
| TRMT5 | ENSG00000126814 | ENST00000261249 |
| FGD6 | ENSG00000180263 | ENST00000343958 |
| C19orf69 | ENSG00000204978 | ENST00000378187 |
| CTNND2 | ENSG00000169862 | ENST00000504499 |
| ZNF101 | ENSG00000181896 | ENST00000592502 |
| C5orf30 | ENSG00000181751 | ENST00000319933 |
| TMEM245 | ENSG00000106771 | ENST00000374586 |
| CDR2L | ENSG00000109089 | ENST00000337231 |
| C6orf62 | ENSG00000112308 | ENST00000378119 |
| PPP3R1 | ENSG00000221823 | ENST00000234310 |
| PIGK | ENSG00000142892 | ENST00000445065 |
| HIBCH | ENSG00000198130 | ENST00000392332 |
| FRMD8 | ENSG00000126391 | ENST00000317568 |
| ERI2 | ENSG00000196678 | ENST00000568805 |
| PPP3CA | ENSG00000138814 | ENST00000512215 |
| FAM126A | ENSG00000122591 | ENST00000440481 |
| NTNG1 | ENSG00000162631 | ENST00000370067 |
| AKAP11 | ENSG00000023516 | ENST00000025301 |
| HLTF | ENSG00000071794 | ENST00000310053 |
| ALG2 | ENSG00000119523 | ENST00000238477 |
| CHAC2 | ENSG00000143942 | ENST00000295304 |
| PNRC2 | ENSG00000189266 | ENST00000334351 |
| RNF186 | ENSG00000178828 | ENST00000375121 |
| THAP5 | ENSG00000177683 | ENST00000415914 |
| KCNA6 | ENSG00000151079 | ENST00000433855 |
| ZNF677 | ENSG00000197928 | ENST00000333952 |
| TBK1 | ENSG00000183735 | ENST00000331710 |
| VASH1 | ENSG00000071246 | ENST00000167106 |
| SRSF12 | ENSG00000154548 | ENST00000452027 |
| EPB41L4B | ENSG00000095203 | ENST00000374566 |
| GRIA4 | ENSG00000152578 | ENST00000393127 |
| PIK3C2A | ENSG00000011405 | ENST00000265970 |
| C15orf60 | ENSG00000183324 | ENST00000331090 |
| RDH10 | ENSG00000121039 | ENST00000240285 |
| CDH26 | ENSG00000124215 | ENST00000350849 |
| MBTPS2 | ENSG00000012174 | ENST00000365779 |
| PPP1R2 | ENSG00000184203 | ENST00000328432 |
| VCPIP1 | ENSG00000175073 | ENST00000310421 |
| SKA2 | ENSG00000182628 | ENST00000330137 |
| FGF7 | ENSG00000140285 | ENST00000267843 |
| KRT222 | ENSG00000213424 | ENST00000394049 |
| PLEKHH1 | ENSG00000054690 | ENST00000329153 |
| SSR1 | ENSG00000124783 | ENST00000244763 |
| CBR4 | ENSG00000145439 | ENST00000306193 |
| PTPN13 | ENSG00000163629 | ENST00000436978 |
| PPARGC1B | ENSG00000155846 | ENST00000309241 |
| CLLU1 | ENSG00000257127 | ENST00000378485 |
| RNF11 | ENSG00000123091 | ENST00000242719 |
| PRRG4 | ENSG00000135378 | ENST00000257836 |
| SENP7 | ENSG00000138468 | ENST00000394095 |
| ANP32B | ENSG00000136938 | ENST00000339399 |
| CPM | ENSG00000135678 | ENST00000551568 |
| ACADSB | ENSG00000196177 | ENST00000358776 |
| STK17A | ENSG00000164543 | ENST00000319357 |
| RLIM | ENSG00000131263 | ENST00000332687 |
| ANKRD34B | ENSG00000189127 | ENST00000338682 |
| TOX | ENSG00000198846 | ENST00000361421 |
| ZFP28 | ENSG00000196867 | ENST00000591844 |
| PHEX | ENSG00000102174 | ENST00000379374 |
| KDM4A | ENSG00000066135 | ENST00000372396 |
| CD38 | ENSG00000004468 | ENST00000226279 |
| RBBP4 | ENSG00000162521 | ENST00000373493 |
| COL6A6 | ENSG00000206384 | ENST00000506143 |
| WDR12 | ENSG00000138442 | ENST00000261015 |
| GTF2A1 | ENSG00000165417 | ENST00000553612 |
| CMTR2 | ENSG00000180917 | ENST00000338099 |
| SULT1B1 | ENSG00000173597 | ENST00000310613 |
| ZNF507 | ENSG00000168813 | ENST00000311921 |
| KIAA1468 | ENSG00000134444 | ENST00000398130 |
| YES1 | ENSG00000176105 | ENST00000584307 |
| DARS | ENSG00000115866 | ENST00000264161 |
| TNRC6B | ENSG00000100354 | ENST00000335727 |
| SELK | ENSG00000113811 | ENST00000495461 |
| ZFP36 | ENSG00000128016 | ENST00000597629 |
| RAB8B | ENSG00000166128 | ENST00000321437 |
| CASP2 | ENSG00000106144 | ENST00000310447 |
| SCN3A | ENSG00000153253 | ENST00000360093 |
| RAB30 | ENSG00000137502 | ENST00000533486 |
| CTBP2 | ENSG00000175029 | ENST00000337195 |
| OTUD7B | ENSG00000163113 | ENST00000369135 |
| CACNB4 | ENSG00000182389 | ENST00000539935 |
| C4orf32 | ENSG00000174749 | ENST00000309733 |
| NABP1 | ENSG00000173559 | ENST00000410026 |
| ABHD17B | ENSG00000107362 | ENST00000333421 |
| MGAT3 | ENSG00000128268 | ENST00000341184 |
| CD274 | ENSG00000120217 | ENST00000381573 |
| PEX2 | ENSG00000164751 | ENST00000419564 |
| TRPM3 | ENSG00000083067 | ENST00000377110 |
| C12orf39 | ENSG00000134548 | ENST00000256969 |
| SUB1 | ENSG00000113387 | ENST00000265073 |
| PLOD2 | ENSG00000152952 | ENST00000282903 |
| ING3 | ENSG00000071243 | ENST00000315870 |
| ELK3 | ENSG00000111145 | ENST00000228741 |
| SREK1IP1 | ENSG00000153006 | ENST00000513458 |
| MOB1B | ENSG00000173542 | ENST00000309395 |
| RNF217 | ENSG00000146373 | ENST00000519565 |
| SPICE1 | ENSG00000163611 | ENST00000295872 |
| FAM96A | ENSG00000166797 | ENST00000557835 |
| DENND2C | ENSG00000175984 | ENST00000393276 |
| HIPK2 | ENSG00000064393 | ENST00000406875 |
| RD3L | ENSG00000227729 | ENST00000557640 |
| CEP97 | ENSG00000182504 | ENST00000341893 |
| STON1 | ENSG00000243244 | ENST00000309835 |
| ZNF181 | ENSG00000197841 | ENST00000392232 |
| NSL1 | ENSG00000117697 | ENST00000366977 |
| RAB27A | ENSG00000069974 | ENST00000396307 |
| FAM217B | ENSG00000196227 | ENST00000360816 |
| RNF38 | ENSG00000137075 | ENST00000353739 |
| SGIP1 | ENSG00000118473 | ENST00000371036 |
| TFEC | ENSG00000105967 | ENST00000265440 |
| TRPS1 | ENSG00000104447 | ENST00000395715 |
| UBR1 | ENSG00000159459 | ENST00000382177 |
| C15orf61 | ENSG00000189227 | ENST00000342683 |
| SMARCA5 | ENSG00000153147 | ENST00000283131 |
| ELTD1 | ENSG00000162618 | ENST00000370742 |
| USP6 | ENSG00000129204 | ENST00000575709 |
| PIK3CB | ENSG00000051382 | ENST00000462898 |
| SPEF2 | ENSG00000152582 | ENST00000506526 |
| REL | ENSG00000162924 | ENST00000295025 |
| GPR85 | ENSG00000164604 | ENST00000297146 |
| MTMR14 | ENSG00000163719 | ENST00000414996 |
| SLAMF8 | ENSG00000158714 | ENST00000289707 |
| RNF13 | ENSG00000082996 | ENST00000392894 |
| MRPS35 | ENSG00000061794 | ENST00000538315 |
| ZNF557 | ENSG00000130544 | ENST00000414706 |
| SRSF2 | ENSG00000161547 | ENST00000392485 |
| SUPT4H1 | ENSG00000213246 | ENST00000581166 |
| NRP2 | ENSG00000118257 | ENST00000360409 |
| LACE1 | ENSG00000135537 | ENST00000368977 |
| SPAG17 | ENSG00000155761 | ENST00000437255 |
| RHOA | ENSG00000067560 | ENST00000418115 |
| RAP2A | ENSG00000125249 | ENST00000245304 |
| POU4F2 | ENSG00000151615 | ENST00000281321 |
| FAM49B | ENSG00000153310 | ENST00000519824 |
| NUP160 | ENSG00000030066 | ENST00000378460 |
| ETNK1 | ENSG00000139163 | ENST00000266517 |
| TRANK1 | ENSG00000168016 | ENST00000301807 |
| TRA2B | ENSG00000136527 | ENST00000453386 |
| PKD1L1 | ENSG00000158683 | ENST00000289672 |
| ARHGDIA | ENSG00000141522 | ENST00000269321 |
| PPIG | ENSG00000138398 | ENST00000260970 |
| C9orf41 | ENSG00000156017 | ENST00000376834 |
| CHD9 | ENSG00000177200 | ENST00000566029 |
| GRB10 | ENSG00000106070 | ENST00000335866 |
| FETUB | ENSG00000090512 | ENST00000420570 |
| LIMA1 | ENSG00000050405 | ENST00000552491 |
| NKRF | ENSG00000186416 | ENST00000304449 |
| ISOC1 | ENSG00000066583 | ENST00000173527 |
| TMEM213 | ENSG00000214128 | ENST00000442682 |
| MCHR2 | ENSG00000152034 | ENST00000281806 |
| FRRS1L | ENSG00000260230 | ENST00000561981 |
| UFL1 | ENSG00000014123 | ENST00000369278 |
| RBBP9 | ENSG00000089050 | ENST00000337227 |
| ATRX | ENSG00000085224 | ENST00000373344 |
| RP11-553A10.1 | ENSG00000206532 | ENST00000485473 |
| HRSP12 | ENSG00000132541 | ENST00000522791 |
| CCNT2 | ENSG00000082258 | ENST00000264157 |
| CHD6 | ENSG00000124177 | ENST00000373233 |
| PMP22 | ENSG00000109099 | ENST00000395938 |
| LACTB2 | ENSG00000147592 | ENST00000276590 |
| REPS2 | ENSG00000169891 | ENST00000357277 |
| KCTD12 | ENSG00000178695 | ENST00000377474 |
| OPA1 | ENSG00000198836 | ENST00000392438 |
| PHLDB1 | ENSG00000019144 | ENST00000525698 |
| CLDND1 | ENSG00000080822 | ENST00000341181 |
| SCN2A | ENSG00000136531 | ENST00000375437 |
| UTP3 | ENSG00000132467 | ENST00000254803 |
| PRKAG2 | ENSG00000106617 | ENST00000287878 |
| CD59 | ENSG00000085063 | ENST00000533403 |
| WDFY3 | ENSG00000163625 | ENST00000322366 |
| SLC2A14 | ENSG00000173262 | ENST00000340749 |
| CHST9 | ENSG00000154080 | ENST00000580774 |
| CPT1A | ENSG00000110090 | ENST00000265641 |
| SHROOM1 | ENSG00000164403 | ENST00000378679 |
| NYAP1 | ENSG00000166924 | ENST00000423930 |
| B3GALNT1 | ENSG00000169255 | ENST00000392780 |
| GRM5 | ENSG00000168959 | ENST00000455756 |
| DCT | ENSG00000080166 | ENST00000377028 |
| NAP1L1 | ENSG00000187109 | ENST00000261182 |
| HDAC2 | ENSG00000196591 | ENST00000519065 |
| NCOA7 | ENSG00000111912 | ENST00000444128 |
| SGCB | ENSG00000163069 | ENST00000381431 |
| ARHGAP26 | ENSG00000145819 | ENST00000378004 |
| KLHL9 | ENSG00000198642 | ENST00000359039 |
| GABARAPL1 | ENSG00000139112 | ENST00000541960 |
| TMEM207 | ENSG00000198398 | ENST00000354905 |
| METTL10 | ENSG00000203791 | ENST00000466270 |
| LRRC8A | ENSG00000136802 | ENST00000372600 |
| EVI2B | ENSG00000185862 | ENST00000330927 |
| TSPAN12 | ENSG00000106025 | ENST00000222747 |
| KIAA1524 | ENSG00000163507 | ENST00000481530 |
| ZNF300 | ENSG00000145908 | ENST00000427179 |
| UTP23 | ENSG00000147679 | ENST00000309822 |
| STX7 | ENSG00000079950 | ENST00000367941 |
| GRIN2B | ENSG00000273079 | ENST00000609686 |
| TBC1D15 | ENSG00000121749 | ENST00000550746 |
| NADK2 | ENSG00000152620 | ENST00000397338 |
| IFIT2 | ENSG00000119922 | ENST00000371826 |
| FITM2 | ENSG00000197296 | ENST00000396825 |
| IL17F | ENSG00000112116 | ENST00000336123 |
| FGF23 | ENSG00000118972 | ENST00000237837 |
| GPR98 | ENSG00000164199 | ENST00000504142 |
| DMXL2 | ENSG00000104093 | ENST00000251076 |
| PTEN | ENSG00000171862 | ENST00000371953 |
| ZNF260 | ENSG00000254004 | ENST00000523638 |
| SLC30A4 | ENSG00000104154 | ENST00000261867 |
| LFNG | ENSG00000106003 | ENST00000402045 |
| TRAT1 | ENSG00000163519 | ENST00000295756 |
| ENAH | ENSG00000154380 | ENST00000366844 |
| CTTNBP2 | ENSG00000077063 | ENST00000441556 |
| FAM178A | ENSG00000119906 | ENST00000238961 |
| AAK1 | ENSG00000115977 | ENST00000409085 |
| ACSL4 | ENSG00000068366 | ENST00000348502 |
| CNR1 | ENSG00000118432 | ENST00000537554 |
| DACT1 | ENSG00000165617 | ENST00000395153 |
| ANKRD44 | ENSG00000065413 | ENST00000328737 |
| MAP3K7 | ENSG00000135341 | ENST00000369325 |
| GOLT1B | ENSG00000111711 | ENST00000229314 |
| TBCK | ENSG00000145348 | ENST00000273980 |
| TMOD1 | ENSG00000136842 | ENST00000259365 |
| NABP2 | ENSG00000139579 | ENST00000267023 |
| TBC1D32 | ENSG00000146350 | ENST00000464622 |
| ZNF267 | ENSG00000185947 | ENST00000300870 |
| SRSF3 | ENSG00000112081 | ENST00000373715 |
| FLRT3 | ENSG00000125848 | ENST00000378053 |
| STAT1 | ENSG00000115415 | ENST00000361099 |
| SCP2 | ENSG00000116171 | ENST00000478631 |
| TSNAX | ENSG00000116918 | ENST00000366639 |
| HACE1 | ENSG00000085382 | ENST00000416605 |
| CUTC | ENSG00000119929 | ENST00000370476 |
| ZNF771 | ENSG00000179965 | ENST00000319296 |
| TAF9 | ENSG00000085231 | ENST00000380822 |
| AQP4 | ENSG00000171885 | ENST00000383168 |
| AVL9 | ENSG00000105778 | ENST00000318709 |
| DNMT3B | ENSG00000088305 | ENST00000344505 |
| CDHR1 | ENSG00000148600 | ENST00000372117 |
| SEC61A2 | ENSG00000065665 | ENST00000379020 |
| TMOD2 | ENSG00000128872 | ENST00000249700 |
| HDHD2 | ENSG00000167220 | ENST00000588183 |
| ZNF362 | ENSG00000160094 | ENST00000539719 |
| HAPLN1 | ENSG00000145681 | ENST00000274341 |
| HNRNPH3 | ENSG00000096746 | ENST00000265866 |
| SLC12A5 | ENSG00000124140 | ENST00000243964 |
| G3BP1 | ENSG00000145907 | ENST00000394123 |
| NUDT19 | ENSG00000213965 | ENST00000397061 |
| RAB5A | ENSG00000144566 | ENST00000273047 |
| LRRIQ3 | ENSG00000162620 | ENST00000415760 |
| SEPHS1 | ENSG00000086475 | ENST00000327347 |
| UBE2B | ENSG00000119048 | ENST00000265339 |
| NECAB1 | ENSG00000123119 | ENST00000417640 |
| FAM114A2 | ENSG00000055147 | ENST00000351797 |
| FNIP1 | ENSG00000217128 | ENST00000307954 |
| RAC1 | ENSG00000136238 | ENST00000348035 |
| SERAC1 | ENSG00000122335 | ENST00000607742 |
| NEDD4L | ENSG00000049759 | ENST00000256832 |
| RIPK4 | ENSG00000183421 | ENST00000332512 |
| TOM1L1 | ENSG00000141198 | ENST00000572405 |
| CCNG2 | ENSG00000138764 | ENST00000316355 |
| ZNF80 | ENSG00000174255 | ENST00000482457 |
| C10orf118 | ENSG00000165813 | ENST00000369287 |
| ZNF548 | ENSG00000188785 | ENST00000366197 |
| CFLAR | ENSG00000003402 | ENST00000309955 |
| IQUB | ENSG00000164675 | ENST00000484508 |
| C20orf194 | ENSG00000088854 | ENST00000252032 |
| PRPF38B | ENSG00000134186 | ENST00000370021 |
| CEP192 | ENSG00000101639 | ENST00000513432 |
| CALD1 | ENSG00000122786 | ENST00000443197 |
| MTERFD1 | ENSG00000156469 | ENST00000523821 |
| SSBP2 | ENSG00000145687 | ENST00000320672 |
| MLLT10 | ENSG00000078403 | ENST00000377072 |
| BLOC1S6 | ENSG00000104164 | ENST00000220531 |
| PIN1 | ENSG00000127445 | ENST00000586352 |
| LPHN2 | ENSG00000117114 | ENST00000370715 |
| PHF12 | ENSG00000109118 | ENST00000577226 |
| TET3 | ENSG00000187605 | ENST00000409262 |
| CDS2 | ENSG00000101290 | ENST00000460006 |
| VPS13C | ENSG00000129003 | ENST00000249837 |
| LIMS1 | ENSG00000169756 | ENST00000393310 |
| FIBP | ENSG00000172500 | ENST00000533037 |
| NKD1 | ENSG00000140807 | ENST00000268459 |
| ITGA10 | ENSG00000143127 | ENST00000369304 |
| KIAA0040 | ENSG00000235750 | ENST00000444639 |
| ANGPTL3 | ENSG00000132855 | ENST00000371129 |
| TSHZ2 | ENSG00000182463 | ENST00000371497 |
| FRK | ENSG00000111816 | ENST00000606080 |
| PLS1 | ENSG00000120756 | ENST00000457734 |
| MXI1 | ENSG00000119950 | ENST00000332674 |
| ME1 | ENSG00000065833 | ENST00000369705 |
| SFMBT1 | ENSG00000163935 | ENST00000394752 |
| FAM13C | ENSG00000148541 | ENST00000513059 |
| TRIM52 | ENSG00000183718 | ENST00000327767 |
| KIAA1715 | ENSG00000144320 | ENST00000272748 |
| TMEM67 | ENSG00000164953 | ENST00000453321 |
| PROM1 | ENSG00000007062 | ENST00000511153 |
| PAK6 | ENSG00000137843 | ENST00000260404 |
| EIF4B | ENSG00000063046 | ENST00000262056 |
| CDCA7 | ENSG00000144354 | ENST00000306721 |
| CHORDC1 | ENSG00000110172 | ENST00000320585 |
| PIAS2 | ENSG00000078043 | ENST00000585916 |
| FMNL2 | ENSG00000157827 | ENST00000475377 |
| RAB23 | ENSG00000112210 | ENST00000317483 |
| B3GALT2 | ENSG00000162630 | ENST00000367434 |
| SORBS1 | ENSG00000095637 | ENST00000371227 |
| PIK3R1 | ENSG00000145675 | ENST00000521381 |
| ADCY2 | ENSG00000078295 | ENST00000338316 |
| KRT80 | ENSG00000167767 | ENST00000313234 |
| VHL | ENSG00000134086 | ENST00000256474 |
| SHCBP1 | ENSG00000171241 | ENST00000303383 |
| MIER3 | ENSG00000155545 | ENST00000452157 |
| SLC26A4 | ENSG00000091137 | ENST00000265715 |
| DOT1L | ENSG00000104885 | ENST00000398665 |
| EBAG9 | ENSG00000147654 | ENST00000337573 |
| PPP1R3D | ENSG00000132825 | ENST00000370996 |
| NIPSNAP3B | ENSG00000165028 | ENST00000374762 |
| C5orf64 | ENSG00000178722 | ENST00000505642 |
| PMS1 | ENSG00000064933 | ENST00000342075 |
| AMOT | ENSG00000126016 | ENST00000304758 |
| AC011897.1 | ENSG00000265629 | ENST00000581929 |
| NUAK1 | ENSG00000074590 | ENST00000261402 |
| HOXC13 | ENSG00000123364 | ENST00000243056 |
| AXIN2 | ENSG00000168646 | ENST00000307078 |
| MOCS1 | ENSG00000124615 | ENST00000373186 |
| CPNE2 | ENSG00000140848 | ENST00000535318 |
| DMRT2 | ENSG00000173253 | ENST00000259622 |
| ZFAND4 | ENSG00000172671 | ENST00000335258 |
| ARHGEF7 | ENSG00000102606 | ENST00000375741 |
| SNRPE | ENSG00000182004 | ENST00000414487 |
| ZNF365 | ENSG00000138311 | ENST00000395254 |
| PDZD8 | ENSG00000165650 | ENST00000334464 |
| ELF2 | ENSG00000109381 | ENST00000394235 |
| DYNLRB2 | ENSG00000168589 | ENST00000561880 |
| PGR | ENSG00000082175 | ENST00000325455 |
| NUP35 | ENSG00000163002 | ENST00000295119 |
| RRP8 | ENSG00000132275 | ENST00000254605 |
| PRKCA | ENSG00000154229 | ENST00000413366 |
| METTL25 | ENSG00000127720 | ENST00000550298 |
| CYP3A4 | ENSG00000160868 | ENST00000354593 |
| DHX29 | ENSG00000067248 | ENST00000251636 |
| MRFAP1 | ENSG00000179010 | ENST00000382581 |
| NOC3L | ENSG00000173145 | ENST00000371361 |
| ADAMTS18 | ENSG00000140873 | ENST00000282849 |
| GRK4 | ENSG00000125388 | ENST00000503518 |
| KIF11 | ENSG00000138160 | ENST00000260731 |
| RASEF | ENSG00000165105 | ENST00000376447 |
| C1orf109 | ENSG00000116922 | ENST00000494120 |
| SEC14L6 | ENSG00000214491 | ENST00000402034 |
| KIF24 | ENSG00000186638 | ENST00000402558 |
| HNRNPH2 | ENSG00000126945 | ENST00000316594 |
| CAND1 | ENSG00000111530 | ENST00000545606 |
| PCDHB14 | ENSG00000120327 | ENST00000239449 |
| BTBD9 | ENSG00000183826 | ENST00000314100 |
| BHMT | ENSG00000145692 | ENST00000274353 |
| EIF4E | ENSG00000151247 | ENST00000450253 |
| YEATS4 | ENSG00000127337 | ENST00000247843 |
| ARID1B | ENSG00000049618 | ENST00000346085 |
| KLK7 | ENSG00000169035 | ENST00000304045 |
| TUSC1 | ENSG00000198680 | ENST00000358022 |
| GABPB1 | ENSG00000104064 | ENST00000380877 |
| ARL14EPL | ENSG00000268223 | ENST00000601302 |
| GOLGA6L6 | ENSG00000215405 | ENST00000427390 |
| ELOVL2 | ENSG00000197977 | ENST00000354666 |
| SYDE2 | ENSG00000097096 | ENST00000341460 |
| ATP7A | ENSG00000165240 | ENST00000343533 |
| ZBTB14 | ENSG00000198081 | ENST00000357006 |
| ZSCAN12 | ENSG00000158691 | ENST00000361028 |
| DYRK1A | ENSG00000157540 | ENST00000339659 |
| SEL1L | ENSG00000071537 | ENST00000336735 |
| TADA2B | ENSG00000173011 | ENST00000310074 |
| GATAD2B | ENSG00000143614 | ENST00000368655 |
| CKAP2L | ENSG00000169607 | ENST00000541405 |
| ETV3 | ENSG00000117036 | ENST00000368192 |
| ST3GAL5 | ENSG00000115525 | ENST00000377332 |
| STARD13 | ENSG00000133121 | ENST00000336934 |
| GRIA2 | ENSG00000120251 | ENST00000296526 |
| DBR1 | ENSG00000138231 | ENST00000260803 |
| CDR2 | ENSG00000140743 | ENST00000268383 |
| RBPMS | ENSG00000157110 | ENST00000320203 |
| SWT1 | ENSG00000116668 | ENST00000367500 |
| HMCN1 | ENSG00000143341 | ENST00000367492 |
| PCSK1 | ENSG00000175426 | ENST00000311106 |
| TPRG1 | ENSG00000188001 | ENST00000345063 |
| USP43 | ENSG00000154914 | ENST00000285199 |
| ARHGAP18 | ENSG00000146376 | ENST00000368149 |
| SYT4 | ENSG00000132872 | ENST00000255224 |
| NUP43 | ENSG00000120253 | ENST00000367403 |
| STAC | ENSG00000144681 | ENST00000273183 |
| BAZ1A | ENSG00000198604 | ENST00000358716 |
| PTGER2 | ENSG00000125384 | ENST00000245457 |
| CDH7 | ENSG00000081138 | ENST00000536984 |
| MAP3K19 | ENSG00000176601 | ENST00000392915 |
| NRK | ENSG00000123572 | ENST00000243300 |
| TOPBP1 | ENSG00000163781 | ENST00000260810 |
| DCX | ENSG00000077279 | ENST00000371993 |
| NDNL2 | ENSG00000185115 | ENST00000332303 |
| YWHAE | ENSG00000108953 | ENST00000264335 |
| ZNF720 | ENSG00000197302 | ENST00000534277 |
| ZNF25 | ENSG00000175395 | ENST00000302609 |
| KCNJ16 | ENSG00000153822 | ENST00000585558 |
| RHBDD1 | ENSG00000144468 | ENST00000341329 |
| HEBP1 | ENSG00000013583 | ENST00000536942 |
| ZBTB34 | ENSG00000177125 | ENST00000319119 |
| RUNX1 | ENSG00000159216 | ENST00000325074 |
| TDRD6 | ENSG00000180113 | ENST00000544460 |
| FOXF2 | ENSG00000137273 | ENST00000259806 |
| RBM45 | ENSG00000155636 | ENST00000455903 |
| FGF12 | ENSG00000114279 | ENST00000445105 |
| COL21A1 | ENSG00000124749 | ENST00000488912 |
| ZNF274 | ENSG00000171606 | ENST00000326804 |
| CPNE4 | ENSG00000196353 | ENST00000512055 |
| LANCL3 | ENSG00000147036 | ENST00000378621 |
| ENKUR | ENSG00000151023 | ENST00000331161 |
| SCML2 | ENSG00000102098 | ENST00000420857 |
| MRAP2 | ENSG00000135324 | ENST00000257776 |
| AP5S1 | ENSG00000125843 | ENST00000379573 |
| KLHL24 | ENSG00000114796 | ENST00000454652 |
| THRB | ENSG00000151090 | ENST00000396671 |
| MSMO1 | ENSG00000052802 | ENST00000261507 |
| FREM2 | ENSG00000150893 | ENST00000280481 |
| MAP3K8 | ENSG00000107968 | ENST00000375321 |
| IGSF11 | ENSG00000144847 | ENST00000425327 |
| TUBB | ENSG00000196230 | ENST00000330914 |
| USP49 | ENSG00000164663 | ENST00000394253 |
| NNT | ENSG00000112992 | ENST00000264663 |
| PTCH1 | ENSG00000185920 | ENST00000375290 |
| CDC42SE2 | ENSG00000158985 | ENST00000505065 |
| GOLGA6L20 | ENSG00000196648 | ENST00000358583 |
| ACER3 | ENSG00000078124 | ENST00000532485 |
| FLJ20373 | ENSG00000233404 | ENST00000414004 |
| TXNDC15 | ENSG00000113621 | ENST00000507024 |
| LHFPL3 | ENSG00000187416 | ENST00000535008 |
| G3BP2 | ENSG00000138757 | ENST00000359707 |
| ISM1 | ENSG00000101230 | ENST00000262487 |
| RP11-156E8.1 | ENSG00000272195 | ENST00000607453 |
| TRIM56 | ENSG00000169871 | ENST00000306085 |
| FLT4 | ENSG00000037280 | ENST00000261937 |
| AUTS2 | ENSG00000158321 | ENST00000342771 |
| STK32A | ENSG00000169302 | ENST00000397936 |
| PPID | ENSG00000171497 | ENST00000507213 |
| FAM114A1 | ENSG00000197712 | ENST00000358869 |
| ST8SIA6 | ENSG00000148488 | ENST00000377602 |
| ALDH1A1 | ENSG00000165092 | ENST00000297785 |
| ATCAY | ENSG00000167654 | ENST00000450849 |
| KLF3 | ENSG00000109787 | ENST00000261438 |
| RASSF6 | ENSG00000169435 | ENST00000307439 |
| CEP55 | ENSG00000138180 | ENST00000371485 |
| C12orf23 | ENSG00000151135 | ENST00000548125 |
| ZNF480 | ENSG00000198464 | ENST00000595962 |
| RAB27B | ENSG00000041353 | ENST00000262094 |
| RAD18 | ENSG00000070950 | ENST00000264926 |
| ZNF213 | ENSG00000085644 | ENST00000576863 |
| GPR116 | ENSG00000069122 | ENST00000362015 |
| PRPF40A | ENSG00000196504 | ENST00000410080 |
| SNX13 | ENSG00000071189 | ENST00000409389 |
| CDK12 | ENSG00000167258 | ENST00000447079 |
| CEP135 | ENSG00000174799 | ENST00000257287 |
| UBE2K | ENSG00000078140 | ENST00000261427 |
| PDE4DIP | ENSG00000178104 | ENST00000529945 |
| BCAP29 | ENSG00000075790 | ENST00000379119 |
| PNISR | ENSG00000132424 | ENST00000369239 |
| GUF1 | ENSG00000151806 | ENST00000281543 |
| RAD51B | ENSG00000182185 | ENST00000487270 |
| XPO4 | ENSG00000132953 | ENST00000400602 |
| HOXB3 | ENSG00000120093 | ENST00000470495 |
| NRCAM | ENSG00000091129 | ENST00000379028 |
| SKIL | ENSG00000136603 | ENST00000458537 |
| SLC16A14 | ENSG00000163053 | ENST00000295190 |
| KAL1 | ENSG00000011201 | ENST00000262648 |
| USP12 | ENSG00000152484 | ENST00000282344 |
| TACC1 | ENSG00000147526 | ENST00000348567 |
| KLHL29 | ENSG00000119771 | ENST00000486442 |
| FBXL4 | ENSG00000112234 | ENST00000369244 |
| CCDC43 | ENSG00000180329 | ENST00000457422 |
| ROCK1 | ENSG00000067900 | ENST00000399799 |
| EPS8L2 | ENSG00000177106 | ENST00000526909 |
| GPN3 | ENSG00000111231 | ENST00000228827 |
| EFCAB10 | ENSG00000185055 | ENST00000486180 |
| USP46 | ENSG00000109189 | ENST00000441222 |
| MAP10 | ENSG00000212916 | ENST00000418460 |
| ZNF354B | ENSG00000178338 | ENST00000322434 |
| SV2C | ENSG00000122012 | ENST00000502798 |
| CKLF | ENSG00000217555 | ENST00000534692 |
| GPR63 | ENSG00000112218 | ENST00000229955 |
| FAM84A | ENSG00000162981 | ENST00000295092 |
| TMEM236 | ENSG00000184040 | ENST00000480516 |
| MBNL1 | ENSG00000152601 | ENST00000357472 |
| PURB | ENSG00000146676 | ENST00000395699 |
| SYNC | ENSG00000162520 | ENST00000373484 |
| ETV1 | ENSG00000006468 | ENST00000430479 |
| TCF21 | ENSG00000118526 | ENST00000367882 |
| NCEH1 | ENSG00000144959 | ENST00000475381 |
| ZNF669 | ENSG00000188295 | ENST00000358785 |
| SLC35A3 | ENSG00000117620 | ENST00000465289 |
| IRAK1BP1 | ENSG00000146243 | ENST00000369940 |
| HOMER1 | ENSG00000152413 | ENST00000334082 |
| TRPV4 | ENSG00000111199 | ENST00000538125 |
| PRKAA2 | ENSG00000162409 | ENST00000371244 |
| KCNH8 | ENSG00000183960 | ENST00000452398 |
| VWDE | ENSG00000146530 | ENST00000521169 |
| BCL11B | ENSG00000127152 | ENST00000345514 |
| AADACL2 | ENSG00000197953 | ENST00000445270 |
| NOTCH2NL | ENSG00000213240 | ENST00000369340 |
| ZNF250 | ENSG00000196150 | ENST00000292579 |
| AASDHPPT | ENSG00000149313 | ENST00000278618 |
| HTR2B | ENSG00000135914 | ENST00000258400 |
| WWC1 | ENSG00000113645 | ENST00000265293 |
| UBE2J1 | ENSG00000198833 | ENST00000435041 |
| SPATA22 | ENSG00000141255 | ENST00000355380 |
| AKAP10 | ENSG00000108599 | ENST00000225737 |
| NOX5 | ENSG00000255346 | ENST00000260364 |
| IGIP | ENSG00000182700 | ENST00000333305 |
| PELO | ENSG00000152684 | ENST00000274311 |
| C9orf84 | ENSG00000165181 | ENST00000394779 |
| PHF20L1 | ENSG00000129292 | ENST00000460236 |
| ISL1 | ENSG00000016082 | ENST00000230658 |
| HSPA4L | ENSG00000164070 | ENST00000296464 |
| CEP19 | ENSG00000174007 | ENST00000409690 |
| KMO | ENSG00000117009 | ENST00000366559 |
| AP1AR | ENSG00000138660 | ENST00000274000 |
| SAR1B | ENSG00000152700 | ENST00000402673 |
| CPEB4 | ENSG00000113742 | ENST00000265085 |
| CA13 | ENSG00000185015 | ENST00000321764 |
| SBF1 | ENSG00000100241 | ENST00000380817 |
| SC5D | ENSG00000109929 | ENST00000264027 |
| RIT1 | ENSG00000143622 | ENST00000368323 |
| SLC10A7 | ENSG00000120519 | ENST00000432059 |
| EFNA5 | ENSG00000184349 | ENST00000333274 |
| DCHS2 | ENSG00000197410 | ENST00000357232 |
| ANKRD28 | ENSG00000206560 | ENST00000399451 |
| EID2B | ENSG00000176401 | ENST00000326282 |
| NTPCR | ENSG00000135778 | ENST00000366628 |
| NOMO2 | ENSG00000185164 | ENST00000564991 |
| UBTD2 | ENSG00000168246 | ENST00000393792 |
| SMAD9 | ENSG00000120693 | ENST00000399275 |
| PRDX2 | ENSG00000167815 | ENST00000334482 |
| RP11-93B14.6 | ENSG00000167046 | ENST00000370520 |
| DIS3 | ENSG00000083520 | ENST00000377767 |
| C12orf29 | ENSG00000133641 | ENST00000550333 |
| IGF2R | ENSG00000197081 | ENST00000356956 |
| TRPM7 | ENSG00000092439 | ENST00000560955 |
| ZBTB49 | ENSG00000168826 | ENST00000503703 |
| TVP23B | ENSG00000171928 | ENST00000476139 |
| POLQ | ENSG00000051341 | ENST00000264233 |
| OSMR | ENSG00000145623 | ENST00000274276 |
| ASAH2 | ENSG00000188611 | ENST00000395526 |
| LEPR | ENSG00000116678 | ENST00000349533 |
| HAUS1 | ENSG00000152240 | ENST00000591715 |
| NAA50 | ENSG00000121579 | ENST00000240922 |
| RTN4 | ENSG00000115310 | ENST00000394611 |
| TIGD1 | ENSG00000221944 | ENST00000408957 |
| DENND5A | ENSG00000184014 | ENST00000328194 |
| CCDC125 | ENSG00000183323 | ENST00000396496 |
| TIMELESS | ENSG00000111602 | ENST00000229201 |
| DBT | ENSG00000137992 | ENST00000370132 |
| TBCEL | ENSG00000154114 | ENST00000422003 |
| FPGT-TNNI3K | ENSG00000259030 | ENST00000370895 |
| TET1 | ENSG00000138336 | ENST00000373644 |
| PTBP3 | ENSG00000119314 | ENST00000374257 |
| AGPS | ENSG00000018510 | ENST00000264167 |
| LEMD3 | ENSG00000174106 | ENST00000308330 |
| STX1A | ENSG00000106089 | ENST00000395156 |
| EPHA5 | ENSG00000145242 | ENST00000273854 |
| FGFR1OP | ENSG00000213066 | ENST00000366847 |
| ATF7 | ENSG00000170653 | ENST00000420353 |
| IQCK | ENSG00000174628 | ENST00000308214 |
| ZMYM2 | ENSG00000121741 | ENST00000382869 |
| PMEPA1 | ENSG00000124225 | ENST00000395816 |
| MAGOH | ENSG00000162385 | ENST00000371470 |
| SYNM | ENSG00000182253 | ENST00000560674 |
| ZNF721 | ENSG00000182903 | ENST00000338977 |
| G2E3 | ENSG00000092140 | ENST00000206595 |
| PTBP1 | ENSG00000011304 | ENST00000350092 |
| GPX6 | ENSG00000198704 | ENST00000361902 |
| RAB2A | ENSG00000104388 | ENST00000262646 |
| KRBOX4 | ENSG00000147121 | ENST00000360017 |
| YY1 | ENSG00000100811 | ENST00000262238 |
| CNTN4 | ENSG00000144619 | ENST00000427331 |
| SLITRK4 | ENSG00000179542 | ENST00000381779 |
| CDKN2AIP | ENSG00000168564 | ENST00000302350 |
| ARHGEF17 | ENSG00000110237 | ENST00000263674 |
| ZNF655 | ENSG00000197343 | ENST00000425063 |
| CPXCR1 | ENSG00000147183 | ENST00000276127 |
| ARHGAP31 | ENSG00000031081 | ENST00000264245 |
| F3 | ENSG00000117525 | ENST00000334047 |
| RP11-133K1.2 | ENSG00000259288 | ENST00000558658 |
| MKRN1 | ENSG00000133606 | ENST00000255977 |
| RTN1 | ENSG00000139970 | ENST00000395090 |
| PLAG1 | ENSG00000181690 | ENST00000316981 |
| ABCA5 | ENSG00000154265 | ENST00000392676 |
| RWDD1 | ENSG00000111832 | ENST00000466444 |
| RBL1 | ENSG00000080839 | ENST00000373664 |
| PTPN4 | ENSG00000088179 | ENST00000263708 |
| TMEM38B | ENSG00000095209 | ENST00000374692 |
| GATC | ENSG00000257218 | ENST00000551765 |
| GRM1 | ENSG00000152822 | ENST00000492807 |
| ZIC3 | ENSG00000156925 | ENST00000287538 |
| RANBP2 | ENSG00000153201 | ENST00000283195 |
| SLC9A2 | ENSG00000115616 | ENST00000233969 |
| NR4A2 | ENSG00000153234 | ENST00000339562 |
| PPP1R8 | ENSG00000117751 | ENST00000311772 |
| MTUS1 | ENSG00000129422 | ENST00000520196 |
| DNMT3A | ENSG00000119772 | ENST00000380756 |
| TAF1A | ENSG00000143498 | ENST00000350027 |
| TGS1 | ENSG00000137574 | ENST00000523948 |
| CEBPD | ENSG00000221869 | ENST00000408965 |
| SOGA3 | ENSG00000214338 | ENST00000556132 |
| ZNF286A | ENSG00000187607 | ENST00000395894 |
| WDR20 | ENSG00000140153 | ENST00000322340 |
| OCIAD1 | ENSG00000109180 | ENST00000425583 |
| AGBL3 | ENSG00000146856 | ENST00000275763 |
| RP11-248J23.6 | ENSG00000269948 | ENST00000469549 |
| PIK3R6 | ENSG00000174083 | ENST00000583984 |
| MAML1 | ENSG00000161021 | ENST00000292599 |
| ROR2 | ENSG00000169071 | ENST00000375708 |
| PFDN4 | ENSG00000101132 | ENST00000371419 |
| NDNF | ENSG00000173376 | ENST00000379692 |
| GRIN3A | ENSG00000198785 | ENST00000361820 |
| GSE1 | ENSG00000131149 | ENST00000253458 |
| MSI2 | ENSG00000153944 | ENST00000284073 |
| GRAMD1B | ENSG00000023171 | ENST00000529750 |
| MED12L | ENSG00000144893 | ENST00000474524 |
| HNRNPM | ENSG00000099783 | ENST00000600806 |
| SRGAP3 | ENSG00000196220 | ENST00000383836 |
| SIK2 | ENSG00000170145 | ENST00000304987 |
| RBM7 | ENSG00000076053 | ENST00000541475 |
| ZNF845 | ENSG00000213799 | ENST00000458035 |
| CDK16 | ENSG00000102225 | ENST00000457458 |
| SUV39H2 | ENSG00000152455 | ENST00000378331 |
| INPP4B | ENSG00000109452 | ENST00000513000 |
| BIRC6 | ENSG00000115760 | ENST00000421745 |
| UHRF1BP1L | ENSG00000111647 | ENST00000279907 |
| CLCN6 | ENSG00000011021 | ENST00000312413 |
| MYO5A | ENSG00000197535 | ENST00000399231 |
| TLR3 | ENSG00000164342 | ENST00000296795 |
| APP | ENSG00000142192 | ENST00000346798 |
| CPOX | ENSG00000080819 | ENST00000264193 |
| NAPB | ENSG00000125814 | ENST00000377026 |
| PILRB | ENSG00000121716 | ENST00000448382 |
| KIAA0226L | ENSG00000102445 | ENST00000378781 |
| TNFSF8 | ENSG00000106952 | ENST00000223795 |
| DCP2 | ENSG00000172795 | ENST00000389063 |
| SNX10 | ENSG00000086300 | ENST00000396376 |
| LARP4 | ENSG00000161813 | ENST00000398473 |
| HDAC4 | ENSG00000068024 | ENST00000345617 |
| CSNK2A2 | ENSG00000070770 | ENST00000563307 |
| SDC2 | ENSG00000169439 | ENST00000302190 |
| MXD1 | ENSG00000059728 | ENST00000264444 |
| FRYL | ENSG00000075539 | ENST00000503238 |
| MYOZ2 | ENSG00000172399 | ENST00000307128 |
| LHX4 | ENSG00000121454 | ENST00000263726 |
| MALT1 | ENSG00000172175 | ENST00000348428 |
| CAPN8 | ENSG00000203697 | ENST00000366873 |
| WDR44 | ENSG00000131725 | ENST00000254029 |
| NR3C2 | ENSG00000151623 | ENST00000344721 |
| MGAT5 | ENSG00000152127 | ENST00000409645 |
| AFF1 | ENSG00000172493 | ENST00000395146 |
| ZFY | ENSG00000067646 | ENST00000383052 |
| VAMP7 | ENSG00000124333 | ENST00000286448 |
| SLITRK5 | ENSG00000165300 | ENST00000325089 |
| ROR1 | ENSG00000185483 | ENST00000371079 |
| UBE2D3 | ENSG00000109332 | ENST00000453744 |
| PDP1 | ENSG00000164951 | ENST00000396200 |
| PPAT | ENSG00000128059 | ENST00000264220 |
| AMBN | ENSG00000178522 | ENST00000322937 |
| MYSM1 | ENSG00000162601 | ENST00000472487 |
| RSPRY1 | ENSG00000159579 | ENST00000394420 |
| POTEB | ENSG00000233917 | ENST00000435397 |
| POTEB2 | ENSG00000230031 | ENST00000438063 |
| C11orf82 | ENSG00000165490 | ENST00000528759 |
| WDFY1 | ENSG00000085449 | ENST00000233055 |
| KSR2 | ENSG00000171435 | ENST00000425217 |
| RNF6 | ENSG00000127870 | ENST00000381588 |
| CDC42BPG | ENSG00000171219 | ENST00000342711 |
| ARHGAP28 | ENSG00000088756 | ENST00000419673 |
| PDE8B | ENSG00000113231 | ENST00000264917 |
| NAALADL2 | ENSG00000177694 | ENST00000454872 |
| CDK13 | ENSG00000065883 | ENST00000181839 |
| FAM13B | ENSG00000031003 | ENST00000033079 |
| AQP11 | ENSG00000178301 | ENST00000313578 |
| MEGF10 | ENSG00000145794 | ENST00000503335 |
| MGEA5 | ENSG00000198408 | ENST00000439817 |
| SATB1 | ENSG00000182568 | ENST00000338745 |
| SMPD4 | ENSG00000136699 | ENST00000454468 |
| ZNF831 | ENSG00000124203 | ENST00000371030 |
| SLC6A4 | ENSG00000108576 | ENST00000401766 |
| RHOBTB1 | ENSG00000072422 | ENST00000357917 |
| CLTC | ENSG00000141367 | ENST00000269122 |
| GMPS | ENSG00000163655 | ENST00000496455 |
| DRGX | ENSG00000165606 | ENST00000374139 |
| SRGAP1 | ENSG00000196935 | ENST00000357825 |
| SEPT6 | ENSG00000125354 | ENST00000394610 |
| NRG3 | ENSG00000185737 | ENST00000602794 |
| CD2AP | ENSG00000198087 | ENST00000359314 |
| LSM12 | ENSG00000161654 | ENST00000591247 |
| UBE2G2 | ENSG00000184787 | ENST00000345496 |
| DAP3 | ENSG00000132676 | ENST00000368336 |
| RAB3GAP2 | ENSG00000118873 | ENST00000358951 |
| RP11-297M9.1 | ENSG00000260362 | ENST00000561538 |
| LYN | ENSG00000254087 | ENST00000520220 |
| NEDD4 | ENSG00000069869 | ENST00000503468 |
| ADAM23 | ENSG00000114948 | ENST00000264377 |
| LMAN2L | ENSG00000114988 | ENST00000434524 |
| TCF19 | ENSG00000137310 | ENST00000376255 |
| CNN3 | ENSG00000117519 | ENST00000370206 |
| DDX6 | ENSG00000110367 | ENST00000264018 |
| RFTN2 | ENSG00000162944 | ENST00000295049 |
| KIAA0196 | ENSG00000164961 | ENST00000318410 |
| ANKRD12 | ENSG00000101745 | ENST00000262126 |
| FBXL20 | ENSG00000108306 | ENST00000264658 |
| ESD | ENSG00000139684 | ENST00000471867 |
| CEBPG | ENSG00000153879 | ENST00000284000 |
| GEM | ENSG00000164949 | ENST00000396194 |
| NR2C2 | ENSG00000177463 | ENST00000323373 |
| CLEC4E | ENSG00000166523 | ENST00000299663 |
| SOAT1 | ENSG00000057252 | ENST00000367619 |
| FAM154B | ENSG00000188659 | ENST00000339465 |
| KIAA1024 | ENSG00000169330 | ENST00000305428 |
| CSGALNACT2 | ENSG00000169826 | ENST00000374466 |
| MAGEC2 | ENSG00000046774 | ENST00000247452 |
| DYNC1LI1 | ENSG00000144635 | ENST00000273130 |
| CASC1 | ENSG00000118307 | ENST00000554533 |
| GK5 | ENSG00000175066 | ENST00000480757 |
| EPHA4 | ENSG00000116106 | ENST00000281821 |
| LRRC8C | ENSG00000171488 | ENST00000370454 |
| ANAPC13 | ENSG00000129055 | ENST00000510994 |
| GALC | ENSG00000054983 | ENST00000261304 |
| MITF | ENSG00000187098 | ENST00000328528 |
| RBM27 | ENSG00000091009 | ENST00000265271 |
| NAIP | ENSG00000249437 | ENST00000517649 |
| DCAF4L1 | ENSG00000182308 | ENST00000333141 |
| FAM115A | ENSG00000198420 | ENST00000479870 |
| TPD52L1 | ENSG00000111907 | ENST00000368388 |
| FAM98B | ENSG00000171262 | ENST00000397609 |
| MARCH1 | ENSG00000145416 | ENST00000274056 |
| TUBB2A | ENSG00000137267 | ENST00000333628 |
| WDR26 | ENSG00000162923 | ENST00000414423 |
| ERV3-1 | ENSG00000213462 | ENST00000394323 |
| SOD2 | ENSG00000112096 | ENST00000538183 |
| SORD | ENSG00000140263 | ENST00000267814 |
| MTMR6 | ENSG00000139505 | ENST00000381801 |
| ERMP1 | ENSG00000099219 | ENST00000339450 |
| SPIN1 | ENSG00000106723 | ENST00000375859 |
| FYB | ENSG00000082074 | ENST00000351578 |
| ZNF229 | ENSG00000167383 | ENST00000591604 |
| IBTK | ENSG00000005700 | ENST00000503400 |
| CDH11 | ENSG00000140937 | ENST00000394156 |
| ATG10 | ENSG00000152348 | ENST00000282185 |
| ARAF | ENSG00000078061 | ENST00000377039 |
| CLDN18 | ENSG00000066405 | ENST00000343735 |
| ZNF81 | ENSG00000197779 | ENST00000338637 |
| ASB2 | ENSG00000100628 | ENST00000555019 |
| AHNAK | ENSG00000124942 | ENST00000378024 |
| NMD3 | ENSG00000169251 | ENST00000351193 |
| MFAP1 | ENSG00000140259 | ENST00000267812 |
| SLC12A2 | ENSG00000064651 | ENST00000262461 |
| RIMS3 | ENSG00000117016 | ENST00000372684 |
| PRPF39 | ENSG00000185246 | ENST00000554081 |
| SNX14 | ENSG00000135317 | ENST00000369635 |
| ALG14 | ENSG00000172339 | ENST00000370205 |
| CHRNB2 | ENSG00000160716 | ENST00000368476 |
| SLC24A1 | ENSG00000074621 | ENST00000339868 |
| C5 | ENSG00000106804 | ENST00000223642 |
| TMX1 | ENSG00000139921 | ENST00000556683 |
| XRCC3 | ENSG00000126215 | ENST00000557439 |
| SH2D1B | ENSG00000198574 | ENST00000367929 |
| LRRC17 | ENSG00000128606 | ENST00000249377 |
| RABEP1 | ENSG00000029725 | ENST00000408982 |
| MDGA2 | ENSG00000139915 | ENST00000426342 |
| CUL3 | ENSG00000036257 | ENST00000264414 |
| CLINT1 | ENSG00000113282 | ENST00000523094 |
| P4HA3 | ENSG00000149380 | ENST00000524388 |
| RNF10 | ENSG00000022840 | ENST00000325954 |
| EPC2 | ENSG00000135999 | ENST00000409654 |
| ZNF527 | ENSG00000189164 | ENST00000356178 |
| DDX46 | ENSG00000145833 | ENST00000452510 |
| BCL2 | ENSG00000171791 | ENST00000398117 |
| DDIT4L | ENSG00000145358 | ENST00000273990 |
| TTF2 | ENSG00000116830 | ENST00000369466 |
| GLRX | ENSG00000173221 | ENST00000379979 |
| HLF | ENSG00000108924 | ENST00000226067 |
| WDR75 | ENSG00000115368 | ENST00000427960 |
| MTMR11 | ENSG00000014914 | ENST00000406732 |
| KLHL41 | ENSG00000239474 | ENST00000284669 |
| C1QTNF7 | ENSG00000163145 | ENST00000429690 |
| ZNF236 | ENSG00000130856 | ENST00000543926 |
| SESTD1 | ENSG00000187231 | ENST00000428443 |
| WRB | ENSG00000182093 | ENST00000380708 |
| GDAP2 | ENSG00000196505 | ENST00000369443 |
| ALG10 | ENSG00000139133 | ENST00000541875 |
| CPD | ENSG00000108582 | ENST00000225719 |
| ZBTB44 | ENSG00000196323 | ENST00000525842 |
| GABBR2 | ENSG00000136928 | ENST00000259455 |
| RFC3 | ENSG00000133119 | ENST00000380071 |
| MCM10 | ENSG00000065328 | ENST00000378694 |
| ALDH7A1 | ENSG00000164904 | ENST00000409134 |
| SETX | ENSG00000107290 | ENST00000436441 |
| NUCKS1 | ENSG00000069275 | ENST00000367142 |
| GABRA2 | ENSG00000151834 | ENST00000507460 |
| PPP2R5C | ENSG00000078304 | ENST00000422945 |
| FOXP1 | ENSG00000114861 | ENST00000318789 |
| SMIM14 | ENSG00000163683 | ENST00000295958 |
| CCT4 | ENSG00000115484 | ENST00000394440 |
| SLC31A1 | ENSG00000136868 | ENST00000374212 |
| IMPACT | ENSG00000154059 | ENST00000284202 |
| UBE2E3 | ENSG00000170035 | ENST00000602710 |
| POU2F2 | ENSG00000028277 | ENST00000389341 |
| HP1BP3 | ENSG00000127483 | ENST00000312239 |
| MDM4 | ENSG00000198625 | ENST00000367182 |
| CCDC127 | ENSG00000164366 | ENST00000296824 |
| UBE2H | ENSG00000186591 | ENST00000355621 |
| NIPA1 | ENSG00000170113 | ENST00000337435 |
| REPS1 | ENSG00000135597 | ENST00000483468 |
| ZNF354C | ENSG00000177932 | ENST00000315475 |
| ZNHIT6 | ENSG00000117174 | ENST00000431532 |
| TACR3 | ENSG00000169836 | ENST00000304883 |
| TLL1 | ENSG00000038295 | ENST00000509505 |
| AFG3L2 | ENSG00000141385 | ENST00000269143 |
| MEF2D | ENSG00000116604 | ENST00000464356 |
| EYA1 | ENSG00000104313 | ENST00000465115 |
| KIF26B | ENSG00000162849 | ENST00000366518 |
| GABRB3 | ENSG00000166206 | ENST00000311550 |
| PPWD1 | ENSG00000113593 | ENST00000510930 |
| SETD2 | ENSG00000181555 | ENST00000330022 |
| ARHGEF26 | ENSG00000114790 | ENST00000356448 |
| SETDB2 | ENSG00000136169 | ENST00000354234 |
| HERC4 | ENSG00000148634 | ENST00000427635 |
| IGF2 | ENSG00000167244 | ENST00000381395 |
| NPR3 | ENSG00000113389 | ENST00000265074 |
| ERCC4 | ENSG00000175595 | ENST00000311895 |
| SIN3A | ENSG00000169375 | ENST00000567289 |
| ZNF567 | ENSG00000189042 | ENST00000536254 |
| ALDH1A3 | ENSG00000184254 | ENST00000346623 |
| BTBD11 | ENSG00000151136 | ENST00000280758 |
| DLG1 | ENSG00000075711 | ENST00000357674 |
| APOBEC1 | ENSG00000111701 | ENST00000467171 |
| OSBPL10 | ENSG00000144645 | ENST00000429492 |
| TMEM30C | ENSG00000235156 | ENST00000476804 |
| ZNF529 | ENSG00000186020 | ENST00000334116 |
| SLC10A2 | ENSG00000125255 | ENST00000245312 |
| DCAF6 | ENSG00000143164 | ENST00000470721 |
| TMEM55B | ENSG00000165782 | ENST00000250489 |
| ZNF85 | ENSG00000105750 | ENST00000300540 |
| PNN | ENSG00000100941 | ENST00000216832 |
| C9orf47 | ENSG00000186354 | ENST00000334490 |
| ADRB2 | ENSG00000169252 | ENST00000305988 |
| SCO1 | ENSG00000133028 | ENST00000255390 |
| TMEM106B | ENSG00000106460 | ENST00000396667 |
| TTC26 | ENSG00000105948 | ENST00000464848 |
| SLC16A7 | ENSG00000118596 | ENST00000261187 |
| GUCY1B3 | ENSG00000061918 | ENST00000264424 |
| RSBN1L | ENSG00000187257 | ENST00000334955 |
| IFNG | ENSG00000111537 | ENST00000229135 |
| TSHR | ENSG00000165409 | ENST00000541158 |
| TMEM229B | ENSG00000198133 | ENST00000357461 |
| ZRANB2 | ENSG00000132485 | ENST00000254821 |
| HAL | ENSG00000084110 | ENST00000544080 |
| KHDRBS3 | ENSG00000131773 | ENST00000521461 |
| ARL5B | ENSG00000165997 | ENST00000377275 |
| PAH | ENSG00000171759 | ENST00000553106 |
| EIF4A2 | ENSG00000156976 | ENST00000443963 |
| SLC16A1 | ENSG00000155380 | ENST00000369626 |
| EFR3A | ENSG00000132294 | ENST00000254624 |
| JADE2 | ENSG00000043143 | ENST00000395003 |
| CREB5 | ENSG00000146592 | ENST00000357727 |
| PRKCB | ENSG00000166501 | ENST00000303531 |
| ELOVL6 | ENSG00000170522 | ENST00000394607 |
| CHRNA7 | ENSG00000175344 | ENST00000306901 |
| SLC30A1 | ENSG00000170385 | ENST00000367001 |
| ST8SIA2 | ENSG00000140557 | ENST00000268164 |
| PAFAH1B1 | ENSG00000007168 | ENST00000397195 |
| SLC9C2 | ENSG00000162753 | ENST00000367714 |
| CDKN1B | ENSG00000111276 | ENST00000228872 |
| SGTB | ENSG00000197860 | ENST00000381007 |
| FYTTD1 | ENSG00000122068 | ENST00000241502 |
| ST3GAL2 | ENSG00000157350 | ENST00000342907 |
| EIF3F | ENSG00000175390 | ENST00000533626 |
| NUPL1 | ENSG00000139496 | ENST00000463407 |
| YTHDF1 | ENSG00000149658 | ENST00000370339 |
| ITGA4 | ENSG00000115232 | ENST00000397033 |
| PIP4K2A | ENSG00000150867 | ENST00000376573 |
| PRKDC | ENSG00000253729 | ENST00000338368 |
| NLK | ENSG00000087095 | ENST00000407008 |
| KBTBD3 | ENSG00000182359 | ENST00000534815 |
| EXOC5 | ENSG00000070367 | ENST00000413566 |
| NCOA1 | ENSG00000084676 | ENST00000288599 |
| RUNDC3B | ENSG00000105784 | ENST00000338056 |
| SMCHD1 | ENSG00000101596 | ENST00000584897 |
| ZNF140 | ENSG00000196387 | ENST00000536790 |
| FLVCR1 | ENSG00000162769 | ENST00000366971 |
| MED14 | ENSG00000180182 | ENST00000324817 |
| LRIF1 | ENSG00000121931 | ENST00000369763 |
| PTPRJ | ENSG00000149177 | ENST00000440289 |
| MCTP2 | ENSG00000140563 | ENST00000456504 |
| ATL3 | ENSG00000184743 | ENST00000398868 |
| RHOBTB3 | ENSG00000164292 | ENST00000379982 |
| NUDCD1 | ENSG00000120526 | ENST00000239690 |
| SLC25A48 | ENSG00000145832 | ENST00000425402 |
| EPAS1 | ENSG00000116016 | ENST00000263734 |
| CYB5R4 | ENSG00000065615 | ENST00000369681 |
| CDK14 | ENSG00000058091 | ENST00000380050 |
| YPEL1 | ENSG00000100027 | ENST00000339468 |
| UBE2V2 | ENSG00000169139 | ENST00000523111 |
| STPG2 | ENSG00000163116 | ENST00000522676 |
| CNIH4 | ENSG00000143771 | ENST00000366857 |
| LPHN3 | ENSG00000150471 | ENST00000512091 |
| RBAK | ENSG00000146587 | ENST00000396912 |
| EYS | ENSG00000188107 | ENST00000393380 |
| TMEM147 | ENSG00000105677 | ENST00000593027 |
| HAUS2 | ENSG00000137814 | ENST00000260372 |
| C14orf28 | ENSG00000179476 | ENST00000325192 |
| PTDSS1 | ENSG00000156471 | ENST00000517309 |
| NSRP1 | ENSG00000126653 | ENST00000394826 |
| ADH1B | ENSG00000196616 | ENST00000305046 |
| ENOPH1 | ENSG00000145293 | ENST00000273920 |
| NFAT5 | ENSG00000102908 | ENST00000426654 |
| RNF169 | ENSG00000166439 | ENST00000299563 |
| POLI | ENSG00000101751 | ENST00000579534 |
| OGFOD1 | ENSG00000087263 | ENST00000566157 |
| IP6K1 | ENSG00000176095 | ENST00000321599 |
| COL24A1 | ENSG00000171502 | ENST00000426639 |
| RC3H2 | ENSG00000056586 | ENST00000373670 |
| MTFR2 | ENSG00000146410 | ENST00000367784 |
| ZNF280D | ENSG00000137871 | ENST00000558320 |
| ZSCAN30 | ENSG00000186814 | ENST00000589178 |
| FAM92A1 | ENSG00000188343 | ENST00000518322 |
| RSPH3 | ENSG00000130363 | ENST00000367069 |
| CRH | ENSG00000147571 | ENST00000276571 |
| FAM120C | ENSG00000184083 | ENST00000375180 |
| TSPEAR | ENSG00000175894 | ENST00000323084 |
| SLC16A4 | ENSG00000168679 | ENST00000461647 |
| FAM129A | ENSG00000135842 | ENST00000367511 |
| ATP5A1 | ENSG00000152234 | ENST00000586592 |
| C1orf63 | ENSG00000117616 | ENST00000473314 |
| SLC7A14 | ENSG00000013293 | ENST00000231706 |
| RAD9B | ENSG00000151164 | ENST00000358071 |
| AMACR | ENSG00000242110 | ENST00000335606 |
| B4GALT4 | ENSG00000121578 | ENST00000467604 |
| NEK7 | ENSG00000151414 | ENST00000367385 |
| CCT5 | ENSG00000150753 | ENST00000280326 |
| IL20RA | ENSG00000016402 | ENST00000316649 |
| AOC2 | ENSG00000131480 | ENST00000253799 |
| MBIP | ENSG00000151332 | ENST00000359527 |
| PTCH2 | ENSG00000117425 | ENST00000438067 |
| FAM199X | ENSG00000123575 | ENST00000493442 |
| AKAP6 | ENSG00000151320 | ENST00000557354 |
| REV3L | ENSG00000009413 | ENST00000434009 |
| RASGRF2 | ENSG00000113319 | ENST00000265080 |
| OGT | ENSG00000147162 | ENST00000373719 |
| KRTAP3-2 | ENSG00000212900 | ENST00000391587 |
| PRKD3 | ENSG00000115825 | ENST00000379066 |
| AGPAT5 | ENSG00000155189 | ENST00000285518 |
| CTD-2510F5.6 | ENSG00000265303 | ENST00000577660 |
| TRERF1 | ENSG00000124496 | ENST00000541110 |
| KCNB1 | ENSG00000158445 | ENST00000371741 |
| UNKL | ENSG00000059145 | ENST00000403703 |
| ZNF286B | ENSG00000249459 | ENST00000285274 |
| FBXO38 | ENSG00000145868 | ENST00000340253 |
| DDX20 | ENSG00000064703 | ENST00000369702 |
| FEZF1 | ENSG00000128610 | ENST00000442488 |
| GRAP2 | ENSG00000100351 | ENST00000344138 |
| ILDR2 | ENSG00000143195 | ENST00000271417 |
| WNT5A | ENSG00000114251 | ENST00000474267 |
| MEF2A | ENSG00000068305 | ENST00000354410 |
| JAKMIP3 | ENSG00000188385 | ENST00000298622 |
| ZNF347 | ENSG00000197937 | ENST00000334197 |
| FSIP1 | ENSG00000150667 | ENST00000350221 |
| LYPLAL1 | ENSG00000143353 | ENST00000366928 |
| CGNL1 | ENSG00000128849 | ENST00000281282 |
| STARD4 | ENSG00000164211 | ENST00000296632 |
| ILDR1 | ENSG00000145103 | ENST00000273691 |
| LEPROTL1 | ENSG00000104660 | ENST00000321250 |
| TCP11L1 | ENSG00000176148 | ENST00000528107 |
| ZNF562 | ENSG00000171466 | ENST00000293648 |
| TBC1D9 | ENSG00000109436 | ENST00000442267 |
| SPIN4 | ENSG00000186767 | ENST00000335144 |
| PPP1R12B | ENSG00000077157 | ENST00000608999 |
| IYD | ENSG00000009765 | ENST00000344419 |
| LSM11 | ENSG00000155858 | ENST00000286307 |
| UBFD1 | ENSG00000103353 | ENST00000395878 |
| NLGN4Y | ENSG00000165246 | ENST00000382872 |
| C11orf65 | ENSG00000166323 | ENST00000527531 |
| LPGAT1 | ENSG00000123684 | ENST00000366997 |
| RP11-156P1.2 | ENSG00000262633 | ENST00000571841 |
| MPC1 | ENSG00000060762 | ENST00000360961 |
| FADS1 | ENSG00000149485 | ENST00000350997 |
| VNN2 | ENSG00000112303 | ENST00000422400 |
| ZFP14 | ENSG00000142065 | ENST00000270001 |
| KLF7 | ENSG00000118263 | ENST00000309446 |
| PDE7B | ENSG00000171408 | ENST00000308191 |
| KDM4D | ENSG00000186280 | ENST00000335080 |
| GOT1 | ENSG00000120053 | ENST00000370508 |
| CHL1 | ENSG00000134121 | ENST00000256509 |
| XG | ENSG00000124343 | ENST00000426774 |
| AKAP7 | ENSG00000118507 | ENST00000263050 |
| ACTR1A | ENSG00000138107 | ENST00000487599 |
| FBXW2 | ENSG00000119402 | ENST00000608872 |
| GLE1 | ENSG00000119392 | ENST00000309971 |
| DDX4 | ENSG00000152670 | ENST00000503129 |
| MRPS25 | ENSG00000131368 | ENST00000253686 |
| TAB3 | ENSG00000157625 | ENST00000467136 |
| SPG11 | ENSG00000104133 | ENST00000261866 |
| CUL4A | ENSG00000139842 | ENST00000326335 |
| RNF175 | ENSG00000145428 | ENST00000503694 |
| SON | ENSG00000159140 | ENST00000381679 |
| IMPG1 | ENSG00000112706 | ENST00000369963 |
| FAM184B | ENSG00000047662 | ENST00000265018 |
| GOLGA6L1 | ENSG00000197414 | ENST00000316397 |
| DTWD2 | ENSG00000169570 | ENST00000304058 |
| NPAP1 | ENSG00000185823 | ENST00000329468 |
| HNRNPA3 | ENSG00000170144 | ENST00000411529 |
| FRMD5 | ENSG00000171877 | ENST00000417257 |
| NFATC2 | ENSG00000101096 | ENST00000371564 |
| BMI1 | ENSG00000168283 | ENST00000376663 |
| PRUNE2 | ENSG00000106772 | ENST00000376717 |
| NDUFB5 | ENSG00000136521 | ENST00000493866 |
| RAN | ENSG00000132341 | ENST00000543796 |
| MBOAT1 | ENSG00000172197 | ENST00000541730 |
| MCM8 | ENSG00000125885 | ENST00000265187 |
| SPRYD4 | ENSG00000176422 | ENST00000338146 |
| KCNA2 | ENSG00000177301 | ENST00000485317 |
| PRKCI | ENSG00000163558 | ENST00000295797 |
| MOK | ENSG00000080823 | ENST00000521766 |
| TMEM43 | ENSG00000170876 | ENST00000306077 |
| GEN1 | ENSG00000178295 | ENST00000381254 |
| COL4A4 | ENSG00000081052 | ENST00000396625 |
| MTHFD2L | ENSG00000163738 | ENST00000423607 |
| CCNY | ENSG00000108100 | ENST00000374706 |
| TRIQK | ENSG00000205133 | ENST00000521988 |
| PRPF38A | ENSG00000134748 | ENST00000257181 |
| DIDO1 | ENSG00000101191 | ENST00000395340 |
| SLC35A1 | ENSG00000164414 | ENST00000369552 |
| HERC3 | ENSG00000138641 | ENST00000402738 |
| ANKRD62 | ENSG00000181626 | ENST00000314074 |
| UNC50 | ENSG00000115446 | ENST00000393493 |
| ALDH1L2 | ENSG00000136010 | ENST00000258494 |
| ARL4A | ENSG00000122644 | ENST00000396663 |
| NOMO3 | ENSG00000103226 | ENST00000575225 |
| SIRPB1 | ENSG00000101307 | ENST00000381605 |
| MCM9 | ENSG00000111877 | ENST00000316316 |
| TVP23C | ENSG00000175106 | ENST00000584811 |
| PRRC1 | ENSG00000164244 | ENST00000442138 |
| CCDC67 | ENSG00000165325 | ENST00000531448 |
| **Shared prediction** |  |  |
| Gene symbol | Gene symbol | Gene symbol |
| A1CF | ARL14EPL | CACHD1 |
| AAK1 | ARL2BP | CACUL1 |
| ABAT | ARL3 | CALHM1 |
| ABCA1 | ARL5A | CALU |
| ABCC9 | ARL6IP6 | CAMSAP2 |
| ABI2 | ASAP1 | CAND1 |
| AC068987.1 | ASF1A | CASC4 |
| ACE2 | ASH1L | CASD1 |
| ACTR1A | ASXL3 | CASK |
| ACVR1C | ATAD2B | CASKIN1 |
| ACVR2A | ATMIN | CASR |
| ADAMTS3 | ATP11B | CBL |
| ADCY2 | ATP11C | CBX4 |
| ADCY9 | ATP2A2 | CBX5 |
| ADD3 | ATP6V0A2 | CCDC82 |
| ADIPOR2 | ATXN1 | CCNA2 |
| AEBP2 | ATXN1L | CCNJ |
| AFF1 | AUTS2 | CCNT2 |
| AFF3 | AVL9 | CCNYL1 |
| AFF4 | B3GNT1 | CCSER1 |
| AGAP1 | B3GNT2 | CDC27 |
| AGFG1 | BACH2 | CDC42EP3 |
| AHNAK | BAG5 | CDC73 |
| AMBRA1 | BAG6 | CDH11 |
| AMFR | BAP1 | CDH20 |
| AMOTL2 | BASP1 | CDH6 |
| ANGEL2 | BAZ2B | CDH7 |
| ANGPTL3 | BBX | CDK12 |
| ANK3 | BCL11B | CDK13 |
| ANKRD28 | BCL2 | CDK16 |
| ANLN | BCL9 | CDK17 |
| ANO5 | BDP1 | CDK19 |
| ANO6 | BHLHE41 | CDK2 |
| ANP32B | BICC1 | CDKN1B |
| AP1S2 | BMI1 | CDR2 |
| APAF1 | BNC2 | CDR2L |
| ARGLU1 | BPTF | CDYL |
| ARHGAP20 | BRWD1 | CDYL2 |
| ARHGAP26 | BRWD3 | CEBPD |
| ARHGAP6 | BTF3L4 | CECR2 |
| ARHGDIA | C10orf118 | CELF1 |
| ARHGEF17 | C11orf87 | CELF2 |
| ARID4A | C16orf52 | CEP350 |
| ARID4B | C16orf72 | CEP41 |
| ARID5B | C4orf32 | CEP85L |
| ARIH1 | C6orf120 | CEP97 |
| ARIH2 | C6orf62 | CERS6 |
| CFL2 | DDIT4 | ERN1 |
| CGGBP1 | DDX26B | ESRP1 |
| CHD2 | DDX3X | ESRRG |
| CHD9 | DDX3Y | ETF1 |
| CHMP5 | DENND2C | ETS1 |
| CHN2 | DENND5A | ETS2 |
| CHRDL1 | DENND5B | ETV5 |
| CHST2 | DESI1 | EVI5 |
| CHST9 | DGKA | FAM107B |
| CHSY1 | DGKH | FAM118B |
| CHSY3 | DHX36 | FAM126A |
| CITED2 | DHX9 | FAM168B |
| CKAP4 | DIRAS2 | FAM178A |
| CKLF | DIXDC1 | FAM179B |
| CLASP1 | DLC1 | FAM19A5 |
| CLASP2 | DLGAP2 | FAM219A |
| CLIC4 | DMD | FAM49B |
| CLIP1 | DMRT2 | FAM60A |
| CLIP2 | DNAJB14 | FAM63B |
| CLOCK | DNAJB5 | FAM8A1 |
| CNEP1R1 | DNAJB9 | FARP1 |
| CNKSR3 | DNAJC3 | FAT3 |
| CNN3 | DNAJC5 | FBXL16 |
| CNOT6 | DNMT3A | FBXL17 |
| CNOT7 | DNMT3B | FBXO22 |
| CNST | DOCK4 | FBXO30 |
| CNTFR | DOT1L | FBXO33 |
| CNTN4 | DPY19L1 | FBXW11 |
| COL4A3 | DR1 | FBXW2 |
| COL4A3BP | DTNA | FBXW7 |
| COPS2 | DUSP1 | FERMT2 |
| COPS8 | DYRK2 | FEZ2 |
| CORO1C | DZIP1 | FGD1 |
| CPED1 | E2F3 | FGF12 |
| CPT1A | EFNA1 | FGF18 |
| CREB5 | EFNA5 | FIGN |
| CRKL | EFNB2 | FLI1 |
| CRTAP | EGLN1 | FLJ20373 |
| CSMD3 | EIF2S1 | FLT1 |
| CSNK1G3 | EIF3J | FMR1 |
| CSRNP3 | EIF4E | FN1 |
| CTBP2 | EIF4E3 | FNBP4 |
| CTD-2510F5.6 | EIF5B | FNDC3B |
| CTDSPL2 | ELAVL2 | FOSL2 |
| CTNND2 | ELAVL4 | FOXF1 |
| CUL4A | ELF2 | FOXF2 |
| CUL5 | ELK3 | FOXG1 |
| CXorf23 | ELK4 | FOXK1 |
| CYP1B1 | ELL2 | FOXN2 |
| CYTH1 | ELMOD2 | FOXP1 |
| CYTH3 | EP300 | FREM2 |
| DACH1 | EPS8 | FRMD4A |
| DACT1 | ERBB4 | FRMD4B |
| DCAF17 | ERG | FRMD5 |
| DCAF5 | ERGIC2 | FRMD6 |
| DCBLD2 | ERI1 | FRS2 |
| FSTL1 | HIPK1 | KIAA0355 |
| FUBP1 | HIPK2 | KIAA0430 |
| FUBP3 | HIPK3 | KIAA0895 |
| FUT4 | HIVEP3 | KIAA1430 |
| FXR1 | HLF | KIAA1432 |
| FXR2 | HMBOX1 | KIAA1456 |
| FYN | HMGB3 | KIAA1462 |
| G6PC | HNF1B | KIAA1468 |
| GAB1 | HNRNPD | KIAA2018 |
| GABBR2 | HNRNPK | KIF13A |
| GABPA | HNRNPU | KIF26B |
| GABRB3 | HOOK1 | KLF10 |
| GAD2 | HOXA5 | KLF12 |
| GAL3ST1 | HS2ST1 | KLF13 |
| GAN | HS3ST1 | KLF3 |
| GATA2 | HS3ST3A1 | KLF4 |
| GATA4 | HSPA13 | KLF9 |
| GATAD2B | HSPA9 | KLHL14 |
| GATSL2 | IAH1 | KLHL3 |
| GDI2 | ICK | KMT2C |
| GFI1 | IER5 | KMT2E |
| GIGYF1 | IFIT5 | KRR1 |
| GIT2 | IGF2R | KRT80 |
| GJC1 | IGSF3 | KSR1 |
| GLCCI1 | IKBKB | LAMC1 |
| GLI3 | IMPAD1 | LARP1B |
| GLIS2 | ING2 | LATS2 |
| GMFB | INHBA | LBR |
| GNAI3 | INPP4A | LCA5 |
| GNAQ | INTS8 | LCOR |
| GNPDA1 | IPO8 | LEMD3 |
| GOLGA7 | IRS1 | LEPROTL1 |
| GOLIM4 | ITM2B | LFNG |
| GPC6 | ITPR1 | LHFP |
| GPM6A | JAKMIP3 | LHX9 |
| GPR107 | JAZF1 | LIMK1 |
| GPR158 | JKAMP | LMAN1 |
| GPR173 | JMY | LMO4 |
| GPR27 | JUN | LMO7 |
| GPR63 | KANK1 | LOX |
| GPRIN3 | KANK2 | LPAR1 |
| GRAP2 | KATNAL1 | LPIN1 |
| GRID2 | KBTBD6 | LPIN2 |
| GRIN2A | KBTBD8 | LPPR1 |
| GRIP1 | KCNB1 | LPPR4 |
| GSE1 | KCND2 | LRIG1 |
| GTF2E1 | KCND3 | LRP1B |
| GUCY1A3 | KCNJ15 | LRP4 |
| GXYLT1 | KCNQ4 | LRRC8A |
| HCCS | KCTD16 | LRRTM3 |
| HCFC2 | KDELC1 | LYSMD3 |
| HCN1 | KDR | MAFG |
| HDAC4 | KHDRBS1 | MAP1B |
| HDHD2 | KIAA0040 | MAP2 |
| HECW2 | KIAA0087 | MAP3K1 |
| HEG1 | KIAA0101 | MAP3K5 |
| MAP4K3 | NCOA7 | PAPD5 |
| MAP4K4 | NCOR2 | PAPOLG |
| MAPK1IP1L | NDN | PAQR3 |
| MAPRE1 | NDNF | PAQR5 |
| MARCH6 | NEDD1 | PARD3B |
| MARCH8 | NEDD4L | PARD6B |
| MARCKS | NEGR1 | PBX3 |
| MATR3 | NEK10 | PCDH19 |
| MBD5 | NEO1 | PCDH8 |
| MBLAC2 | NFASC | PCMTD1 |
| MBNL1 | NFIA | PCNP |
| MBNL3 | NFIB | PCNX |
| MBOAT2 | NFYA | PCSK2 |
| MCC | NGEF | PDE7B |
| MCFD2 | NIPBL | PDIK1L |
| MED1 | NKD1 | PDS5B |
| MED13 | NLGN4X | PEAK1 |
| MED6 | NOG | PFDN4 |
| MEF2D | NOTCH1 | PHACTR3 |
| MEX3B | NOVA1 | PHF12 |
| MFAP5 | NOVA2 | PHF21A |
| MFHAS1 | NPNT | PHF21B |
| MGA | NPTX1 | PHF6 |
| MGAT2 | NR2C2 | PHLDB1 |
| MIB1 | NR3C1 | PHTF2 |
| MIEF1 | NR5A2 | PI4K2B |
| MIER3 | NRBF2 | PI4KB |
| MKL2 | NRBP1 | PIGM |
| MMD | NRG1 | PIK3CA |
| MMD2 | NRIP1 | PIK3CB |
| MMP16 | NRP2 | PIKFYVE |
| MOB4 | NTF3 | PIN1 |
| MOCS1 | NTPCR | PIP4K2A |
| MOSPD2 | NUDT3 | PISD |
| MPRIP | NUDT4 | PITPNM3 |
| MRPS25 | NUFIP2 | PKIA |
| MSL2 | NUMB | PLCG1 |
| MSN | NUP153 | PLCL1 |
| MTF2 | NUP35 | PLCXD3 |
| MTFR1 | NYAP1 | PLK2 |
| MTSS1L | OCLN | PLXNA2 |
| MXD4 | ONECUT2 | PLXNA4 |
| MXI1 | ORMDL3 | PLXNC1 |
| MYB | OSBPL11 | PMAIP1 |
| MYCN | OSMR | POLK |
| MYO9A | OSTM1 | POU6F1 |
| MYT1 | OTUD4 | PPAP2B |
| MYZAP | OXR1 | PPAPDC2 |
| N4BP2 | PAG1 | PPFIA1 |
| NAB1 | PAIP2 | PPFIBP1 |
| NANOS1 | PAK3 | PPM1F |
| NAPB | PAK6 | PPP1CB |
| NBR1 | PAK7 | PPP1R10 |
| NCAM1 | PALM2 | PPP1R12B |
| NCOA2 | PAM | PPP1R18 |
| NCOA3 | PAN3 | PPP1R9A |
| PPP1R9B | RBM20 | SESN1 |
| PPP2R2C | RDH10 | SESN3 |
| PPP2R5C | RECK | SETD7 |
| PPP2R5E | REEP1 | SFXN1 |
| PPP4R2 | REL | SGIP1 |
| PPP6C | RELN | SH3GL1 |
| PPP6R3 | REV1 | SH3PXD2A |
| PRDM1 | RFX7 | SHC1 |
| PRDM16 | RGL1 | SHROOM4 |
| PRKACB | RHOA | SIAH1 |
| PRKAR2B | RHOT1 | SIK2 |
| PRKCA | RIMKLB | SIN3A |
| PRKG1 | RIMS2 | SIX1 |
| PROX1 | RIMS3 | SIX3 |
| PRPF38B | RIPK2 | SLC14A1 |
| PRRG4 | RLF | SLC16A10 |
| PSAT1 | RLIM | SLC16A2 |
| PSIP1 | RND3 | SLC1A2 |
| PSPH | RNF11 | SLC23A2 |
| PTAR1 | RNF169 | SLC24A4 |
| PTBP1 | RNF2 | SLC2A14 |
| PTCH1 | RNF38 | SLC30A10 |
| PTDSS1 | ROBO2 | SLC30A5 |
| PTEN | ROCK2 | SLC31A1 |
| PTP4A1 | RPRD1A | SLC35E2B |
| PTPN11 | RPS6KA3 | SLC38A2 |
| PTPN12 | RPS6KB1 | SLC39A14 |
| PTPN13 | RSPRY1 | SLC4A4 |
| PTPN14 | RTF1 | SLC4A7 |
| PTPN21 | RUSC2 | SLC5A3 |
| PUM2 | S100PBP | SLC6A1 |
| PVRL4 | SAMD8 | SLC6A11 |
| QKI | SAR1B | SLIT2 |
| R3HDM2 | SBF1 | SLITRK1 |
| RAB11FIP2 | SBSPON | SLK |
| RAB18 | SCAMP1 | SMAD2 |
| RAB21 | SCD | SMAD9 |
| RAB23 | SCN2A | SMARCAD1 |
| RAB37 | SCN3B | SMIM5 |
| RAB4A | SCN5A | SMURF1 |
| RAB7A | SCN8A | SMURF2 |
| RAB8B | SCOC | SNAI2 |
| RABEP1 | SCRT2 | SNAP25 |
| RAC1 | SDC2 | SNAPC1 |
| RALGPS2 | SDK2 | SNTB2 |
| RANBP10 | SEC23A | SNX30 |
| RANBP9 | SEC24A | SOAT1 |
| RAP1B | SEC61A2 | SOCS6 |
| RAP2C | SECISBP2L | SORT1 |
| RAPGEF2 | SELK | SOX1 |
| RASA2 | SEMA3F | SOX2 |
| RASSF8 | SEMA6D | SOX5 |
| RBFOX1 | SENP5 | SOX6 |
| RBFOX2 | SEPHS1 | SPAG9 |
| RBFOX3 | SERINC1 | SPAST |
| RBM12B | SERPINI1 | SPATS2L |
| SPG20 | TMEM136 | VLDLR |
| SPRED1 | TMEM14A | VTI1A |
| SPRYD4 | TMEM17 | VTI1B |
| SPTSSA | TMEM170A | WAPAL |
| SRF | TMEM170B | WASF1 |
| SRGAP1 | TMEM178A | WASF3 |
| SRP72 | TMEM189-UBE2V1 | WDFY3 |
| SRSF1 | TMEM229B | WDR12 |
| SRSF10 | TMEM245 | WDR45B |
| SRSF2 | TMEM33 | WDR82 |
| ST3GAL2 | TMOD3 | WDR91 |
| ST6GALNAC5 | TMX4 | WIPF1 |
| STARD13 | TOB1 | WIPF3 |
| STRN | TP53INP1 | WNK3 |
| STRN3 | TP73 | WWC3 |
| STX1A | TPCN1 | XIAP |
| STXBP6 | TRAPPC8 | XKR4 |
| STYX | TRHDE | XKR6 |
| SUGT1 | TRIM2 | XKR8 |
| SULF1 | TRIM33 | YOD1 |
| SURF4 | TRIM62 | YPEL2 |
| SUV420H1 | TRIM71 | YWHAB |
| SUZ12 | TRIO | YWHAG |
| SWAP70 | TRPM3 | YWHAQ |
| SYDE1 | TSC22D1 | ZBTB38 |
| SYNCRIP | TSC22D2 | ZBTB5 |
| SYNJ1 | TTC5 | ZBTB7C |
| SYT1 | TUBB | ZBTB8A |
| SYVN1 | TWISTNB | ZBTB8B |
| TAF12 | TXLNG | ZC3H4 |
| TAF4 | UBA6 | ZC3H6 |
| TAOK1 | UBE2B | ZCCHC14 |
| TAOK3 | UBE2D1 | ZCCHC24 |
| TARDBP | UBE2I | ZDHHC17 |
| TBC1D12 | UBE2J1 | ZEB1 |
| TBC1D22B | UBE2R2 | ZEB2 |
| TBCEL | UBE2V1 | ZFAND5 |
| TBK1 | UBE2W | ZFAND6 |
| TBL1XR1 | UBN2 | ZFHX4 |
| TBX5 | UBQLN1 | ZFPM2 |
| TCAIM | UGCG | ZFX |
| TCF4 | UHRF1BP1 | ZIC3 |
| TEAD1 | ULK2 | ZMAT3 |
| TEC | UNC119B | ZMYM4 |
| TENM1 | USH1G | ZNF148 |
| TFAP2A | USP25 | ZNF217 |
| THAP1 | USP27X | ZNF236 |
| THRA | USP31 | ZNF281 |
| THRB | USP46 | ZNF292 |
| THSD7A | USP47 | ZNF362 |
| TIMP2 | USP6NL | ZNF365 |
| TJP1 | VASH1 | ZNF395 |
| TLL2 | VASH2 | ZNF423 |
| TLN1 | VAT1L | ZNF516 |
| TLN2 | VCPIP1 | ZNF532 |
| TMCC1 | VEGFA | ZNF652 |
| ZNF662 | ZNF711 | ZSWIM4 |
| ZNF697 | ZNF771 | ZYG11B |
