# Supplementary material for: MicroRNA-200b/c-3p regulate epithelial plasticity and inhibit cutaneous wound healing by modulating TGF-β-mediated RAC1 signaling
Source: Cell Death Dis. 2020 Oct 29;11(10):931. doi: 10.1038/s41419-020-03132-2 (PMC7596237; doi:10.1038/s41419-020-03132-2)
Supplement: Supplementary file 4 — Supplementary Table 3 [file 41419_2020_3132_MOESM4_ESM.docx]

| **Supplementary Table 3. Enriched pathways for predicted miR-200b/c-3p target genes** | | | | | |
| --- | --- | --- | --- | --- | --- |
| DAVID bioinformatics resources | | |  |  |  |
| <https://david.ncifcrf.gov/> | |  |  |  |  |
| **BIOCARTA_PATHWAY** | |  |  |  |  |
| **Term** | **Count** | **%** | **PValue** | **Genes** | **Fold Enrichment** |
| h_rac1Pathway:Rac 1 cell motility signaling pathway | 8 | 0.8256 | 0.00193 | LIMK1, PPP1R12B, WASF1, MAP3K1, RAC1, TRIO, PIK3CA, RPS6KB1 | 4.12567 |
|  |  |  |  |  |  |
| h_pyk2Pathway:Links between Pyk2 and Map Kinases | 8 | 0.8256 | 0.00654 | PRKCA, CRKL, PLCG1, GNAQ, JUN, MAP3K1, RAC1, SHC1 | 3.38895 |
|  |  |  |  |  |  |
| h_PparaPathway:Mechanism of Gene Regulation by Peroxisome Proliferators via PPARa(alpha) | 11 | 1.1352 | 0.00901 | PRKCA, PRKAR2B, EP300, DUSP1, JUN, PIK3CA, PRKACB, NCOR2, NRIP1, CITED2, MED1 | 2.50912 |
|  |  |  |  |  |  |
| h_vegfPathway:VEGF, Hypoxia, and Angiogenesis | 8 | 0.8256 | 0.01174 | PRKCA, FLT1, PLCG1, EIF2S1, VEGFA, PIK3CA, SHC1, KDR | 3.06098 |
|  |  |  |  |  |  |
| h_egfPathway:EGF Signaling Pathway | 7 | 0.7224 | 0.02091 | PRKCA, PLCG1, JUN, MAP3K1, PIK3CA, SHC1, SRF | 3.07516 |
|  |  |  |  |  |  |
| h_pdgfPathway:PDGF Signaling Pathway | 7 | 0.7224 | 0.02483 | PRKCA, PLCG1, JUN, MAP3K1, PIK3CA, SHC1, SRF | 2.96533 |
|  |  |  |  |  |  |
| h_igf1Pathway:IGF-1 Signaling Pathway | 6 | 0.6192 | 0.02594 | JUN, PIK3CA, SHC1, IRS1, SRF, PTPN11 | 3.38895 |
|  |  |  |  |  |  |
| h_insulinPathway:Insulin Signaling Pathway | 6 | 0.6192 | 0.02594 | JUN, PIK3CA, SHC1, IRS1, SRF, PTPN11 | 3.38895 |
|  |  |  |  |  |  |
| h_ccr3Pathway:CCR3 signaling in Eosinophils | 6 | 0.6192 | 0.03748 | PRKCA, GNAQ, ROCK2, LIMK1, PPP1R12B, RHOA | 3.09426 |
|  |  |  |  |  |  |
| h_carm-erPathway:CARM1 and Regulation of the Estrogen Receptor | 7 | 0.7224 | 0.03945 | GTF2E1, HDAC4, EP300, GRIP1, NCOR2, NRIP1, MED1 | 2.67836 |
|  |  |  |  |  |  |
| h_eif4Pathway:Regulation of eIF4e and p70 S6 Kinase | 6 | 0.6192 | 0.04425 | PRKCA, EIF4E, PIK3CA, RPS6KB1, PTEN, IRS1 | 2.96533 |
|  |  |  |  |  |  |
|  |  |  |  |  |  |
|  |  |  |  |  |  |
|  |  |  |  |  |  |
|  |  |  |  |  |  |
|  |  |  |  |  |  |
|  |  |  |  |  |  |
|  |  |  |  |  |  |
|  |  |  |  |  |  |
|  |  |  |  |  |  |
| **KEGG_PATHWAY** | |  |  |  |  |
| **Term** | **Count** | **%** | **PValue** | **Genes** | **Fold Enrichment** |
| hsa04722:Neurotrophin signaling pathway | 23 | 2.3736 | 1.6E-07 | NTF3, PIK3CB, IRS1, TP73, PTPN11, MAP3K5, RPS6KA3, CRKL, PLCG1, JUN, BCL2, MAP3K1, GAB1, RAC1, RHOA, RIPK2, SORT1, PIK3CA, SHC1, RAP1B, IKBKB, FRS2, ARHGDIA | 3.69321 |
|  |  |  |  |  |  |
| hsa04014:Ras signaling pathway | 29 | 2.9928 | 1.3E-05 | FGF18, TBK1, EFNA1, FGF12, RGL1, PAK6, REL, PAK3, GAB1, RAC1, RHOA, PIK3CA, SHC1, PRKACB, RASA2, PRKCA, FLT1, PIK3CB, GRIN2A, KDR, PTPN11, PLCG1, ETS1, ETS2, VEGFA, RAP1B, EFNA5, IKBKB, KSR1 | 2.47256 |
|  |  |  |  |  |  |
| hsa04510:Focal adhesion | 27 | 2.7864 | 1.9E-05 | PRKCA, COL4A3, TLN1, FLT1, XIAP, TLN2, PIK3CB, ROCK2, PPP1R12B, PTEN, PPP1CB, KDR, PAK6, CRKL, FYN, PAK3, JUN, BCL2, RAC1, VEGFA, RHOA, PIK3CA, SHC1, RAP1B, RELN, LAMC1, FN1 | 2.52554 |
|  |  |  |  |  |  |
| hsa04360:Axon guidance | 20 | 2.064 | 2.5E-05 | NGEF, PLXNC1, GNAI3, ROCK2, EFNA1, PLXNA2, LIMK1, EFNB2, SLIT2, PAK6, SEMA6D, PAK3, FYN, CFL2, SEMA3F, RAC1, RHOA, ROBO2, EFNA5, SRGAP1 | 3.03447 |
|  |  |  |  |  |  |
| hsa05211:Renal cell carcinoma | 14 | 1.4448 | 2.6E-05 | PIK3CB, EGLN1, PTPN11, PAK6, CRKL, EP300, PAK3, ETS1, JUN, RAC1, VEGFA, GAB1, PIK3CA, RAP1B | 4.08734 |
|  |  |  |  |  |  |
| hsa04120:Ubiquitin mediated proteolysis | 20 | 2.064 | 7.2E-05 | SYVN1, XIAP, CBL, UBE2J1, UBA6, UBE2I, CDC27, UBE2B, UBE2R2, FBXW7, CUL5, CUL4A, MAP3K1, UBE2W, SMURF2, SIAH1, NEDD4L, SMURF1, UBE2D1, FBXW11 | 2.81298 |
|  |  |  |  |  |  |
| hsa04015:Rap1 signaling pathway | 26 | 2.6832 | 7.5E-05 | FGF18, TLN1, ADCY2, GNAI3, EFNA1, TLN2, LPAR1, FGF12, RAC1, RHOA, PIK3CA, RAPGEF2, PRKCA, PARD6B, FLT1, PIK3CB, GRIN2A, KDR, DOCK4, CRKL, GNAQ, PLCG1, ADCY9, VEGFA, RAP1B, EFNA5 | 2.38567 |
|  |  |  |  |  |  |
| hsa04012:ErbB signaling pathway | 15 | 1.548 | 0.00013 | PRKCA, ERBB4, PIK3CB, CBL, RPS6KB1, PAK6, CRKL, CDKN1B, PLCG1, PAK3, JUN, GAB1, PIK3CA, SHC1, NRG1 | 3.32223 |
|  |  |  |  |  |  |
| hsa04151:PI3K-Akt signaling pathway | 35 | 3.612 | 0.00019 | FGF18, EFNA1, OSMR, PPP2R5C, RPS6KB1, FGF12, LPAR1, PTEN, BCL2, RAC1, PIK3CA, MYB, PPP2R2C, FN1, PRKCA, COL4A3, FLT1, PIK3CB, YWHAB, CREB5, IRS1, CDK2, KDR, DDIT4, G6PC, YWHAG, EIF4E, CDKN1B, VEGFA, YWHAQ, EFNA5, PPP2R5E, RELN, LAMC1, IKBKB | 1.95482 |
|  |  |  |  |  |  |
| hsa05205:Proteoglycans in cancer | 24 | 2.4768 | 0.00024 | PRKCA, ERBB4, ROCK2, PIK3CB, PPP1R12B, CBL, RPS6KB1, PPP1CB, SDC2, ITPR1, KDR, PTPN11, PLCG1, ANK3, GAB1, RAC1, VEGFA, RHOA, PIK3CA, PTCH1, MSN, PRKACB, FRS2, FN1 | 2.31227 |
|  |  |  |  |  |  |
| hsa04919:Thyroid hormone signaling pathway | 17 | 1.7544 | 0.00026 | PRKCA, THRA, THRB, PIK3CB, SLC16A10, MED13, SLC16A2, NOTCH1, EP300, NCOA2, SIN3A, NCOA3, PLCG1, GATA4, PIK3CA, PRKACB, MED1 | 2.84845 |
|  |  |  |  |  |  |
| hsa05161:Hepatitis B | 19 | 1.9608 | 0.00046 | PRKCA, E2F3, TBK1, PIK3CB, YWHAB, CREB5, PTEN, CDK2, EP300, CDKN1B, DDX3X, JUN, BCL2, MAP3K1, YWHAQ, PIK3CA, APAF1, IKBKB, CCNA2 | 2.52489 |
|  |  |  |  |  |  |
| hsa04070:Phosphatidylinositol signaling system | 15 | 1.548 | 0.00047 | PRKCA, IMPAD1, PIK3CB, SYNJ1, DGKH, PI4KB, PI4K2B, PTEN, ITPR1, DGKA, PLCG1, PIKFYVE, PIK3CA, INPP4A, PIP4K2A | 2.94932 |
|  |  |  |  |  |  |
| hsa04150:mTOR signaling pathway | 11 | 1.1352 | 0.00068 | PRKCA, RPS6KA3, EIF4E, PIK3CB, ULK2, PIK3CA, RPS6KB1, IKBKB, PTEN, IRS1, DDIT4 | 3.65445 |
|  |  |  |  |  |  |
| hsa04611:Platelet activation | 17 | 1.7544 | 0.00103 | TLN1, ADCY2, GNAI3, ROCK2, TLN2, PIK3CB, PRKG1, PPP1CB, ITPR1, ADCY9, GNAQ, FYN, RHOA, PIK3CA, GUCY1A3, RAP1B, PRKACB | 2.51978 |
|  |  |  |  |  |  |
| hsa05200:Pathways in cancer | 36 | 3.7152 | 0.00103 | FGF18, E2F3, GNAI3, ADCY2, XIAP, EGLN1, FGF12, LPAR1, PTEN, GLI3, BCL2, RAC1, RHOA, PIK3CA, PRKACB, FN1, PRKCA, COL4A3, CTBP2, PIK3CB, ROCK2, CBL, SMAD2, CDK2, CRKL, CDKN1B, EP300, ADCY9, PLCG1, GNAQ, ETS1, JUN, VEGFA, PTCH1, LAMC1, IKBKB | 1.76509 |
|  |  |  |  |  |  |
| hsa04666:Fc gamma R-mediated phagocytosis | 13 | 1.3416 | 0.00118 | PRKCA, CRKL, PLCG1, WASF3, LIMK1, PIK3CB, CFL2, WASF1, RAC1, ASAP1, PIK3CA, MARCKS, RPS6KB1 | 2.98209 |
|  |  |  |  |  |  |
| hsa04071:Sphingolipid signaling pathway | 16 | 1.6512 | 0.00125 | PRKCA, GNAI3, ROCK2, PIK3CB, PPP2R5C, CERS6, PTEN, MAP3K5, GNAQ, FYN, BCL2, RAC1, RHOA, PIK3CA, PPP2R5E, PPP2R2C | 2.56919 |
|  |  |  |  |  |  |
| hsa05222:Small cell lung cancer | 13 | 1.3416 | 0.00132 | COL4A3, E2F3, CDKN1B, XIAP, PIK3CB, BCL2, PIK3CA, APAF1, LAMC1, IKBKB, PTEN, CDK2, FN1 | 2.94701 |
|  |  |  |  |  |  |
| hsa04114:Oocyte meiosis | 15 | 1.548 | 0.00165 | ADCY2, PPP2R5C, YWHAB, CDC27, PPP1CB, CDK2, ITPR1, YWHAG, RPS6KA3, SLK, ADCY9, YWHAQ, PPP2R5E, PRKACB, FBXW11 | 2.60391 |
|  |  |  |  |  |  |
| hsa05206:MicroRNAs in cancer | 28 | 2.8896 | 0.00167 | BMI1, E2F3, CYP1B1, ZEB2, ZEB1, TRIM71, PTEN, HNRNPK, BCL2, RHOA, SHC1, DNMT3B, RECK, PRKCA, DNMT3A, UBE2I, MMP16, IRS1, DDIT4, NOTCH1, CDKN1B, EP300, CRKL, PLCG1, VEGFA, MARCKS, ZFPM2, IKBKB | 1.88647 |
|  |  |  |  |  |  |
| hsa05203:Viral carcinogenesis | 22 | 2.2704 | 0.00202 | PIK3CB, YWHAB, CREB5, PMAIP1, SRF, CDK2, GTF2E1, HDAC4, YWHAG, EP300, HNRNPK, CDKN1B, DDX3X, REL, JUN, RAC1, RHOA, YWHAQ, PIK3CA, PRKACB, CCNA2, RASA2 | 2.06788 |
|  |  |  |  |  |  |
| hsa04141:Protein processing in endoplasmic reticulum | 19 | 1.9608 | 0.00272 | SEC23A, SYVN1, SEC24A, CKAP4, UBE2J1, LMAN1, UBQLN1, MARCH6, MAP3K5, EIF2S1, BCL2, ERN1, DNAJC5, YOD1, AMFR, DNAJC3, SAR1B, UBE2D1, SEC61A2 | 2.16633 |
|  |  |  |  |  |  |
| hsa00562:Inositol phosphate metabolism | 11 | 1.1352 | 0.00333 | IMPAD1, PLCG1, PIK3CB, SYNJ1, PIKFYVE, PIK3CA, PI4K2B, PI4KB, INPP4A, PIP4K2A, PTEN | 2.98532 |
|  |  |  |  |  |  |
| hsa04727:GABAergic synapse | 12 | 1.2384 | 0.00415 | PRKCA, PLCL1, GAD2, GNAI3, SLC38A2, ADCY2, SLC6A1, GABRB3, ADCY9, ABAT, PRKACB, GABBR2 | 2.72032 |
|  |  |  |  |  |  |
| hsa04540:Gap junction | 12 | 1.2384 | 0.00542 | PRKCA, TUBB, TJP1, GNAI3, ADCY2, ADCY9, GNAQ, GUCY1A3, PRKACB, LPAR1, PRKG1, ITPR1 | 2.62758 |
|  |  |  |  |  |  |
| hsa04810:Regulation of actin cytoskeleton | 21 | 2.1672 | 0.0059 | FGD1, FGF18, ROCK2, LIMK1, PIK3CB, WASF1, PPP1R12B, ABI2, FGF12, PPP1CB, PAK6, CRKL, PAK3, CFL2, RAC1, PIKFYVE, RHOA, PIK3CA, MSN, PIP4K2A, FN1 | 1.92689 |
|  |  |  |  |  |  |
| hsa04066:HIF-1 signaling pathway | 12 | 1.2384 | 0.01035 | PRKCA, EIF4E, CDKN1B, FLT1, EP300, PLCG1, PIK3CB, BCL2, VEGFA, PIK3CA, RPS6KB1, EGLN1 | 2.40861 |
|  |  |  |  |  |  |
| hsa04520:Adherens junction | 10 | 1.032 | 0.01046 | TJP1, EP300, WASF3, FYN, WASF1, RAC1, RHOA, LMO7, SMAD2, SNAI2 | 2.71393 |
|  |  |  |  |  |  |
| hsa04350:TGF-beta signaling pathway | 11 | 1.1352 | 0.01099 | ACVR2A, INHBA, NOG, SMAD9, EP300, RHOA, SMAD2, SMURF2, RPS6KB1, SMURF1, ACVR1C | 2.52331 |
|  |  |  |  |  |  |
| hsa05220:Chronic myeloid leukemia | 10 | 1.032 | 0.01143 | E2F3, CDKN1B, CRKL, CTBP2, PIK3CB, CBL, PIK3CA, SHC1, IKBKB, PTPN11 | 2.67624 |
|  |  |  |  |  |  |
| hsa04725:Cholinergic synapse | 13 | 1.3416 | 0.01185 | PRKCA, KCNQ4, GNAI3, ADCY2, ADCY9, GNAQ, FYN, PIK3CB, BCL2, PIK3CA, CREB5, PRKACB, ITPR1 | 2.25672 |
|  |  |  |  |  |  |
| hsa04915:Estrogen signaling pathway | 12 | 1.2384 | 0.0129 | GNAI3, ADCY2, ADCY9, GNAQ, PIK3CB, JUN, PIK3CA, CREB5, SHC1, PRKACB, GABBR2, ITPR1 | 2.33563 |
|  |  |  |  |  |  |
| hsa04022:cGMP-PKG signaling pathway | 16 | 1.6512 | 0.01642 | ADCY2, GNAI3, ROCK2, CREB5, PRKG1, PPP1CB, IRS1, SRF, ITPR1, MEF2D, ATP2A2, GNAQ, ADCY9, GATA4, RHOA, GUCY1A3 | 1.95128 |
|  |  |  |  |  |  |
| hsa05100:Bacterial invasion of epithelial cells | 10 | 1.032 | 0.01869 | CRKL, PIK3CB, WASF1, GAB1, RAC1, CBL, RHOA, PIK3CA, SHC1, FN1 | 2.47037 |
|  |  |  |  |  |  |
| hsa04720:Long-term potentiation | 9 | 0.9288 | 0.01979 | PRKCA, RPS6KA3, EP300, GNAQ, GRIN2A, RAP1B, PRKACB, PPP1CB, ITPR1 | 2.62758 |
|  |  |  |  |  |  |
| hsa04010:MAPK signaling pathway | 22 | 2.2704 | 0.02115 | PRKCA, FGF18, NTF3, TAOK1, TAOK3, FGF12, SRF, MAP4K3, MAP4K4, MAP3K5, RPS6KA3, CRKL, DUSP1, ELK4, JUN, MAP3K1, RAC1, RAP1B, PRKACB, IKBKB, RAPGEF2, RASA2 | 1.67556 |
|  |  |  |  |  |  |
| hsa04115:p53 signaling pathway | 9 | 0.9288 | 0.0215 | ZMAT3, SIAH1, APAF1, PMAIP1, PTEN, SESN1, CDK2, TP73, SESN3 | 2.58836 |
|  |  |  |  |  |  |
| hsa04931:Insulin resistance | 12 | 1.2384 | 0.02342 | G6PC, RPS6KA3, PIK3CB, PIK3CA, CREB5, RPS6KB1, IKBKB, PTEN, IRS1, PPP1CB, CPT1A, PTPN11 | 2.14099 |
|  |  |  |  |  |  |
| hsa04144:Endocytosis | 21 | 2.1672 | 0.02411 | PARD6B, RAB7A, CHMP5, CYTH1, RAB4A, CBL, ASAP1, SMAD2, CYTH3, RAB11FIP2, RABEP1, IGF2R, GIT2, RHOA, SPG20, SMURF2, NEDD4L, SMURF1, WIPF1, AGAP1, SH3GL1 | 1.67903 |
|  |  |  |  |  |  |
| hsa04923:Regulation of lipolysis in adipocytes | 8 | 0.8256 | 0.02444 | GNAI3, ADCY2, ADCY9, PIK3CB, PIK3CA, PRKACB, PRKG1, IRS1 | 2.7527 |
|  |  |  |  |  |  |
| hsa04152:AMPK signaling pathway | 13 | 1.3416 | 0.02491 | PIK3CB, SCD, PPP2R5C, ADIPOR2, CREB5, RPS6KB1, IRS1, CPT1A, G6PC, PIK3CA, PPP2R5E, PPP2R2C, CCNA2 | 2.03655 |
|  |  |  |  |  |  |
| hsa05202:Transcriptional misregulation in cancer | 16 | 1.6512 | 0.02578 | CCNT2, BMI1, ERG, FLT1, AFF1, MYCN, DOT1L, FLI1, CDKN1B, SIN3A, REL, ELK4, SIX1, PBX3, ETV5, KLF3 | 1.84612 |
|  |  |  |  |  |  |
| hsa04024:cAMP signaling pathway | 18 | 1.8576 | 0.02714 | ADCY2, GNAI3, ROCK2, PIK3CB, GRIN2A, CREB5, GABBR2, PPP1CB, GLI3, EP300, ADCY9, JUN, RAC1, RHOA, PIK3CA, PTCH1, RAP1B, PRKACB | 1.75172 |
|  |  |  |  |  |  |
| hsa04660:T cell receptor signaling pathway | 11 | 1.1352 | 0.0334 | PAK6, PLCG1, PAK3, FYN, PIK3CB, JUN, RHOA, PIK3CA, GRAP2, IKBKB, TEC | 2.11958 |
|  |  |  |  |  |  |
| hsa04670:Leukocyte transendothelial migration | 12 | 1.2384 | 0.03514 | PRKCA, GNAI3, OCLN, PLCG1, ROCK2, PIK3CB, RAC1, RHOA, PIK3CA, RAP1B, MSN, PTPN11 | 2.01067 |
|  |  |  |  |  |  |
| hsa04914:Progesterone-mediated oocyte maturation | 10 | 1.032 | 0.03515 | RPS6KA3, GNAI3, ADCY2, ADCY9, PIK3CB, PIK3CA, PRKACB, CDC27, CCNA2, CDK2 | 2.21482 |
|  |  |  |  |  |  |
| hsa05231:Choline metabolism in cancer | 11 | 1.1352 | 0.03544 | DGKA, PRKCA, WASF3, PLCG1, PIK3CB, JUN, WASF1, RAC1, PIK3CA, DGKH, RPS6KB1 | 2.09859 |
|  |  |  |  |  |  |
| hsa05215:Prostate cancer | 10 | 1.032 | 0.03745 | E2F3, CDKN1B, EP300, PIK3CB, BCL2, PIK3CA, CREB5, IKBKB, PTEN, CDK2 | 2.18965 |
|  |  |  |  |  |  |
| hsa04270:Vascular smooth muscle contraction | 12 | 1.2384 | 0.03914 | PRKCA, ADCY2, ADCY9, GNAQ, ROCK2, PPP1R12B, RHOA, GUCY1A3, PRKACB, PRKG1, PPP1CB, ITPR1 | 1.9763 |
|  |  |  |  |  |  |
| hsa05166:HTLV-I infection | 21 | 2.1672 | 0.03926 | TLN1, E2F3, ADCY2, XIAP, TLN2, PIK3CB, SMAD2, CDC27, SRF, EP300, ADCY9, ETS1, ELK4, JUN, ETS2, MAP3K1, PIK3CA, PRKACB, IKBKB, MYB, TP53INP1 | 1.5931 |
|  |  |  |  |  |  |
| hsa04068:FoxO signaling pathway | 13 | 1.3416 | 0.04416 | G6PC, CDKN1B, EP300, PLK2, PIK3CB, FOXG1, SETD7, PIK3CA, SMAD2, IKBKB, PTEN, IRS1, CDK2 | 1.86937 |
|  |  |  |  |  |  |
| hsa04340:Hedgehog signaling pathway | 5 | 0.516 | 0.04826 | PTCH1, PRKACB, CSNK1G3, GLI3, FBXW11 | 3.56832 |
|  |  |  |  |  |  |
| hsa04390:Hippo signaling pathway | 14 | 1.4448 | 0.04853 | PARD6B, YWHAG, FRMD6, SOX2, YWHAB, YWHAQ, TEAD1, SMAD2, SNAI2, FBXW11, PPP2R2C, PPP1CB, TP73, LATS2 | 1.78652 |
|  |  |  |  |  |  |
| **REACTOME_PATHWAY** | |  |  |  |  |
| **Term** | **Count** | **%** | **PValue** | **Genes** | **Fold Enrichment** |
| R-HSA-2032785:YAP1- and WWTR1 (TAZ)-stimulated gene expression | 9 | 0.9288 | 8.8E-05 | TBL1XR1, CHD9, NCOA2, HIPK1, TBX5, HIPK2, GATA4, TEAD1, MED1 | 5.89201 |
|  |  |  |  |  |  |
| R-HSA-201451:Signaling by BMP | 8 | 0.8256 | 0.00012 | ACVR2A, NOG, CHRDL1, SMAD9, SMURF2, FSTL1, SMURF1, UBE2D1 | 6.6036 |
|  |  |  |  |  |  |
| R-HSA-194840:Rho GTPase cycle | 18 | 1.8576 | 0.00025 | DLC1, NGEF, FGD1, GDI2, TRIO, ARHGEF17, MYO9A, ARHGAP26, STARD13, ARHGAP6, ARHGAP20, SYDE1, RAC1, RHOA, RHOT1, CHN2, ARHGDIA, SRGAP1 | 2.75594 |
|  |  |  |  |  |  |
| R-HSA-4420097:VEGFA-VEGFR2 Pathway | 12 | 1.2384 | 0.00033 | WASF3, FYN, ROCK2, PIK3CB, WASF1, VEGFA, RAC1, RHOA, ABI2, PIK3CA, PRKACB, KDR | 3.67458 |
|  |  |  |  |  |  |
| R-HSA-1368082:RORA activates gene expression | 8 | 0.8256 | 0.00038 | TBL1XR1, CHD9, NCOA2, EP300, CLOCK, CPT1A, NRIP1, MED1 | 5.62529 |
|  |  |  |  |  |  |
| R-HSA-399954:Sema3A PAK dependent Axon repulsion | 6 | 0.6192 | 0.00106 | PLXNA4, PAK3, FYN, LIMK1, PLXNA2, RAC1 | 7.11951 |
|  |  |  |  |  |  |
| R-HSA-1368108:BMAL1:CLOCK,NPAS2 activates circadian gene expression | 9 | 0.9288 | 0.00156 | TBL1XR1, CHD9, NCOA2, EP300, NR3C1, BHLHE41, CLOCK, NRIP1, MED1 | 3.97368 |
|  |  |  |  |  |  |
| R-HSA-3928663:EPHA-mediated growth cone collapse | 8 | 0.8256 | 0.00197 | NGEF, FYN, ROCK2, EFNA1, VEGFA, RHOA, EFNA5, KDR | 4.33951 |
|  |  |  |  |  |  |
| R-HSA-1989781:PPARA activates gene expression | 15 | 1.548 | 0.00232 | CDK19, TBL1XR1, TEAD1, NFYA, MED13, ABCA1, CPT1A, RGL1, MED6, CHD9, EP300, NCOA2, NCOA3, CLOCK, MED1 | 2.52018 |
|  |  |  |  |  |  |
| R-HSA-381340:Transcriptional regulation of white adipocyte differentiation | 12 | 1.2384 | 0.00266 | MED6, CDK19, TBL1XR1, CHD9, NCOA2, EP300, NCOA3, CEBPD, MED13, KLF4, NCOR2, MED1 | 2.88385 |
|  |  |  |  |  |  |
| R-HSA-5625740:RHO GTPases activate PKNs | 7 | 0.7224 | 0.00286 | YWHAG, PPP1R12B, RAC1, RHOA, YWHAB, YWHAQ, PPP1CB | 4.74634 |
|  |  |  |  |  |  |
| R-HSA-400206:Regulation of lipid metabolism by PPARalpha | 6 | 0.6192 | 0.00315 | TBL1XR1, CHD9, SIN3A, NCOA2, NCOR2, MED1 | 5.69561 |
|  |  |  |  |  |  |
| R-HSA-983168:Antigen processing: Ubiquitination & Proteasome degradation | 29 | 2.9928 | 0.00317 | HECW2, TMEM189-UBE2V1, UBA6, UBE2V1, LMO7, RLIM, TRIM71, KLHL3, UBE2R2, CUL5, ARIH2, FBXW7, KBTBD6, KBTBD8, FBXW2, NEDD4L, UBE2D1, FBXL16, FBXO22, FBXW11, UBE2J1, GAN, CDC27, UBE2B, FBXO30, UBE2W, SMURF2, SIAH1, SMURF1 | 1.78758 |
|  |  |  |  |  |  |
| R-HSA-888590:GABA synthesis, release, reuptake and degradation | 5 | 0.516 | 0.00369 | SYT1, STX1A, GAD2, DNAJC5, SNAP25 | 7.30206 |
|  |  |  |  |  |  |
| R-HSA-400253:Circadian Clock | 9 | 0.9288 | 0.00369 | TBL1XR1, CHD9, MEF2D, NCOA2, EP300, PPP1CB, CLOCK, NRIP1, MED1 | 3.48711 |
|  |  |  |  |  |  |
| R-HSA-2424491:DAP12 signaling | 7 | 0.7224 | 0.00412 | PLCG1, FYN, PIK3CB, RAC1, PIK3CA, SHC1, GRAP2 | 4.42992 |
|  |  |  |  |  |  |
| R-HSA-2219530:Constitutive Signaling by Aberrant PI3K in Cancer | 10 | 1.032 | 0.00429 | FGF18, ERBB4, FYN, PIK3CB, GAB1, PIK3CA, NRG1, IRS1, FRS2, PTPN11 | 3.11235 |
|  |  |  |  |  |  |
| R-HSA-383280:Nuclear Receptor transcription pathway | 9 | 0.9288 | 0.00475 | NRBP1, THRA, NRBF2, THRB, ESRRG, NR3C1, NR5A2, NR2C2, MED1 | 3.35036 |
|  |  |  |  |  |  |
| R-HSA-399955:SEMA3A-Plexin repulsion signaling by inhibiting Integrin adhesion | 5 | 0.516 | 0.00495 | TLN1, PLXNA4, FYN, PLXNA2, RAC1 | 6.78048 |
|  |  |  |  |  |  |
| R-HSA-983231:Factors involved in megakaryocyte development and platelet production | 14 | 1.4448 | 0.00576 | MAFG, CDK2, CBX5, DOCK4, PRKAR2B, GATA2, EP300, SIN3A, RAC1, GATA4, PHF21A, ZFPM2, PRKACB, MYB | 2.37317 |
|  |  |  |  |  |  |
| R-HSA-2173795:Downregulation of SMAD2/3:SMAD4 transcriptional activity | 6 | 0.6192 | 0.00601 | TRIM33, SMAD2, SMURF2, NEDD4L, UBE2D1, NCOR2 | 4.9527 |
|  |  |  |  |  |  |
| R-HSA-4419969:Depolymerisation of the Nuclear Lamina | 5 | 0.516 | 0.00647 | PRKCA, LEMD3, CNEP1R1, LPIN2, LPIN1 | 6.32845 |
|  |  |  |  |  |  |
| R-HSA-5637810:Constitutive Signaling by EGFRvIII | 5 | 0.516 | 0.00647 | PLCG1, GAB1, CBL, PIK3CA, SHC1 | 6.32845 |
|  |  |  |  |  |  |
| R-HSA-210500:Glutamate Neurotransmitter Release Cycle | 6 | 0.6192 | 0.00727 | SYT1, SLC1A2, STX1A, SLC38A2, PPFIA1, SNAP25 | 4.74634 |
|  |  |  |  |  |  |
| R-HSA-1660499:Synthesis of PIPs at the plasma membrane | 7 | 0.7224 | 0.00903 | PIK3CB, SYNJ1, PIK3CA, PI4K2B, INPP4A, PIP4K2A, PTEN | 3.79707 |
|  |  |  |  |  |  |
| R-HSA-198203:PI3K/AKT activation | 4 | 0.4128 | 0.00956 | PIK3CB, RHOA, PIK3CA, IRS1 | 8.43794 |
|  |  |  |  |  |  |
| R-HSA-170968:Frs2-mediated activation | 4 | 0.4128 | 0.00956 | CRKL, PLCG1, YWHAB, FRS2 | 8.43794 |
|  |  |  |  |  |  |
| R-HSA-1257604:PIP3 activates AKT signaling | 11 | 1.1352 | 0.00962 | FGF18, ERBB4, FYN, PIK3CB, GAB1, PIK3CA, NRG1, PTEN, IRS1, FRS2, PTPN11 | 2.57826 |
|  |  |  |  |  |  |
| R-HSA-109704:PI3K Cascade | 7 | 0.7224 | 0.01037 | FGF18, PIK3CB, GAB1, PIK3CA, IRS1, FRS2, PTPN11 | 3.6916 |
|  |  |  |  |  |  |
| R-HSA-1433557:Signaling by SCF-KIT | 7 | 0.7224 | 0.01184 | PRKCA, FYN, RAC1, PIK3CA, GRAP2, TEC, PTPN11 | 3.59182 |
|  |  |  |  |  |  |
| R-HSA-5654710:PI-3K cascade:FGFR3 | 5 | 0.516 | 0.0128 | ROCK2, LIMK1, PPP1R12B, RHOA, PPP1CB | 5.27371 |
|  |  |  |  |  |  |
| R-HSA-5627117:RHO GTPases Activate ROCKs | 5 | 0.516 | 0.0128 | FGF18, GAB1, PIK3CA, FRS2, PTPN11 | 5.27371 |
|  |  |  |  |  |  |
| R-HSA-194306:Neurophilin interactions with VEGF and VEGFR | 3 | 0.3096 | 0.01541 | NRP2, FLT1, KDR | 14.239 |
|  |  |  |  |  |  |
| R-HSA-1236382:Constitutive Signaling by Ligand-Responsive EGFR Cancer Variants | 5 | 0.516 | 0.01556 | PLCG1, GAB1, CBL, PIK3CA, SHC1 | 4.99615 |
|  |  |  |  |  |  |
| R-HSA-2565942:Regulation of PLK1 Activity at G2/M Transition | 11 | 1.1352 | 0.0167 | PRKAR2B, NEDD1, TUBB, YWHAG, ACTR1A, PPP1R12B, CEP41, CLASP1, MAPRE1, FBXW11, PPP1CB | 2.37317 |
|  |  |  |  |  |  |
| R-HSA-180292:GAB1 signalosome | 4 | 0.4128 | 0.01735 | PLCG1, GAB1, PIK3CA, FRS2 | 6.90377 |
|  |  |  |  |  |  |
| R-HSA-1502540:Signaling by Activin | 4 | 0.4128 | 0.01735 | GAB1, PIK3CA, PAG1, PTPN11 | 6.90377 |
|  |  |  |  |  |  |
| R-HSA-5655291:Signaling by FGFR4 in disease | 4 | 0.4128 | 0.01735 | ACVR2A, INHBA, SMAD2, ACVR1C | 6.90377 |
|  |  |  |  |  |  |
| R-HSA-5654720:PI-3K cascade:FGFR4 | 5 | 0.516 | 0.01866 | FGF18, GAB1, PIK3CA, FRS2, PTPN11 | 4.74634 |
|  |  |  |  |  |  |
| R-HSA-3928662:EPHB-mediated forward signaling | 7 | 0.7224 | 0.02145 | FYN, ROCK2, LIMK1, EFNB2, RAC1, RHOA, SDC2 | 3.16423 |
|  |  |  |  |  |  |
| R-HSA-445095:Interaction between L1 and Ankyrins | 6 | 0.6192 | 0.02151 | SCN3B, ANK3, SCN2A, NFASC, SCN8A, SCN5A | 3.67458 |
|  |  |  |  |  |  |
| R-HSA-5218921:VEGFR2 mediated cell proliferation | 5 | 0.516 | 0.02211 | PRKCA, PLCG1, VEGFA, ITPR1, KDR | 4.52032 |
|  |  |  |  |  |  |
| R-HSA-5578768:Physiological factors | 4 | 0.4128 | 0.02225 | HIPK1, TBX5, HIPK2, GATA4 | 6.32845 |
|  |  |  |  |  |  |
| R-HSA-68911:G2 Phase | 3 | 0.3096 | 0.0248 | E2F3, CCNA2, CDK2 | 11.3912 |
|  |  |  |  |  |  |
| R-HSA-204005:COPII-mediated vesicle transport | 9 | 0.9288 | 0.02547 | SEC23A, PPP6C, SEC24A, ANKRD28, MCFD2, PPP6R3, NAPB, LMAN1, SAR1B | 2.51277 |
|  |  |  |  |  |  |
| R-HSA-912631:Regulation of signaling by CBL | 5 | 0.516 | 0.02593 | CRKL, FYN, PIK3CB, CBL, PIK3CA | 4.31485 |
|  |  |  |  |  |  |
| R-HSA-212676:Dopamine Neurotransmitter Release Cycle | 5 | 0.516 | 0.03013 | SYT1, STX1A, PPFIA1, CASK, SNAP25 | 4.12725 |
|  |  |  |  |  |  |
| R-HSA-5654695:PI-3K cascade:FGFR2 | 5 | 0.516 | 0.03013 | FGF18, GAB1, PIK3CA, FRS2, PTPN11 | 4.12725 |
|  |  |  |  |  |  |
| R-HSA-512988:Interleukin-3, Interleukin-5 and GM-CSF signaling | 5 | 0.516 | 0.0347 | PIK3CB, PIK3CA, SHC1, TEC, PTPN11 | 3.95528 |
|  |  |  |  |  |  |
| R-HSA-5627123:RHO GTPases activate PAKs | 5 | 0.516 | 0.0347 | PAK3, LIMK1, PPP1R12B, RAC1, PPP1CB | 3.95528 |
|  |  |  |  |  |  |
| R-HSA-4570464:SUMOylation of RNA binding proteins | 7 | 0.7224 | 0.03529 | BMI1, NUP153, HNRNPK, RNF2, CBX4, UBE2I, NUP35 | 2.82761 |
|  |  |  |  |  |  |
| R-HSA-5663213:RHO GTPases Activate WASPs and WAVEs | 6 | 0.6192 | 0.03857 | WASF3, WASF1, RAC1, ABI2, WIPF3, WIPF1 | 3.16423 |
|  |  |  |  |  |  |
| R-HSA-428540:Activation of RAC1 | 4 | 0.4128 | 0.04095 | PAK6, PAK3, RAC1, SLIT2 | 5.06276 |
|  |  |  |  |  |  |
| R-HSA-111447:Activation of BAD and translocation to mitochondria | 4 | 0.4128 | 0.04095 | YWHAG, BCL2, YWHAB, YWHAQ | 5.06276 |
|  |  |  |  |  |  |
| R-HSA-2173788:Downregulation of TGF-beta receptor signaling | 5 | 0.516 | 0.04498 | SMAD2, SMURF2, NEDD4L, SMURF1, PPP1CB | 3.65103 |
|  |  |  |  |  |  |
| R-HSA-5675221:Negative regulation of MAPK pathway | 6 | 0.6192 | 0.04719 | DUSP1, PPP2R5C, YWHAB, PPP2R5E, KSR1, PAQR3 | 2.99769 |
|  |  |  |  |  |  |
| R-HSA-392517:Rap1 signalling | 4 | 0.4128 | 0.04849 | PRKCA, FYN, CBL, SOCS6 | 4.74634 |
|  |  |  |  |  |  |
| R-HSA-1963642:PI3K events in ERBB2 signaling | 4 | 0.4128 | 0.04849 | ERBB4, GAB1, PIK3CA, NRG1 | 4.74634 |
|  |  |  |  |  |  |
| R-HSA-1433559:Regulation of KIT signaling | 4 | 0.4128 | 0.04849 | PRKAR2B, ADCY2, ADCY9, PRKACB | 4.74634 |
|  |  |  |  |  |  |
| R-HSA-163615:PKA activation | 4 | 0.4128 | 0.04849 | YWHAB, RAP1B, PRKACB, PRKG1 | 4.74634 |
|  |  |  |  |  |  |
| R-HSA-69563:p53-Dependent G1 DNA Damage Response | 3 | 0.3096 | 0.04856 | CDKN1B, CCNA2, CDK2 | 8.13658 |
